# New benzothieno[2,3-*c*]pyridines as non-steroidal CYP17 inhibitors: design, synthesis, anticancer screening, apoptosis induction, and *in silico* ADME profile studies

**DOI:** 10.1080/14756366.2021.1958212

**Published:** 2021-08-02

**Authors:** Nadia A. Khalil, Eman M. Ahmed, Ashraf F. Zaher, Eman A. Sobh, Samiha A. El-Sebaey, Mona S. El-Zoghbi

**Affiliations:** a Pharmaceutical Organic Chemistry Department, Faculty of Pharmacy, Cairo University, Cairo, Egypt; b Pharmaceutical Chemistry Department, Faculty of Pharmacy, Menoufia University, Menoufia, Egypt; c Pharmaceutical Organic Chemistry Department, Faculty of Pharmacy (Girls), Al-Azhar University, Cairo, Egypt

**Keywords:** Cancer, CYP17 enzyme inhibitors, apoptosis

## Abstract

A series of [1]benzothieno[2,3-*c*]pyridines was synthesised. Most compounds were chosen by NCI-USA to evaluate their anticancer activity. Compounds **5a–c** showed prominent growth inhibition against most cell lines. **5c** was selected at five dose concentration levels. It exhibited potent broad-spectrum anticancer activity with a GI_50_ of 4 nM–37 µM. Cytotoxicity of **5a–c** was further evaluated against prostate, renal, and breast cancer cell lines. **5c** showed double and quadruple the activity of staurosporine and abiraterone, respectively, against the PC-3 cell line with IC_50_ 2.08 µM. The possible mechanism of anti-prostate cancer was explored *via* measuring the CYP17 enzyme activity in mice prostate cancer models compared to abiraterone. The results revealed that **5c** suppressed the CYP17 enzyme to 15.80 nM. Moreover, it was found to be equipotent to abiraterone in testosterone production. Cell cycle analysis and apoptosis were performed. Additionally, the ADME profile of compound **5c** demonstrated both good oral bioavailability and metabolic stability.

## 1. Introduction

Prostate cancer remains a significant health problem that affects men worldwide, that is characterised by excessive uncontrolled growth of prostate gland cells^1^. It is considered the second leading cause of cancer deaths after lung cancer^2^^,^^3^. In 2019, the American Cancer Society estimated a 6% increase in prostate cancer cases and about a 7% increase in deaths from prostate cancer compared to the previous years^4^.

Serum Prostate-Specific Antigen (PSA) with Digital Rectal Exam (DRE) is the most widely used first-line test in urology for the detection of the risk of prostate cancer^5^. They can help to catch the disease at an early stage when treatment is thought to be more effective and potentially has fewer side effects^6^. However, no set cut-off point that can ensure whether a man has or hasn’t had prostate cancer, as high levels of PSA may be only observed after certain medical procedures, in the presence of infection or cases of non-cancerous overgrowth of the prostate, known as benign prostatic hyperplasia (BPH). Therefore, PSA tests may be useful as a signal for the need for a biopsy to examine the prostate cells and determine whether they are cancerous^7^.

There is no obvious reason for prostate cancer, however, family history, race, age, hormonal disturbances, and diet are known to be the most common risk factors^8^. Therapeutic management of prostate cancer includes radiation and prostatectomy for early stages (**I**, **II**)^9^^,^^10^. Moreover, Androgen Deprivation Therapy (ADT) reduces the level of androgen hormones in the prostate, thereby preventing the growth of cancer cells. However, this approach should be accompanied by radiation therapy to ensure the eradication of cancer inside the prostate^11^^,^^12^. In stage **III**, the level of androgen hormones could be reduced by surgery (orchiectomy or surgical castration)^13^, medication (chemical castration that reduces LH hormone production in the pituitary gland)^14^^,^^15^ and hormonal therapy or antiandrogens (that prevent androgen receptors (AR) in the prostate to bind to testosterone hormone)^16^. Advanced prostate cancer cases that persist even after reduction of the testosterone level are known as castrate-resistant prostate cancer (CRPC)^17^. This type of prostate cancer needs chemotherapeutic agents that work by total blockage of androgen biosynthesis *via* inhibition of CYP17.

**Figure 1.**
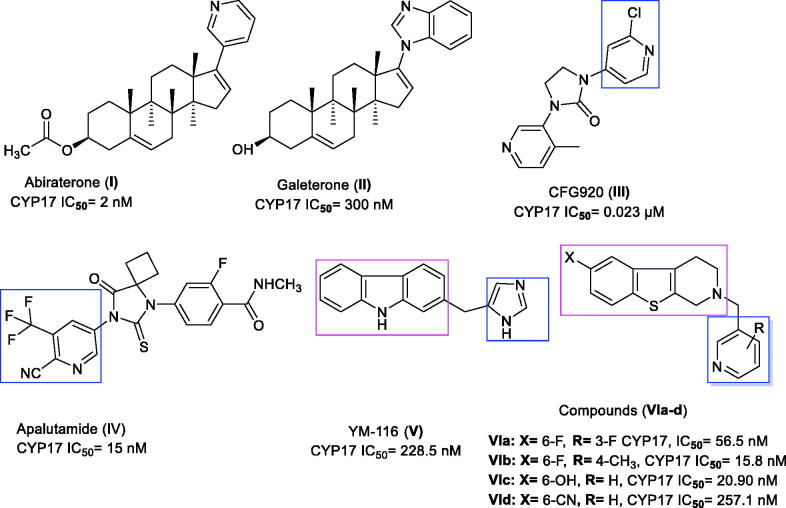
Certain active CYP17 inhibitors.

Several categories of steroidal and non-steroidal CYP17 inhibitors were developed and characterised as an effective treatment of advanced prostate cancer cases^18^. Such include abiraterone acetate **I**, a steroidal antiandrogen prodrug, which was described for the treatment of metastatic castration-resistant prostate cancer (mCRPC)^19^, however, it showed undesirable side effects as a result of its non-selectivity. The solution to this problem came in the concomitant administration of prednisone. Galeterone **II** is an investigational steroidal antiandrogen drug used for the treatment of mCRPC cases that acts by a dual mechanism^20^. The first involves AR antagonism, thereby preventing the testosterone from binding to its receptor, while the second mechanism is inhibition of CYP17 that reduces the synthesis of androgens, being more specific to 17α-lyase than 17α-hydrolyase^21^. CFG920 **III** is a new CYP17 inhibitor in phase I clinical trial used in CRPC patients who are abiraterone resistant^22^. A very recent drug approved by FDA in 2018 is apalutamide **IV,** to be used for advanced prostate cancer cases by preventing testosterone hormone from binding to androgen receptor^23^. Compounds **Va–e** bearing various bicyclic fused ring systems were reported to have potent CYP17 inhibitory activity with IC_50_ values ranging from 16–95 nM, except **Vc** displayed moderate inhibition. The most promising scaffold was **Ve** with a benzothiophene core in both *vitro* and *vivo* evaluation^24^. The selective CYP17 inhibitors include **YM116 VI**, which has a tricyclic fused ring system, which showed a loss in rat prostatic weight by reducing androgen production in the testes and adrenal glands^25^. Literature survey on non-steroidal prostate cancer inhibitors demonstrated that the tetrahydrobenzo[4,5]thieno[2,3-*c*]pyridine derivatives, **VIIa–d**, exhibited a comparable potency to abiraterone in inhibiting rat CYP17 enzyme and were able to decrease plasma testosterone level in a dose-dependent manner^26^ (Figure 1).

**Figure 2.**
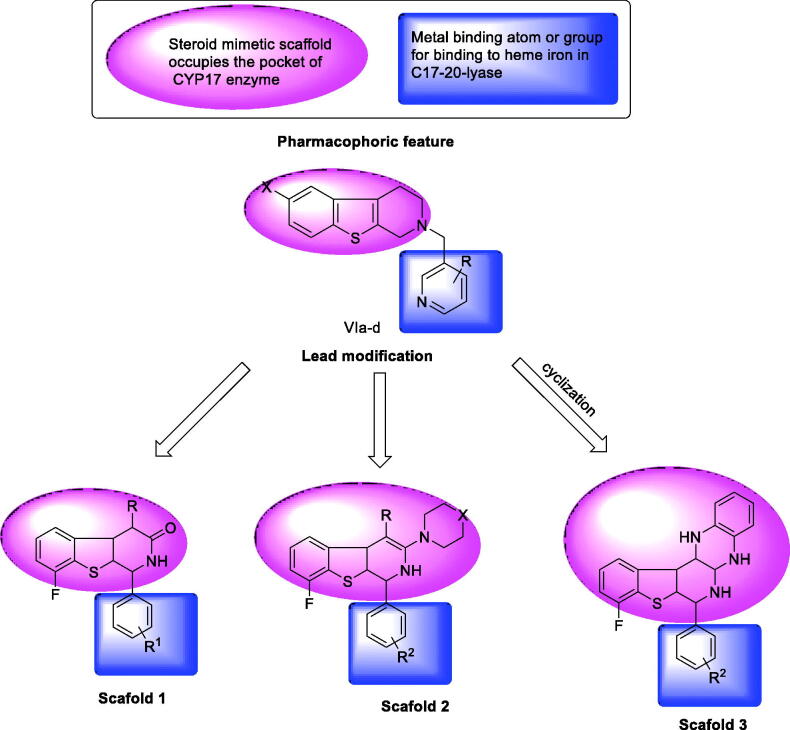
Rational of molecular design of new CYP17 inhibitors.

Due to the previously described side effects observed in steroidal drugs^27^^,^^28^, we were inspired to synthesise more specific and safer inhibitors against CYP17 lyase enzyme that could be able to block adrenal androgens and testosterone synthesis. SAR studies of some previously reported non-steroidal CYP17 inhibitors observe that most compounds belonging to this category consist of two parts essential for enzyme inhibition; one is the metal-binding atom or group, which should contain a lone pair of electrons, which is important for binding to haem iron in CYP17, while the second part involves the steroid mimetic scaffold, such as stilbene and biphenyl, or fused ring systems, such as naphthalene, benzothiophene, and 9*H*-carbazole occupying the pocket of CYP17 enzyme. Previously reported literature studies in this field, discovered the excellent CYP17 inhibitory effect of benzothienopyridine derivatives **VIIa–d**, making them promising leads for our design strategy (Figure 2).

This work comprises the synthesis of new series of 1,2,3,4-tetrahydro[1]benzothieno[2,3-*c*]pyridines as a promising candidate for CYP17 inhibition. Our strategy involves the replacement of a steroid nucleus by 8-fluoro-1,2-dihydro[1]benzothieno[2,3-*c*]pyridine core bearing phenyl ring at position 1, as a structural analog for compounds **VIIa–d**. In addition, groups or substituents bearing lone pair of electrons were present on the phenyl ring at C-_1_, representing a fundamental requirement for the enzyme inhibition, as the free electron, lone pairs are essential to interact with haem iron in CYP17, besides the lone pair of electrons, which is present on the nitrogen atom of benzothienopyridine scaffold (Figure 2).

Twenty-seven of the newly synthesised compounds were subjected to *in-vitro* anticancer screening by National Cancer Institute (USA) against 60 cell lines at one dose concentration followed by a five-dose screening for the most active candidate **5c**. The most active compounds were selected for measuring their IC_50_ against prostate cancer cell line (PC-3), renal cancer cell line (UO-31), and breast cancer cell line (MCF-7). Moreover, compound **5c** was evaluated *in-vivo* for inhibition of the CYP17 enzyme and gauging plasma testosterone level. Furthermore, the mechanism of action of compound **5c** was studied on the cell cycle of PC-3 cells and induction of apoptosis. Finally, the ADME profile of compound **5c** has been examined to investigate its potential as a promising drug candidate.

## 2. Experimental section

### 2.1. Chemistry

#### 2.1.1. General

All chemicals and reagents were obtained from Aldrich (Sigma-Aldrich) and used without further purification. Reactions were monitored by TLC, performed on silica gel glass plates containing 60 GF-254, and visualised on TLC using UV light or iodine indicator. IR spectra were determined on Shimadzu IR 435 spectrophotometer (KBr, cm^−1^). ^1^H-NMR and ^13^C-NMR spectra were carried out using Bruker 400 and 100 MHz spectrophotometers, respectively, using TMS as internal standards. Chemical shifts were recorded in ppm on *δ* scale, Microanalytical Centre, Faculty of Pharmacy, Cairo University, Egypt. Mass spectra and elemental analyses were recorded on Shimadzu Qp-2010 plus spectrometer at the Regional Centre for Mycology and Biotechnology, Al-Azhar University, Cairo, Egypt. The results correspond to the calculated values within experimental error. Melting points were determined with the Stuart apparatus and are uncorrected.

#### 2.1.2. General procedure for the synthesis of 2-arylidene-7-fluoro[1]benzothio-phen-3(2H)-ones (2a–d)

7-Fluoro[1]benzothiophen-3-(2*H*)one **(1)** (1.68 g, 0.01 mol) was added to a solution of an appropriate aromatic aldehyde (0.01 mmol) in glacial acetic acid (20 ml) containing anhydrous sodium acetate (0.82 g, 0.01 mol), and the reaction mixture was heated under reflux for 2 h. The solvent was concentrated under reduced pressure, and the resulting solid product was dried then crystallised from acetonitrile.

##### 2.1.2.1. 2-(2,4-Dimethoxybenzylidene)-7-fluoro[1]benzothiophen-3(2H)-one (2a)

Yield 77%, mp 195–197 °C, IR (KBr, cm^−1^): 3062 (C-H aromatic), 2951, 2813 (C-H aliphatic), 1670 (C=O), 1604 (C=C). ^1^H-NMR (DMSO-*d_6_*, 400 MHz, δ ppm): 3.92 (s, 3H, CH_3_O), 3.96 (s, 3H, CH_3_O), 6.66 (d, 1H, *J* = 8.30 Hz, 2,4-(CH_3_O)_2_-C_6_H_3-_C_5_-H), 6.72 (s, 1H, 2,4-(CH_3_O)_2_-C_6_H_3_-C_3_-H), 6.80 (d, 1H, *J* = 8.30 Hz, 2,4-(CH_3_O)_2_-C_6_H_3_-C_6_-H), 7.43–7.52 (m, 1H, [1]benzothiophene-C_5_-H), 7.64–7.73 (m, 1H, [1]benzothiophene-C_4_-H), 7.76 (d, 1H, *J* = 7.56 Hz, [1]benzothiophene-C_6_-H), 8.27 (s, 1H, benzylidene-H).^13^C-NMR (DMSO-*d_6_*, 100 MHz, δ ppm): 56.28, 56.44 (OCH_3_)_2_, 98.63, 99.15, 107.24, 107.33 (d, *J* = 18 Hz, C-_5_) 115.22, 118.59, 123.26, 125.86, 129.66, 130.28, 131.51, 132.62, 161.32, 165.25 (d, *J* = 260 Hz, C-F) (ArCs), 187.68 (C=O). Anal. Calcd. (%) for C_17_H_13_FO_3_S (316): C, 64.54, H, 4.14. Found: C, 64.78, H 4.37.

##### 2.1.2.2. 7-Fluoro-2-(4-methoxybenzylidene)[1]benzothiophen-3(2H)-one (2b)

Was previously reported^29^.

##### 2.1.2.3. 7-Fluoro-2-(2-nitrobenzylidene)[1]benzothiophen-3(2H)-one (2c)

Yield 70%, mp 170–172 °C, IR (KBr, cm^−1^): 3059, 3022 (C-H aromatic), 1689 (C=O), 1593 (C=C), 1519, 1346 (NO_2_). ^1^H-NMR (DMSO-*d_6_*, 400 MHz, δ ppm): 7.49–7.54 (m, 1H, [1]benzothiophene-C_4_-H), 7.72 (t, 1H, *J* = 8.76 Hz, [1]benzothiophene-C_5_-H), 7.76–7.78 (m, 1H, [1]benzothiophene-C_6_-H), 7.81 (d, 1H, *J* = 7 Hz, 2-NO_2_-C_6_H_4_-C_6_-H), 7.93–7.96 (m, 2H, 2-NO_2_-C_6_H_4_-C_4,5_-H), 8.15 (d, 1H, *J* = 7 Hz, 2-NO_2_-C_6_H_4_-C_3_-H), 8.26 (s, 1H, benzylidene C-H). ^13^C-NMR (DMSO-*d_6_*, 100 MHz, δ ppm): 122.95, 123.58, 124.78, 125.99, 128.92, 128.95 (d, *J* = 6 Hz, C-_5_), 129.61, 130.60, 131.87 (d, *J* = 255 Hz, C-F), 133.15, 134.65, 134.74, 134.99, 148.81 (ArCs), 190.41 (C=O). Anal. Calcd. (%) for C_15_H_8_FNO_3_S (301): C, 59.80, H, 2.68, S 10.64, N 4.65. Found: C, 59.63, H, 3.02, S, 10.75, N, 4.89.

##### 2.1.2.4. 7-Fluoro-2-(3-nitrobenzylidene)[1]benzothiophen-3(2H)-one (2d)

Yield 83%, mp 200–202 °C, IR (KBr, cm^−1^): 3070, 3001 (C-H aromatic), 1681 (C=O), 1589 (C=C), 1527, 1354 (NO_2_). ^1^H-NMR (DMSO-*d_6_*, 400 MHz, δ ppm): 7.52–8.00 (m, 3H, [1]benzothiophene-C_4,5,6_-H), 8.16–8.22 (m, 1H, 3-NO_2_-C_6_H_4_-C_5_-H), 8.23–8.42 (m, 2H, 3-NO_2_-C_6_H_4_-C_4,6_-H), 8.55 (s, 1H, 3-NO_2_-C_6_H_4_-C_2_-H), 8.70 (s, 1H, benzylidene C-H). ^13^C-NMR (DMSO-*d_6_*, 100 MHz, δ ppm): 123.56, 124.55, 125.86, 129.04, 131.44, 131.54 (d, *J* = 20 Hz, C-_5_), 132.41, 133.89 (d, *J* = 260 Hz, C-F), 135.38, 137.66, 139.38, 140.25, 148.76, 157.58 (ArCs), 192.32 (C=O). Anal. Calcd. (%) for C_15_H_8_FNO_3_S (301): C, 59.80, H, 2.68, N, 4.65. Found: C, 60.13, H, 2.89, N, 4.76.

#### 2.1.3. General procedure for synthesis of 1-aryl-8-fluoro-1,2-dihydro[1]benzo-thieno[2,3-c]pyridin-3(4H)-ones (3a–d)

A mixture of α,β-unsaturated ketones **2a–d** (1 mmol), acetamide (0.118 g, 0.002 mol), and potassium hydroxide (0.28 g, 0.005 mol) in 95% ethanol (25 ml) was heated under reflux for 8 h. The reaction mixture was allowed to cool and poured onto ice-cooled water. The precipitated solid was filtered and crystallised from ethanol to give the target compounds **3a–d**.

##### 2.1.3.1. 1-(2,4-Dimethoxyphenyl)-8-fluoro-[1]benzothieno[2,3-c]pyridin-3(4H)-one (3a)

Yield 55%, mp 70–72 °C, IR (KBr, cm^−1^): 3417 (OH, taut), 3313 (NH), 3078 (C-H aromatic), 2943–2889 (C-H aliphatic), 1681 (C=O), 1604 (C=N), 1570 (C=C). ^1^H-NMR (DMSO-*d_6_*, 400 MHz, δ ppm): 3.84 (s, 2H, CH_2_), 3.87 (s, 3H, OCH_3_), 3.91 (s, 3H, OCH_3_), 3.95 (s, 1H, C_1_-H), 6.65 (d, 1H, *J* = 8.20 Hz, 2,4-(OCH_3_)_2_-C_6_H_3_-C_5_-H), 6.69 (s, 1H, 2,4-(OCH_3_)_2_-C_6_H_3_-C_3_-H), 6.78 (d, 1H, *J* = 8.20 Hz, 2,4-(OCH_3_)_2_-C_6_H_3_-C_6_-H), 7.30 (d, 1H, *J* = 8.40 Hz, C_5_-H), 7.40–7.52 (m, 1H, C_6_-H), 7.67 (d, 1H, *J* = 8.40 Hz, C_7_-H), 8.26 (s, 1H, NH, D_2_O exchangeable). ^13^C-NMR (DMSO-*d_6_*, 100 MHz, δ ppm): 31.72 (CH_2_), 55.92 (C-_1_), 56.28, 56.44 (OCH_3_)_2_, 98.63, 99.15, 99.23 (d, *J* = 17 Hz, C-_6_), 107.24, 115.21, 118.59, 122.97, 125.67, 128.46, 129.67, 130.28, 131.50, 165.25 (d, *J* = 260 Hz, C-F), 166.55 (ArCs), 187.86 (C=O). Anal. Calcd. (%) for C_19_H_16_FNO_3_S (357): C, 63.85, H, 4.51, N, 3.92. Found: C, 63.50, H, 4.74, N, 4.25.

##### 2.1.3.2. 8-Fluoro-1-(4-methoxyphenyl)[1]benzothieno[2,3-c]pyridin-3(4H)-one (3b)

Yield 65%, mp 150–152 °C, IR (KBr, cm^−1^): 3336 (NH), 3070 (C-H aromatic), 2843 (C-H aliphatic), 1674 (C=O), 1604 (C=N), 1581 (C=C). ^1^H-NMR (DMSO-*d_6_*, 400 MHz, δ ppm): 3.79 (s, 2H, CH_2_), 3.84 (s, 1H, C_1_-H), 3.87 (s, 3H, OCH_3_), 7.16 (d, 2H, *J* = 8.56 Hz, 4-OCH_3_-C_6_H_4_-C_3,5_-H), 7.49 (d, 2H, *J* = 8.56 Hz, 4-OCH_3_-C_6_H_4_-C_2,6_-H), 7.68 (t, 1H, *J* = 8 Hz, C_6_-H), 7.77 (d, 1H, *J* = 8 Hz, C_5_-H), 7.82 (d, 1H, *J* = 8 Hz, C_7_-H), 8.01 (s, 1H, NH, D_2_O exchangeable). ^13^C-NMR (DMSO-*d_6_*, 100 MHz, δ ppm): 36.75 (CH_2_), 55.77 (OCH_3_), 56.03 (C-_1_), 114.76, 115.52, 120.14, 121.85, 122.03, 122.99, 126.07, 126.18 (d, *J* = 23 Hz, C-_6_), 128.56, 132.36 (d, *J* = 266 Hz, C-F), 133.69, 135.38, (ArCs), 186.70 (C=O). Anal. Calcd. (%) for C_18_H_14_FNO_2_S (327): C, 66.04, H, 4.31, N, 4.28. Found: C, 65.87, H, 4.53, N, 4.67.

##### 2.1.3.3. 8-Fluoro-1-(2-nitrophenyl)[1]benzothieno[2,3-c]pyridin-3(4H)-one (3c)

Yield 70% mp 160–162 °C, IR (KBr, cm^−1^): 3332 (NH), 3071 (C-H aromatic), 2951 (C-H aliphatic), 1681 (C=O), 1589 (C=N), 1570 (C=C), 1527, 1342 (NO_2_). ^1^H-NMR (DMSO-*d_6_*, 400 MHz, δ ppm): 3.00 (s, 2H, CH_2_), 3.92 (s, 1H, C_1_-H), 6.67–7.28 (m, 3H, C_5,6,7_-H), 7.30–7.65 (m, 3H, 2-NO_2_-C_6_H_4_-C_4,5,6_-H), 7.67 (s, 1H, NH, D_2_O exchangeable), 7.97 (d, 1H, *J* = 8.28 Hz, 2-NO_2_-C_6_H_4_-C_3_-H). ^13^C-NMR (DMSO-*d_6_*, 100 MHz, δ ppm): 37.10 (CH_2_), 56.40 (C-_1_), 113.19, 115.46, 116.78, 121.10, 122.91, 124.12, 124.55, 127.07, 128.21, 129.76, 131.77, 132.79, 135.56 (d, *J* = 276 Hz, C-F), 136.94 (ArCs), 189.62 (C=O). Anal. Calcd. (%) for C_17_H_11_FN_2_O_3_S (342): C, 59.64, H, 3.24, N, 8.18, S, 9.37. Found: C, 59.87, H, 3.51, N, 7.95, S, 9.12.

##### 2.1.3.4. 8-Fluoro-1-(3-nitrophenyl)[1]benzothieno[2,3-c]pyridin-3(4H)-one (3d)

Yield 80%, mp 110–112 °C, IR (KBr, cm^−1^): 3468 (OH, taut), 3360 (NH), 3075 (C-H aromatic), 2927 (C-H aliphatic), 1685 (C=O), 1616 (C=N), 1595 (C=C), 1527, 1350 (NO_2_). ^1^H-NMR (DMSO-*d_6_*, 400 MHz, δ ppm): 3.93 (s, 2H, CH_2_), 4.19 (s, 1H, C_1_-H), 7.15–7.92 (m, 4H, C_5,6,7_-H and 3-NO_2_-C_6_H_4_-C_5_-H), 8.05 (d, 1H, *J* = 8 Hz, 3-NO_2_-C_6_H_4_-C_6_-H), 8.53 (d, 1H, *J* = 8 Hz, 3-NO_2_-C_6_H_4_-C_4_-H), 8.60 (s, 1H, 3-NO_2_-C_6_H_4_-C_2_-H), 8.63 (s, 0.5H, NH, D_2_O exchangeable, taut), 8.70 (s, 0.5H, OH, D_2_O exchangeable, taut). ^13^C-NMR (DMSO-*d_6_*, 100 MHz, δ ppm): 32.17 (C-_1_), 62.20 (C-_4_), 122.02, 124.44, 124.84, 125.93, 127.92, 128.80, 128.83 (d, *J* = 6 Hz, C-_6_), 130.06, 131.41, 132.40, 134.66 (d, *J* = 266 Hz, C-F), 135.99, 145.42, 148.23 (ArCs), 192.27 (C=O). MS: *m/z* (% relative abundance): 342 (M^+.^, 20.52%) and 300 (100%). Anal. Calcd. (%) for C_17_H_11_FN_2_O_3_S (342): C, 59.64, H, 3.24, N, 8.18. Found: C, 59.79, H, 3.50, N, 8.40.

#### 2.1.4. General procedure for synthesis of 1-aryl-4-chloro-8-fluoro-1,2-dihydro [1]benzothieno[2,3-c]pyridine-3(4H)-ones (4a–d)

To a solution of α,β-unsaturated ketones **2a–d** (1 mmol) and potassium hydroxide (0.28 g, 0.005 mol) in 95% ethanol (25 ml), 2-chloroacetamide (0.187 g, 0.002 mol) was added, and the reaction mixture was heated under reflux for 8 h. After cooling, the mixture was poured on water, and the obtained solid was filtered off and crystallised from ethanol.

##### 2.1.4.1. 4-Chloro-1-(2,4-Dimethoxyphenyl)-8-fluoro-1,2-dihydro[1]benzothieno [2,3-c]pyridine-3(4H)-one (4a)

Yield 60%, mp 120–122 °C, IR (KBr, cm^−1^): 3441 (OH, taut), 3332 (NH), 3078 (C-H aromatic), 2943 (C-H aliphatic), 1681 (C=O), 1604 (C=N), 1566 (C=C). ^1^H-NMR (DMSO-*d_6_*, 400 MHz, δ ppm): 3.82 (s, 1H, C_1_-H), 3.88 (s, 3H, CH_3_O), 3.92 (s, 3H, CH_3_O), 3.95 (s, 1H, C_4_-H), 6.65 (d, 1H, *J* = 8.20 Hz, 2,4-(CH_3_O)_2_-C_6_H_3_-C_5_-H), 6.69 (s, 1H, 2,4-(CH_3_O)_2_-C_6_H_3_-C_3_-H), 6.78 (d, 1H, *J* = 8.20 Hz, 2,4-(CH_3_O)_2_-C_6_H_3_-C_6_-H), 7.40–7.52 (m, 1H, C_6_-H), 7.67 (d, 1H, *J* = 8.50 Hz, C_5_-H), 7.74–7.76 (m, 1H, C_7_-H), 8.26 (s, 0.5H, NH, D_2_O exchangeable, taut), 10.18 (s, 0.5H, OH, D_2_O exchangeable, taut). ^13^C-NMR (DMSO-*d_6_*, 100 MHz, δ ppm): 56.20 (C-_1_), 56.27, 56.43 (OCH_3_)_2_, 56.55 (C-_4_), 98.84 (d, *J* = 47 Hz, C-_6_), 99.08, 107.34, 115.20, 118.58, 121.62, 122.90, 125.61, 128.33, 129.59, 130.27, 131.46, 162.76 (d, *J* = 290 Hz, C-F), 164.24, 166.53 (ArCs), 186.67 (C=O). Anal. Calcd. (%) for C_19_H_15_ClFNO_3_S (391): C, 58.24, H, 3.86, N, 3.57. Found: C, 58.53, H, 4.10, N, 3.80.

##### 2.1.4.2. 4-Chloro-8-fluoro-1-(4-methoxyphenyl)-1,2-dihydro[1]benzothieno[2,3-c] pyridine-3(4H)-one (4b)

Yield 50%, mp 154–156 °C, IR (KBr, cm^−1^): 3441 (OH, taut), 3380 (NH), 3039 (C-H aromatic), 2880 (C-H aliphatic), 1674 (C=O), 1604 (C=N), 1585 (C=C). ^1^H-NMR (DMSO-*d_6_*, 400 MHz, δ ppm): 3.78 (s, 1H, C_1_-H), 3.85 (s, 1H, C_4_-H), 3.87 (s, 3H, CH_3_O), 7.16 (d, 2H, *J* = 8.30 Hz, 4-CH_3_O-C_6_H_4_-C_3,5_-H), 7.49 (d, 2H, *J* = 8.30 Hz, 4-OCH_3_-C_6_H_4_-C_2,6_-H), 7.68 (t, 1H, *J* = 8 Hz, C_6_-H), 7.76 (d, 1H, *J* = 8 Hz, C_5_-H), 7.82 (d, 1H, *J* = 8 Hz, C_7_-H), 8.01 (s, 1H, NH, D_2_O exchangeable). ^13^C-NMR (DMSO-*d_6_*, 100 MHz, δ ppm): 55.82 (C-_1_), 56.07 (OCH_3_), 56.16 (C-_4_), 114.98, 115.59, 121.93, 122.11, 123.07, 126.11, 126.22 (d, *J* = 22 Hz, C-_6_), 128.57, 132.27, 132.44 (d, *J* = 260 Hz, C-F), 135.46, 162.09 (ArCs), 191.78 (C=O). Anal. Calcd. (%) for C_18_H_13_ClFNO_2_S (361): C, 59.75, H, 3.62, N, 3.87. Found: C, 59.91, H, 3.84, N, 4.05.

##### 2.1.4.3. 4-Chloro-8-fluoro-1-(2-nitrophenyl)-1,2-dihydro[1]benzothieno[2,3-c] pyridine-3(4H)-one (4c)

Yield 62%, mp 110–112 °C, IR (KBr, cm^−1^): 3332 (NH), 3070 (C-H aromatic), 2904, 2835(C-H aliphatic), 1681 (C=O), 1550 (C=C), 1527, 1342 (NO_2_). ^1^H-NMR (DMSO-*d_6_*, 400 MHz, δ ppm): 3.95 (s, 1H, C_1_-H), 4.16 (s, 1H, C_4_-H), 7.21 (d, 1H, *J* = 8.40 Hz, C_5_-H), 7.18–7.25 (m, 1H, C_6_-H), 7.30 (d, 1H, *J* = 8.40 Hz, C_7_-H), 7.40–7.52 (m, 1H, 2-NO_2_-C_6_H_4_-C_5_-H), 7.56–7.60 (m, 1H, 2-NO_2_-C_6_H_4_-C_4_-H), 7.67 (d, 1H, *J* = 7.70 Hz, 2-NO_2_-C_6_H_4_-C_6_-H), 7.83 (d, 1H, *J* = 7.70 Hz, 2-NO_2_-C_6_H_4_-C_3_-H), 8.60 (s, 1H, NH, D_2_O exchangeable). ^13^C-NMR (DMSO-*d_6_*, 100 MHz, ppm): 54.61 (C-_1_), 56.17 (C-_4_), 124.00, 126.68, 127.92, 128.88, 129.14, 129.90, 130.04, 130.21 (d, *J* = 30 Hz, C-_6_), 130.90, 131.00, 131.05 (d, *J* = 223 Hz, C-F), 131.90, 131.99, 132.13 (ArCs), 181.68 (C=O). Anal. Calcd. (%) for C_17_H_10_ClFN_2_O_3_S (376): C, 54.11, H, 2.68, N, 7.43, S, 8.51. Found: C, 54.36, H, 2.85, N, 7.60, S, 8.64.

##### 2.1.4.4. 4-Chloro-8-fluoro-1-(3-nitrophenyl)-1,2-dihydro[1]benzothieno[2,3-c] pyridine-3(4H)-one (4d)

Yield 85%, mp 160–162 °C, IR (KBr, cm^−1^): 3390 (NH), 3078 (C-H aromatic), 2927(C-H aliphatic), 1685 (C=O), 1600 (C=N), 1560 (C=C), 1527, 1350 (NO_2_). ^1^H-NMR (DMSO-*d_6_*, 400 MHz, δ ppm): 3.81 (s, 1H, C_1_-H), 3.93 (s, 1H, C_4_-H), 7.20–8.15 (m, 4H, C_5,6,7_-H and 3-NO_2_-C_6_H_4_-C_5_-H), 8.34 (d, 1H, *J* = 7.50 Hz, 3-NO_2_-C_6_H_4_-C_6_-H), 8.53 (d, 1H, *J* = 7.50 Hz, 3-NO_2_-C_6_H_4_-C_4_-H), 8.54 (s, 1H, 3-NO_2_-C_6_H_4_-C_2_-H), 8.70 (s, 1H, NH, D_2_O exchangeable). ^13^C-NMR (DMSO-*d_6_*, 100 MHz, δ ppm): 51.51 (C-_1_), 58.68 (C-_4_), 118.11, 120.21, 122.00, 124.39, 124.59, 128.89 (d, *J* = 10 Hz, C-_6_), 130.03, 130.15 (d, *J* = 243 Hz, C-F), 131.37, 135.96, 136.05, 137.58, 145.71, 148.67 (ArCs), 192.19 (C=O). MS: *m/z* (% relative abundance): 378 (M^+.^+2, 6.38%), 376 (M^+.^, 14.45%) and 329 (100%). Anal. Calcd. (%) for C_17_H_10_ClFN_2_O_3_S (376): C, 54.11, H, 2.68, N, 7.43. Found: C, 54.35, H, 2.43, N, 7.72.

#### 2.1.5. General procedure for synthesis of 1-aryl-4-cyano-8-fluoro-1,2-dihydro [1]benzothieno[2,3-c]pyridin-3(4H)-ones (5a–d)

A mixture of α,β-unsaturated ketones **2a–d** (1 mmol), 2-cyanoacetamide (0.168 g, 0.002 mol), and potassium hydroxide (0.28 g, 0.005 mol) in 95% ethanol (25 ml) was heated under reflux for 8 h. The reaction mixture was poured on water after cooling, and the produced solid was filtered and crystallised from ethanol to afford the target compounds **5a–d**.

##### 2.1.5.1. 1-(2,4-Dimethoxyphenyl)-4-cyano-8-fluoro-1,2-dihydro[1]benzothieno [2,3-c]pyridin-3(4H)-one (5a)

Yield 75%, mp 100–102 °C, IR (KBr, cm^−1^): 3379 (OH, taut), 3313 (NH), 3078 (C-H aromatic), 2943 (C-H aliphatic), 2210 (CN), 1681 (C=O), 1604 (C=N), 1570 (C=C). ^1^H-NMR (DMSO-*d_6_*, 400 MHz, δ ppm): 3.75 (s, 3H, CH_3_O), 3.78 (s, 3H, CH_3_O), 3.89 (s, 1H, C_4_-H), 3.95 (s, 1H, C_1_-H), 6.40 (s, 1H, 2,4-(CH_3_O)_2_-C_6_H_3_-C_3_-H), 6.45–6.53 (m, 1H, 2,4-(CH_3_O)_2_-C_6_H_3_-C_5_-H), 6.78–6.83 (m, 1H, 2,4-(CH_3_O)_2_-C_6_H_3_-C_6_-H), 7.19–7.39 (m, 1H, C_6_-H), 7.42–7.59 (m, 1H, C_5_-H), 7.71 (d, 1H, *J* = 8.50 Hz, C_7_-H), 8.27 (s, 0.5H, NH, D_2_O exchangeable, taut), 10.17 (s, 0.5H, OH, D_2_O exchangeable, taut). ^13^C-NMR (DMSO-*d_6_*, 100 MHz, δ ppm): 33.46 (C-_4_), 55.49 (C-_1_), 56.22, 56.57 (OCH_3_)_2_, 99.11, 107.39, 108.69, 115.63, 118.23, 119.44, 122.91, 125.68, 128.46, 129.64, 131.48, 132.44, 145.92 (d, *J* = 268 Hz, C-F), 149.26, 155.15 (ArCs and CN), 180.29 (C=O). Anal. Calcd. (%) for C_20_H_15_FN_2_O_3_S (382): C, 62.82, H, 3.95, N, 7.33. Found: C, 62.71, H, 4.23, N, 7.62.

##### 2.1.5.2. 4-Cyano-8-fluoro-1-(4-methoxyphenyl)-1,2-dihydro[1]benzothieno[2,3-c] pyridin-3(4H)-one (5b)

Yield 64%, mp 103–105 °C, IR (KBr, cm^−1^): 3336 (NH), 3097 (C-H aromatic), 2904 (C-H aliphatic), 2206 (CN), 1674 (C=O), 1604 (C=N), 1581 (C=C). ^1^H-NMR (DMSO-*d_6_*, 400 MHz, δ ppm): 3.82 (s, 3H, CH_3_O), 3.84 (s, 1H, C_4_-H), 3.86 (s, 1H, C_1_-H), 6.91 (d, 2H, *J* = 8.30 Hz, 4-CH_3_O-C_6_H_4_-C_3,5_-H), 7.12 (d, 2H, *J* = 8.30 Hz, 4-CH_3_O-C_6_H_4_-C_2,6_-H), 7.35 (d, 1H, *J* = 8.50 Hz, C_5_-H), 7.66 (t, 1H, *J* = 8.50 Hz, C_6_-H), 7.80 (d, 1H, *J* = 8 Hz, C_7_-H), 7.99 (s, 1H, NH, D_2_O exchangeable). ^13^C-NMR (DMSO-*d_6_*, 100 MHz, δ ppm): 33.11 (C-_4_), 55.83 (C-_1_), 56.07 (OCH_3_), 115.59, 119.51, 122.12, 123.08, 126.33, 128.57, 128.76 (d, *J* = 38 Hz, C-_6_), 129.92, 132.44 (d, *J* = 259 Hz, C-F), 133.74, 135.47, 162.09, 163.95 (ArCs and CN), 177.52 (C=O). Anal. Calcd. (%) for C_19_H_13_FN_2_O_2_S (352): C, 64.76, H, 3.72, N, 7.95. Found: C, 64.98, H, 3.86, N, 8.19.

##### 2.1.5.3. 4-Cyano-8-fluoro-1-(2-nitrophenyl)-1,2-dihydro[1]benzothieno[2,3-c] pyridin-3(4H)-one (5c)

Yield 70%, mp 140–142 °C, IR (KBr, cm^−1^): 3194 (NH), 3067 (C-H aromatic), 2904(C-H aliphatic), 2199 (CN), 1720 (C=O), 1593 (C=N), 1565 (C=C), 1554, 1384 (NO_2_).^1^H-NMR (DMSO-*d_6_*, 400 MHz, δ ppm): 4.09 (s, 1H, C_4_-H), 4.31 (s, 1H, C_1_-H), 7.14 (d, 1H, *J* = 7.00 Hz, C_5_-H), 7.31–7.34 (m, 1H, C_6_-H), 7.44 (d, 1H, *J* = 7.00 Hz, C_7_-H), 7.62–7.66 (m, 2H, 2-NO_2_-C_6_H_4_-C_4,5_-H), 8.01–8.03 (m, 1H, 2-NO_2_-C_6_H_4_-C_6_-H), 8.44 (d, 1H, *J* = 8.00 Hz, 2-NO_2_-C_6_H_4_-C_3_-H), 9.50 (s, 1H, NH, D_2_O exchangeable). Anal. Calcd. (%) for C_18_H_10_FN_3_O_3_S (367): C, 58.85, H, 2.74, N, 11.44, S, 8.73. Found: C, 59.03, H, 2.61, N, 11.73, S, 8.94.

##### 2.1.5.4. 4-Cyano-8-fluoro-1-(3-nitrophenyl)-1,2-dihydro[1]benzothieno[2,3-c] pyridin-3(4H)-one (5d)

Yield 84%, mp 180–182 °C, IR (KBr, cm^−1^): 3417 (OH, taut), 3226 (NH), 3082 (C-H aromatic), 2974(C-H aliphatic), 2210 (CN), 1670 (C=O), 1620 (C=N), 1600 (C=C), 1527, 1350 (NO_2_). ^1^H-NMR (DMSO-*d_6_*, 400 MHz, δ ppm): 4.21 (s, 1H, C_4_-H), 4.75 (s, 1H, C_1_-H), 7.07 (d, 1H, *J* = 8.50 Hz, C_5_-H), 7.15–8.40 (m, 5H, C_6,7_-H and 3-NO_2_-C_6_H_4_-C_4,5,6_-H), 8.60 (s, 1H, 3-NO_2_-C_6_H_4_-C_2_-H), 9.10 (s, 1H, NH, D_2_O exchangeable). ^13^C-NMR (DMSO-*d_6_*, 100 MHz, δ ppm): 18.93 (C-_4_), 56.53 (C-_1_), 116.13, 117.98, 119.65, 120.62, 121.98, 123.13, 124.64, 128.53, 128.58 (d, *J* = 11 Hz, C-_6_), 130.98, 135.14, 136.79, 146.98 (d, *J* = 259 Hz, C-F), 156.03, 158.45 (ArCs and CN), 189.71 (C=O). MS: *m/z* (% relative abundance): 367 (M^+.^, 25.61%) and 326 (50.23%). Anal. Calcd. (%) for C_18_H_10_FN_3_O_3_S (367): C, 58.85, H, 2.74, H, 11.44. Found: C, 59.11, H, 2.96, N, 11.78.

#### 2.1.6. General procedure for synthesis of 4-substitutedamino-8-fluoro-1-(3-nitro phenyl)-1,2-dihydro[1]benzothieno[2,3-c]pyridin-3(4H)-ones (6a–c)

An equimolar mixture of **4d** (0.376 g, 0.001 mol), an appropriate secondary amine (0.001 mol), and triethylamine (two drops) in absolute ethanol (25 ml) was heated under reflux for 5 h. After cooling, the precipitated solid was filtered and crystallised from ethanol.

##### 2.1.6.1. 4-(4-Ethylpiperazin-1-yl)-8-fluoro-1-(3-nitrophenyl)-1,2-dihydro[1]benzo thieno[2,3-c]pyridin-3(4H)-one (6a)

Yield 53%, mp 240–241 °C, IR (KBr, cm^−1^): 3417 (NH), 3070 (C-H aromatic), 2916 (C-H aliphatic), 1680 (C=O), 1620 (C=N), 1600 (C=C), 1527, 1350 (NO_2_). ^1^H-NMR (DMSO-*d_6_*, 400 MHz, δ ppm): 0.99 (t, 3H, *J* = 7 Hz, CH_3_-CH_2_), 1.16–1.30 (m, 4H, piperazine-C_3,5_-H), 2.38 (q, 2H, *J* = 7 Hz, CH_3_-CH_2_), 2.90–3.09 (m, 4H, piperazine-C_2,6_-H), 3.89 (s, 1H, C_1_-H), 4.01 (s, 1H, C_4_-H), 7.10–8.40 (m, 6H, C_5,6,7_-H and 3-NO_2_-C_6_H_4_-C_4,5,6_-H), 8.43 (s, 1H, 3-NO_2_-C_6_H_4_-C_2_-H), 8.70 (s, 1H, NH, D_2_O exchangeable). ^13^C-NMR (DMSO-*d_6_*, 100 MHz, δ ppm): 12.02 (CH_3_), 15.60 (CH_2_), 29.12 (piperazine-C-3,5), 49.41 (piperazine-C-_2,6_), 51.49 (C-_1_), 52.53 (C-_4_), 97.60, 113.90, 120.98, 122.74, 123.95, 125.84, 127.94, 130.43, 131.06, 135.03 (d, *J* = 275 Hz, C-F), 136.41, 136.78, 144.13, 149.34 (ArCs), 187.21 (C=O). Anal. Calcd. (%) for C_23_H_23_FN_4_O_3_S (454): C, 60.78, H, 5.10, N, 12.33. Found: C, 60.61, H, 5.34, N, 12.49.

##### 2.1.6.2. 8-Fluoro-4-morpholino-1(3-nitrophenyl)-1,2-dihydro[1]benzothieno[2,3-c]pyridin-3(4H)-one (6b)

Yield 55%, mp 250–251 °C, IR (KBr, cm^−1^): 3417 (NH), 3074 (C-H aromatic), 2900 (C-H aliphatic), 1700 (C=O), 1590 (C=N), 1575 (C=C), 1531, 1350 (NO_2_). ^1^H-NMR (DMSO-*d_6_*, 400 MHz, δ ppm): 2.93–3.19 (m, 4H, morpholine-C_3,5_-H), 3.68–3.80 (m, 4H, morpholine-C_2,6_-H), 4.06 (s, 1H, C_1_-H), 4.83 (s, 1H, C_4_-H), 7.24–7.39 (m, 2H, C_5,6_-H), 7.44 (d, 1H, *J* = 7.16 Hz, C_7_-H), 7.77–8.34 (m, 1H, 3-NO_2_-C_6_H_4_-C_5_-H), 8.10 (d, 1H, *J* = 7.00 Hz, 3-NO_2_-C_6_H_4_-C_6_-H), 8.34 (d, 1H, *J* = 7.00 Hz, 3-NO_2_-C_6_H_4_-C_4_-H), 8.44 (s, 1H, 3-NO_2_-C_6_H_4_-C_2_-H), 8.70 (s, 1H, NH, D_2_O exchangeable). ^13^C-NMR (DMSO-*d_6_*, 100 MHz, δ ppm): 19.00 (morpholine C-_3,5_), 56.49 (morpholine C-_2,6_), 68.50 (C-_1_), 70.68 (C-_4_), 120.70, 122.86, 129.36, 130.69, 132.78 (d, *J* = 250 Hz, C-F), 134.03, 135.86, 139.36, 142.03, 145.36, 148.87, 156.20, 161.20, 169.20 (ArCs), 180.54 (C=O). MS: *m/z* (% relative abundance): 427 (M^+.^, 33.23%) and 62 (100%). Anal. Calcd. (%) for C_21_H_18_FN_3_O_4_S (427): C, 59.01, H, 4.24, N, 9.83. Found: C, 58.79, H, 4.47, N, 10.11.

##### 2.1.6.3. 8-Fluoro-1-(3-nitrophenyl)-4-(piperidin-1-yl)-1,2-dihydro[1]benzothieno [2,3-c]pyridin-3(4H)-one (6c)

Yield 61%, mp 230–232 °C, IR (KBr, cm^−1^): 3350 (NH), 3070 (C-H aromatic), 2943 (C-H aliphatic), 1670 (C=O), 1580 (C=N), 1556 (C=C), 1531, 1350 (NO_2_). ^1^H-NMR (DMSO-*d_6_*, 400 MHz, δ ppm): 1.50–1.57 (m, 2H, piperdine-C_4_-H), 1.58–1.70 (m, 4H, piperdine-C_3,5_-H), 2.90–3.07 (m, 4H, piperdine-C_2,6_-H), 3.93 (s, 1H, C_1_-H), 4.06 (s, 1H, C_4_-H), 7.20–7.32 (m, 1H, C_6_-H), 7.34 (d, 1H, *J* = 6.35 Hz, C_5_-H), 7.44 (d, 1H, *J* = 6.35 Hz, C_7_-H), 7.90 (t, 1H, *J* = 7.90 Hz, 3-NO_2_-C_6_H_4_-C_5_-H), 8.34 (d, 1H, *J* = 7.90 Hz, 3-NO_2_-C_6_H_4_-C_6_-H), 8.53 (d, 1H, *J* = 7.90 Hz, 3-NO_2_-C_6_H_4_-C_4_-H), 8.54 (s, 1H, 3-NO_2_-C_6_H_4_-C_2_-H), 8.69 (s, 1H, NH, D_2_O exchangeable). ^13^C-NMR (DMSO-*d_6_*, 100 MHz, ppm): 22.45 (piperdine-C-_4_), 22.92 (piperdine-C-_3,5_), 38.12 (piperdine-C-_2,6_), 44.11 (C-_1_) 56.51 (C-_4_), 122.22, 123.95, 124.64, 125.51, 127.59, 128.88, 130.94 (d, *J* = 11 Hz, C-_6_), 131.00, 134.33 (d, *J* = 212 Hz, C-F), 135.39, 136.95, 138.68, 148.39, 156.37 (ArCs), 191.73 (C=O). Anal. Calcd. (%) for C_22_H_20_FN_3_O_3_S (425): C, 62.10, H, 4.74, N, 9.88. Found: C, 62.37, H, 4.90, N, 10.09.

#### 2.1.7. General procedure for synthesis of 3,4-dichloro-8-fluoro-1-(3-nitrophenyl)-1,2-dihydro[1]benzothieno[2,3-c]pyridine (7a) and 3-chloro-4-cyano-8-fluoro-1-(3-nitrophenyl)-1,2-dihydro[1]benzothieno[2,3-c]pyridine (7b)

To a solution of phosphorus oxychloride (10 ml) and pyridine (2 ml), **4d** or **5d** (0.002 mol) was added, and the mixture was heated under reflux for 1 h. After cooling, the reaction mixture was poured cautiously to the crushed ice (40 g), and the solid product was filtered, washed with water, dried, and crystallised from acetonitrile.

##### 2.1.7.1. 3,4-Dichloro-8-fluoro-1-(3-nitrophenyl)-1,2-dihydro[1]benzothieno[2,3-c] pyridine (7a)

Yield 87%, mp 120–122 °C, IR (KBr, cm^−1^): 3360 (NH), 3078 (C-H aromatic), 2947 (C-H aliphatic), 1608 (C=N), 1580 (C=C), 1531, 1350 (NO_2_). ^1^H-NMR (DMSO-*d_6_*, 400 MHz, δ ppm): 4.06 (s, 1H, C_1_-H), 4.93 (s, 0.5 H, C_4_-H), 7.18–7.38 (m, 2H, C_5,6_-H), 7.46 (d, 1H, *J* = 7.80 Hz, C_7_-H), 7.70 (t, 1H, *J* = 8.00 Hz, 3-NO_2_-C_6_H_4_-C_5_-H), 8.05 (d, 1H, *J* = 8.00 Hz, 3-NO_2_-C_6_H_4_-C_6_-H), 8.21 (d, 1H, *J* = 8.00 Hz, 3-NO_2_-C_6_H_4_-C_4_-H), 8.34 (s, 1H, 3-NO_2_-C_6_H_4_-C_2_-H), 8.43 (s, 0.5H, NH, D_2_O exchangeable). ^13^C-NMR (DMSO-*d_6_*, 100 MHz, δ ppm): 46.00 (C-_1_), 72.72 (C-_4_), 123.65, 123.85, 128.81, 128.83 (d, *J* = 6 Hz, C-_6_), 129.12, 130.19, 130.55 (d, *J* = 286 Hz, C-F), 130.88, 130.99, 131.98, 135.94, 140.36, 148.37, 156.03, 158.87 (ArCs). MS: *m/z* (% relative abundance): 399 (M^+.^+4, 1.82%), 397 (M^+.^+2, 3.41%), 395 (M^+.^, 7.76%) and 352 (100%). Anal. Calcd (%) for C_17_H_9_Cl_2_FN_2_O_2_S (395): C, 51.66, H, 2.30, N, 7.09. Found: C, 51.92, H, 2.62, N, 7.36.

##### 2.1.7.2. 3-Chloro-4-cyano-8-fluoro-1-(3-nitrophenyl)-1,2-dihydro[1]benzothieno [2,3-c]pyridine (7b)

Yield 79%, mp 140–142 °C, IR (KBr, cm^−1^): 3367 (NH), 3078 (C-H aromatic), 2958 (C-H aliphatic), 2229 (CN), 1639 (C=N), 1593 (C=C), 1531, 1350 (NO_2_). ^1^H-NMR (DMSO-*d_6_*, 400 MHz, δ ppm): 4.06 (s, 1H, C_1_-H), 4.66 (s, 0.5H, C_4_-H, taut), 7.08–7.39 (m, 3H, C_5,6,7_-H), 7.53 (t, 1H, *J* = 8.00 Hz, 3-NO_2_-C_6_H_4_-C_5_-H), 7.68 (d, 1H, *J* = 8.00 Hz, 3-NO_2_-C_6_H_4_-C_4_-H), 7.86 (d, 1H, *J* = 8.00 Hz, 3-NO_2_-C_6_H_4_-C_6_-H), 7.97 (s, 1H, 3-NO_2_-C_6_H_4_-C_2_-H), 8.31 (s, 0.5H, NH, D_2_O exchangeable, taut). ^13^C-NMR (DMSO-*d_6_*, 100 MHz, δ ppm): 18.97 (C-_1_), 56.51 (C-_4_), 114.54, 124.07, 124.89, 128.57, 128.71 (d, *J* = 29 Hz, C-_6_), 130.62, 130.87, 130.98, 131.56, 133.62 (d, *J* = 275 Hz, C-F), 135.50, 136.59, 147.72, 151.29, 158.28, 164.17 (CN and ArCs). MS: *m/z* (% relative abundance): 387 (M^+.^+2, 14.61%), 385 (M^+.^, 38.03%) and 95 (100%). Anal. Calcd (%) for C_18_H_9_ClFN_3_O_2_S (385): C, 56.04, H, 2.35, N, 10.89. Found: C, 56.33, H, 2.59, N, 11.08.

#### 2.1.8. General procedure for synthesis of 3-substitutedamino-4-chloro-8-fluoro-1-(3-nitrophenyl)-1,2-dihydro[1]benzothieno[2,3-c]pyridines (8a–c) and 3-substitutedamino-4-cyano-8-fluoro-1-(3-nitrophenyl)-1,2-dihydro[1]benzo-thieno[2,3-c]pyridines (8d–f)

A mixture of **7a** or **7b** (0.001 mol), an appropriate secondary amine (0.001 mol), and triethylamine (two drops) in absolute ethanol (25 ml) was heated under reflux for 5 h. The solvent was concentrated under reduced pressure, and the precipitated solid was filtered, washed with water, dried, and crystallised from acetonitrile.

##### 2.1.8.1. 4-Chloro-3–(4-ethylpiperzin-1-yl)-8-fluoro-1-(3-nitrophenyl)-1,2-dihydro [1]benzothieno[2,3-c]pyridine (8a)

Yield 55%, mp 221–223 °C, IR (KBr, cm^−1^): 3390 (NH), 3074 (C-H aromatic), 2935 (C-H aliphatic), 1608 (C=N), 1580 (C=C), 1531, 1350 (NO_2_). ^1^H-NMR (DMSO-*d_6_*, 400 MHz, δ ppm): 0.99 (t, 3H, *J* = 7.16 Hz, CH_3_-CH_2_), 2.39 (q, 2H, *J* = 7.16 Hz, CH_3_-CH_2_), 2.54–2.60 (m, 4H, piperazine-C_3,5_-H), 3.02–3.09 (m, 4H, piperazine-C_2,6_-H), 4.06 (s, 1H, C_1_-H), 4.23 (s, 0.5H, C_4_-H, taut), 7.18–7.38 (m, 2H, C_5,6_-H), 7.42–7.52 (m, 1H, C_7_-H), 7.63–7.72 (m, 1H, 3-NO_2_-C_6_H_4_-C_5_-H), 7.86–7.90 (m, 1H, 3-NO_2_-C_6_H_4_-C_6_-H), 8.08 (d, 1H, *J* = 7.88 Hz, 3-NO_2_-C_6_H_4_-C_4_-H), 8.35 (s, 1H, 3-NO_2_-C_6_H_4_-C_2_-H), 9.02 (s, 0.5H, NH, D_2_O exchangeable, taut). ^13^C-NMR (DMSO-*d_6_*, 100 MHz, δ ppm): 12.31 (CH_3_), 22.02 (CH_2_), 27.74 (piperazine C-_3,5_), 49.58 (piperazine C-_2,6_), 51.77 (C-_1_), 76.80 (C-_4_), 118.23, 123.78, 124.47, 125.16, 126.23, 127.76, 128.88, 130.19, 130.90, 131.91, 135.04, 138.16 (d, *J* = 313 Hz, C-F), 139.73, 148.39, 161.22 (ArCs). Anal. Calcd (%) for C_23_H_22_ClFN_4_O_2_S (472): C, 58.41, H, 6.69, N, 11.85. Found: C, 58.59, H, 6.75, N, 11.99.

##### 2.1.8.2. 4-Chloro-3-(4-morpholino)-8-fluoro-1-(3-nitrophenyl)-1,2-dihydro [1]benzothieno[2,3-c]pyridine (8b)

Yield 74%, mp 175–177 °C, IR (KBr, cm^−1^): 3387 (NH), 3074 (C-H aromatic), 2927 (C-H aliphatic), 1608 (C=N), 1580 (C=C), 1531, 1350 (NO_2_). ^1^H-NMR (DMSO-*d_6_*, 400 MHz, δ ppm): 3.02–3.10 (m, 4H, morpholine-C_3,5_-H), 3.70–3.78 (m, 4H, morpholine-C_2,6_-H), 4.07 (s, 1H, C_1_-H), 4.20 (s, 0.5H, C_4_-H, taut), 7.19–7.40 (m, 2H, C_5,6_-H), 7.43–7.53 (m, 1H, C_7_-H), 7.64–7.73 (m, 2H, 3-NO_2_-C_6_H_4_-C_5,6_-H), 8.23 (d, 1H, *J* = 7.00 Hz, 3-NO_2_-C_6_H_4_-C_4_-H), 8.42 (s, 1H, 3-NO_2_-C_6_H_4_-C_2_-H), 8.80 (s, 0.5H, NH, D_2_O exchangeable, taut). ^13^C-NMR (DMSO-*d_6_*, 100 MHz, δ ppm): 40.62 (morpholine C-_3,5_), 55.99 (morpholine C-_2,6_), 64.14 (C-_1_), 66.65 (C-_4_), 124.30, 125.68, 126.72, 128.11, 128.90, 129.67, 130.30, 130.88, 132.01, 132.62, 133.66, 133.85 (d, *J* = 238 Hz, C-F), 138.68, 139.90, 148.39 (ArCs). Anal. Calcd (%) for C_21_H_17_ClFN_3_O_3_S (445): C, 56.57, H, 3.84, N, 9.42. Found: C, 56.85, H, 3.99, N, 9.50.

##### 2.1.8.3. 4-Chloro-3-(piperidin-1-yl)-8-fluoro-1-(3-nitrophenyl)-1,2-dihydro [1]benzothieno[2,3-c]pyridine (8c)

Yield 68%, mp 241–243 °C, IR (KBr, cm^−1^): 3410 (NH), 3070 (C-H aromatic), 2935 (C-H aliphatic), 1608 (C=N), 1580 (C=C), 1527, 1350 (NO_2_). ^1^H-NMR (DMSO-*d_6_*, 400 MHz, δ ppm): 0.86–0.89 (m, 2H, piperidine-C_4_-H), 1.11–1.22 (m, 4H, piperidine-C_3,5_-H), 1.54–1.63 (m, 4H, piperidine-C_2,6_-H), 2.97 (s, 1H, C_1_-H), 4.14 (s, 0.5H, C_4_-H, taut), 7.08–7.71 (m, 5H, C_5,6,7_-H and 3-NO_2_-C_6_H_4_-C_5,6_-H), 8.12 (d, 1H, *J* = 8.24 Hz, 3-NO_2_-C_6_H_4_-C_4_-H), 8.41 (s, 1H, 3-NO_2_-C_6_H_4_-C_2_-H), 9.01 (s, 0.5H, NH, D_2_O exchangeable, taut). ^13^C-NMR (DMSO-*d_6_*, 100 MHz, δ ppm): 14.33 (piperidine C-_4_), 22.62 (piperidine C-_3,5_), 25.92 (piperidine C-_2,6_), 44.11 (C-_1_), 54.20 (C-_4_), 127.92, 128.84 (d, *J* = 6 Hz, C-_6_), 129.11, 129.89, 130.88, 130.99, 132.05, 132.17, 132.51 (d, *J* = 300 Hz, C-F), 147.70, 148.70, 155.53, 155.34, 167.54, 169.05 (ArCs). MS: *m/z* (% relative abundance): 443 (M^+.^+2, 3.73%), 445 (M^+.^, 8.18%) and 69 (100%). Anal. Calcd (%) for C_22_H_19_ClFN_3_O_2_S (443): C, 59.52, H, 4.31, N, 9.47. Found: C, 59.68, H, 4.40, N, 9.55.

##### 2.1.8.4. 4-Cyano-3-(4-ethylpiperazin-1-yl)-8-fluoro-1-(3-nitrophenyl)-1,2-dihydro [1]benzothieno[2,3-c]pyridine (8d)

Yield 63%, mp 224–226 °C, IR (KBr, cm^−1^): 3390 (NH), 3062 (C-H aromatic), 2823 (C-H aliphatic), 2210 (CN), 1593 (C=N), 1545 (C=C), 1531, 1350 (NO_2_). ^1^H-NMR (DMSO-*d_6_*, 400 MHz, δ ppm): 1.00 (t, 3H, *J* = 7 Hz, CH_3_-CH_2_), 2.40 (q, 2H, *J* = 7 Hz, CH_3_-CH_2_), 2.70–2.95 (m, 4H, piperazine-C_3,5_-H), 3.01–3.21 (m, 4H, piperazine-C_2,6_-H), 3.94 (s, 0.5H, C_4_-H, taut), 4.07 (s, 1H, C_1_-H), 6.71 (t, 1H, *J* = 7.00 Hz, C_6_-H), 7.09 (d, 1H, *J* = 7.00 Hz, C_5_-H), 7.20–7.40 (m, 1H, 3-NO_2_-C_6_H_4_-C_5_-H), 7.50 (d, 1H, *J* = 8.00 Hz, 3-NO_2_-C_6_H_4_-C_6_-H), 7.69 (d, 1H, *J* = 8.00 Hz, 3-NO_2_-C_6_H_4_-C_4_-H), 7.85 (d, 1H, *J* = 7.00 Hz, C_7_-H), 8.20 (s, 1H, 3-NO_2_-C_6_H_4_-C_2_-H), 8.65 (s, 0.5H, NH, D_2_O exchangeable, taut). ^13^C-NMR (DMSO-*d_6_*, 100 MHz, δ ppm): 11.94 (CH_3_), 31.55 (CH_2_), 43.21 (piperazine-C-_3,5_), 49.34 (piperazine-C-_2,6_), 51.75 (C-_4_), 60.15 (C-_1_), 114.42, 120.31, 123.03, 125.86, 127.94, 128.80, 129.67, 131.06, 132.10, 132.27 (d, *J* = 242 Hz, C-F), 137.99, 139.21, 144.41, 149.26, 152.55, 158.97 (CN and ArCs). Anal. Calcd (%) for C_24_H_22_FN_5_O_2_S (463): C, 62.19, H, 4.78, N, 15.11. Found: C, 62.44, H, 4.89, N, 15.34.

##### 2.1.8.5. 4-Cyano-3-(4-morpholino)-8-fluoro-1-(3-nitrophenyl)-1,2-dihydro [1]benzothieno[2,3-c]pyridine (8e)

Yield 43%, mp 242–244 °C, IR (KBr, cm^−1^): 3356 (NH), 2843 (C-H aromatic), 2843 (C-H aliphatic), 2210 (CN), 1610 (C=N), 1593 (C=C), 1535, 1350 (NO_2_). ^1^H-NMR (DMSO-*d_6_*, 400 MHz, δ ppm): 2.91–3.15 (m, 4H, morpholine-C_3,5_-H), 3.61–3.79 (m, 4H, morpholine-C_2,6_-H), 4.08 (s, 0.5H, C_4_-H, taut), 4.22 (s, 1H, C_1_-H), 6.65–6.90 (m, 1H, C_6_-H), 7.09 (d, 1H, *J* = 6.50 Hz, C_5_-H), 7.43 (d, 1H, *J* = 6.50 Hz, C_7_-H), 7.18–7.32 (m, 1H, 3-NO_2_-C_6_H_4_-C_5_-H), 7.68 (d, 1H, *J* = 8.00 Hz, 3-NO_2_-C_6_H_4_-C_6_-H), 7.84 (d, 1H, *J* = 8.00 Hz, 3-NO_2_-C_6_H_4_-C_4_-H), 8.19 (s, 1H, 3-NO_2_-C_6_H_4_-C_2_-H), 8.80 (s, 0.5H, NH, D_2_O exchangeable, taut). ^13^C-NMR (DMSO-*d_6_*, 100 MHz, δ ppm): 43.23 (morpholine-C-_3,5_), 48.43 (morpholine-C-_2,6_), 63.74 (C-_4_), 66.40 (C-_1_), 114.61, 116.93, 124.17, 124.37, 125.44, 128.24, 128.84 (d, *J* = 6 Hz, C-_6_), 130.36, 130.88, 131.56, 137.04 (d, *J* = 234 Hz, C-F), 138.13, 138.24, 147.56, 159.28, 160.35 (CN and ArCs). MS: *m/z* (% relative abundance): 436 (M^+.^, 21.55%) and 328 (100.00%). Anal. Calcd (%) for C_22_H_17_FN_4_O_3_S (436): C, 60.64, H, 3.93, N, 12.84. Found: C, 60.38, H, 4.18, N, 13.12.

##### 2.1.8.6. 4-Cyano-3-(piperidin-1-yl)-8-fluoro-1-(3-nitrophenyl)-1,2-dihydro [1]benzothieno[2,3-c]pyridine (8f)

Yield 50%, mp 280–282 °C, IR (KBr, cm^−1^): 3390 (NH), 3012 (C-H aromatic), 2947 (C-H aliphatic), 2210 (CN), 1593 (C=N), 1566 (C=C), 1531, 1350 (NO_2_). ^1^H-NMR (DMSO-*d_6_*, 400 MHz, δ ppm): 0.89–1.10 (m, 2H, piperidine-C_4_-H), 1.20–1.60 (m, 4H, piperidine-C_3,5_-H), 2.50–2.90 (m, 4H, pipeirdine-C_2,6_-H), 3.81 (s, 0.5H, C_4_-H, taut), 3.92 (s, 1H, C_1_-H), 6.50–7.60 (m, 5H, C_5,6,7_-H and 3-NO_2_-C_6_H_4_-C_5,6_-H), 7.90–8.10 (m, 2H, 3-NO_2_-C_6_H_4_-C_2,4_-H), 8.35 (s, 0.5H, NH, D_2_O exchangeable, taut). ^13^C-NMR (DMSO-*d_6_*, 100 MHz, δ ppm): 22.54 (piperidine C-_4_), 26.01 (piperidine C-_3,5_), 44.03 (piperidine C-_2,6_), 48.44 (C-_4_), 55.30 (C-_1_), 122.74, 124.47, 125.34, 126.35, 128.25, 128.97, 130.36, 132.65 (d, *J* = 235 Hz, C-F), 133.62, 133.83, 135.91, 139.55, 141.04, 145.94, 147.53, 153.59 (CN and ArCs). Anal. Calcd (%) for C_23_H_19_FN_4_O_2_S (434): C, 63.58, H, 4.41, N, 12.89. Found: C, 63.71, H, 4.60, N, 13.10.

##### 2.1.9. 9-Fluoro-7-(3-nitrophenyl)-5,6,7,13-tetrahydro[1]benzothieno[3′,2′:4,5]-pyrido[2,3-b]quinoxaline (9)

An equimolar mixture of **7a** (0.39 g, 0.001 mol), *o*-phenylenediamine (0.1 g, 0.001 mol), and triethylamine (2 drops) in dry benzene (25 ml) was heated under reflux for 5 h. After cooling, the separated solid was filtered, washed with ethanol, dried, and crystallised from acetonitrile.

Yield 83%, mp 160–162 °C, IR (KBr, cm^−1^): 3375–3321 (3NH), 3078 (C-H aromatic), 2924 (C-H aliphatic), 1635 (C=N), 1589 (C=C), 1527, 1350 (NO_2_). ^1^H-NMR (DMSO-*d_6_*, 400 MHz, δ ppm): 4.06 (s, 1H, C_7_-H), 6.53–6.55 (m, 2H, C_2,3_-H) 6.65–6.68 (m, 2H, C_1,4_-H), 7.20–7.30 (m, 1H, C_11_-H), 7.35 (d, 1H, *J* = 8.16 Hz, C_12_-H), 7.60–7.91 (m, 2H, C_10_-H and 3-NO_2_-C_6_H_4_-C_5_-H), 8.16 (s, 2H, 2NH of quinoxaline, D_2_O exchangeable), 8.20–8.28 (m, 1H, 3-NO_2_-C_6_H_4_-C_6_-H), 8.33 (d, 1H, *J* = 8.44 Hz, 3-NO_2_-C_6_H_4_-C_4_-H), 8.39 (s, 1H, 3-NO_2_-C_6_H_4_-C_2_-H), 8.64 (s, 1H, NH, D_2_O exchangeable). ^13^C-NMR (DMSO-*d_6_*, 100 MHz, δ ppm: 57.21(C-_7_), 117.42, 117.75, 117.85 (d, *J* = 20 Hz, C-_11_), 119.25, 120.33, 124.68, 126.16, 126.50, 127.74, 128.98, 131.06, 132.64 (d, *J* = 317 Hz, C-F), 134.23, 136.50, 139.40, 142.10, 145.80, 149.77, 150.11 (ArCs). MS: *m/z* (% relative abundance): 430 (M^+.^, 7.00%) and 65 (100%). Anal. Calcd (%) for C_23_H_15_FN_4_O_2_S (430): Calcd, C, 63.88; H, 3.96; N, 12.96. Found: C, 64.12; H, 4.10; N, 13.24.

##### 2.1.10. 4-Cyano-8-fluoro-3((2-fluorophenyl) amino)-1–(3-nitrophenyl)-1,2-dihydro[1]benzothieno[2,3-c]pyridine (10)

Triethylamine (0.3 g, 0.003 mol) was added to a mixture of **7b** (0.38 g, 0.001 mol) and 2-fluoroaniline (0.22 g, 0.002 mol) in absolute ethanol (25 ml), and the reaction mixture was heated under reflux for 8 h. The solvent was concentrated under reduced pressure, and the resulting solid product was filtered, dried, and crystallised from benzene.

Yield 81%, mp 240–242 °C, IR (KBr, cm^−1^): 3348, 3410 (2NH), 3050 (C-H aromatic), 2939 (C-H aliphatic), 2229 (CN), 1610 (C=N), 1593 (C=C), 1531, 1350 (NO_2_). ^1^H-NMR (DMSO-*d_6_*, 400 MHz, δ ppm): 4.06 (s, 0.5H, C_4_-H, taut), 4.20 (s, 1H, C_1_-H), 4.40 (s, 1H, NH, D_2_O exchangeable), 6.75–6.85 (m, 1H, C_6_-H), 6.89 (t, 1H, *J* = 7.50 Hz, 2-F-C_6_H_4_NH_2_-C_4_-H), 7.05–7.18 (m, 1H, C_5_-H), 7.22 (t, 1H, *J* = 7.50 Hz, 2-F-C_6_H_3_NH_2_-C_5_-H), 7.32–7.39 (m, 1H, 2-F-C_6_H_4_NH_2_-C_6_-H), 7.44 (d, 1H, *J* = 7.50 Hz, 2-F-C_6_H_3_NH_2_-C_3_-H), 7.68 (d, 1H, *J* = 8.40 Hz, C_7_-H), 7.71–7.78 (m, 1H, 3-NO_2_-C_6_H_4_-C_5_-H), 7.87 (d, 1H, *J* = 8.40 Hz, 3-NO_2_-C_6_H_4_-C_6_-H), 7.93–8.00 (m, 1H, 3-NO_2_-C_6_H_4_-C_4_-H), 8.31 (s, 1H, 3-NO_2_-C_6_H_4_-C_2_-H), 8.46 (s, 0.5H, NH, D_2_O exchangeable). ^13^C-NMR (DMSO-*d_6_*, 100 MHz, δ ppm): 45.82 (C-_4_), 52.38 (C-_1_), 116.05, 124.09, 124.92, 125.53, 127.93, 128.71 (d, *J* = 24 Hz, C-_6_), 128.83, 128.88, 129.71, 130.01, 130.64, 130.90, 131.00, 131.57, 133.77 (d, *J* = 305 Hz, C-F), 135.30, 135.52, 136.61, 136.76, 140.59, 147.73, 151.32 (CN and ArCs). Anal. Calcd (%) for C_24_H_14_F_2_N_4_O_2_S (460): C, 62.60, H, 3.06, N, 12.17. Found: C, 62.47, H, 3.28, N, 12.06.

### 2.2. Anticancer activity

#### 2.2.1. Measurement of cytotoxic activity

The cytotoxic activity of the twenty-seven newly synthesised compounds was evaluated using the NCI disease-oriented human cell lines screening assay. The cytotoxic assay was performed according to the protocol of the Drug evaluation branch, NCI, Bethesda, MD, USA. The new compounds were evaluated against a panel of 60 cancer cell lines derived from leukaemia, melanoma, lung, colon, CNS, ovarian, renal, prostate, and breast cancers at a single concentration of 10^−5 ^M. The cultures were incubated for 48 h before performing the sulforhodamine (SRB) protein assay as described previously^30^^,^^31^. The compound with significant cytotoxic activity was further evaluated at five different concentrations ranging from 10^−4^ to 10^−8 ^M. Cell viability and growth were estimated, and the results were reported as a percentage growth of the treated cells *vs.* untreated control cells. The molar concentration that produced a 50% decrease in cell growth (GI_50_) was calculated.

#### 2.2.2. MTT cytotoxicity assay protocol

The MTT method for monitoring *in vitro* cytotoxicity was used with multiwell plates. Three human cell lines, namely prostate cancer cell line (PC-3), renal cancer cell line (UO-31), and breast cancer cell line (MCF-7) were obtained from the American Type Culture Collection ATCC, Manassas, VA, USA). The cells were trypsinized and seeded in 96 well plates with a density of 1.2 − 1.8 × 10,000 cells/well for 24 h at 37 °C. After treating the cells with five concentrations of compound **5c** (0.01, 0.1, 1, 10, 100 µM), an MTT solution (Sigma Co., St. Louis, MO, USA) (5 mg/mL in phosphate buffer solution) was applied to each well and left for 48 h. The plates were cultivated in 5% CO_2_ for 4 h at 37 °C. Successively, the remaining formazan crystals were dissolved in 100 µl of DMSO, and the absorbance was determined with the ROBONIK P2000 spectrophotometer at a wavelength of 570 nm. The obtained values were analysed using Gen5 software (BioTek, UK). The proliferation percentage in each treated cell line was standardised based on their control wells. Both tests have been conducted in triplicate. The IC_50_ values were calculated using sigmoidal dose-response curve fitting models. For best results, cells in the log phase of growth were employed, and the final cell number did not exceed 106 cells/cm^2^. Each test included a blank containing a complete medium without cells.

#### 2.2.3. In vitro cytotoxicity on WI-38 human cell line protocol

Cell Line cells were obtained from American Type Culture Collection and were cultured using DMEM (Invitrogen/Life Technologies) supplemented with 10% FBS (Hyclone), 10 µg/ml of insulin (Sigma), and 1% penicillin-streptomycin. All of the other chemicals and reagents were obtained from Sigma or Invitrogen. Plate cells were incubated (cells density 1.2–1.8 × 10,000 cells/well) in a volume of 100 µL complete growth medium and 100 ul of the tested compound per well in a 96-well plate for 24 h before the MTT assay. The cultures were removed from the incubator into a laminar flow hood or other sterile work area followed by the addition of reconstituted MTT in an amount equal to 10% of the culture medium volume, then incubated for an additional 2 h. After the incubation period, cultures were removed from the incubator, and the resulted formazan crystals were dissolved by adding an amount of MTT solubilisation solution [M-8910] equal to the original culture medium volume, then the absorbance at a wavelength of 570 nm was measured. Finally, the IC_50_ of the tested compound compared to the reference was calculated using GraphPad Prism software.

#### 2.2.4. Cyp17 Enzyme inhibition in mice prostate cancer model

##### 2.2.4.1. Animals and materials

Animals’ treatment protocol was approved by the Faculty of Pharmacy, Cairo University Animal Rights Committee (OC 2740). In all tests, adequate considerations were adopted to reduce the pain or discomfort of animals.

A total of 16 mice from the TRAMP/FVB background (Sprague-Dawley, male, average 45 days, average weight 120 gm, mice with prostate cancer) were taken and divided into 2 groups with 8 animals in each group. One group of mice was given a drug in 0.2 ml of DMSO and phosphate-buffered saline (1:10) ratio (5 mg/kg body weight, *i.p*., 5 days a week). The control group animals were treated with 0.2 ml of DMSO and phosphate-buffered saline (1:10) ratio. A total of eight mice, four animals from each group, were examined at 13 weeks of age, and the remaining animals from each group were examined at 20 weeks for prostate tumour development. Treatment was stopped 24 h before killing the animals.

##### 2.2.4.2. ELISA assay for CYP17 enzyme

BioSource 2704060 96 assay kit is a sandwich enzyme immunoassay for *in vitro* quantitative measurement of CYP17 enzyme activity in mouse tissue homogenates, cell lysates, cell culture supernatant, and other biological fluids^32^. Tested tissues were rinsed in ice-cold PBS to remove excess blood and weighed before homogenisation. The tissues were minced and homogenised in fresh lysis buffer with a glass homogeniser. (catalog: IS007, different lysis buffer needs to be chosen based on subcellular location of the target protein) (w:v = 1:20–1:50, e.g. 1 ml lysis buffer is added in 20–50 mg tissue sample) The resulting suspension was sonicated with an ultrasonic cell disrupter till the solution was clarified. The homogenates were centrifuged for 5 min at 10,000 × g, and the supernatant was collected. An Aliquot was assayed and stored at ≤ −20 °C.

The assay sample and standard were incubated together with CYP-HRP conjugate in an appropriate number of microplates pre-coated with an antibody specific for the CYP17 enzyme for 1 h. After the incubation period, the wells were decanted and washed five times. The wells were then incubated with a substrate for the HRP enzyme. A blue-colored complex was produced as a result of an enzyme-substrate reaction. Finally, a stop solution (2*N* hydrochloric acid solution) was added to stop the reaction, turning the solution yellow. Colour intensity was measured spectrophotometrically at 450 nm in a microplate reader. A standard curve was plotted between the intensity of the colour *vs.* the concentration of standards. The CYP17 concentration in each sample was interpolated from this standard curve.

**Assay procedures**: Wells for a standard, blank, and sample were prepared. Dilutions of standard, blank, and sample (100 µL each) were added into the appropriate wells, covered with the plate sealer, then incubated for 1 h at 37 °C. The liquid was removed from each well without washing. Detection reagent **A (**100 µL) was added to each well, covered with the plate sealer, and incubated for 1 h at 37 °C. The solution was aspirated and washed three times with 350 µL of wash solution to each well. After the last wash, any remaining wash buffer was removed by aspirating or decanting. Detection reagent **B (**100 µL) was added to each well, covered with the plate sealer, and incubated for 30 min at 37 °C. The aspiration/washing process has been repeated a total of 5 times as before. Substrate solution (90 µL) was added to each well, covered with a plate sealer, and incubated for 10–20 min at 37 °C in the dark. The liquid turns blue by the addition of substrate solution. Stop solution (2 N hydrochloric acid solution, 50 µL) was added to each well till the liquid turns yellow. The absorbance was measured immediately at 450 nm.

#### 2.2.5. Testosterone assay

The BioVendor mouse/rat testosterone ELISA assay is a competitive immunoassay for the measurement of testosterone in rat and mouse serum or plasma based on the principle of competitive binding^33^. An unknown amount of testosterone present in the sample (plasma or serum) and a known amount of testosterone conjugated to horseradish peroxidase compete for the binding sites of testosterone antiserum coated to the wells of a microplate. The sample was incubated with the microplate for 1 h, then washed four times. The HRP substrate solution was added followed by the stop solution. Colour change has been observed, and the optical density was measured at 450 nm.

**Assay procedures:** Sufficient number of microplate wells were prepared to accommodate standard and samples in duplicates. The standard, sample, and control (10 µL each) were dispensed with new disposable tips into appropriate wells. Incubation buffer 100 µL was dispensed into each well. Afterward, enzyme conjugate (50 µL) was added to each well and incubated for 60 min at room temperature on a microplate mixer. The content of the wells was discarded, and the wells were rinsed 4 times with diluted wash solution (300 µL per well). The substrate solution (200 µL) was added to each well and incubated in the dark without shaking for 30 min. The reaction was stopped by adding a stop solution (2 N hydrochloric acid solution, 50 µL) to each well. The absorbance of each well was determined at 450 nm.

#### 2.2.6. Cell cycle analysis

The prostate PC-3 cells were placed in a six-well plate at 1 × 10^5^ conc. of cells/well, then incubated for 24 h. The cells were treated with 2.34 µM of compound **5c** or (0.1% DMSO) for 24 h. Thereafter, cells were collected and fixed for 12 h using ice-cold 70% ethanol at 4 °C. Then, ethanol was removed, and the cells were washed with cold Phosphate Buffer Saline (PBS) and incubated for 30 min at 37 °C in 0.5 ml of PBS. The cells were stained for 30 min with propidium iodide in the dark. The flow cytometer was used to detect DNA contents^34^.

#### 2.2.7. Annexin V-FITC assay

PC-3 cells were placed in a 6-well plate at 1 × 10^5^ conc. of cells per well, then incubated for 24 h. Afterward, the cells were treated with 2.34 µM of compound **5c** or (0.1% DMSO) for 24 h, then harvested, washed with PBS, and stained with annexin V-FITC and PI in binding buffer (10 µM HEPES, 140 µM NaCl, and 2.5 µM CaCl_2_ at pH 7.4) for 15 min at room temperature in the dark, then analysed by the flow cytometer^35^.

## 3. Results and discussion

### 3.1. Chemistry

**Scheme 1.**
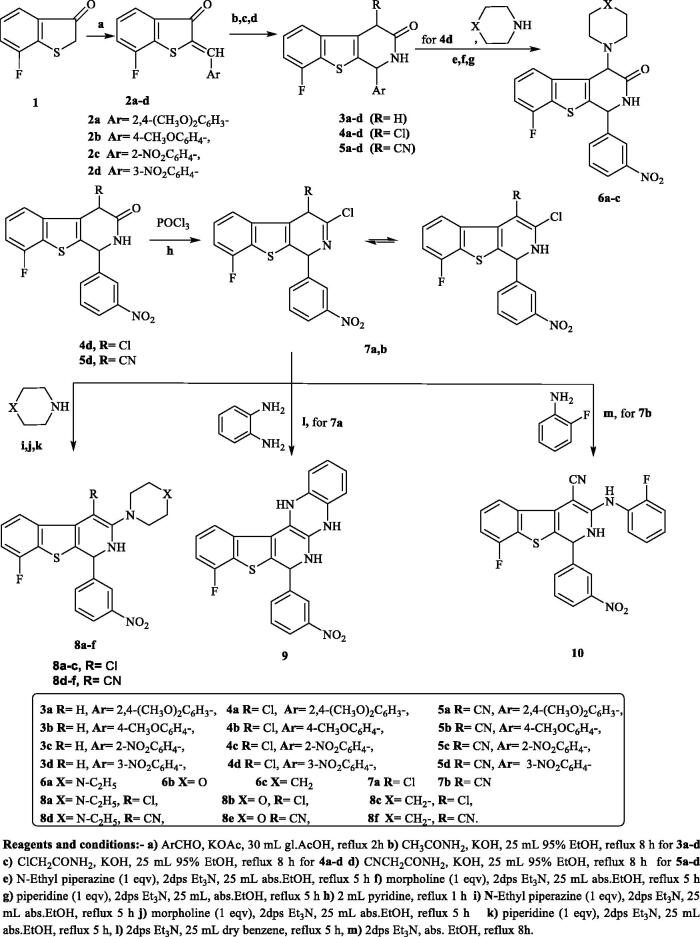
The synthetic pathways and reagents for the preparation of the target compounds **2a–d**, **3a–d**, **4a–d**, **5a–d**, **6a–c**, **7a,b**, **8a–f**, **9**, and **10**. Reagents and conditions: (a) ArCHO, KOAc, 30 mL gl.AcOH, reflux 2h, (b) CH3CONH2, KOH, 25 mL 95% EtOH, reflux 8 h for **3a–d**, (c) ClCH2CONH2, KOH, 25 mL 95% EtOH, reflux 8 h for **4a–d**, (d) CNCH_2_CONH_2_, KOH, 25 mL 95% EtOH, reflux 8 h for **5a–d**, (e) *N*-Ethyl piperazine (1 eqv), 2dps Et_3_N, 25 mL abs.EtOH, reflux 5 h, (f) morpholine (1 eqv), 2dps Et_3_N, 25 mL abs.EtOH, reflux 5 h, (g) piperidine (1 eqv), 2dps Et_3_N, 25 mL, abs.EtOH, reflux 5 h, (h) 2 mL pyridine, reflux 1 h, (i) *N***-**Ethyl piperazine (1 eqv), 2dps Et_3_N, 25 mL abs.EtOH, reflux 5 h, (j) morpholine (1 eqv), 2dps Et3N, 25 mL abs.EtOH, reflux 5 h, (k) piperidine (1 eqv), 2dps Et_3_N, 25 mL abs.EtOH, reflux 5 h, (l) 2dps Et_3_N, 25 mL dry benzene, reflux 5 h, and (m) 2dps Et_3_N, abs. EtOH, reflux 8 h.

The synthetic pathways of the target compounds are illustrated in Scheme 1. Herein, the arylidene derivatives **2a–d** are the key intermediates for the synthesis of the designed [1]benzothieno[2,3-*c*]pyridine derivatives **3a–d**, **4a–d**, and **5a–d**. Compounds **2a–d** were prepared by condensation of 7-fluoro[1]benzothiophen-3(2*H*)-one (**1)** with some aromatic aldehydes in glacial acetic acid and anhydrous potassium acetate as a catalyst, following previously reported procedure^36^. After that, cyclisation of the produced α,β-unsaturated ketones **2a–d** with acetamide, 2-chloroacetamide, or 2-cyanoacetamide in the presence of potassium hydroxide as a catalyst afforded the target compounds **3a–d**, **4a–d**, and **5a–d**, respectively, according to a reported procedure^37^. Previous studies in this field showed that compounds containing 3-nitrophenyl moiety at the 4-position of 1,2-dihydro[1]bezothienopyridine ring expressed promising anticancer activity^29^. On this basis, compounds **4d** and **5d** were chosen in the preparation of other new bezothienopyridine derivatives hoping to obtain compounds potentially active as anticancer agents. The reaction of **4d** with several secondary amines in the presence of triethylamine afforded **6a–c**. On the other hand, the preparation of **7a,b** has been accomplished *via* chlorination of **4d** or **5d** with excess phosphorus oxychloride in pyridine, adopting the reported procedure^38^. Furthermore, amination of **7a,b** with a number of secondary amines afforded 3-substitutedamino-4-chloro[1]benzothieno[2,3-*c*]pyridines **8a–c** and 3-substituted amino-4-cyano-[1]benzothieno[2,3-*c*]pyridine derivatives **8d–f**. Moreover, the reaction of **7a** with *o*-phenylenediamine in the presence of triethylamine produced the quinoxaline derivative **9.** Finally, heating of **7b** with *o*-fluoroaniline in absolute ethanol and triethylamine as a catalyst produced 4-cyano-8-fluoro-3[(2-fluorophenyl) amino][1]benzothieno[2,3-*c*]pyridine **10** (Scheme 1).

^1^H-NMR spectra of compounds **2a,c,d** revealed the appearance of benzylidene proton (=CH) in the range of 8.26–8.70 ppm.

IR spectra of the target derivatives **3a–d**, **4a–d**, and **5a–d**, showed tautomeric OH, NH and C=O bands. Compounds **5a–d** displayed sharp bands at 2206–2210 cm^−1^, confirming the presence of CN function. ^1^H-NMR spectra of **3a–d**, **4a–d**, and **5a–d**, demonstrated the disappearance of benzylidene proton signal. On the other hand, they revealed a singlet signal in a range of 3.78–4.75 ppm corresponding to C_1_-H proton and a D_2_O exchangeable singlet signal attributable to NH proton. In addition, ^1^H-NMR spectra of compounds **3a–d** showed a singlet signal at about 3.00–3.93 ppm corresponding to CH_2_ protons, while the spectra of compounds **4a–d** and **5a–d** revealed a singlet signal at about 3.84–4.21 ppm corresponding to C_4_-H. Finally, tautomeric OH singlet signal integrated for half proton was observed in compounds **3d**, **4a,** and **5a**, indicating the presence of two tautomers. ^13^C-NMR spectra of the target compounds revealed two signals in the aliphatic region corresponding to carbons at the position 1 and 4. The characteristic C=O signal was displayed in the range of 177.52–192.27 ppm. Mass spectra of compounds **3d, 4d,** and **5d** were consistent with their structures. Compound **4d** showed M^+^ and M^+^ +2 peaks confirming the presence of the Cl atom.

Further, IR spectra of **6a–c** showed NH str. and C=O str. bands. The ^1^H-NMR spectrum of **6a** displayed triplet and quartette signals confirming the ethyl group of *N*-ethylpiperazine. Moreover, the spectrum showed two multiplets corresponding to the piperazine ring protons. However, the ^1^H-NMR spectrum of **6b** revealed two multiplet signals of the morpholine ring protons. On the other hand, compound **6c** showed three multiplet signals at about 1.50–1.57, 1.58–1.70, and 2.90–3.07 ppm corresponding to piperidine-C_4_-H, piperidine-C_3,5_-H, and piperidine-C_2,6_-H protons, respectively. Further structural evidence steamed from the ^13^C-NMR spectra of **6a**, **6b**, and **6c**, which were consistent with the exact structure of these compounds. The ^13^C-NMR spectrum of **6a** illustrated six signals of aliphatic carbons corresponding to ethyl group carbons, ethylpiperazine-C-_3,5_, ethylpiperazine-C-_2,6_, C-_1_, and C-_4_. Also, the ^13^C-NMR spectrum of **6b** showed four signals in the aliphatic region, attributable to morpholine-C-_3,5_, morpholine-C-_2,6_, C-_1_, and C-_4_. However, the ^13^C-NMR spectrum of **6c** demonstrated five signals in the aliphatic area for piperidine-C-_4_, piperidine-C-_3,5_, C-_1_, piperidine-C-_2,6_, and C-_4_. All compounds displayed a carbonyl carbon. Moreover, the mass spectrum of compound **6b** demonstrated its molecular ion peak with the absence of M^+.^+2 peak of its chloro precursor **4d**.

IR spectrum of the chloro derivatives **7a** and **7b** lacked the C=O str. band of the precursors **4d** and **5d**. On the other hand, they displayed NH absorption bands. Their ^1^H-NMR spectra demonstrated a singlet signal at 4.06 corresponding to C_1_-H. In addition, compounds **7a** and **7b** exhibited two singlets, each integrated for half proton, attributed to C_4_-H tautomer, as well as a D_2_O exchangeable signal of NH proton. Their ^13^C-NMR spectra showed two signals corresponding to the aliphatic carbons at C-_1_ and C-_4_. In addition, the aromatic carbons were in full correspondence to the exact structures. Mass spectra of compound **7a** showed M^+.^, M^+.^+2, and M^+.^+4 isotopes, confirming the presence of two Cl atoms. On the other hand, the presence of one chlorine atom in **7b** was approved by the presence of M^+.^ and M^+.^+2 peaks.

The 3-substituted amino derivatives **8a–f** revealed an NH absorption band. Besides, compounds **8d–f** showed the band of CN function.^1^H-NMR spectra of compounds **8a** and **8d** showed triplet and quartette signals of the ethyl group protons of *N*-ethylpiperazine. In addition, two multiplets of the piperazine-C_3,5_-H and piperazine-C_2,6_-H were observed. Besides, two singlet signals integrated as half proton each were attributed to C_4_-H and the D_2_O exchangeable singlet signal of NH. ^1^H-NMR spectra of compounds **8b** and **8e** demonstrated two multiplets corresponding to morpholine-C_3,5_-H and morpholine-C_2,6_-H, in addition to three singlet signals for C_1_-H, C_4_-H, and a D_2_O exchangeable singlet signal of NH. The signals for C_4_-H and NH were integrated for half proton, each attributed to the presence of two tautomers. On the other hand, the ^1^H-NMR of **8c** and **8f** showed three multiplets of piperidine-C_4_-H, piperidine-C_3,5_-H, and piperidine-C_2,6_-H. Besides that, the spectra showed two singlets; for C_1_-H and for C_4_-H (integrated for half proton) in addition to the NH D_2_O exchangeable singlet signal. ^13^C-NMR spectra of compounds **8a** and **8d** illustrated the carbons of the ethyl group of ethylpiperazine as well as the piperazine ring carbons in the aliphatic region. The number of the other carbons was consistent with the proposed structure. Further, ^13^C-NMR spectra of compounds **8b** and **8e** demonstrated signals of morpholine C-_3,5_ morpholine C-_2,6_. The position and the total number of the signals were consistent with the proposed structures. The piperidine ring in compounds **8c** and **8f** exhibited three signals in the aliphatic region, which were attributed to piperidine C-_4_, piperidine C-_3,5_, and piperidine C-_2,6_. Other carbons in these compounds were observed in the expected chemical shift. The mass spectra of compounds **8c** and **8e** confirmed the presence of their molecular ion peaks. Furthermore, **8c** showed the M**^+.^**+2 peak, indicating the existence of one chlorine atom.

The ^1^H-NMR spectrum of the quinoxaline derivative **9** revealed D_2_O exchangeable signals corresponding to two NH protons of the quinoxaline ring and pyridine-NH proton. Also, the ^13^C-NMR spectrum showed C-_7_ at 57.21 ppm, and the total number of the signals was consistent with the proposed structure. Moreover, the mass spectrum demonstrated the molecular ion peak with the absence of a peak corresponding to M^+.^+2, confirming the absence of the Cl atom of the precursor, which ascertains ring closure to the corresponding quinoxaline.

On the other hand, the IR spectrum of compound **10** showed 2NH absorption bands and the CN band at 2229 cm^−1^. Its ^1^H-NMR spectrum demonstrated two singlet signals corresponding to C_1_-H and C_4_-H (integrated for half proton), in addition to two D_2_O exchangeable signals (integrated for half proton) for 2NH protons. The ^13^C-NMR spectrum of **10** demonstrated two signals in the aliphatic region for C-_4_ and C-_1_, which ensures the existence of the compound in two tautomeric forms.

### 3.2. Cytotoxic activity

#### 3.2.1. *In vitro* anticancer activity against a panel of 60 human tumour cell lines

Herein, twenty-seven newly synthesised compounds; **2a,c**, **3a–d**, **4a–d**, **5a–d**, **6a–c**, **7a,b**, **8a–f, 9**, and **10** were evaluated by National Cancer Institute (NCI, USA) at a single dose (10^−5 ^M) against 60 different human cell lines, representing leukaemia, melanoma, lung, colon, CNS, ovary, kidney, prostate and breast cancer. The growth inhibition percentages for the tested compounds were obtained from a single dose screening, and the mean growth inhibition percent of the treated cells compared to the untreated control cells was calculated for each compound (Tables 1–4). The results revealed that compounds **5a–c** exhibited prominent anticancer activity against almost all human cancer cell lines, with mean growth inhibition ranging from 46.88 to 52.52%.

**Table 1.** Growth inhibition (%) obtained from a single dose (10^−5 ^M) of the tested compounds **2a,c**, **3a–d**, and **4a–c**.

	Compound								
Cell line	2a	2c	3a	3b	3c	3d	4a	4b	4c
Leukaemia									
CCRF-CEM	−5.14	26.28	−5.88	−9.35	−10.16	0.90	−1.02	−0.42	−0.04
HL-60 (TB)	10.97	28.75	−7.79	−7.38	−15.87	12.00	0.73	−5.26	−5.53
K-562 86.10	4.76	67.82	12.84	33.50	2.86	15.78	12.46	31.32	33.50
MOLT-4	4.10	19.80	2.64	0.27	−5.89	2.90	4.69	7.88	11.85
RPMI-8226	−8.46	30.57	−2.54	1.11	4.62	29.04	11.74	7.32	5.90
SR	6.30	37.25	28.66	30.47	0.60	23.81	36.64	39.32	0.20
Non-small cell lung carcinoma									
A549/ATCC	−7.82	4.91	−10.00	58.07	−5.43	−5.27	−4.25	55.45	3.15
EKVX	−5.71	15.63	7.10	23.00	15.84	7.98	2.22	22.96	11.80
HOP-62	5.99	20.37	11.63	8.26	8.52	14.32	9.42	17.16	3.32
HOP-92	−8.14	−11.40	2.88	5.43	7.78	10.96	9.64	16.44	−11.25
NCI-H226	0.31	23.54	13.11	9.99	20.72	21.32	13.57	7.14	18.47
NCI-H23	−4.04	13.26	0.15	−3.36	1.41	1.04	1.72	6.41	6.95
NCI-H322M	0.25	1.98	−3.18	16.25	1.00	2.78	−4.72	22.02	−4.23
NCI-H460	−6.08	59.24	2.47	36.93	23.13	0.53	−1.09	31.42	17.18
NCI-H522	6.14	2.94	4.48	11.40	−1.98	17.55	8.58	21.72	7.95
Colon cancer									
COLO 205	−15.99	−6.61	−16.49	−17.67	−12.74	−7.27	−16.67	−20.46	−21.65
HCC-2998	−12.75	−11.43	−15.27	−12.11	−18.72	−6.11	−7.97	−6.39	−27.42
HCT-116	−3.66	80.50	−0.50	−0.50	14.07	23.07	−2.40	1.62	11.44
HCT-15	−9.73	−1.05	−3.42	8.32	−10.00	21.33	−9.27	14.20	−1.51
HT-29	−9.18	−7.17	−7.86	−10.51	−12.09	0.03	−10.48	−6.98	−9.93
KM12	−5.55	3.27	7.40	4.33	−0.49	3.93	3.86	7.60	−0.66
SW-620	−4.67	68.81	3.98	4.97	20.70	2.40	−0.16	6.49	2.43
CNS cancer									
SF-268	4.51	5.43	8.45	1.85	6.16	14.60	9.64	14.28	10.54
SF-295	−4.69	27.03	−2.52	2.74	−0.58	2.30	−2.33	1.82	−7.26
SF-539	−0.64	3.65	11.49	12.66	10.42	6.60	3.36	15.83	3.36
SNB-19	−2.10	14.24	1.20	7.57	−0.33	6.23	0.33	7.47	−2.16
SNB-75	12.62	−1.55	17.27	25.44	10.03	21.53	22.30	22.84	11.66
U251	−7.91	32.54	2.07	13.61	14.88	20.57	0.40	21.06	8.82
Melanoma									
LOX IMVI	−0.02	11.09	5.88	2.21	2.71	12.77	4.84	6.46	8.72
MALME-3M	4.19	12.55	15.80	11.07	5.53	−6.36	9.21	10.78	9.46
M14	−5.61	−1.11	4.96	6.88	−0.72	1.35	3.25	3.86	1.70
MDA-MB435	−0.27	−12.11	9.40	31.80	−7.97	−3.38	8.55	33.66	−7.17
SK-MEL-2	−17.93	−9.11	−25.13	−12.61	−25.53	−1.12	−23.16	−18.30	−16.71
SK-MEL-28	−7.93	−4.83	−11.36	1.50	−20.20	−9.19	−1.74	−0.07	8.45
SK-MEL-5	−2.91	8.44	2.83	1.99	2.72	5.55	−1.74	−0.07	8.45
UACC-275	−15.16	78.82	−6.31	−12.46	−19.98	−7.53	−12.81	−6.58	−0.18
UACC-62	7.33	60.94	10.62	11.65	12.83	14.07	10.87	8.74	20.84
Ovarian cancer									
IGROV1	0.46	66.51	8.40	10.25	34.55	20.45	10.33	4.86	32.96
OVCAR-3	−7.34	70.82	−0.33	−2.13	2.18	0.09	1.99	0.61	7.25
OVCAR-4	−6.44	40.46	3.35	−0.19	7.00	−1.20	−1.63	7.51	11.14
OVCAR-5	−9.42	43/78	−3.20	−8.59	−8.05	−8.70	−3.64	−12.95	−2.88
OVCAR-8	−4.32	6.75	−3.27	−1.02	−7.33	2.86	−0.71	2.95	1.73
NCI/ADR-RES	−3.61	13.48	−2.37	−2.15	−5.76	4.50	−5.94	9.18	−1.98
SK-OV-3	6.36	2.42	−0.85	−2.91	6.94	−3.95	0.53	−2.04	−9.85
Renal cancer									
786-0	−12.20	−10.98	−7.41	−8.85	−9.68	−10.00	4.93	−0.46	−16.91
A498	−4.03	55.09	9.33	4.22	−15.47	4.88	14.90	11.10	−6.23
ACHN	−4.06	7.28	1.30	−2.10	−0.56	6.70	12.41	−3.20	−4.45
CAKI-1	11.93	85.07	23.33	16.33	24.17	21.82	27.47	17.66	12.41
RXF 393	−27.86	−3.10	−2.63	−18.20	−24.83	−5.23	−0.71	9.68	−17.33
SN 12 C	−0.73	7.57	−0.49	−0.17	0.94	4.04	9.45	3.66	4.76
TK-10	−34.90	−30.93	−29.42	−29.74	−41.81	−52.28	−25.46	−38.15	−51.99
UO-31	24.99	29.91	32.68	25.81	30.62	34.84	40.33	71.67	33.22

NT: not tested.

**Table 2.** Growth inhibition (%) obtained from a single dose (10^−5 ^M) of the tested compounds **4d**, **5a–d**, **6a–c**, and **7a**.

	Compound								
Cell line	4d	5a	5b	5c	5d	6a	6b	6c	7a
Leukaemia									
CCRF-CEM	1.99	28.11	65.96	13.17	−0.08	−2.46	−0.89	1.84	2.12
HL-60 (TB)	−16.76	43.05	89.47	18.79	−0.44	−2.00	3.00	−16.98	−8.33
K-562	22.96	76.14	85.98	64.10	8.17	10.96	6.63	6.11	17.99
MOLT-4	9.18	28.61	50.86	13.47	18.85	9.90	7.90	8.91	15.17
RPMI-8226	9.61	n.t	36.89	58.75	13.99	4.93	14.38	5.15	11.50
SR	11.38	86.10	77.87	33.17	17.30	8.60	14.36	0.30	16.16
Non-small cell lung carcinoma									
A549/ATCC	−2.89	41.53	58.45	52.76	−6.53	−7.34	−0.83	−0.32	2.30
EKVX	0.87	20.71	38.32	35.32	11.52	−2.24	2.98	5.86	7.10
HOP-62	1.22	42.59	54.60	66.27	21.38	8.57	12.74	11.53	15.50
HOP-92	5.89	57.75	45.89	114.94	17.20	−1.02	13.34	14.10	14.70
NCI-H226	5.26	37.80	39.82	31.22	3.95	10.00	19.41	8.71	14.16
NCI-H23	−1.19	35.21	24.79	32.88	−0.19	−1.88	5.78	5.16	8.55
NCI-H322M	−0.93	9.60	34.79	97.99	4.25	0.49	−2.43	2.60	−2.48
NCI-H460	1.43	68.17	71.40	11.49	18.13	0.82	0.21	−0.58	1.24
NCI-H522	8.55	92.03	79.90	17.29	22.38	0.67	10.38	14.03	12.70
Colon cancer									
COLO 205	−17.16	11.65	8.44	29.89	−14.81	−13.79	−10.90	−12.72	−14.59
HCC-2998	−15.95	11.73	8.46	6.16	−14.24	−18.06	−9.62	2.82	−16.11
HCT-116	9.54	77.24	62.45	88.95	7.78	6.68	12.49	20.56	13.38
HCT-15	1.31	63.64	66.48	12.29	0.05	−7.17	−4.99	3.74	−1.63
HT-29	2.30	35.95	49.50	14.72	−2.29	−2.32	3.10	9.00	−1.97
KM12	−5.21	68.99	59.95	8.58	5.70	−0.94	2.35	2.91	3.25
SW-620	3.43	68.68	75.68	88.07	7.44	5.15	7.48	7.46	4.18
CNS cancer									
SF-268	6.94	48.93	38.96	14.44	16.38	7.67	6.10	2.64	5.16
SF-295	−3.10	72.38	69.50	79.16	1.95	−3.39	1.10	2.28	−2.06
Melanoma									
LOX IMVI	5.22	60.33	54.42	8.64	17.44	−2.53	−0.60	2.50	1.24
MALME-3M	4.62	60.34	52.89	25.30	−12.10	−3.44	0.18	−1.85	−2.96
M14	−0.99	62.20	66.71	7.30	−8.86	−1.65	−1.23	1.90	4.95
MDA-MB435	−7.63	−128.6	109.11	−1.70	−6.69	−2.64	−9.63	−8.63	−9.15
SK-MEL-2	−26.10	35.91	39.98	7.40	−13.05	−13.26	−12.67	−8.06	−5.95
SK-MEL-28	−10.50	45.90	47.40	−10.62	−21.56	−12.98	−11.46	−12.43	−11.40
SK-MEL-5	4.05	18.22	39.46	24.88	4.83	−1.71	0.57	1.15	1.94
UACC-275	−15.75	31.78	36.15	137.34	−18.98	−14.74	−10.74	−6.77	−7.18
UACC-62	10.37	58.78	64.69	90.63	12.15	8.90	3.55	4.85	7.18
Ovarian cancer									
IGROV1	22.77	48.25	58.88	99.69	36.61	16.37	21.54	20.70	10.70
OVCAR-3	−4.91	86.28	99.38	114.48	9.36	0.35	1.90	−2.74	−3.96
OVCAR-4	1.96	46.31	28.44	100.46	9.55	−6.65	0.30	−4.39	3.16
OVCAR-5	−3.92	1.24	14.42	115.10	1.54	−3.55	−6.35	−1.56	3.21
OVCAR-8	−1.82	38.70	30.20	25.05	5.37	2.02	2.60	5.00	1.90
NCI/ADR-RES	−8.05	84.39	70.73	31.46	2.18	−2.46	1.78	−1.66	−2.76
SK-OV-3	−10.25	49.28	36.89	28.20	−0.90	−1.47	0.40	−0.84	5.95
Renal cancer									
786-0	−11.18	31.64	29.12	10.02	−6.80	−15.03	−11.10	−6.36	−7.16
A498	−0.03	74.75	68.82	160.74	17.32	0.35	13.54	7.34	3.36
ACHN	6.28	62.51	55.49	34.27	13.54	6.81	4.34	9.32	6.96
CAKI-1	27.40	78.73	60.00	94.73	14.39	18.35	23.04	17.65	18.84
RXF 393	−18.11	90.35	44.07	30.05	−7.73	−24.01	6.95	6.16	7.81
SN 12 C	2.95	26.28	38.64	20.94	5.16	2.15	2.85	2.45	2.54
TK-10	−40.69	1.90	−12.69	58.88	−58.32	−51.93	−28.16	−42.18	−37.25
UO-31	34.70	66.03	64.20	46.19	41.32	35.81	31.84	32.18	30.16
Prostate cancer									
PC-3	3.80	43.20	50.65	20.03	13.20	9.37	10.40	8.67	3.11
DU-145	−8.57	9.51	35.21	10.10	−10.04	−10.81	−9.40	−9.90	−4.75
Breast cancer									
MCF-7	8.44	75.53	67.39	76.38	15.13	0.60	10.32	10.19	5.05
MDAMB231/ATCC	5.22	27.31	28.82	24.49	19.17	−0.79	7.92	0.28	8.09
BT-549	3.62	78.64	44.61	10.82	8.35	−0.58	1.66	1.61	0.77
T-47D	8.70	38.33	19.88	97.49	20.13	−1.25	15.40	8.12	17.74
MDA-MB-468	−8.27	75.70	66.31	149.2	−6.48	−7.69	−3.26	−9.31	−4.71

NT: not tested.

**Table 3.** Growth inhibition (%) obtained from a single dose (10^−5 ^M) of the tested compounds **7b**, **8a–f**, **9**, and **10**.

	Compound								
Cell line	7b	8a	8b	8c	8d	8e	8f	9	10
Leukaemia									
CCRF-CEM	3.22	1.89	5.00	30.31	−0.37	6.67	3.32	−1.14	2.96
HL-60(TB)	1.47	−9.32	−10.05	18.70	−15.57	−16.49	−3.35	−3.65	−13.14
K-562	8.80	13.16	18.62	14.11	4.11	9.66	23.09	16.65	2.72
RPMI-8226	12.71	15.37	16.16	8.74	1.72	6.66	14.95	12.11	1.46
MOLT-4	7.93	5.94	13.57	35.58	8.62	5.54	5.31	−1.99	6.48
SR	15.93	23.49	20.41	10.06	11.80	9.91	11.09	16.52	13.03
Non-small cell lung carcinoma									
A549/ATCC	−10.05	1.80	−0.74	5.05	−2.72	0.10	−2.65	−2.98	−7.45
EKVX	−1.36	4.50	14.33	19.13	12.48	9.90	5.51	4.82	9.27
HOP-62	9.67	3.76	16.05	5.22	16.11	18.01	10.21	9.45	8.27
HOP-92	10.38	10.71	22.19	9.27	18.88	11.76	4.90	−1.53	9.60
NCI-H226	9.35	16.96	20.94	28.22	15.72	10.44	15.67	10.44	12.84
NCI-H23	4.25	7.10	5.35	3.42	3.90	2.35	4.09	0.22	−1.96
NCI-H322M	−3.17	−1.60	0.04	−0.59	1.85	3.90	−5.64	−1.56	−0.11
NCI-H460	2.14	0.60	6.40	3.00	−1.12	2.61	2.20	1.16	−1.10
NCI-H522	−1.38	14.34	17.30	17.71	4.68	8.18	9.54	4.63	−2.13
Colon cancer									
COLO 205	−14.62	−11.90	−12.90	−12.73	−5.97	−17.44	−16.69	−17.10	−13.32
HCC-2998	−18.20	−2.53	−13.48	−6.85	−12.24	−8.91	−23.24	−17.51	−20.34
HCT-116	11.73	15.00	25.30	1.95	1.31	8.86	4.50	−1.05	3.44
HCT-15	−7.25	2.50	4.11	3.84	−6.34	−6.78	−4.78	−4.08	−5.46
HT-29	−3.29	2.30	0.78	−2.62	−4.51	−5.64	−4.42	−5.27	−14.25
KM12	−0.12	−1.16	6.99	3.41	−0.49	0.52	5.13	−0.46	0.14
SW-620	3.78	4.03	7.44	1.71	3.43	5.32	4.11	2.00	2.45
CNS cancer									
SF-268	6.82	7.38	7.66	6.19	4.87	7.51	9.44	7.06	4.70
SF-295	−4.69	0.40	4.37	−6.60	−1.19	−6.46	−9.62	−7.10	−4.19
SF-539	3.78	4.31	6.36	−6.63	6.11	3.33	6.49	3.93	6.04
SNB-19	−0.67	1.26	1.40	−3.36	−1.27	−0.92	1.38	−0.03	−3.45
SNB-75	8.28	11.85	10.40	22.37	8.49	7.28	5.45	10.22	10.11
U251	−7.36	6.16	6.85	−7.81	0.90	−2.20	−6.11	−3.59	−9.90
Melanoma									
LOX IMVI	1.46	6.70	8.33	−0.70	4.73	3.90	2.61	3.13	3.26
MALME-3M	−2.31	1.57	−3.09	4.61	−7.54	−7.29	−10.60	−7.56	−15.06
M14	−5.17	6.73	7.49	1.39	4.65	−1.97	0.58	1.33	1.68
MDA-MB435	−8.18	−6.27	−2.51	2.00	−7.84	−7.70	−6.93	−7.00	−4.45
SK-MEL-2	−2.07	3.85	8.89	2.66	1.77	0.38	2.71	3.87	0.67
SK-MEL-28	−11.22	−10.83	−7.54	−16.73	−15.82	−10.55	−10.68	−7.98	−10.97
SK-MEL-5	−20.08	−14.07	−8.74	0.27	−10.67	−14.59	−14.83	−22.04	−19.64
UACC-257	−9.77	−7.62	−3.74	−10.30	−23.69	−6.70	−12.71	−15.24	−23.17
UACC-62	8.84	11.63	17.50	8.57	6.81	6.70	15.47	11.48	6.75
Ovarian cancer									
IGROV1	15.63	23.83	16.17	17.36	23.66	25.41	14.95	24.22	16.09
OVCAR-3	−1.67	1.03	2.72	−1.68	−2.65	0.17	0.09	−3.93	−1.64
OVCAR-4	−3.81	3.25	13.48	12.27	−0.52	−2.02	0.90	−2.74	−3.90
OVCAR-5	−3.54	1.90	8.49	−1.61	−4.10	0.99	5.19	3.15	−3.49
OVCAR-8	−4.49	2.09	1.71	−6.18	−1.56	4.28	−0.33	−3.40	−6.58
NCI/ADR-RES	1.73	−1.63	1.45	−2.62	−4.88	−0.28	−1.61	−6.50	−8.27
SK-OV-3	2.24	1.74	−1.60	9.90	11.46	7.67	7.22	−1.70	10.13
Renal cancer									
786-0	−13.52	−7.31	−6.62	−0.24	−9.68	−9.79	−12.07	−10.32	−12.31
A498	19.72	0.93	10.43	N.T	12.11	3.17	−0.87	−0.12	3.94
ACHN	4.44	9.21	9.98	6.20	9.28	11.93	14.30	15.47	9.97
CAKI-1	19.82	21.90	32.49	18.80	25.23	27.35	28.70	28.29	21.11
RXF 393	−15.77	−2.44	11.89	−15.60	−3.67	−2.93	−6.31	−15.26	−5.32
SN 12 C	4.43	2.25	3.71	3.66	1.95	6.28	3.70	1.71	0.49
TK-10	−53.79	−41.70	−39.72	−22.35	−36.96	−59.32	−53.09	−54.74	−55.35
UO-31	32.54	35.83	38.65	37.95	33.48	38.44	33.28	36.06	31.23
Prostate cancer									
PC-3	3.50	7.70	14.53	11.89	9.10	12.29	8.87	7.47	7.80
DU-145	−14.25	−7.24	−1.71	−12.51	−11.15	−12.31	−8.32	−11.41	−8.61
Breast cancer									
MCF-7	8.22	4.08	20.98	23.32	9.44	−1.68	−0.95	1.91	4.92
MDA-MB 231/ATCC	7.25	12.03	18.84	13.93	19.77	13.40	15.05	17.76	17.40
BT-549	−2.93	9.38	4.09	−10.51	1.51	1.14	1.03	5.02	1.04
T-47D	−9.14	11.06	20.78	17.49	22.26	−4.79	2.45	−5.91	−2.94
MDA-MB-468	−7.25	−9.64	1.73	−13.89	−2.69	−11.00	−8.03	−10.57	−7.35

**Table 4.** Mean growth inhibition (%) for the tested compounds **2a,c**, **3a–d**, **4a–d**, **5a–d**, **6a–c**, **7a,b 8a–f**, **9**, and **10**.

Compound	Mean growth inhibition (%)	Compound	Mean growth inhibition (%)
**2a**	−3.74	**6a**	−0.71
**2c**	11.47	**6b**	3.19
**3a**	6.67	**6c**	2.54
**3b**	6.02	**7a**	3.15
**3c**	1.33	**7b**	2.14
**3d**	6.24	**8a**	−0.20
**4a**	1.85	**8b**	6.10
**4b**	8.66	**8c**	7.22
**4c**	3.00	**8d**	1.33
**4d**	0.15	**8e**	1.30
**5a**	52.52	**8f**	0.008
**5b**	52.47	**9**	7.87
**5c**	46.88	**10**	−0.095
**5d**	5.00		

#### 3.2.2. Determination of GI_50_, IC_50_, TGI, and LC_50_ on full NCI-60 cell panel

Compound **5c** was further selected by National Cancer Institute (NCI, USA) to be evaluated against 60 cell lines at 5 dose concentration levels (0.01, 0.1, 1, 10, and 100 µM). The results were exhibited in terms of four response parameters: median growth inhibition (GI_50_, the compound’s concentration that causes a 50% decrease in net cell growth), half-maximal inhibitory concentration (IC_50_, the concentration of drug which exhibited 50% cell viability_)_ , total growth inhibition (TGI, the compound’s concentration leading to total inhibition of cell growth) and median lethal concentration (LC_50_, the compound’s concentration causing a net 50% loss of initial cells at the end of the incubation period) as well as mean graph midpoints (MG-MID) to obtain an average activity parameter overall tested cell lines (Table 5). The *in-vitro* screening revealed that compound **5c** exhibited potent broad-spectrum anticancer activity against almost all human cancer cell lines showing GI_50_ range between 4 nM to 37 µM with MG-MID GI_50_, LC_50_, and TGI values of 0.21, 0.03, and 0.01 µM, respectively. Moreover, compound **5c** exerted GI_50_ at a submicromolar concentration (<1 µM) in 13 tested cancer cell lines.

**Table 5.** GI_50_, IC_50_, LC_50_, and TGI of compound **5c** on 60 cancer cell lines.

	**5c** (µM)			
Cell lines	GI_50_	IC_50_	LC_50_	TGI
Leukaemia				
CCRF-CEM	26.30	4.43	100	100
HL-60(TB)	14.20	4.55	60	100
K-562	1.98	5.50	80.19	100
MOLT-4	19.10	4.51	100	100
RPMI-8226	10.60	4.46	100	100
SR	14.10	4.60	100	100
Non-small cell lung carcinoma				
A549/ATCC	1.31	5.63	29.80	100
EKVX	2.69	4.86	28.90	100
HOP-62	10.00	4.64	39.20	100
HOP-92	12.60	4.10	49.90	100
NCI-H226	0.27	6.06	2.55	100
NCI-H23	7.33	4.71	36.50	100
NCI-H322M	7.37	4.63	37.30	100
NCI-H460	0.29	6.43	10.20	100
NCI-H522	18.40	4.38	57.60	100
Colon cancer				
COLO 205	10.30	4.75	55.30	100
HCC-2998	17.40	4.47	40.50	94.90
HCT-116	0.14	6.71	12.10	44.50
HCT-15	11.40	4.78	54.90	100
HT29	19.60	4.59	100	100
KM12	19.90	4.49	48.80	100
SW-620	0.37	6.17	100	100
CNS cancer				
SF-268	16.40	4.33	53.40	100
SF-295	0.60	4.98	32.60	100
SF-539	23.00	4.33	71.00	100
SNB-19	24.60	4.35	100	100
SNB-75	6.12	4.00	94.00	100
U251	2.50	4.90	50.90	100
Melanoma				
LOX IMVI	15.30	4.60	33.50	73.20
MALME-3M	22.60	4.08	61.90	100
M14	16.30	4.55	44.30	100
MDA-MB-435	27.20	4.42	100	100
SK-MEL-2	12.70	4.47	49.80	100
SK-MEL-28	31.20	4.18	100	100
SK-MEL-5	13.00	4.68	29.60	67.60
UACC-257	1.73	5.23	5.44	100
UACC-62	0.36	5.75	4.61	47.20
Ovarian cancer				
IGROV1	0.004	6.75	4.12	100
OVCAR-3	0.32	6.17	15.40	87.80
OVCAR-4	1.61	4.61	72.10	100
OVCAR-5	0.73	5.47	4.12	100
OVCAR-8	12.00	4.69	73.20	100
NCI/ADR-RES	8.21	4.78	34.50	100
SK-OV-3	12.00	4.35	73.70	100
Renal cancer				
786-0	37.00	4.11	100	100
A498	1.32	5.51	2.81	5.98
ACHN	15.70	4.53	100	100
CAKI-1	0.17	6.25	4.53	100
RXF 393	11.30	4.43	41.30	100
SN12C	18.60	4.46	92.80	100
TK-10	17.70	4.14	70.70	100
UO-31	10.70	4.71	24.30	55.20
Prostate cancer				
PC-3	13.60	4.60	90.50	100
DU-145	10.50	4.72	41.80	100
Breast cancer				
MCF7	0.26	6.26	50.60	10.00
MDA-MB-231/ATCC	11.60	4.48	52.30	100
HS 578 T	18.10	4.00	71.30	100
BT-549	16.50	4.14	74.70	100
T-47D	0.32	4.00	44.00	100
MDA-MB-468	0.15	6.62	0.27	0.65
MG-MID	0.21		0.03	0.01

GI_50_: the compound’s concentration that cause 50% decrease in net cell growth; IC_50_: the concentration of drug which exhibited 50% cell viability; LC_50_: the compound’s concentration causing a net 50% loss of initial cells at the end of the incubation period; TGI: the compound’s concentration leading to total inhibition of cell growth.

#### 3.2.3. *In vitro* MTT cytotoxicity assay

**Figure 3.**
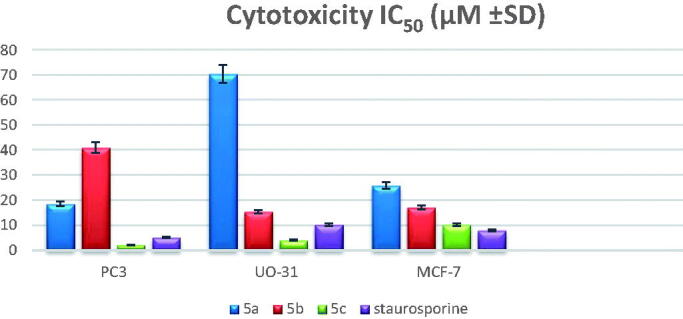
Graphical representation of IC_50_ of the tested compounds **5a–c** compared to staurosporine as a reference standard.

Compounds **5a–c** were subjected to MTT cytotoxicity assays based on the results of one dose screening, particularly against prostate cancer (PC-3), renal cancer (UO-31), and breast cancer (MCF-7) cell lines, and their IC_50_ was determined using staurosporine as the reference drug. The mean estimations of three-fold experiments are represented in (Figure 3). Compound **5c** showed higher potency than the reference drug staurosporine against the PC-3 and UO-31 cell lines, but it exhibited half potency against the MCF-7 cell line. On the other hand, compounds **5a,b** generally showed moderate activity against the three cancer cell lines, except for compound **5a** which exhibited weak activity against the UO-31 cell line, compared to the reference drug. Furthermore, compound **5c** demonstrated fourfold the potency of the abiraterone reference drug against the PC-3 cell line (IC_50_ 8.3 M).

#### 3.2.4. *In-vitro* cytotoxicity on WI-38 human cell line

**Figure 4.**
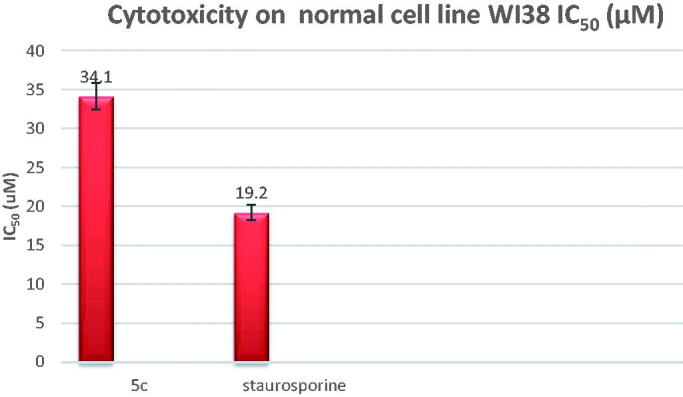
*In-vitro* cytotoxicity (IC_50_) of compound **5c** and staurosporine on WI-38 human cell line.

Compound **5c** was further evaluated against the WI-38 human cell line (Normal cell composed of fibroblasts and derived from lung tissue of a 3-month-gestation aborted female fetus). The tested compound **5c** showed low cellular cytotoxicity with an IC_50_ of 34.1 µM compared to the reference drug staurosporine (IC_50_= 19.2 µM), as shown in Figure 4.

#### 3.2.5. Cyp17 enzyme inhibition assay

**Figure 5.**
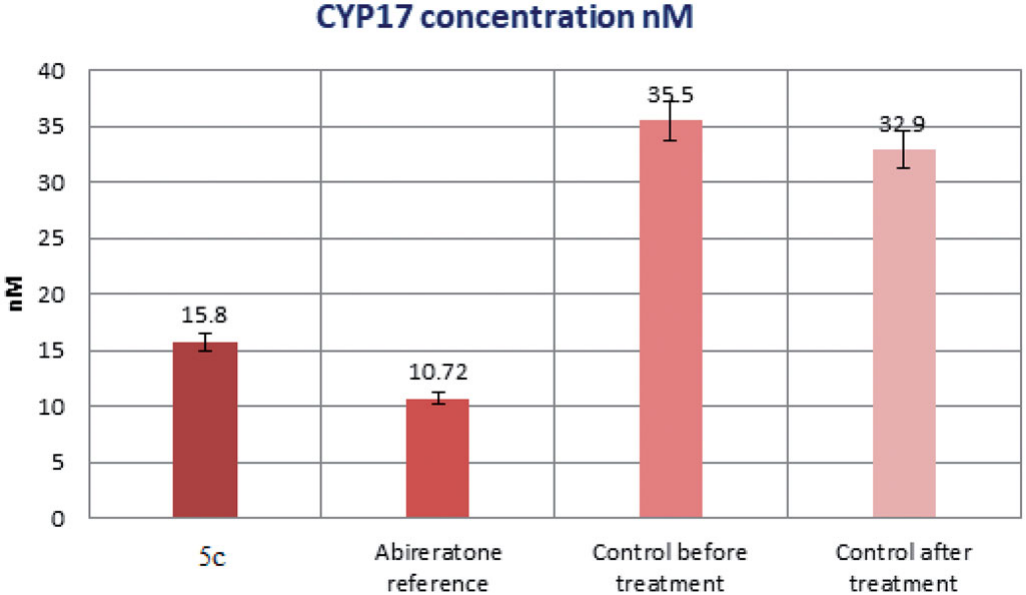
The effect of compound **5c** and abiraterone reference on CYP17 enzyme compared to control.

MTT cytotoxicity assay showed that compound **5c** had high cytotoxic activity against PC-3 cancer cell line and was selected by NCI for further screening at a five-dose concentration level since it was the most active of the other tested derivatives. On this basis, the effect of compound **5c** was further evaluated as a CYP17 enzyme inhibitor in mice with prostate cancer in comparison to the abiraterone reference drug. Afterward, *in vitro* ELISA quantitative measurement of CYP17 enzyme activity in mice tissue was carried out. The results showed that compound **5c** was able to decrease the enzyme concentration to 15.80 nM, which was almost comparable to the reference drug (Figure 5).

#### 3.2.6. Testosterone assay

**Figure 6.**
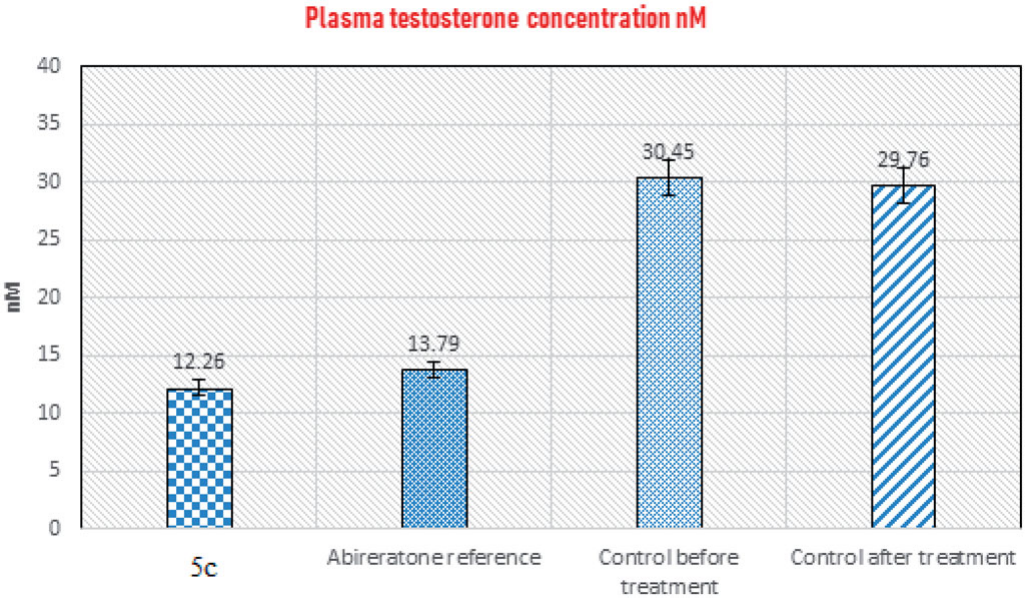
The effect of compound **5c** and abiraterone reference on plasma testosterone.

Furthermore, compound **5c** was tested for its ability to inhibit testosterone production in serum mice prostate cancer models. The plasma concentration of testosterone in the samples of each group was quantified using testosterone Biovendor rat ELISA assay. Testosterone inhibitory activity was compared to abiraterone as a reference standard. The results revealed that compound **5c** had 1.1 folds the potency of abiraterone (Figure 6).

#### 3.2.7. Cell cycle analysis

**Figure 7.**
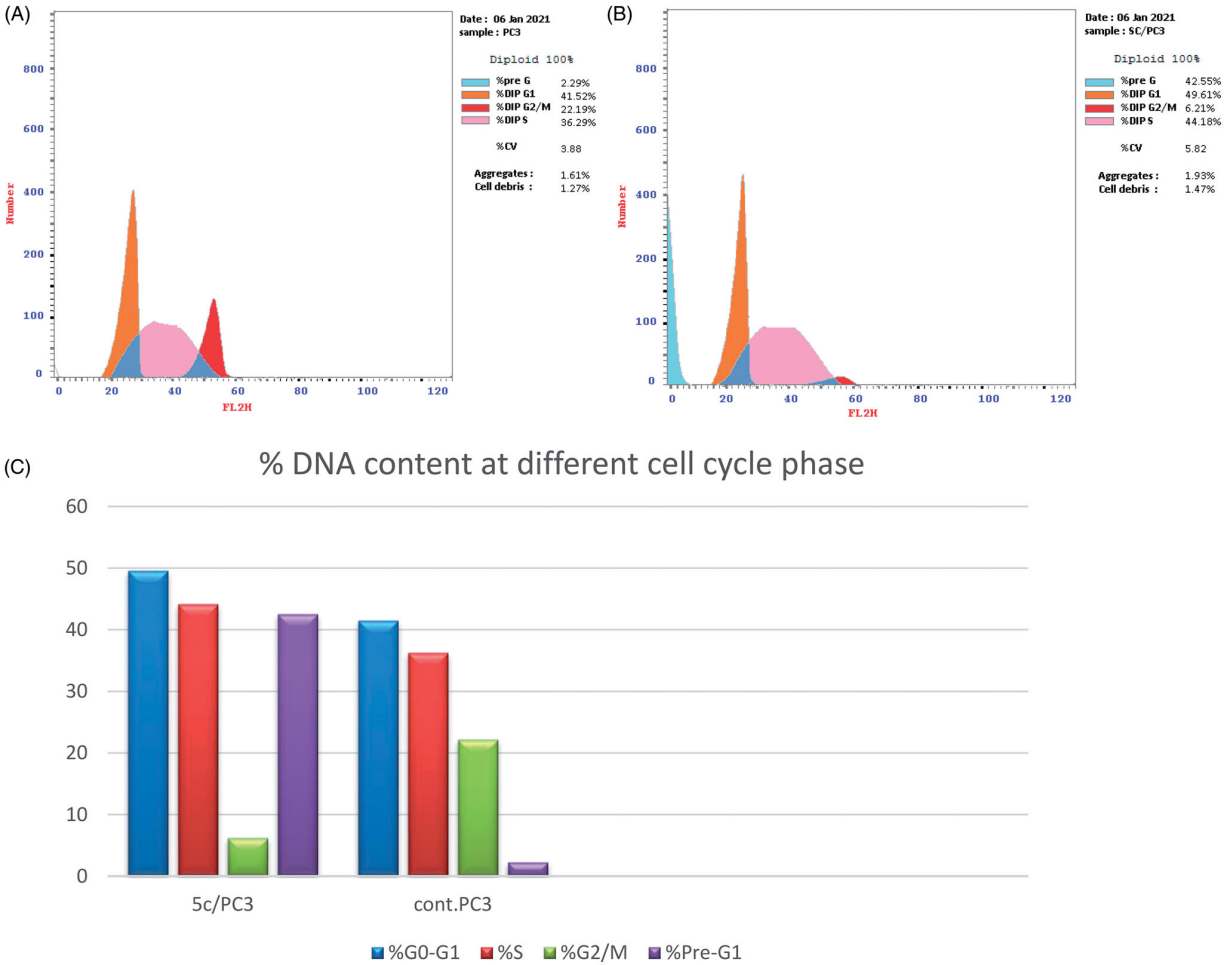
(A) Cell cycle analysis of PC-3 treated with DMSO only. (B) Cell cycle analysis of PC-3 after treatment with **5c** (2.34 µM). (C) Graphical representation of effect of compound **5c** on cell cycle profile of PC-3 cells.

To investigate the mechanism of the antiproliferative activity of the most active compound on the cell cycle progression, the prostate cancer PC-3 cells were treated with compound **5c** for 24 h then analysed. The results indicated that compound **5c** increased the accumulation of cells at both G0/G1-phase (49.61%) and S-phase (44.18%) compared to 41.52 and 36.29% of the control cells, respectively (Figures 7(A–C)). In addition, compound **5c** significantly suppressed cell accumulations in the G2/M-phase from 22.19 to 6.21%. Furthermore, compound **5c** showed a marked increase in cells in Pre-G1-phase by 15.58-fold from 2.29 to 42.55%, thereby indicating the induction of apoptosis.

#### 3.2.8. Annexin V-FITC apoptosis determination

**Figure 8.**
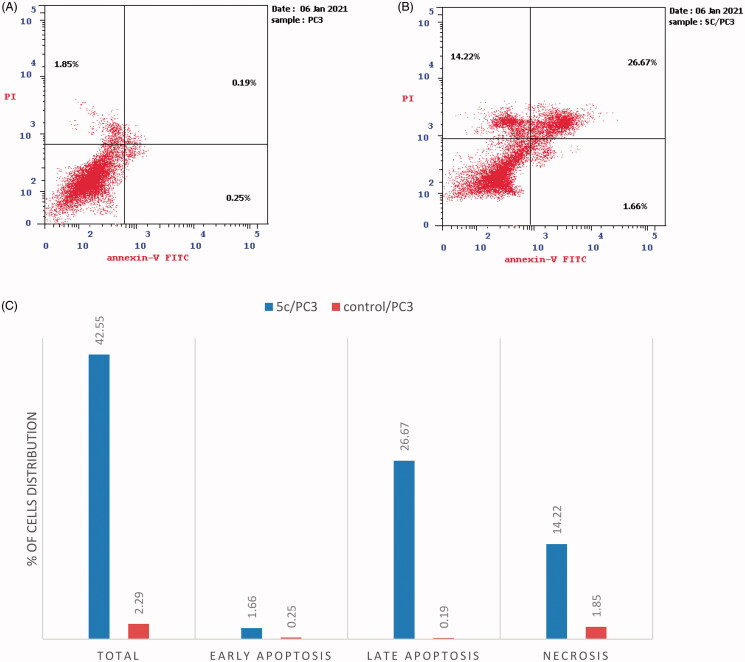
(A) Apoptosis and necrosis in prostate cancer PC-3 cell line. (B) The effect of compound **5c** (2.34 µM) on apoptosis and necrosis in prostate cancer PC-3 cells. (C) Graphical representation of the effect on apoptosis and necrosis of **5c** on PC3 cell line in comparison with control cells.

Apoptosis has significant effects on both carcinogenesis and cancer treatment. A large number of synthetic and natural compounds have been reported to be effective against several cancer diseases through the induction of apoptosis in their target cells^39^. Herein, the apoptotic potential of **5c** was further studied using Annexin V-FITC assay in prostate cancer PC-3 cell line. The results expressed in (Figures 8(A–C)) showed a significant increase in the early and late apoptotic cells by 6.64- and 140.36-fold, respectively, in addition to the elevation in the percentage of necrosis by 7.68-fold with a total increase by 18.58-fold as compared to the control cells.

#### 3.2.9. *In silico* physicochemical properties, ADMET profiles, and drug-likeness data of 5c compared to abiraterone

**Figure 9.**
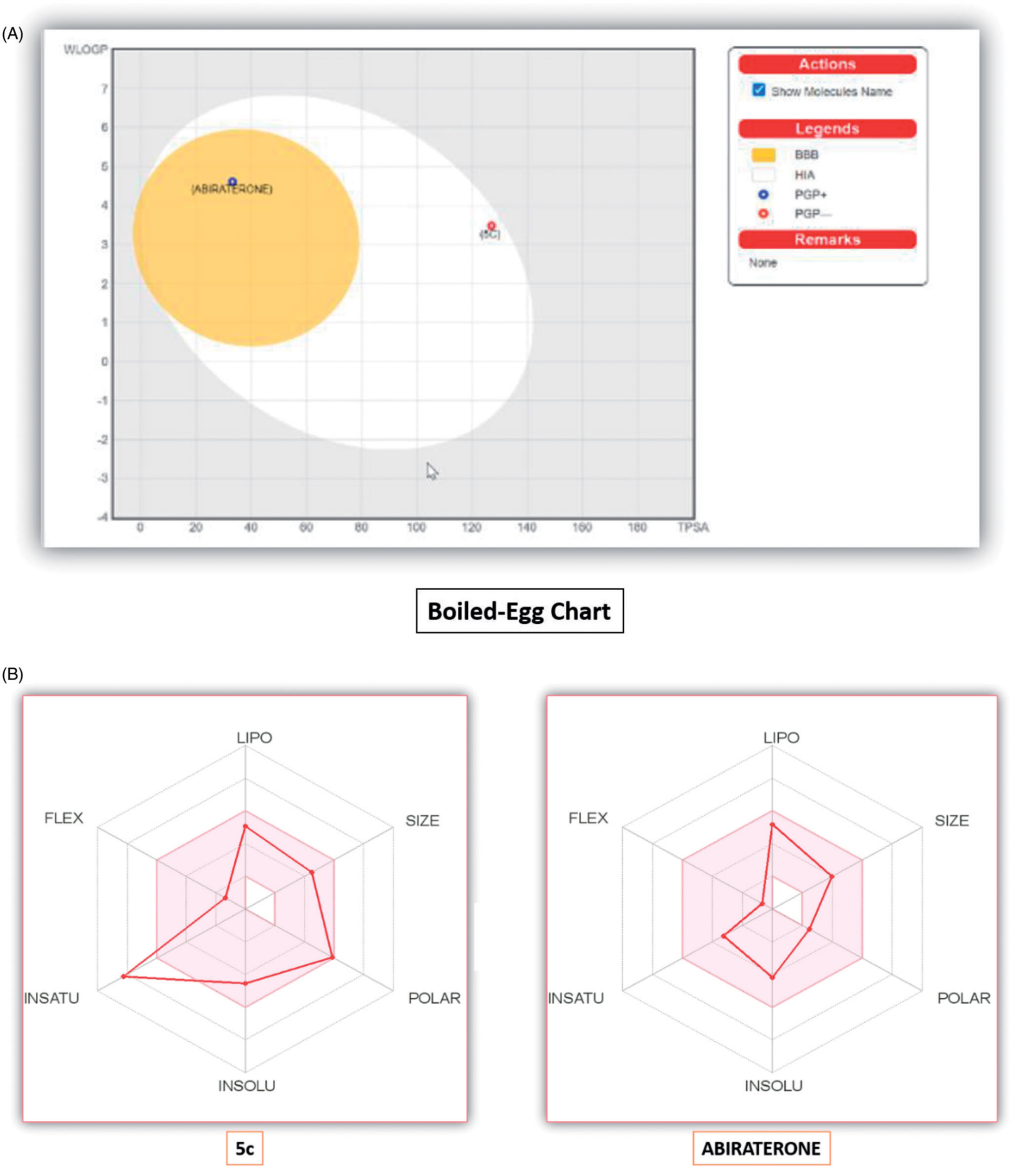
(A) The boiled-egg chart of the compound **5c** and the reference abiraterone. (B) The bioavailability radar chart for compound **5c** and abiraterone.

Novel molecules with appropriate pharmacokinetic or pharmacodynamic features are considered potential drug candidates. The efficacy of newly synthesised compounds is determined by their biological activity as well as their physicochemical, pharmacokinetic, and drug-likeness properties. Swiss ADME Online software (www.SwissADME.ch) has been used to measure *in silico* ADME profile of the compound **5c** in comparison with abiraterone as a reference drug. The Boiled-Egg chart^40^ showed that **5c** is expected to be highly absorbable by the gastrointestinal tract, similar to the standard abiraterone, due to its location in the human intestinal absorption (HIA) area. Also, the target compound **5c** was characterised by a lack of BBB permeability, unlike abiraterone, implying that it will not reach the CNS (Figure 9(A)). Furthermore, the metabolism of compound **5c** is postulated to inhibit four of the five main cytochrome P-450 (CYP) isoforms (CYP2C9, CYP1A 2, CYP2D6, CYP2C19, and CYP3A4) in the liver, indicating that **5c** should be administered in a time interval with any other medications to avoid any potential drug-drug interactions (Table 6).

**Table 6.** The *in silico* predicted ADME profiles for compound **5c** and abiraterone.

Molecule	GI absorption	BBB permeability	Pgp substrate	CYP1A2 inhibitor	CYP2C19 inhibitor	CYP2C9 inhibitor	CYP2D6 inhibitor	CYP3A4 inhibitor
**5c**	High	No	No	Yes	Yes	Yes	No	Yes
Abiraterone	High	Yes	Yes	Yes	No	No	Yes	No

The oral bioavailability of the compound **5c** and abiraterone is demonstrated in the radar chart (Figure 9(B)). It includes six important oral bioavailability parameters; SIZE (size), POLAR (polarity), INSATU (saturation), LIPO (lipophilicity), INSOLU (solubility), and FLEX (flexibility). The radar chart's pink area represents the ideal range for each value of the six parameters, and the red lines indicate the computed physicochemical features of the compound being studied. The measured physicochemical properties for **5c** were located in the ideal pink area for the parameters, except for the INSATU parameter, which demonstrated a violation.

The physicochemical properties of **5c** are shown in Table 7. The molecular weight of **5c** is < 500 Da, proposing its easy diffusion and absorption *via* the cell membrane. Also, **5c** is supposed to possess a strong membrane permeability, as it fulfils the ideal log *p*-values. Furthermore, compound **5c** was displayed ideal H-bond acceptors (5) and H-bond donors (1) that enhance water solubility and allow the molecule to transmit with passive diffusion *via* the aqueous pores of biological membranes. Moreover, **5c** includes four rotatable bonds, every single bond attached to a heavy atom, which proposes reasonable molecular flexibility. On the other hand, it showed moderate TPSA generated by the molecule's polar atoms. NRB and TPSA have revealed that **5c** has interesting oral bioavailability. In general, compound **5c** demonstrated almost comparable physicochemical properties to abiraterone (Table 7).

**Table 7.** *In silico* physicochemical properties for compound **5c** and abiraterone.

Molecule	MW < 500	Log *P*_o/w_ < 5	HBA < 10	HBD < 5	#Heavy atoms	NRB < 5	TPSA Ǻ^2^ < 160	Log S
**5c**	367.35	2.61	5	1	26	2	126.95	4.54*
Abiraterone	321.46	3.79	2	1	24	1	33.12	5.50*

MW: molecular weight; Log *P*_o/w_: partition coefficient octanol/water; HBA: number of H-bond acceptors; HBD: number of H-bond donors; NRB: number of rotatable bonds; TPSA: topological polar surface area; Log S: aqueous solubility

*Moderately soluble.

The SwissADME Web-tool illustrated that the studied compound **5c** compiled almost all rules of drug-likeness created by the leading pharmaceutical companies; Lipinski's (Pfizer),^41^ Ghose's (Amgen)^42^, Veber's (GSK)^43^, Egan's (Pharmacia)^44^, and Muegge's (Bayer)^45^ filters. One of the most important identifications of drug-like compounds is Lipinski and Veber rules. Lipinski's rules are concerned with identifying compounds that have absorption and permeability issues, whereas Veber's rules indicate the topological polar surface area and molecular flexibility, both of which are important in determining oral bioavailability. The investigated compound **5c** was completely aligned with both Lipinski and Veber’s rules. From the point of view of medicinal chemistry, compound **5c** lacks PAINS (Pan Assay Interference Structures) alerts^46^ (Table 8), which emphasises the absence of interference of **5c** in any protein test, supposing that the results obtained from *in vitro* bioassays should be robust.

**Table 8.** The drug-likeness of compound **5c** and the reference abiraterone drug.

Molecule	Lipinski #violations	Ghose #violations	Veber #violations	Egan #violations	Muegge #violations	Bioavail. Score	PAINS #alerts
**5c**	0	0	0	0	0	0.55	0
Abiraterone	0	0	0	0	0	0.55	0

### 3.3. Structure-activity relationship study

**Figure 10.**
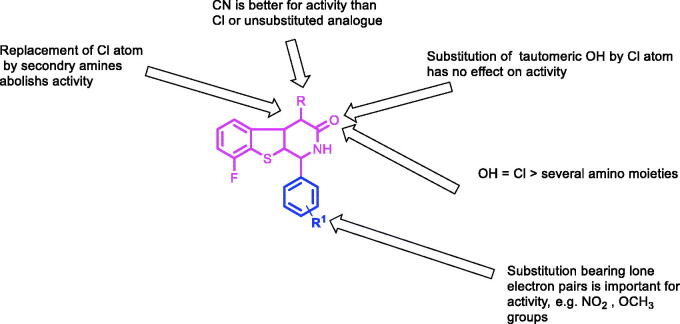
SAR study of the synthesised benzothienopyridine derivatives.

SAR studies of the tested compounds revealed that compounds **5a–c** with a CN group at C-_4_ were of particular interest since they demonstrated potent anticancer activity with growth inhibition percentages ranging from 18.98 to 109.11% when compared to the unsubstituted and chloro substituted analogs **3a–d** and **4a–d**, respectively. The replacement of the Cl substituent at C-_4_ in compound **4d** with secondary amines (compounds **6a–c**) was found to be unfavourable for cytotoxicity. Furthermore, the substitution of 3-OH in **4d** and **5d** by a Cl atom (compounds **7a,b**) does not affect activity. However, replacing Cl in compounds **7a,b** with certain amino compounds in compounds **8a–f**, **9**, and **10** resulted in a decrease in cytotoxic activity (Figure 10).

## 4. Conclusions

Several [1]benzothieno[2,3-*c*]pyridine derivatives were synthesised and selected by NCI, USA, for one-dose anticancer screening. Among these, compounds **5a–c**, possessing CN group at C-_4_, demonstrated potent anticancer activity with GI_50_% ranging from 18.98 to 109.11%. Five dose screening of **5c** exerted broad-spectrum anticancer activity with a GI_50_ range of 4 nM-37 µM. The IC_50_ of compounds **5a–c** was determined against three cancer cell lines using *in vitro* MTT cytotoxicity assay. The results revealed that compound **5c** was the most active derivative against prostate cancer PC3 cell line, with an IC_50_ of 2.08 µM, almost double the activity of staurosporine (IC_50_ = 5.10 µM) and quadruple the activity of abiraterone (IC_50_ = 8.3 µM) reference drugs. In addition, compound **5c** showed low cellular cytotoxicity on the WI-38 human cell line with an IC_50_ of 34.1 µM when compared to staurosporine (IC_50_= 19.2 µM). Furthermore, compound 5c inhibited the CYP17 enzyme in mice prostate cancer models at concentrations ranging from 35.50 to 15.80 nM and was nearly equipotent to abiraterone in decreasing plasma testosterone levels in prostate cancer-treated mice. Moreover, compound **5c** induced a significant disruption in the cell cycle profile besides marked induction of apoptosis. It also exhibited significant physicochemical properties and drug-likeness *via* analysing ADME parameters and drug-likeness data. On this basis, [1]benzothieno[2,3-*c*]pyridines have proven to be an attractive chemical scaffold with potential cytotoxic properties, and compound **5c** is a potentially active orally absorbed CYP17 inhibitor with lower cytotoxicity on normal cells than steroidal derivatives, such as abiraterone.

## Supplementary Material

Supplemental Material

## Abbreviations

**Abbreviations**
CYP17: cytochrome P450 17α-hydroxylase/C17,20-lyase
ADME: absorption, distribution, metabolism, and excretion
NCI-USA: National Cancer Institute-United States of America
PC-3: prostate cancer cell line
UO-31: renal cancer cell line
MCF-7: breast cancer cell line
PSA: prostate specific antigen
DRE: digital rectal exam
PBH: prostatic hyperplasia
ADT: androgen deprivation therapy
AR: androgen receptors
m-CRPC: metastatic castrate-resistant prostate cancer
ELISA: enzyme-linked immunoassay
MTT: 3-(4,5-dimethylthiazol-2-yl)-2,5-diphenyl-2H-tetrazolium bromide
CNS: central nervous system
