# Correction

**DOI:** 10.1080/14756366.2021.1971880

**Published:** 2021-09-06

**Article title:** New benzothieno[2,3-*c*]pyridines as nonsteroidal CYP17 inhibitors: Design, synthesis, anticancer screening, apoptosis induction and *in silico* ADME profile studies.

**Authors:** Nadia A. Khalil, Eman M. Ahmed, Ashraf F. Zaher, Eman A. Sobh, Samiha A. El-Sebaey, Mona S. El-Zoghbi.

**Journal**: Journal of Enzyme Inhibition and Medicinal Chemistry

**DOI**: https://doi.org/10.1080/14756366.2021.1958212

Figures in this article should be changed as below:

Explanation regarding the change

1. Please change figure 1 with the below figure

**Figure 1.**
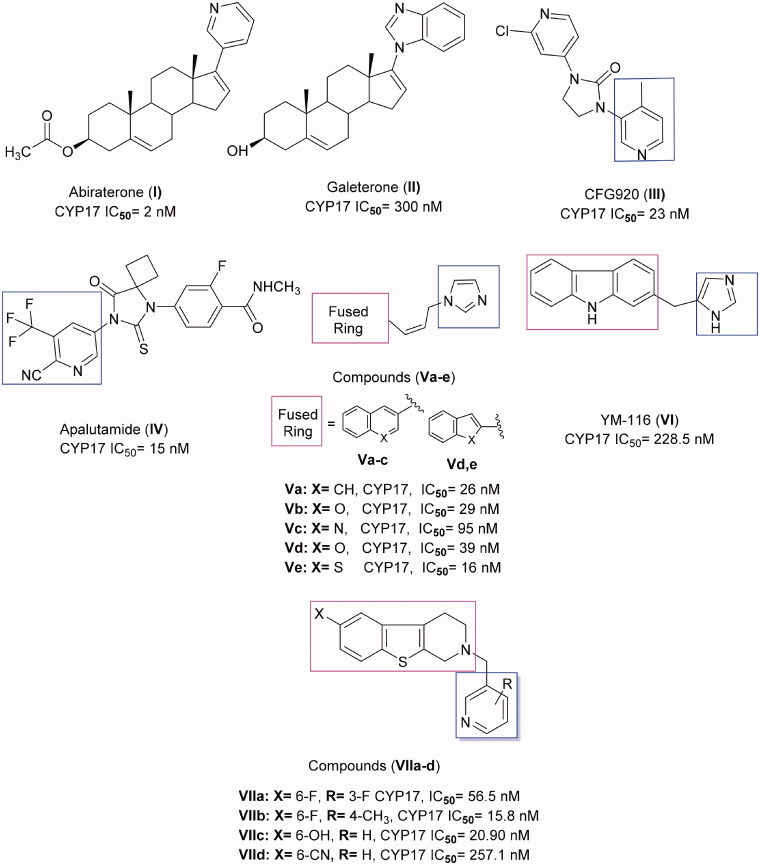
Certain active CYP17 inhibitors.

2. Please change figure 2 with the below figure

**Figure 2.**
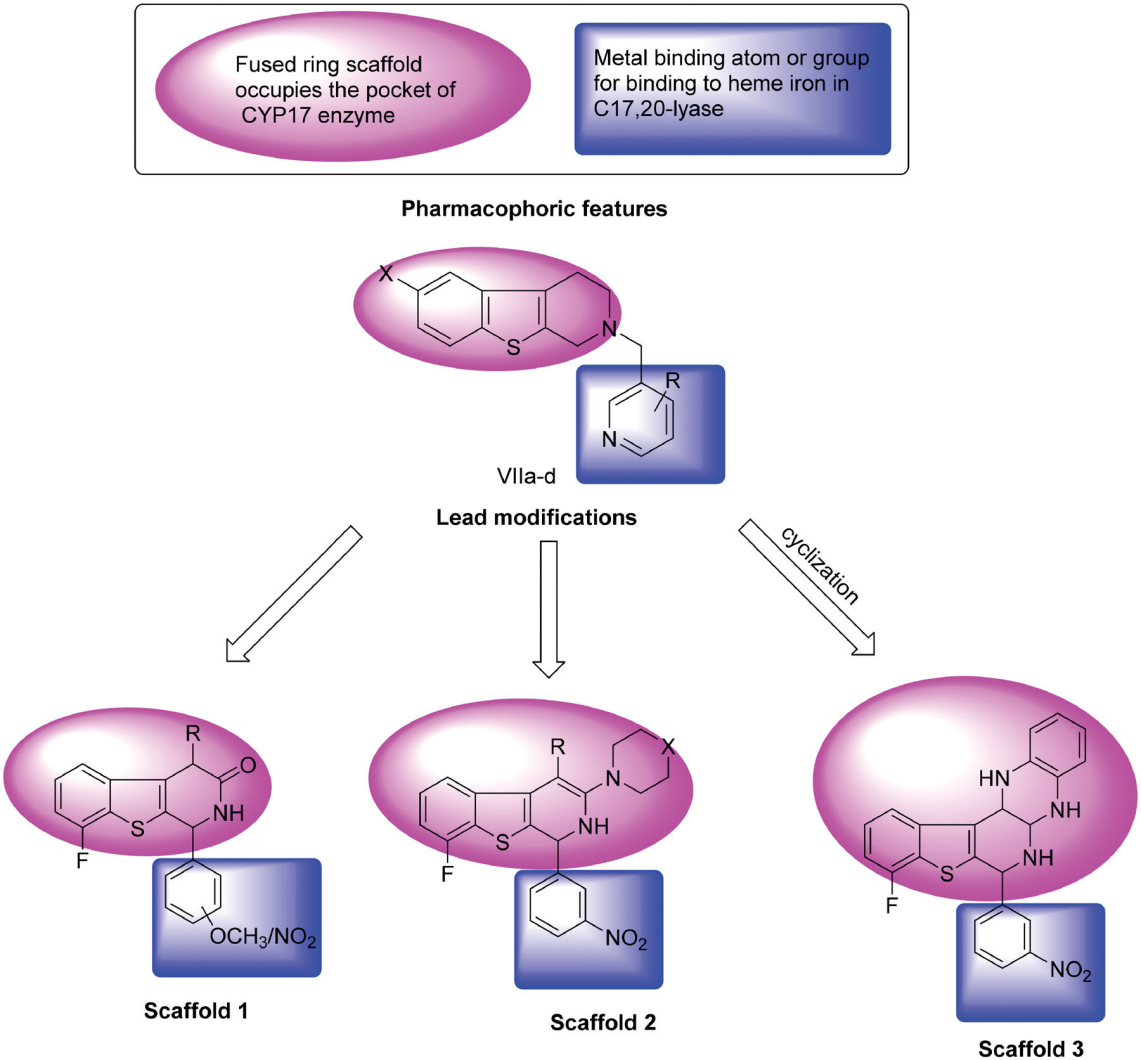
Rational of molecular design of new CYP17 inhibitors.

3. Please change figure 3 with the below figure

**Figure 3.**
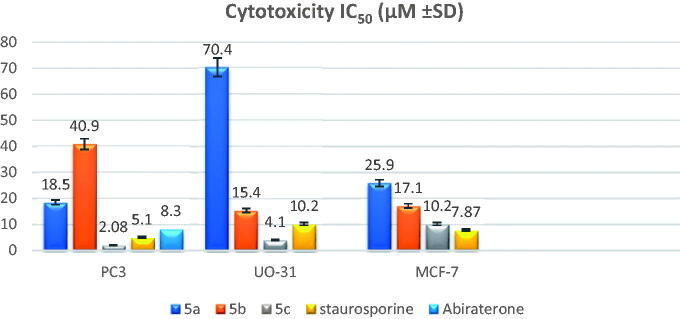
Graphical representation of IC_50_ of the tested compounds **5a-c** compared to staurosporine as a reference standard.

4. Please change figure 4 with the below figure

**Figure 4.**
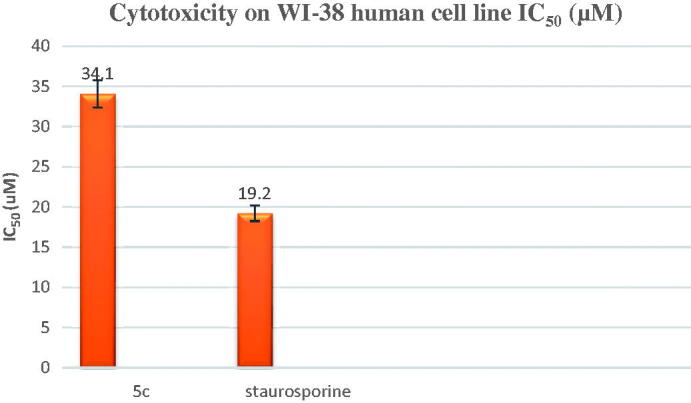
*In-vitro* cytotoxicity (IC_50_) of compound **5c** and staurosporine on WI-38 human cell line.

5. Please change figure 5 with the below figure

**Figure 5.**
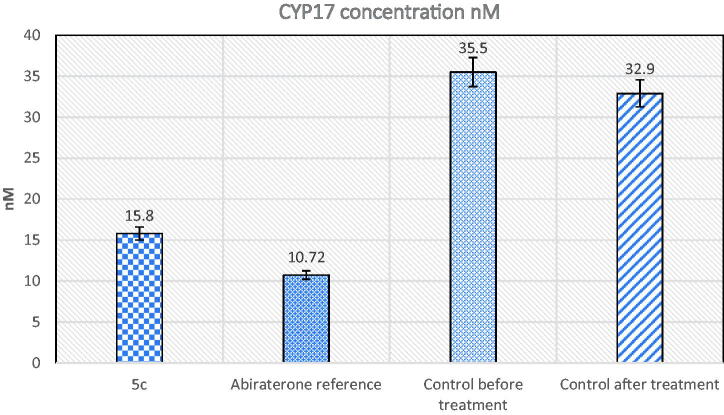
The effect of compound **5c** and abiraterone reference on CYP17 enzyme compared to control.

6. Please change figure 6 with the below figure

**Figure 6.**
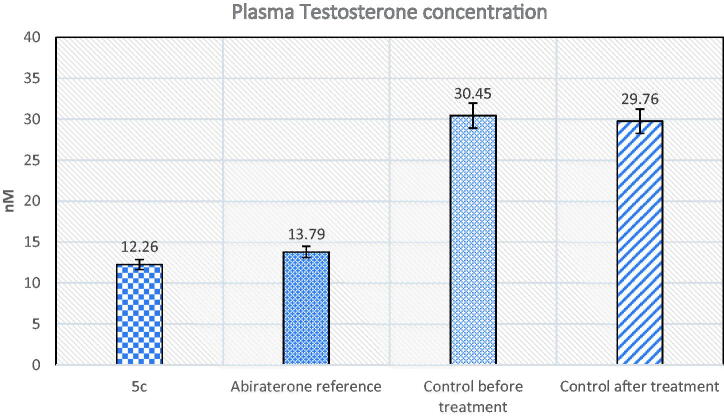
The effect of compound **5c** and abiraterone reference on plasma testosterone.

7. Please change figure 7c with the below figure

**Figure 7.**
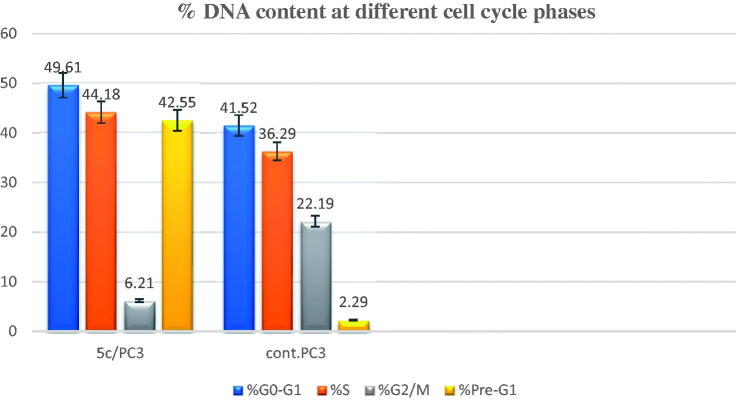
A) Cell cycle analysis of PC-3 treated with DMSO only. (B) Cell cycle analysis of PC-3 after treatment with **5c** (2.34 µM) (C) Graphical representation of effect of compound **5c** on cell cycle profile of PC-3 cells.

8. Please change figure 10 with the below figure

**Figure 10.**
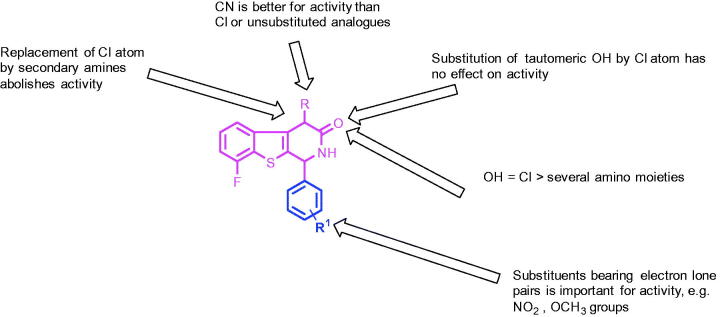
SAR study of the synthesized benzothienopyridine derivatives.

