# Supplementary material for: New benzothieno[2,3-c]pyridines as non-steroidal CYP17 inhibitors: design, synthesis, anticancer screening, apoptosis induction, and in silico ADME profile studies
Source: J Enzyme Inhib Med Chem. 2021 Aug 2;36(1):1839–59. doi: 10.1080/14756366.2021.1958212 (PMC8330742; doi:10.1080/14756366.2021.1958212)
Supplement: Supplemental Material [file IENZ_A_1958212_SM8830.pdf]

2a ^{1}HNMR

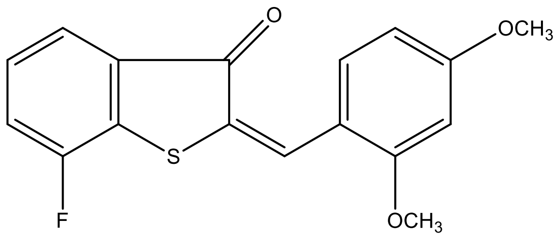

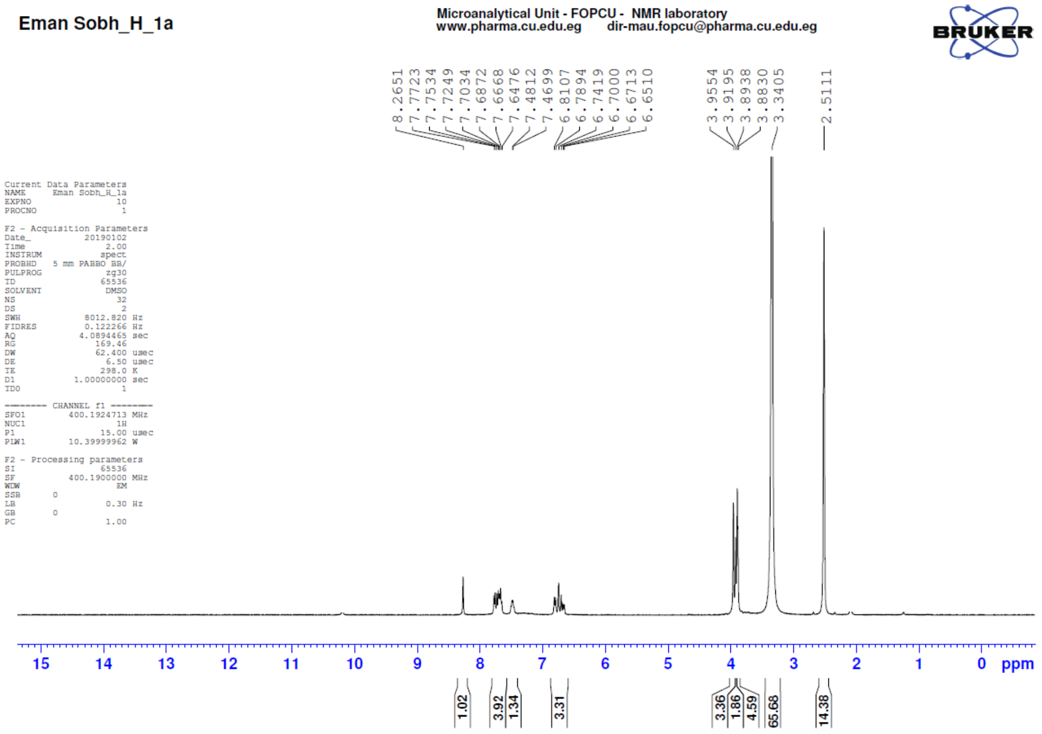

Figure S1: ^{1}HNMR of compound **2a**.

2a ^{13}CNMR

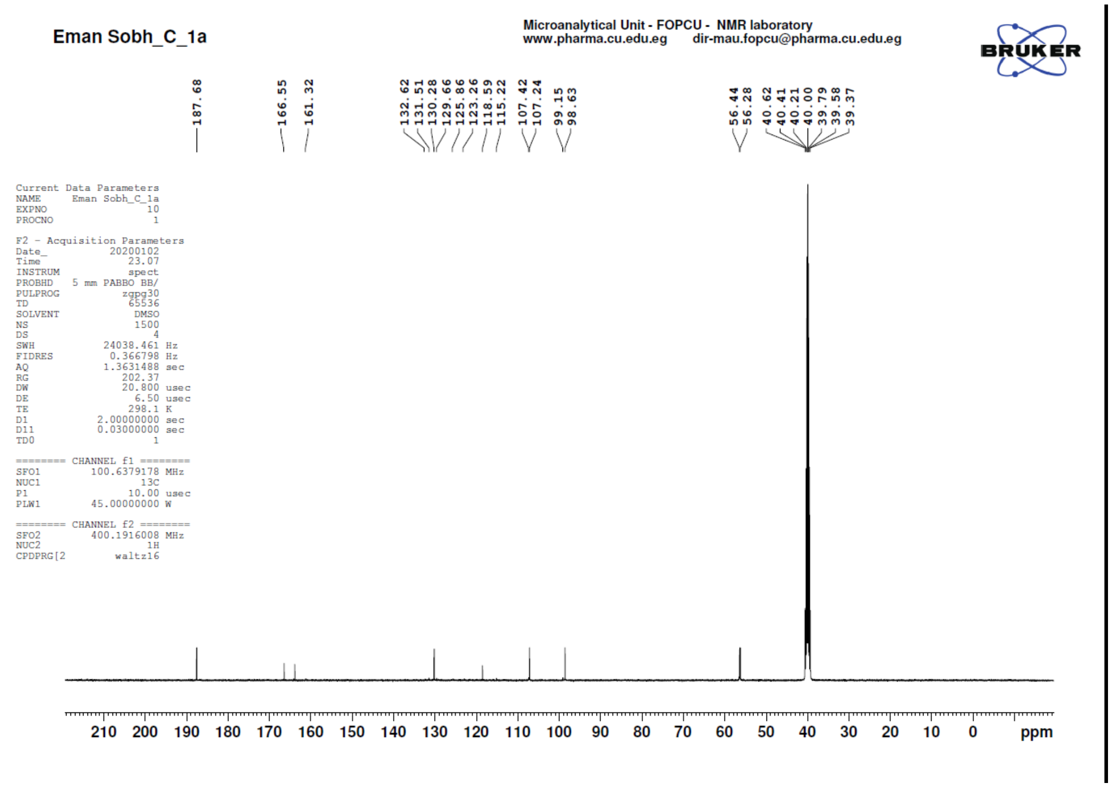

Figure S2: ^{13}CNMR of compound **2a**.

2c ^{1}HNMR

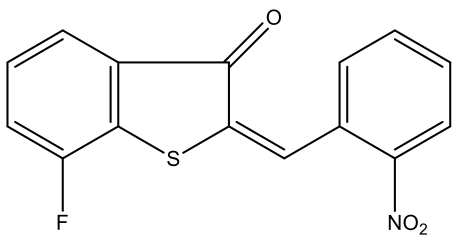

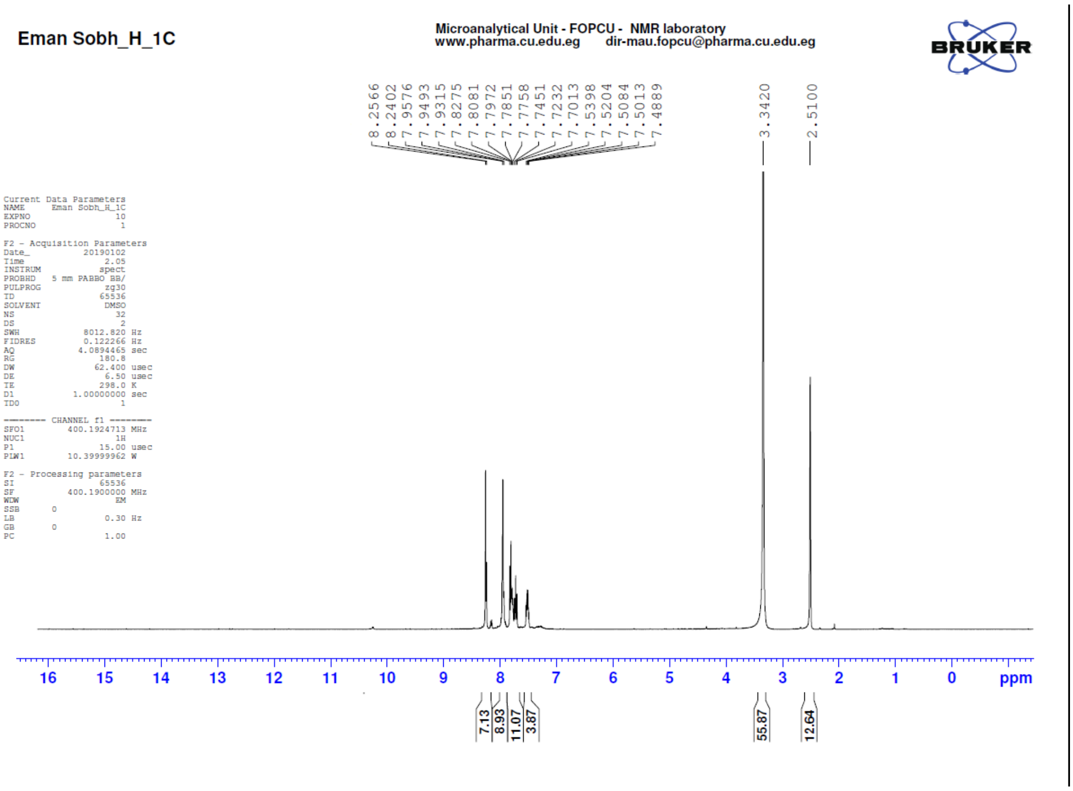

Figure S3: ^{1}HNMR of compound **2c**.

2c ^{13}CNMR

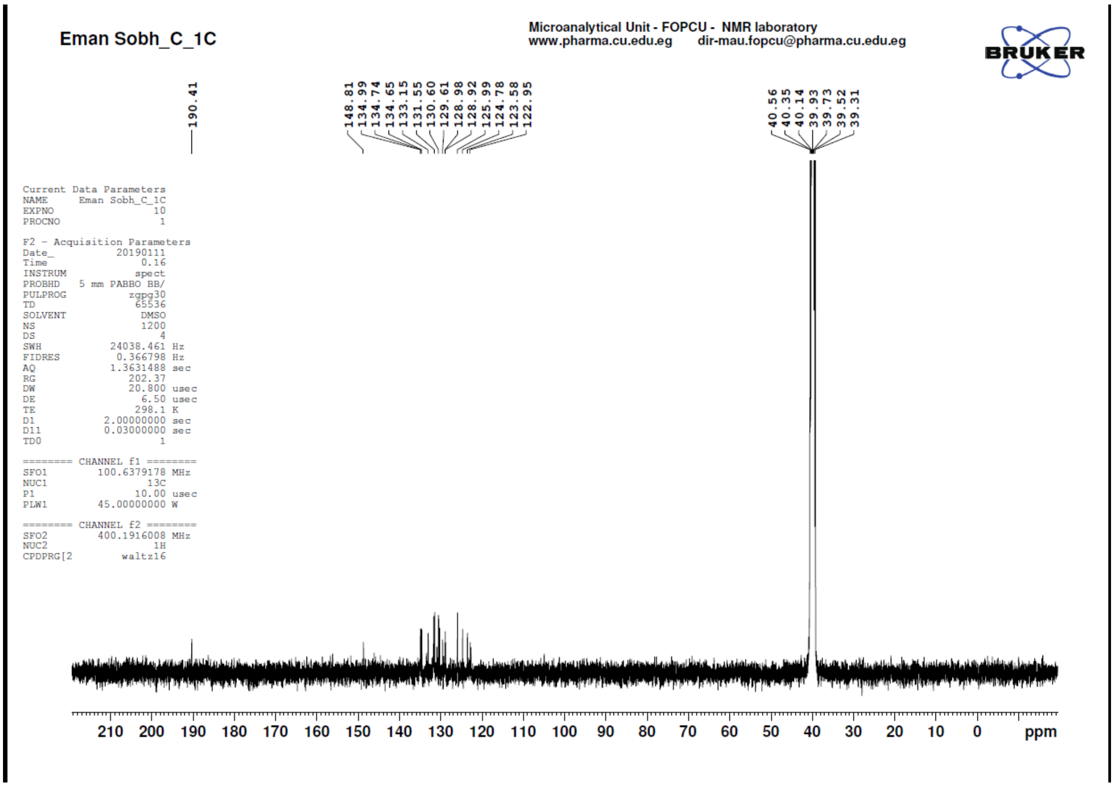

Figure S4: ^{13}CNMR of compound **2c**.

2d ^{1}HNMR

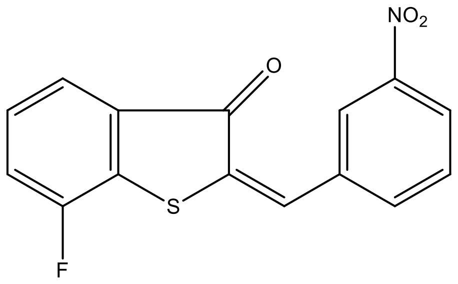

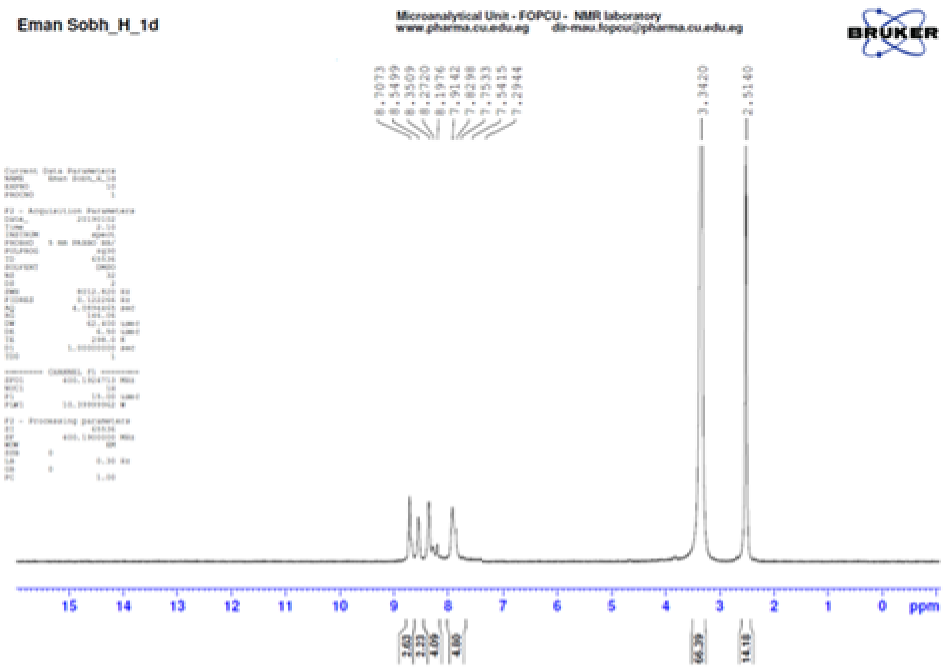

Figure S5: ^{1}HNMR of compound **2d**.

2d ^{13}CNMR

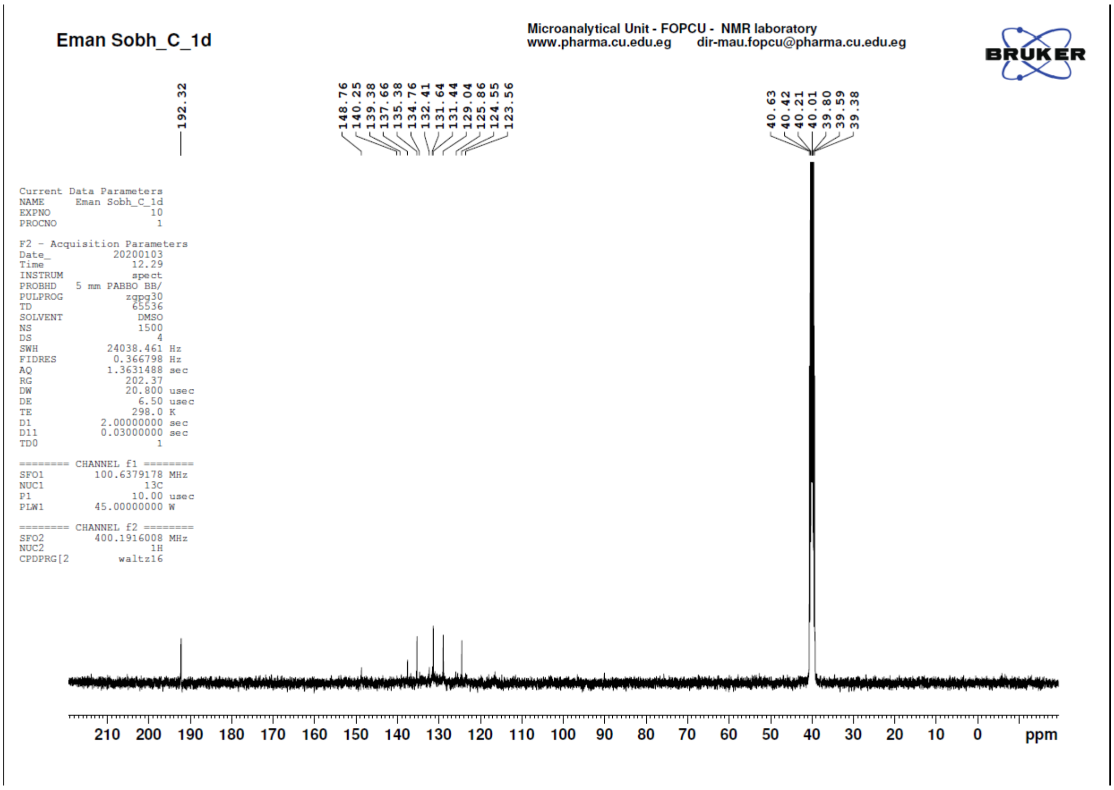

Figure S6: ^{13}CNMR of compound **2d**.

3a ^{1}HNMR

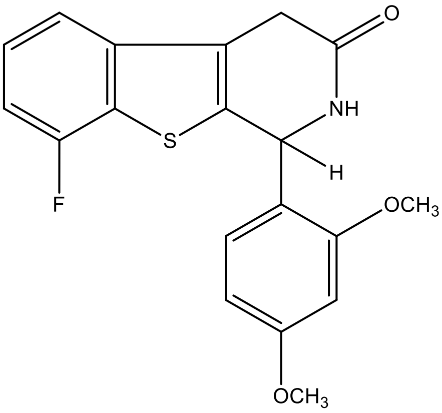

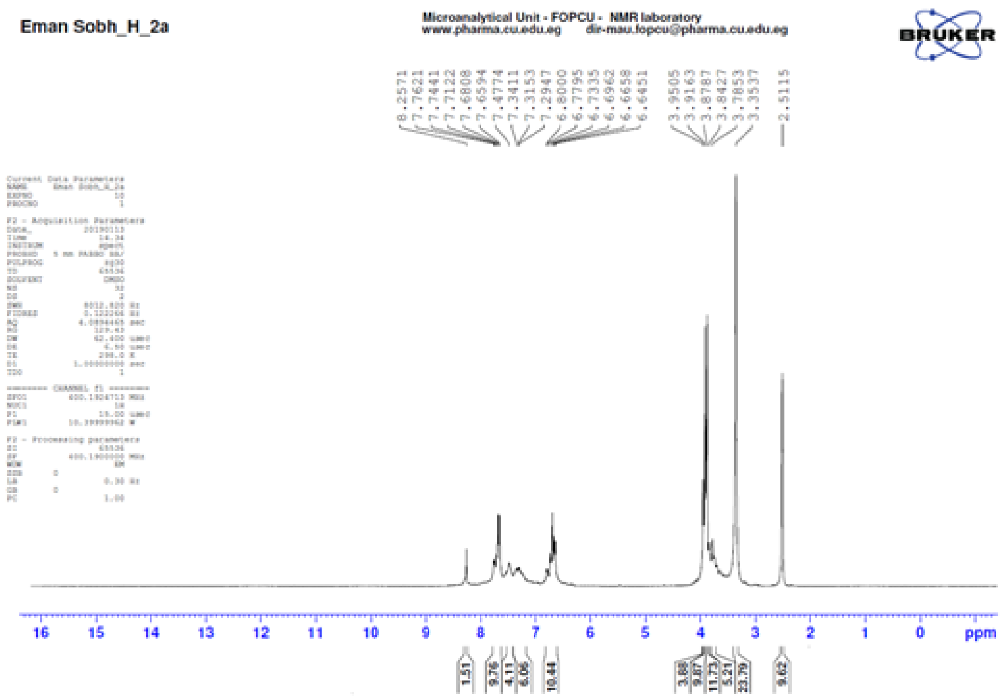

Figure S7: ^{1}HNMR of compound **3a**.

3a^{13}CNMR

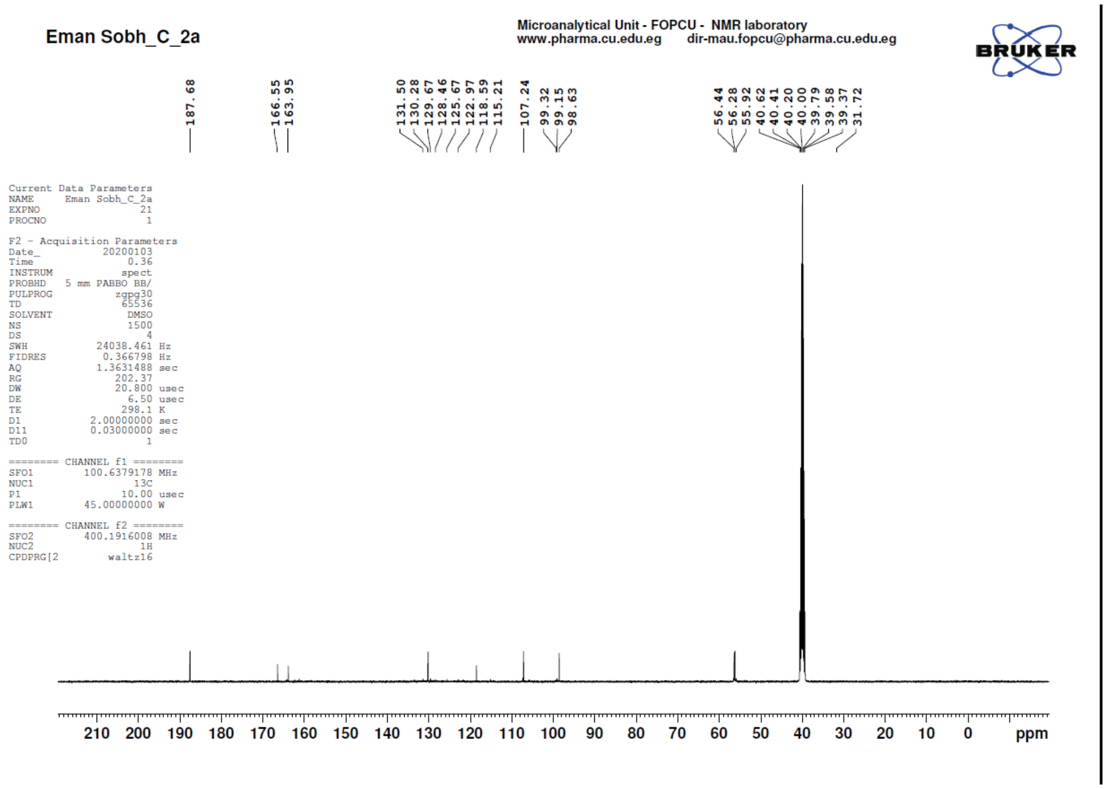

Figure S8: ^{13}CNMR of compound **3a**.

3b ^{1}HNMR

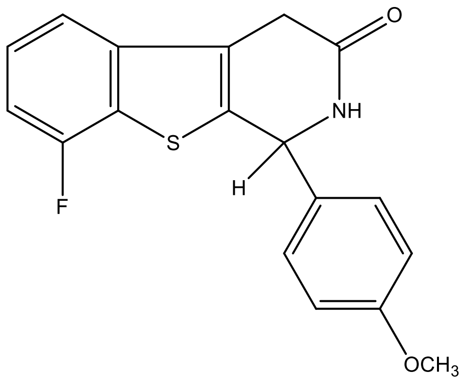

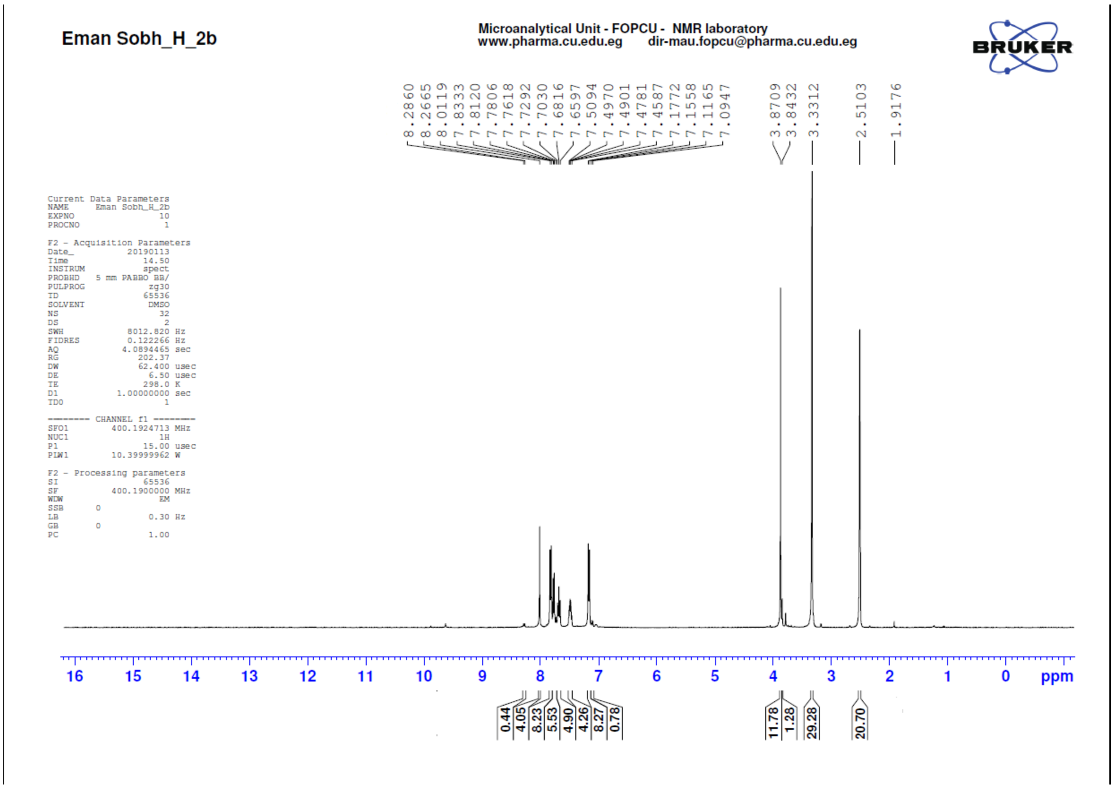

Figure S9: ^{1}HNMR of compound **3b**.

3b ^{13}CNMR

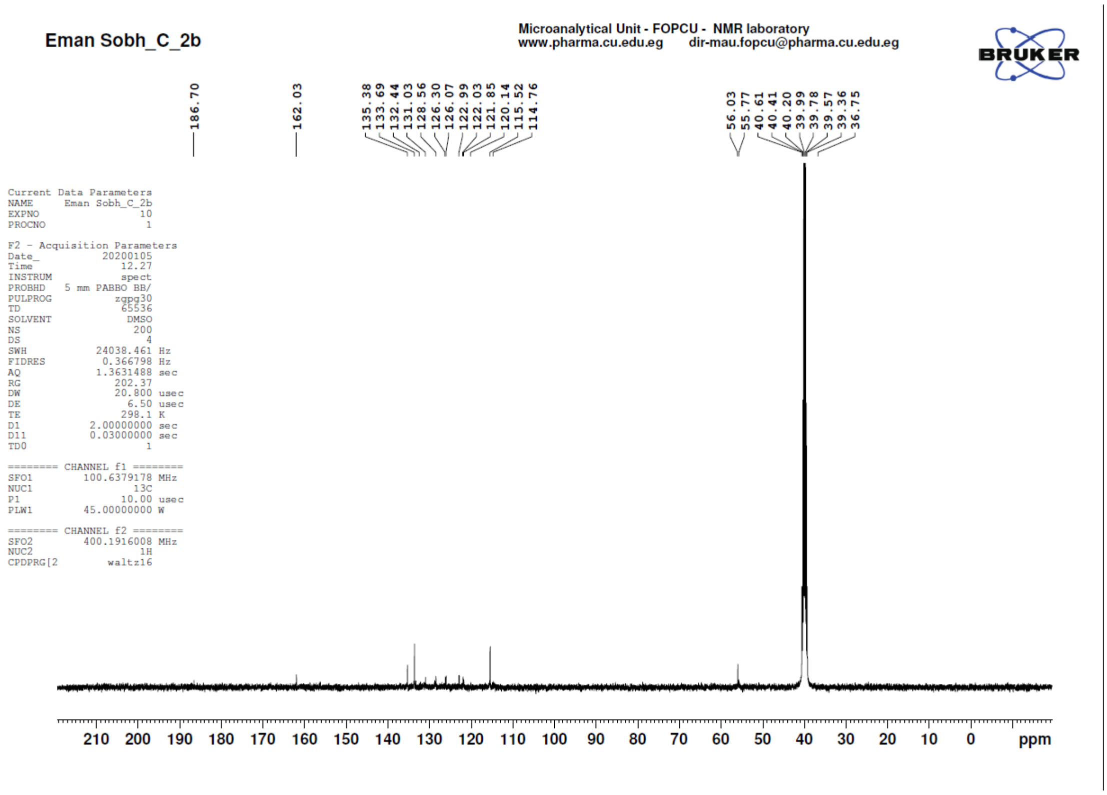

Figure S10: ^{13}CNMR of compound **3b**.

3c ^{1}HNMR

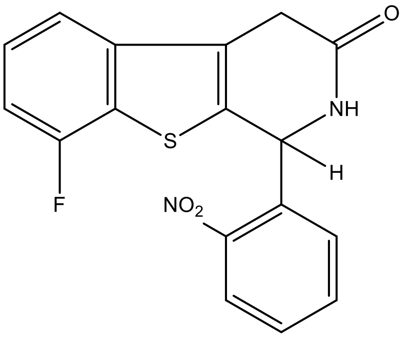

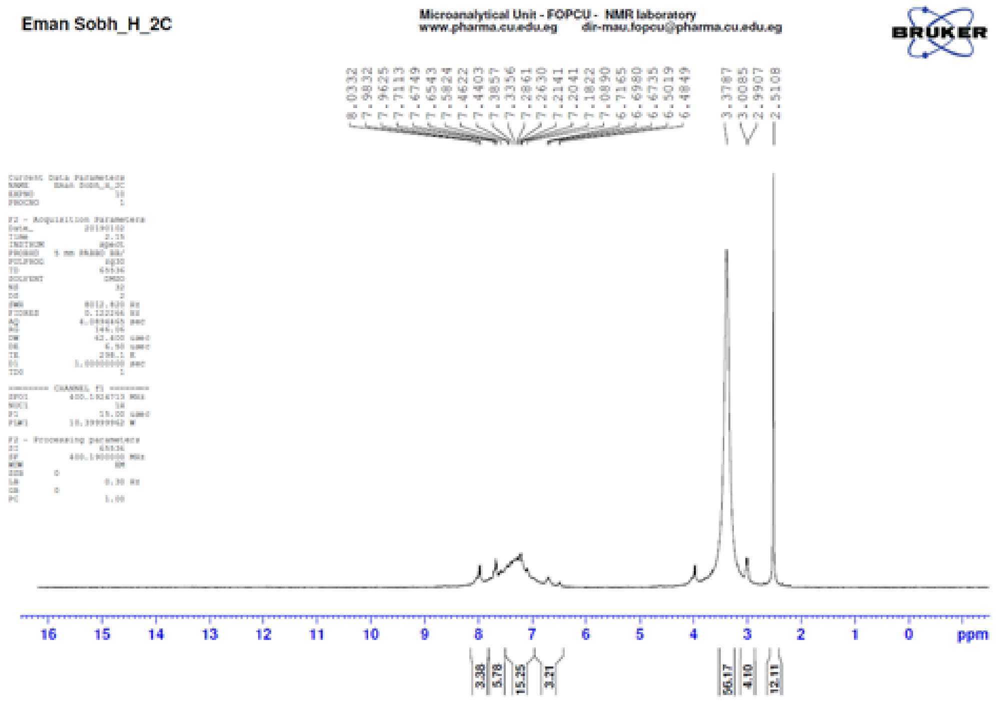

Figure S11: ^{1}HNMR of compound **3c**.

3c ^{13}CNMR

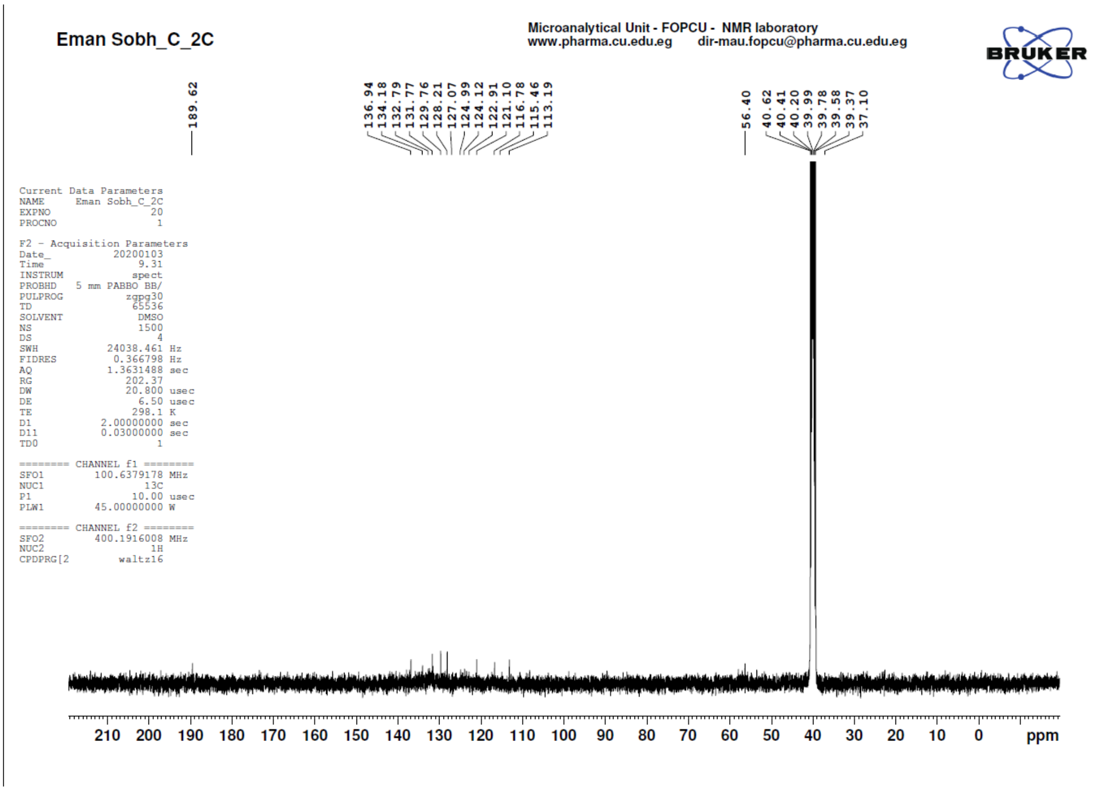

Figure S12: ^{13}CNMR of compound **3c**.

3d ^{1}HNMR

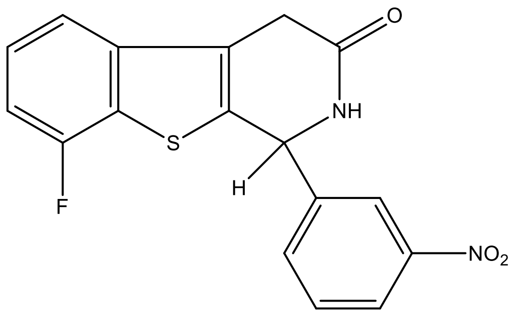

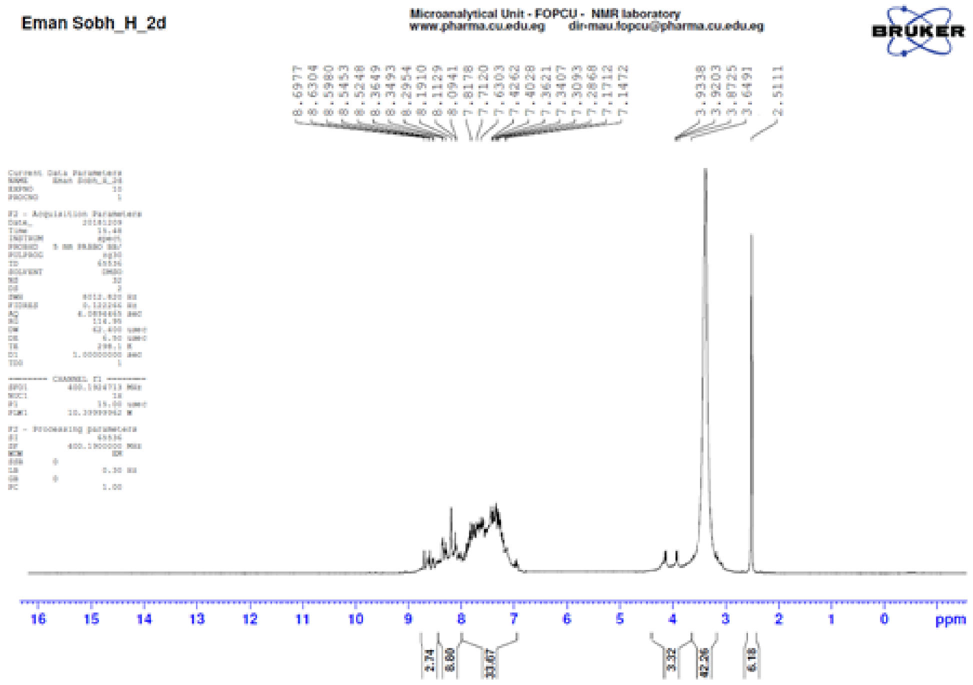

Figure S13: ^{1}HNMR of compound **3d**.

3d ^{13}CNMR

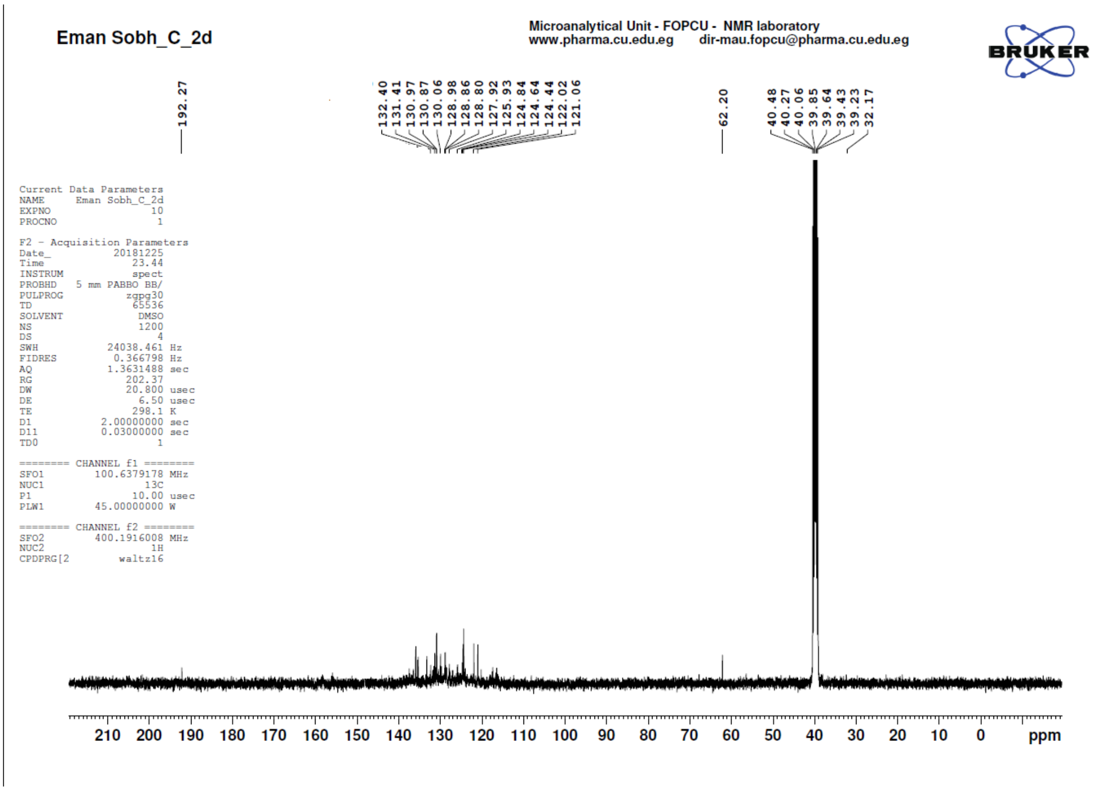

Figure S14: ^{13}CNMR of compound **3d**.

4a^{1}HNMR

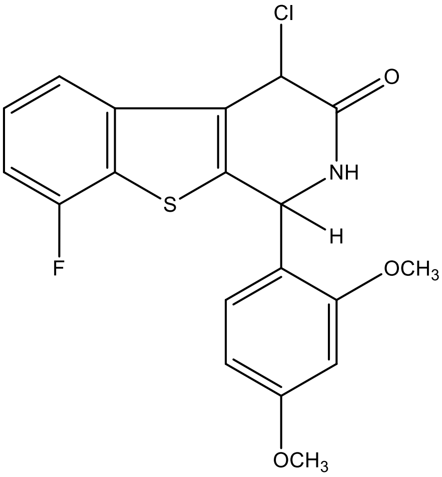

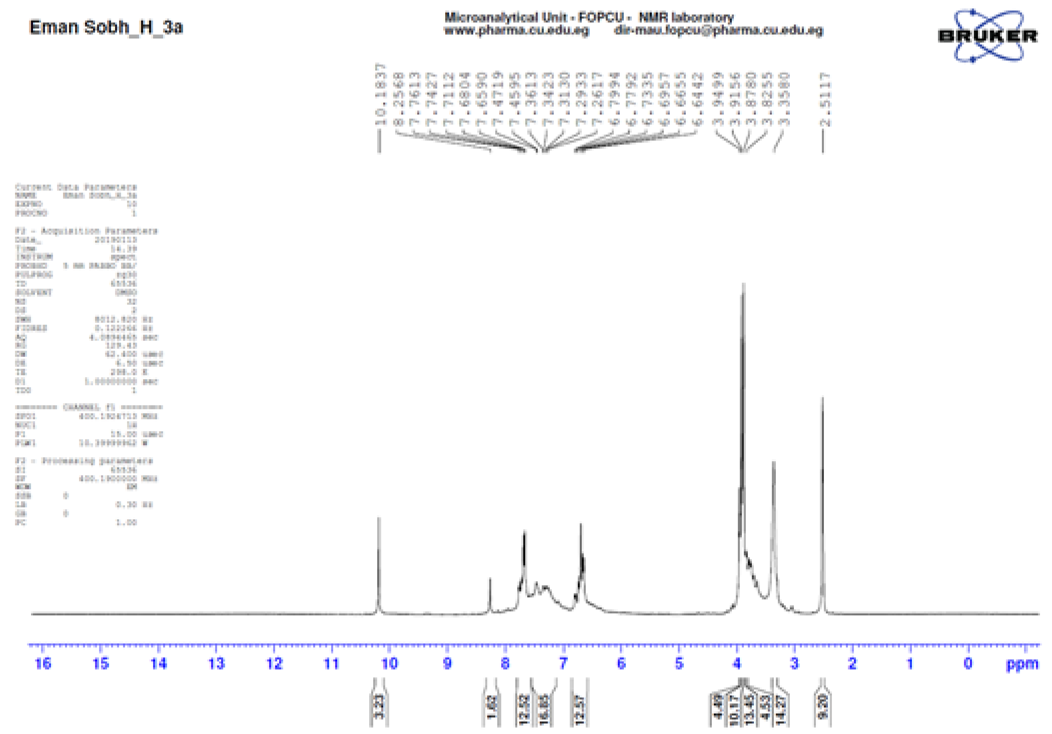

Figure S15: ^{1}HNMR of compound **4a**.

4a ^{13}CNMR

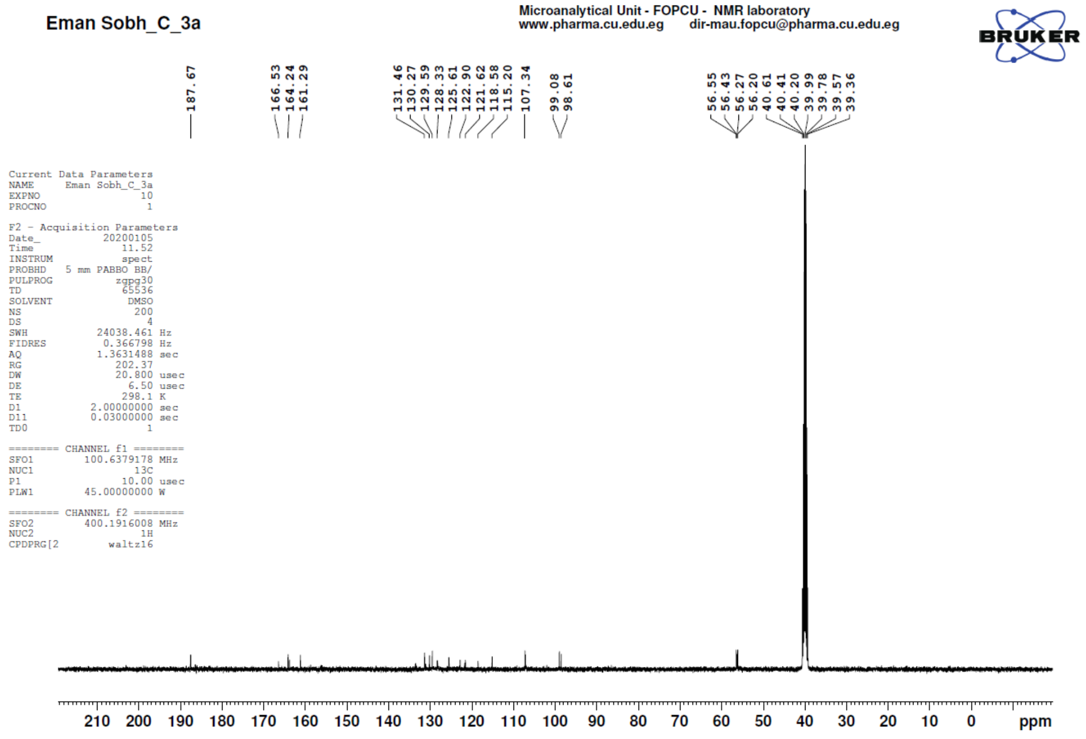

Figure S16: ^{13}CNMR of compound **4a**.

4b ^{1}HNMR

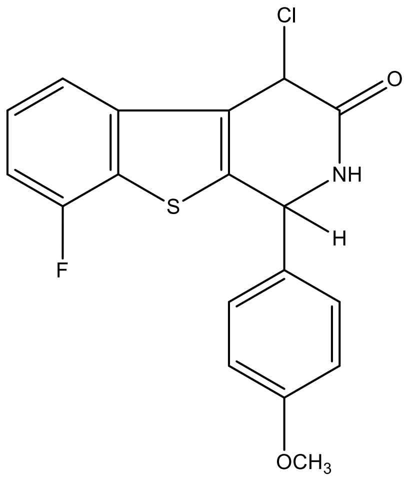

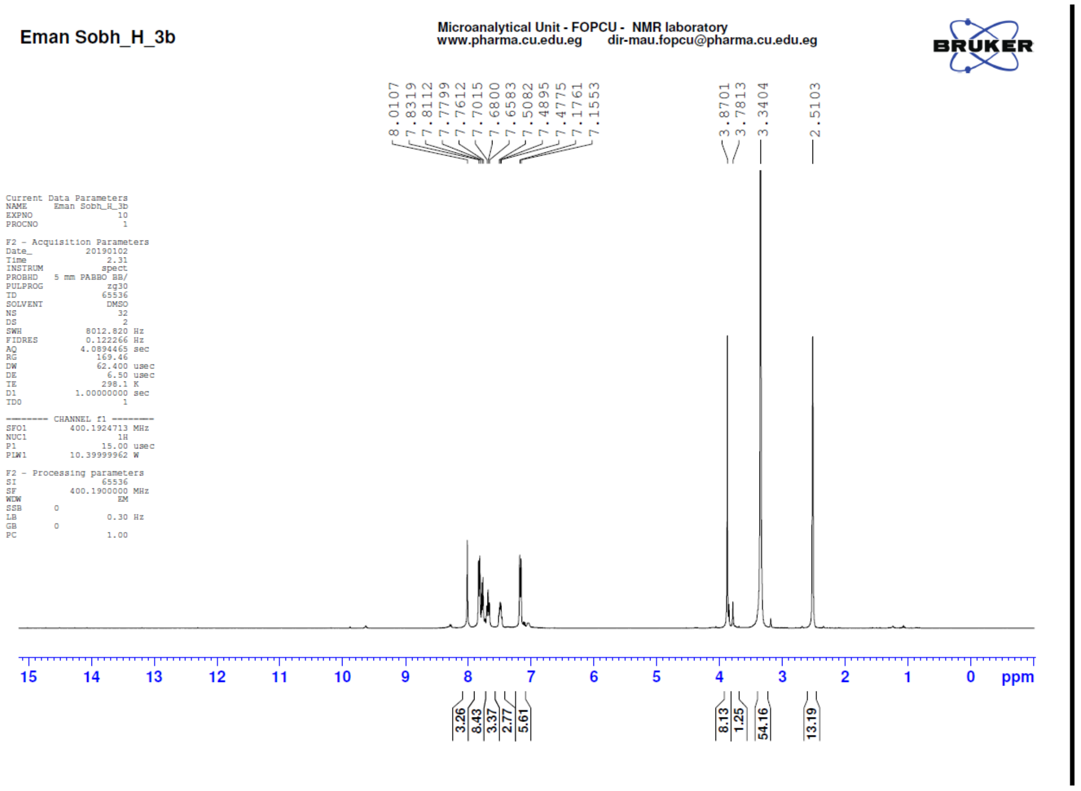

Figure S17: ^{1}HNMR of compound **4b**.

4b ^{13}CNMR

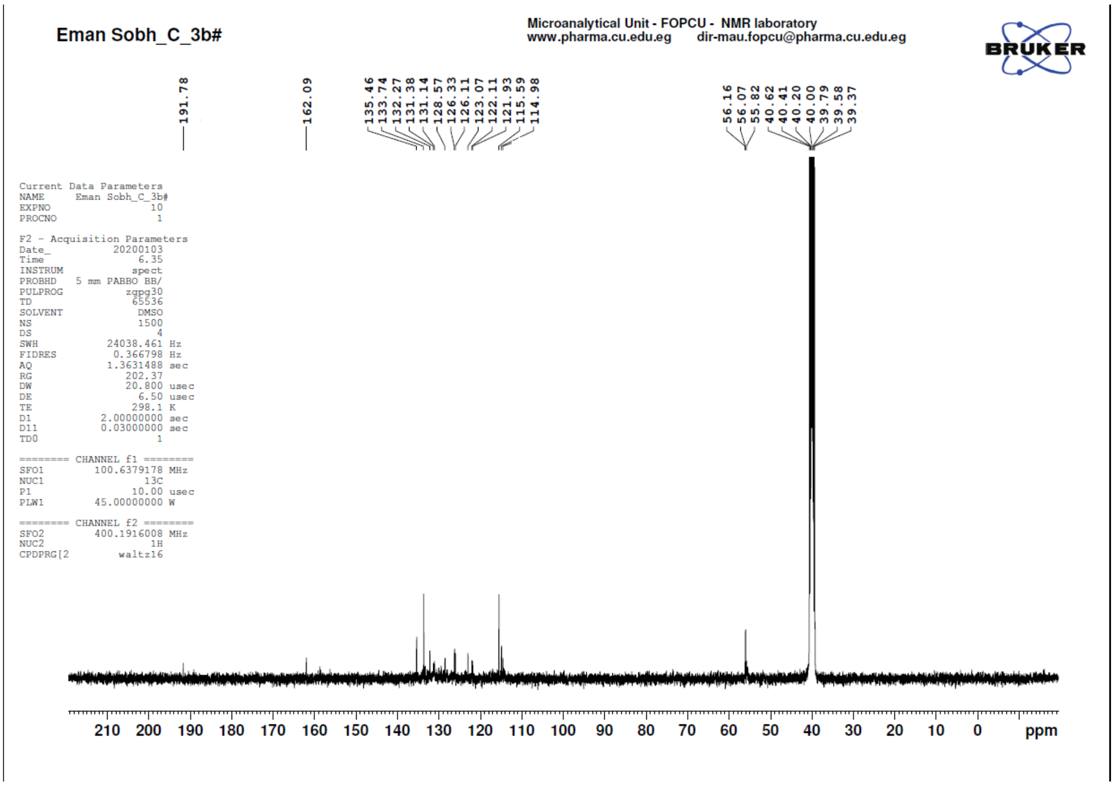

Figure S18: ^{13}CNMR of compound **4b**.

4c ^{1}HNMR

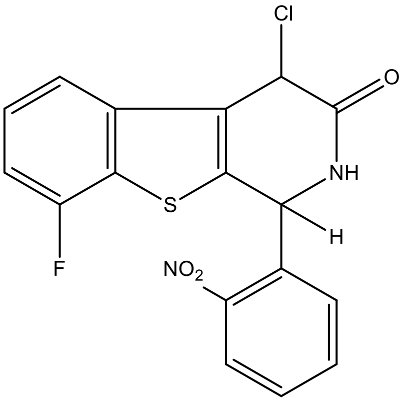

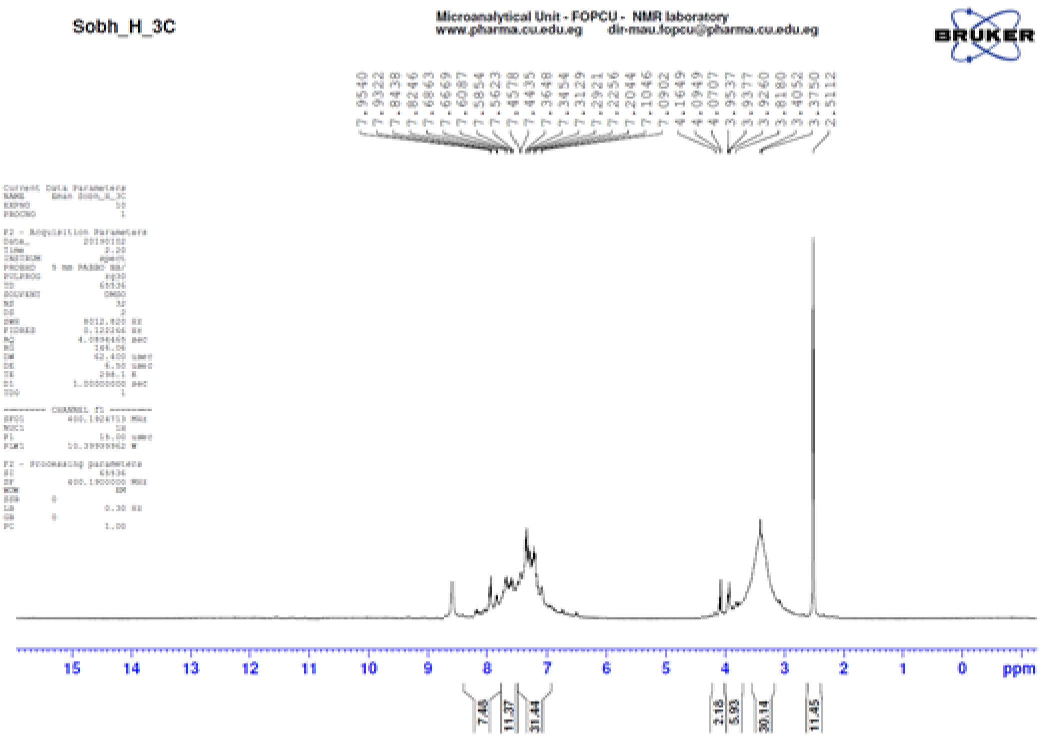

Figure S19: ^{1}HNMR of compound **4c**

4d ^{1}HNMR

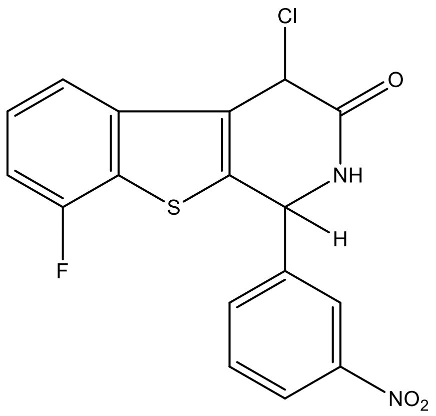

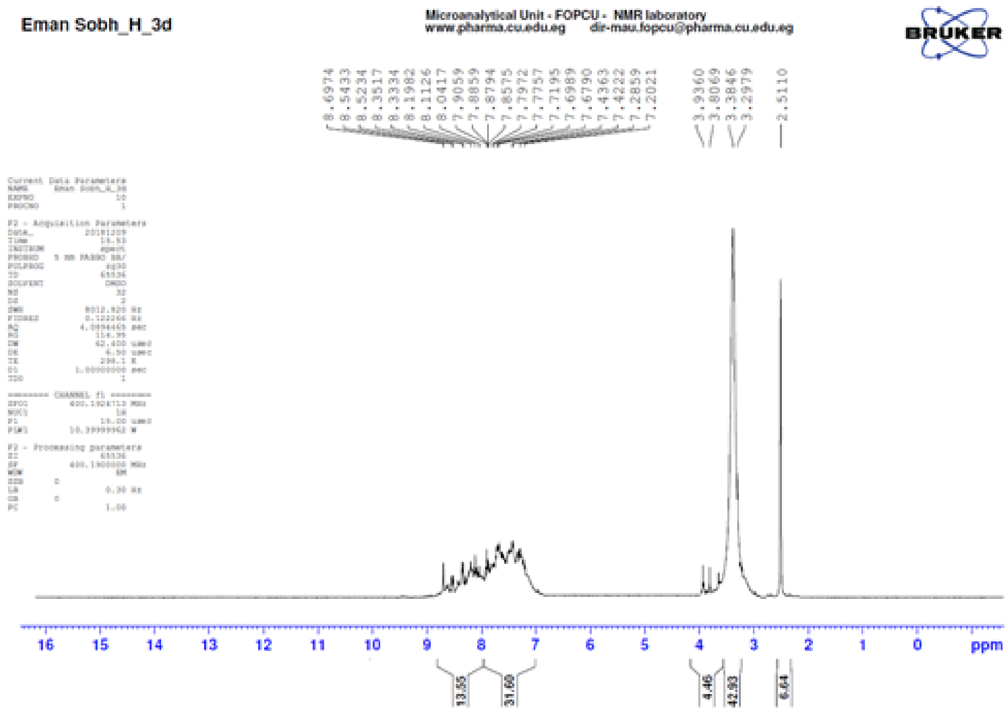

Figure S20: ^{1}HNMR of compound **4d.**

4d ^{13}CNMR

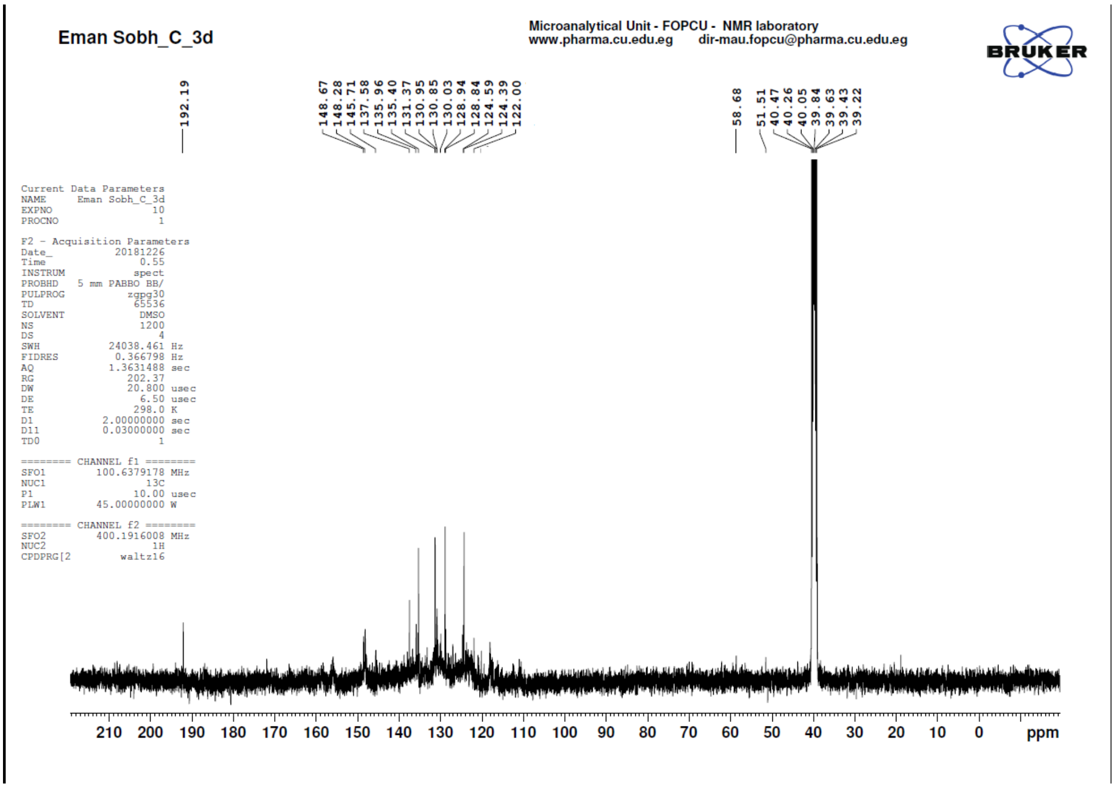

Figure S21: ^{13}CNMR of compound **5a.**

5a ^{1}HNMR

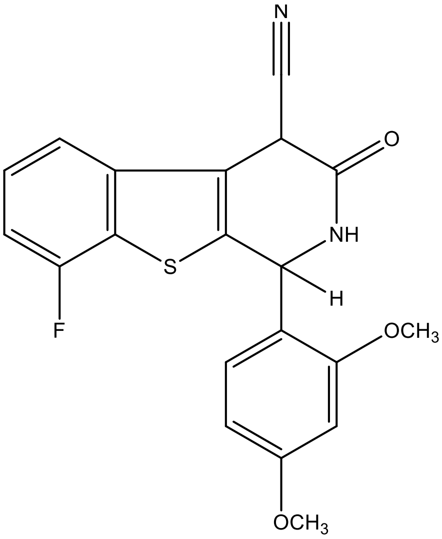

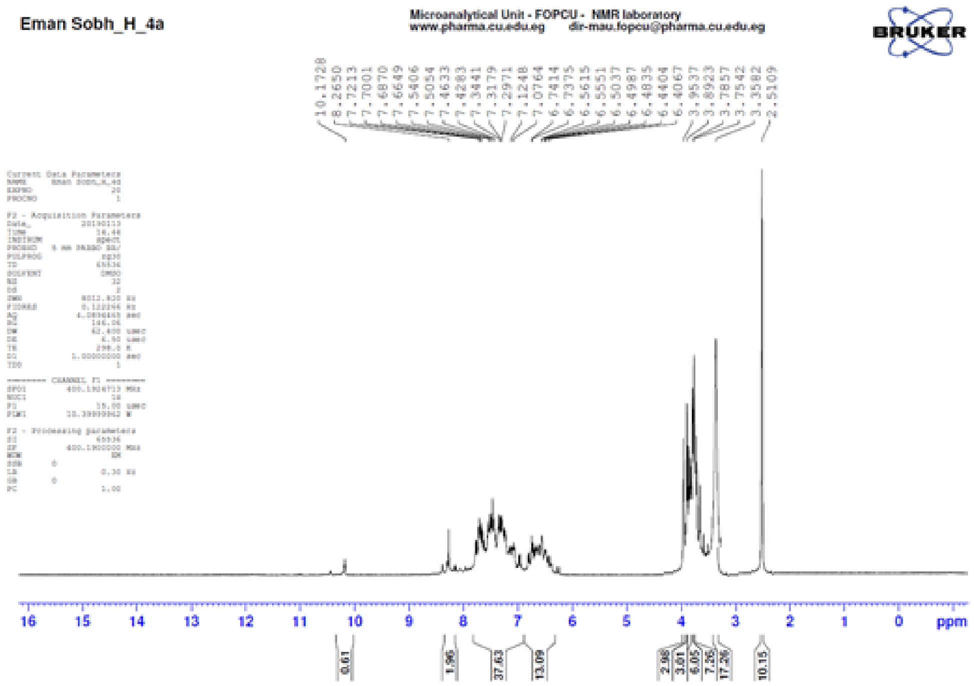

Figure S22: ^{1}HNMR of compound **5a.**

5b ^{1}HNMR

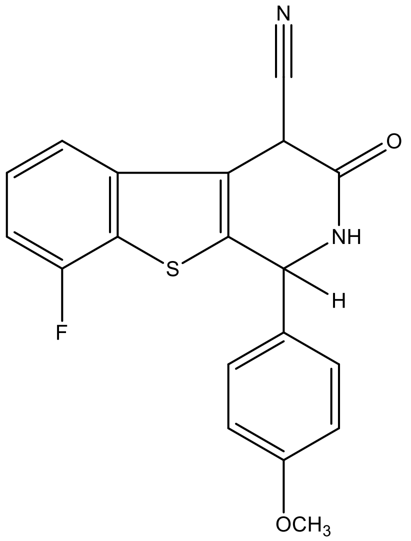

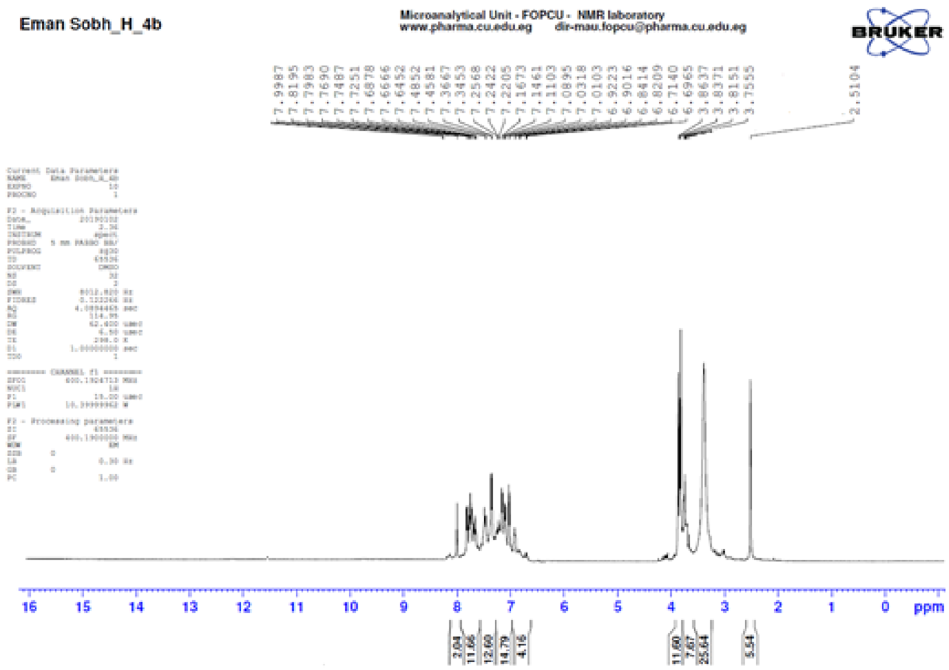

Figure S23: ^{13}CNMR of compound **5b.**

5b ^{13}CNMR

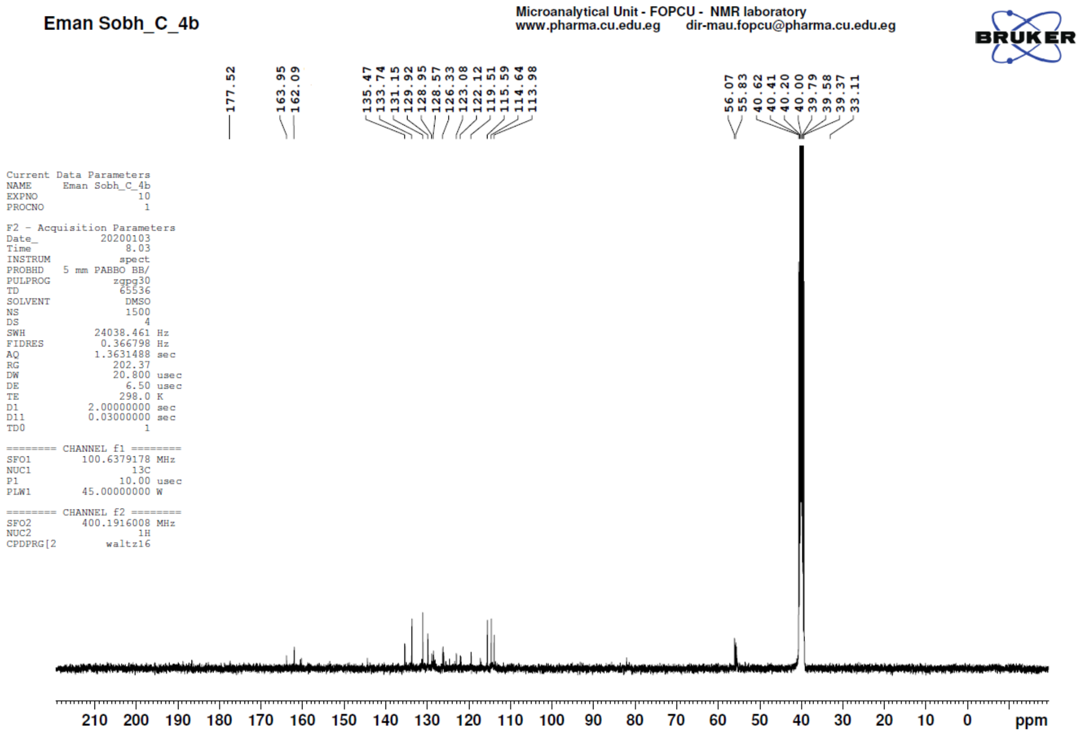

Figure S24: ^{13}CNMR of compound **5c.**

5c ^{1}HNMR

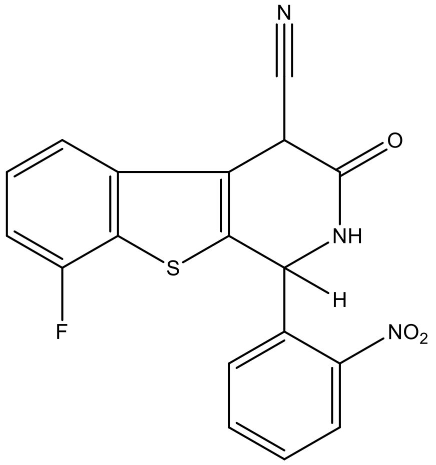

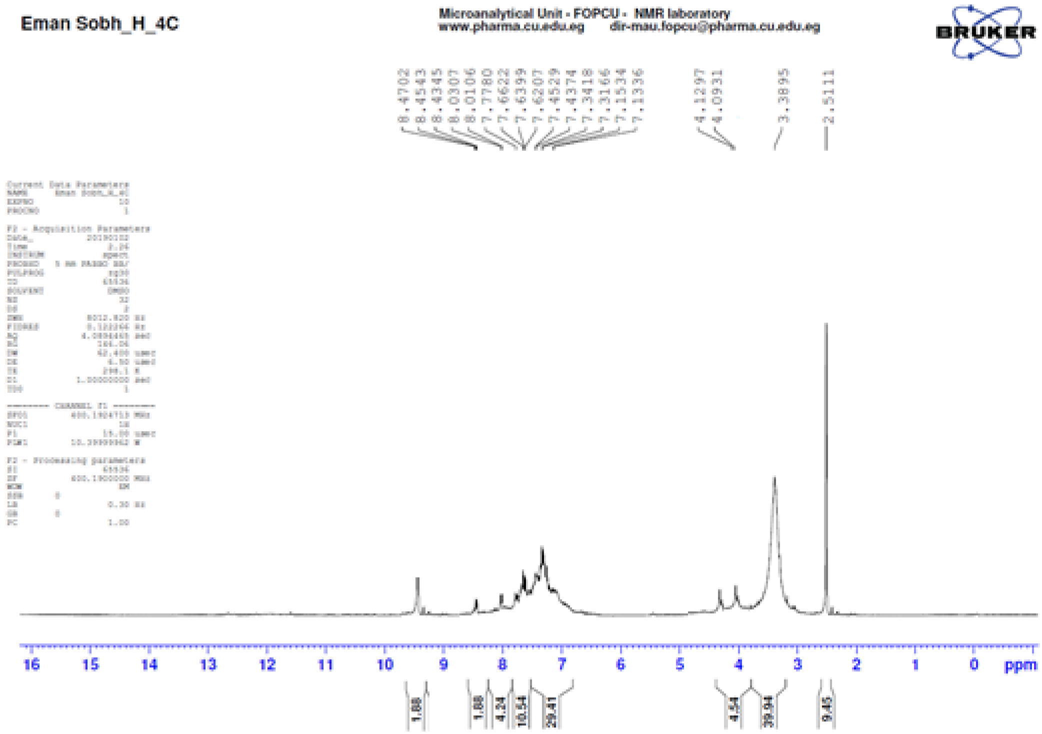

Figure S25: ^{13}CNMR of compound **5c.**

5d ^{1}HNMR

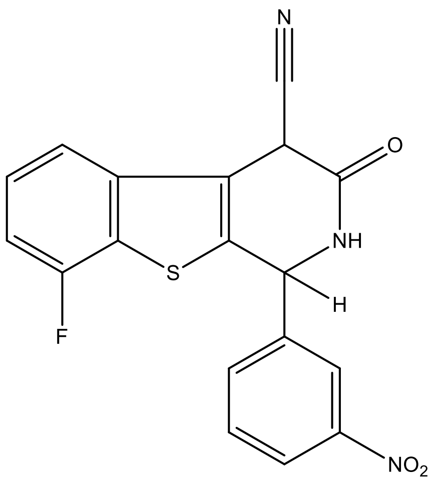

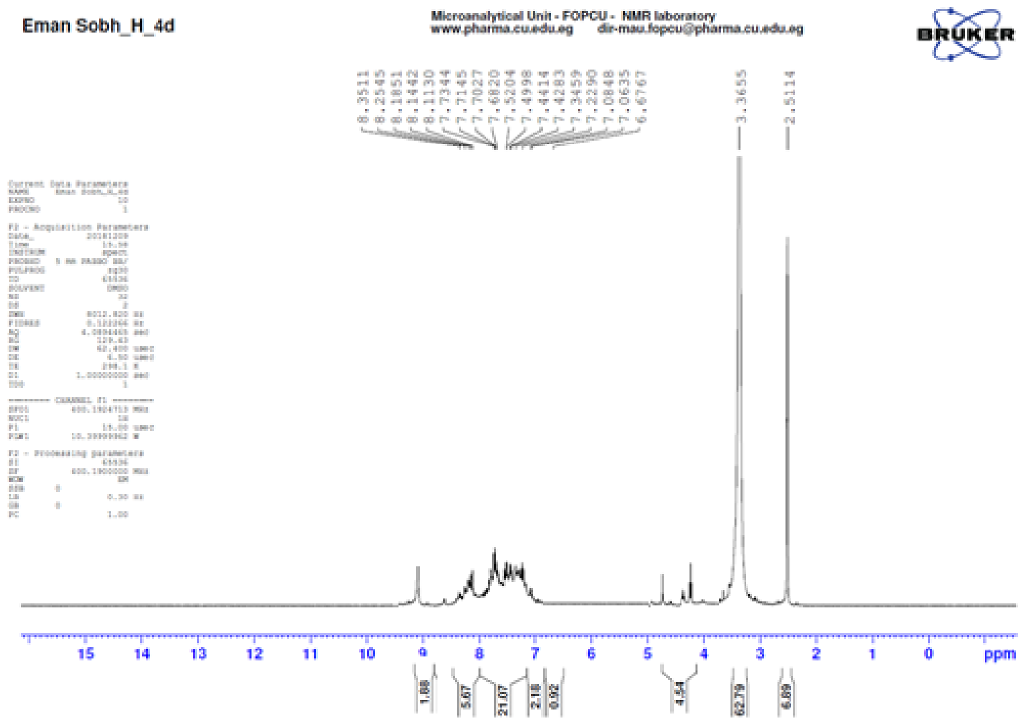

Figure S26: ^{1}HNMR of compound **5d.**

5d ^{13}CNMR

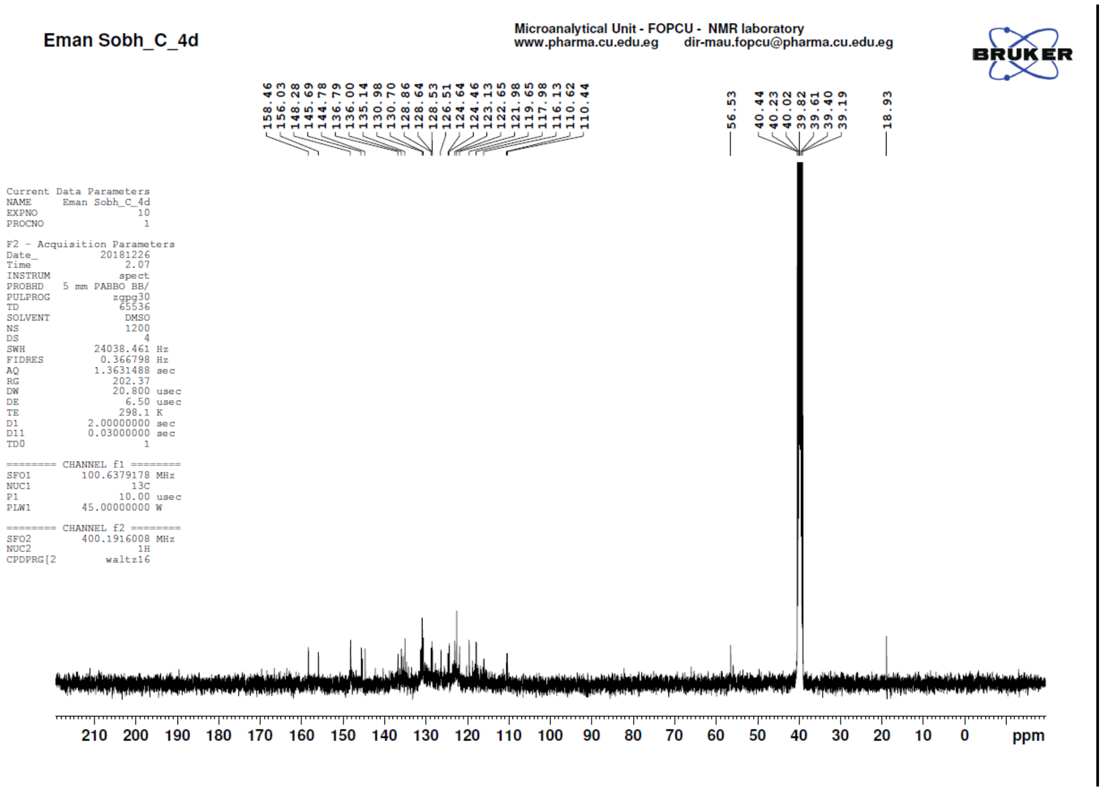

Figure S27: ^{13}CNMR of compound **5d.**

6a ^{1}HNMR

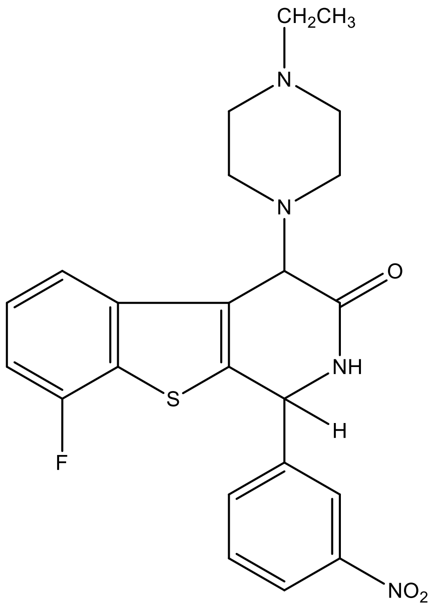

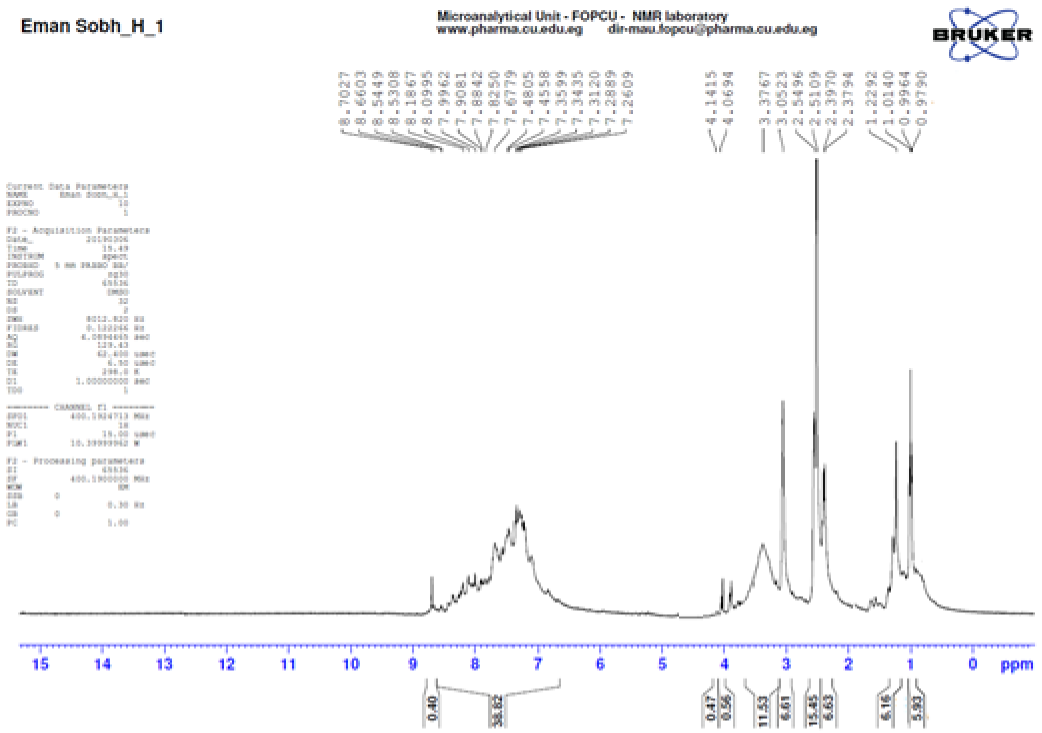

Figure S28: ^{1}HNMR of compound **6a.**

6b ^{1}HNMR

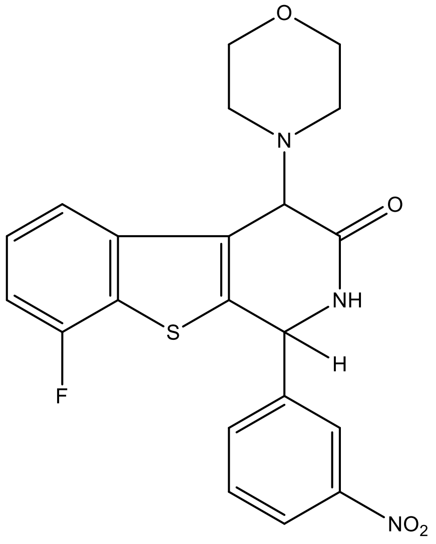

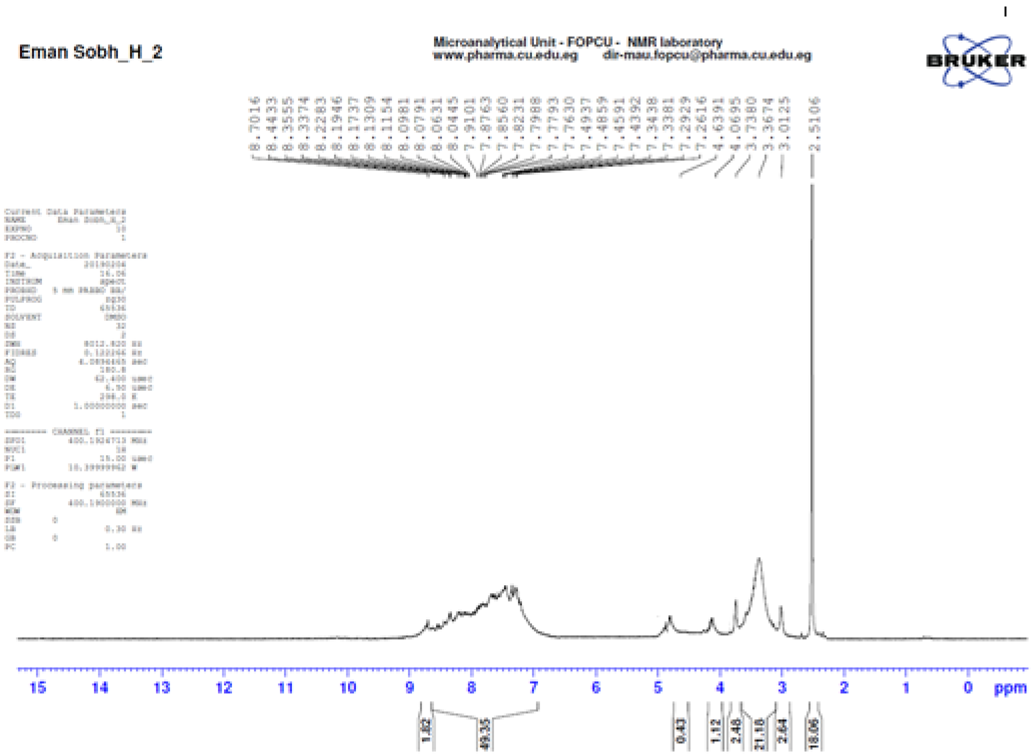

Figure S29: ^{1}HNMR of compound **6b.**

6c ^{1}HNMR

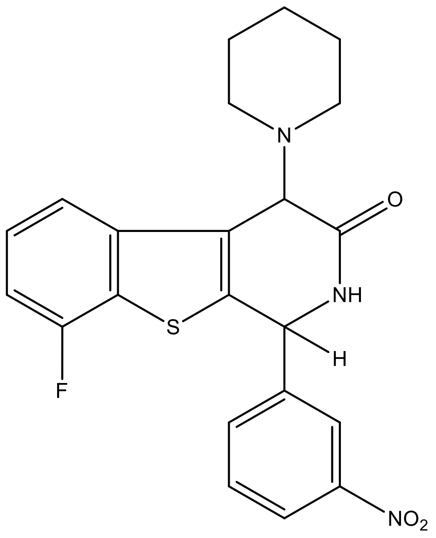

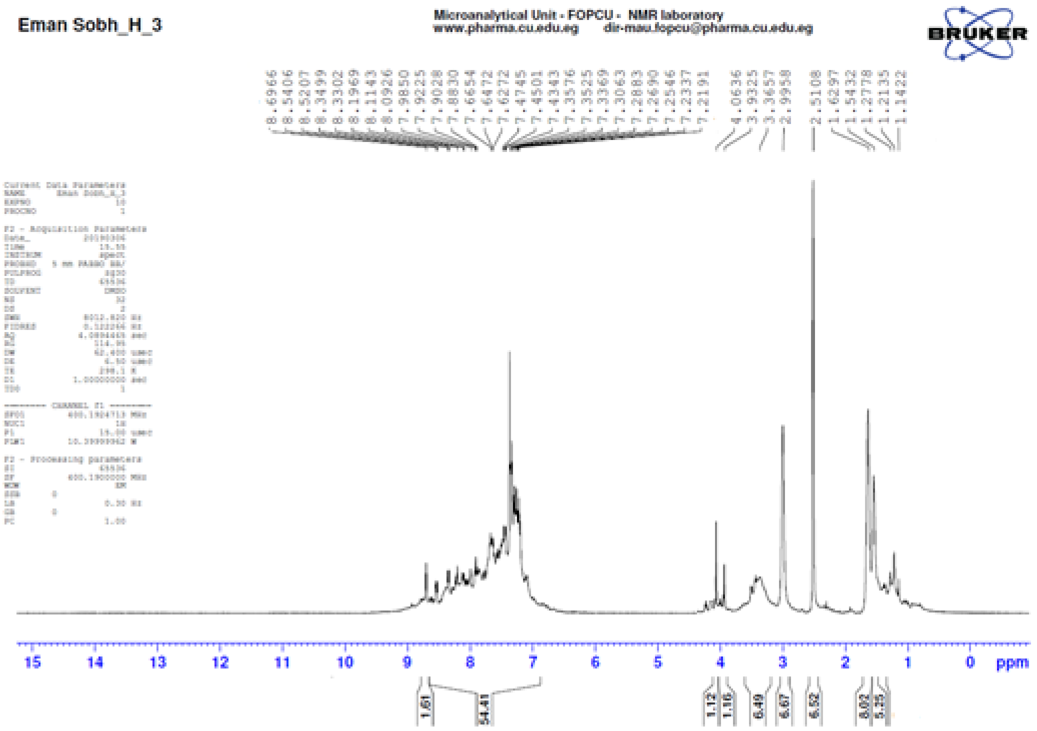

Figure S30: ^{1}HNMR of compound **6c.**

6c ^{13}CNMR

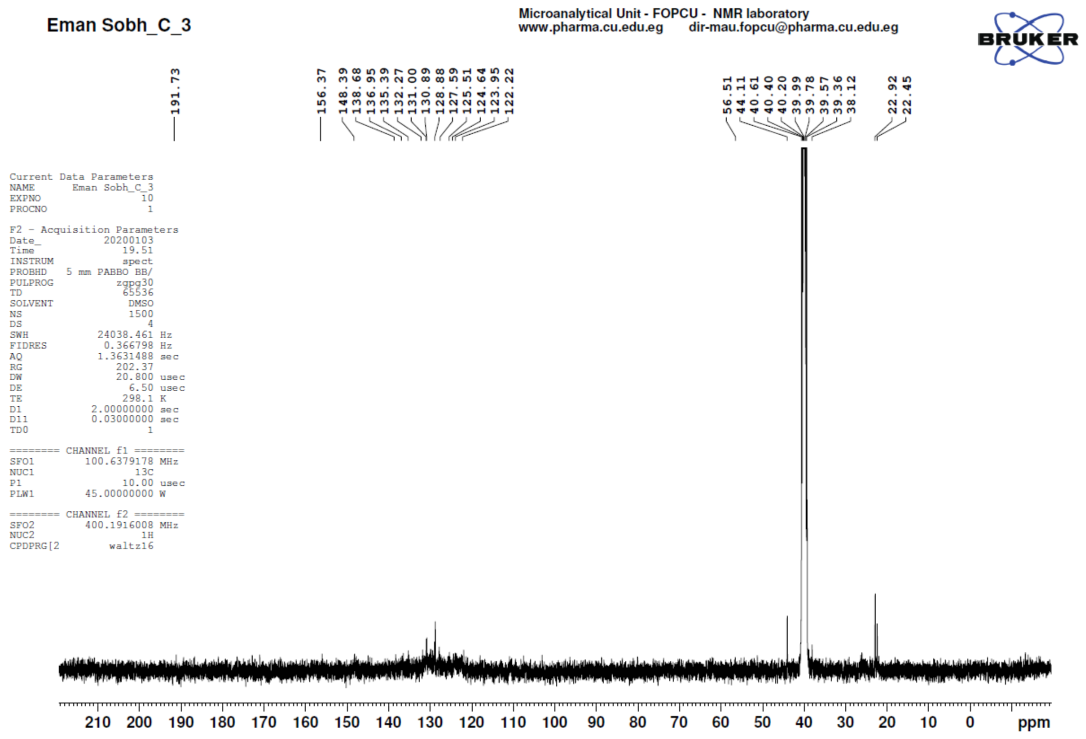

Figure S31: ^{13}CNMR of compound **6c.**

7a ^{1}HNMR

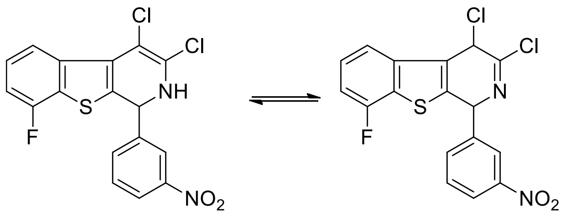

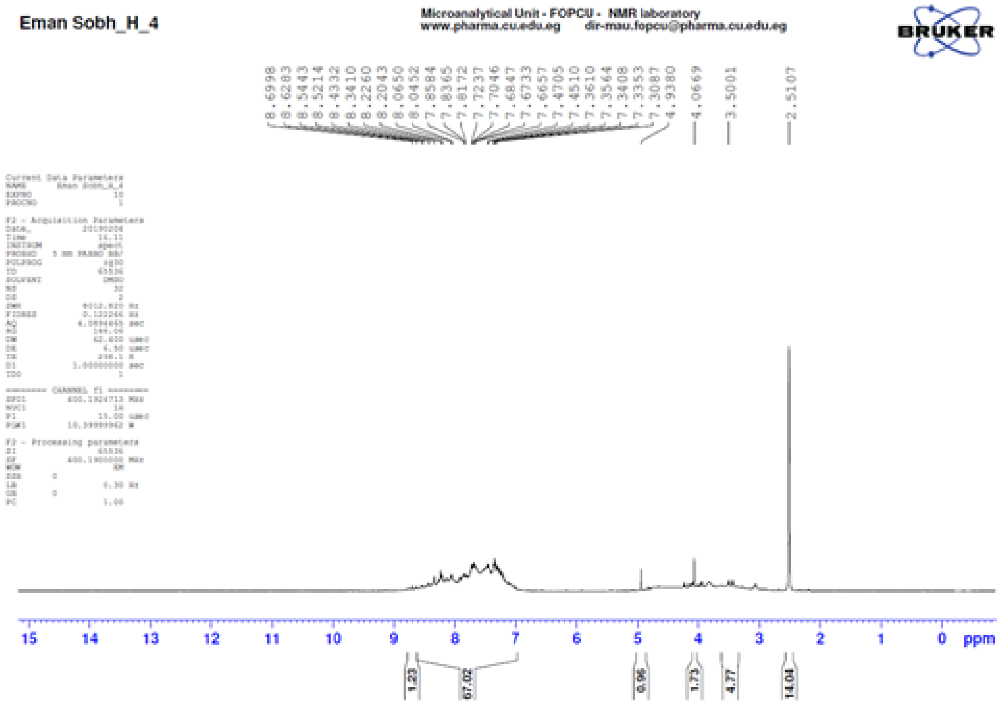

Figure S32: ^{1}HNMR of compound **7a.**

7b ^{1}HNMR

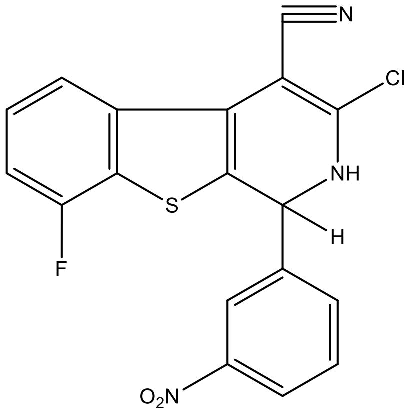

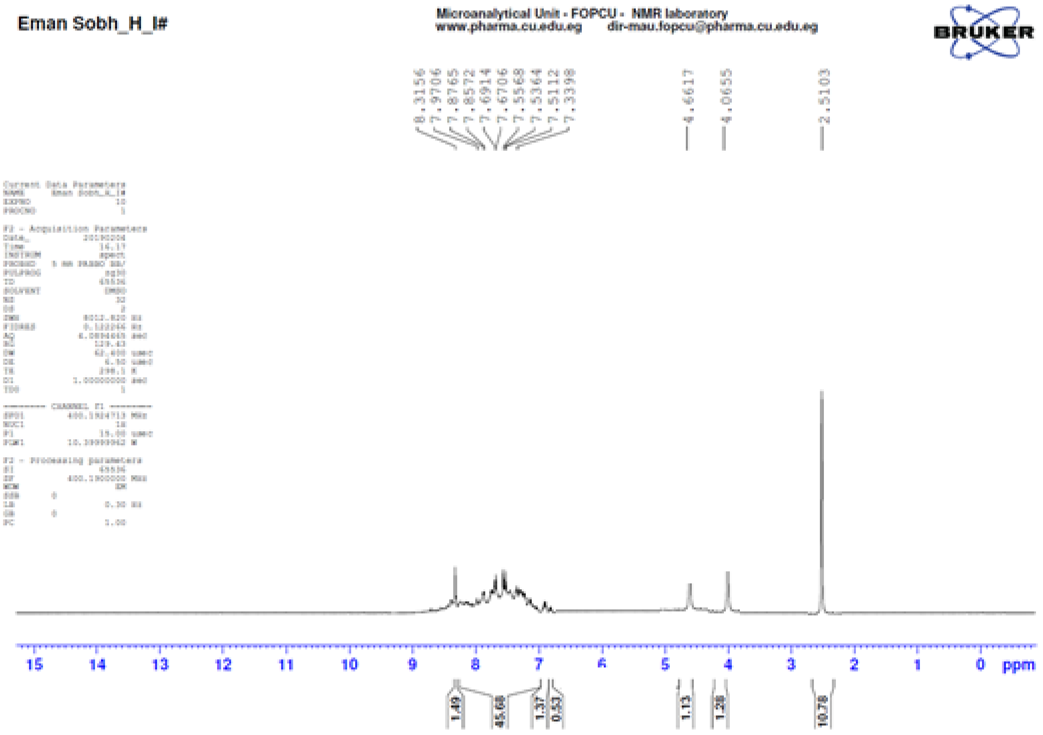

Figure S33: ^{1}HNMR of compound **7b.**

7b ^{13}CNMR

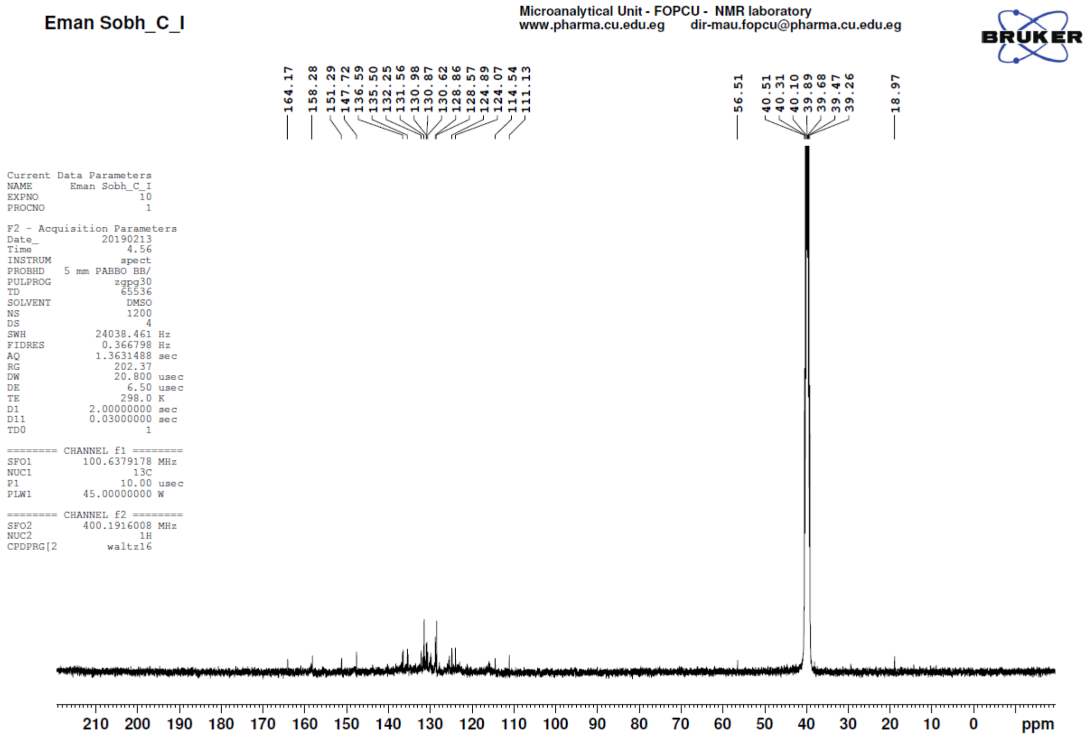

Figure S34: ^{13}CNMR of compound **7b.**

8a ^{1}HNMR

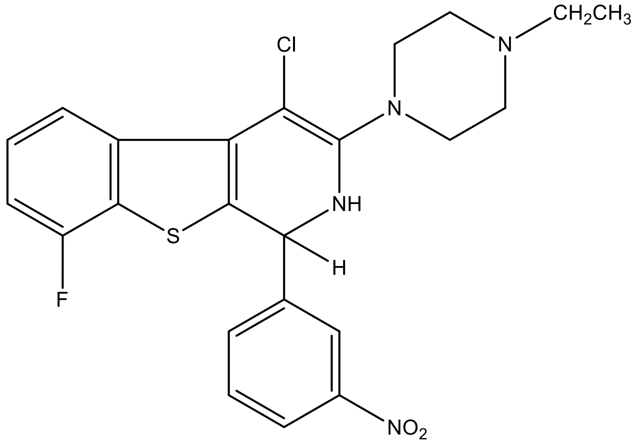

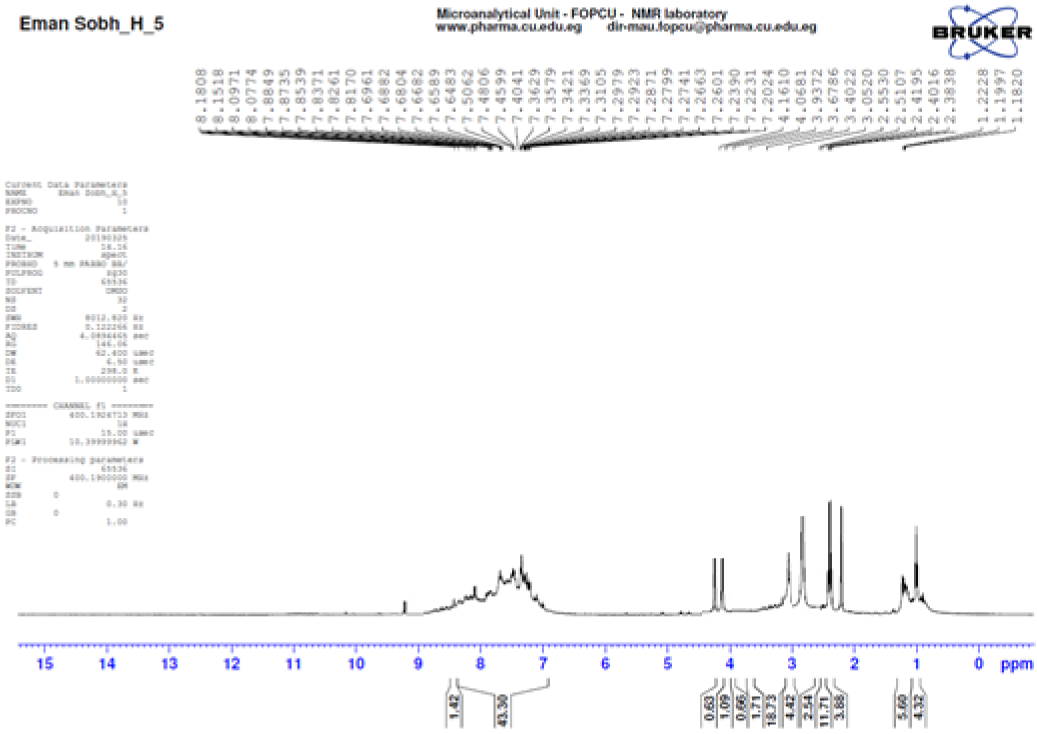

Figure S35: ^{13}CNMR of compound **8a.**

8b ^{1}HNMR

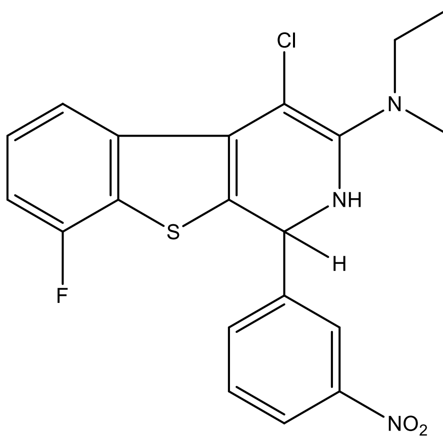

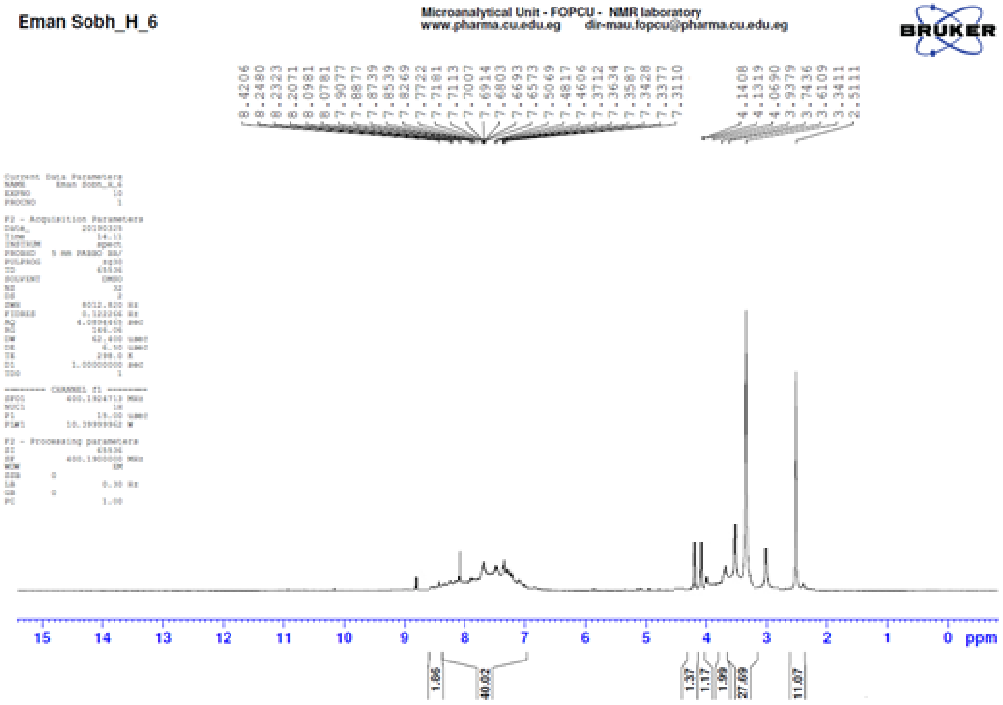

Figure S36: ^{1}HNMR of compound **8b.**

8c ^{1}HNMR

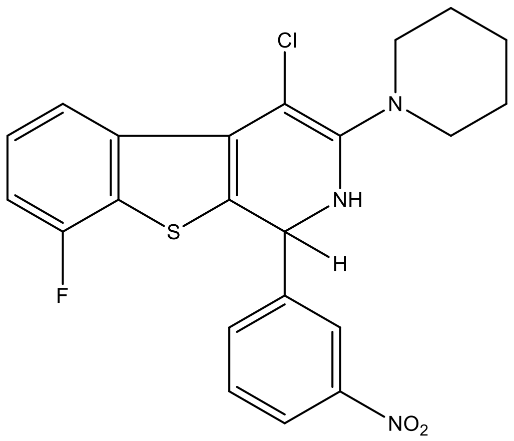

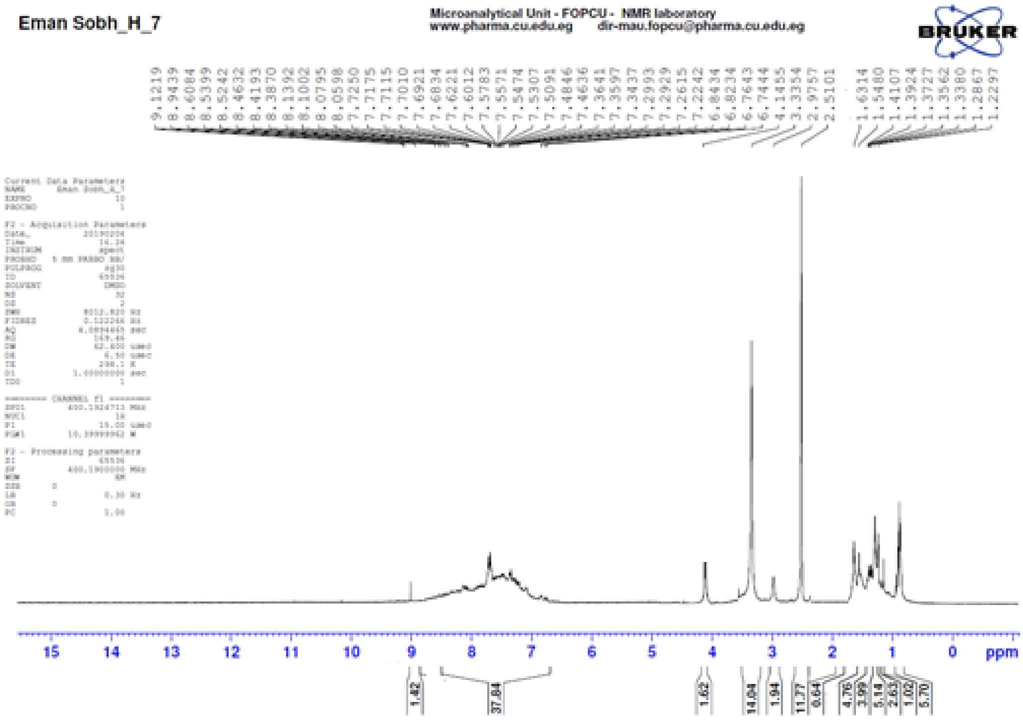

Figure S37: ^{1}HNMR of compound **8c.**

8c ^{13}CNMR

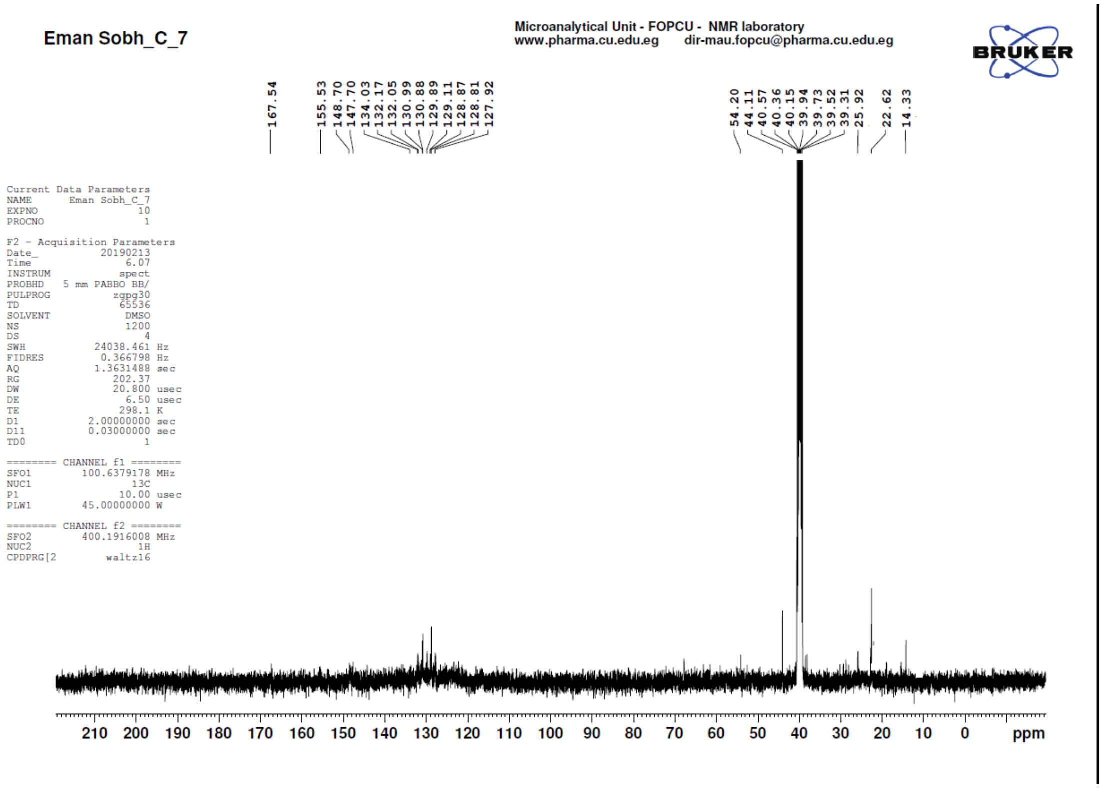

Figure S38: ^{13}CNMR of compound **8c.**

8d ^{1}HNMR

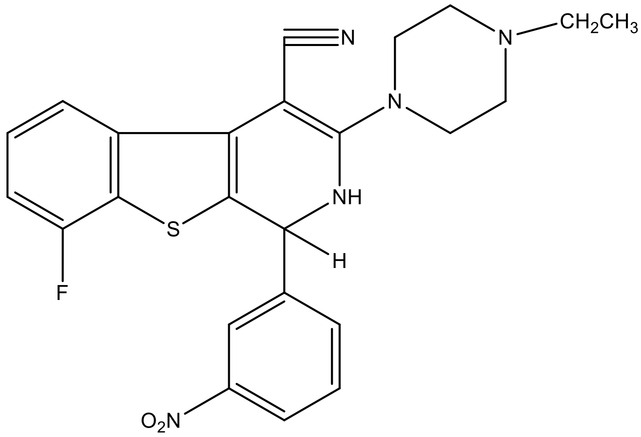

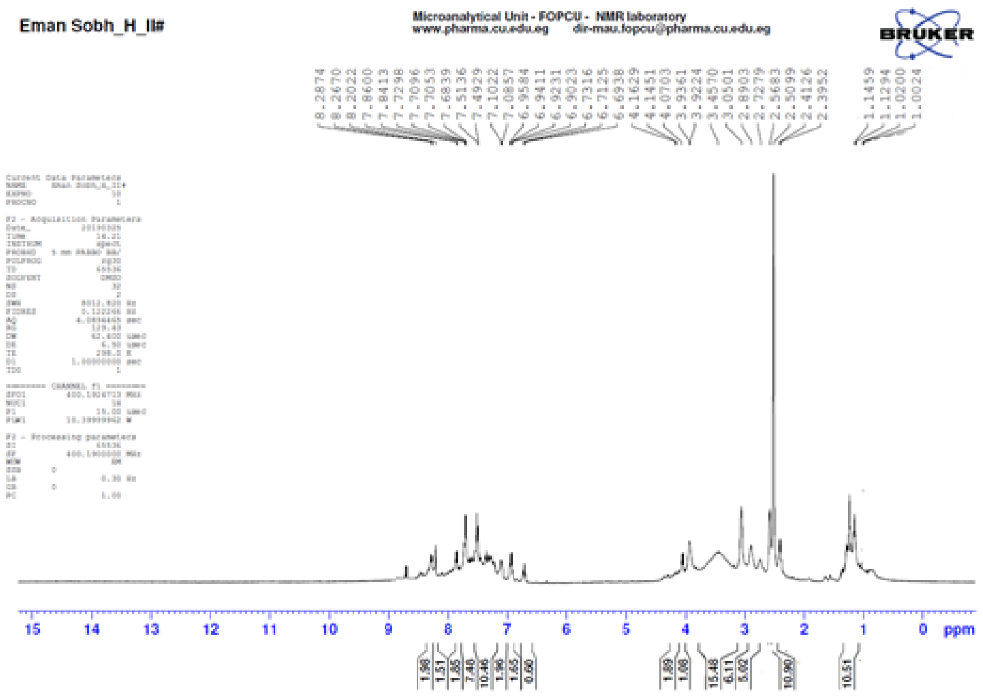

Figure S39: ^{1}HNMR of compound **8d.**

8e ^{1}HNMR

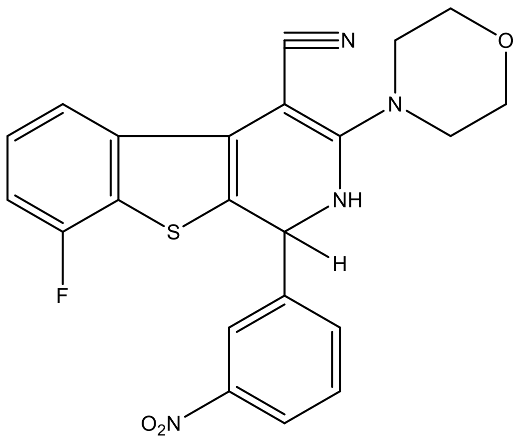

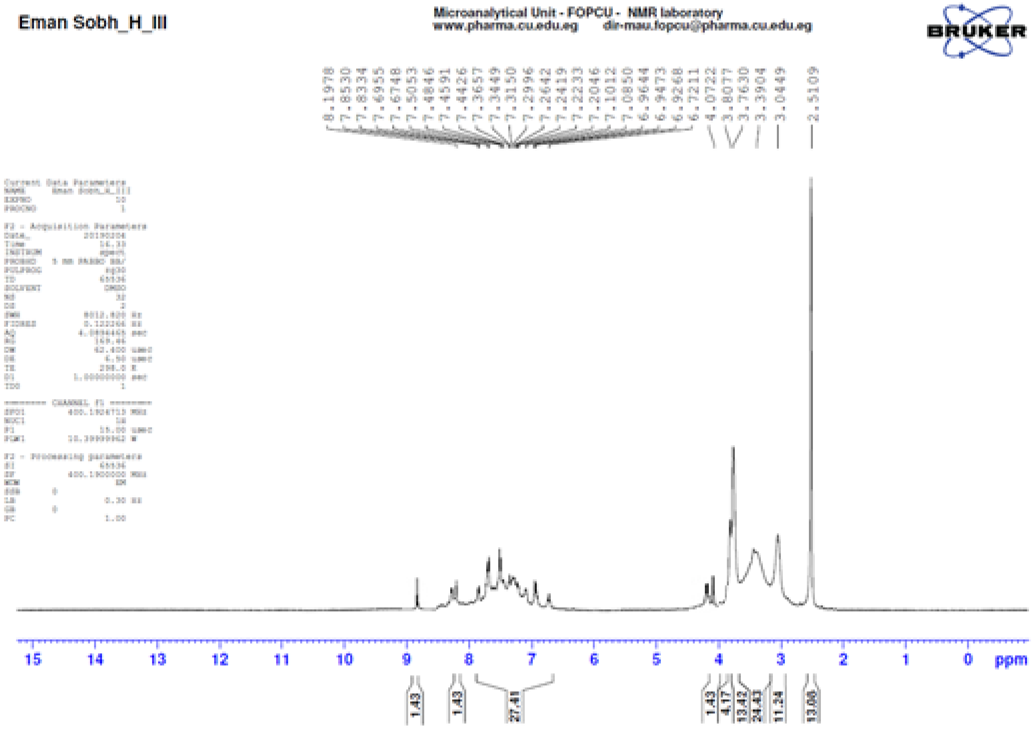

Figure S40: ^{1}HNMR of compound **8e.**

8e ^{13}CNMR

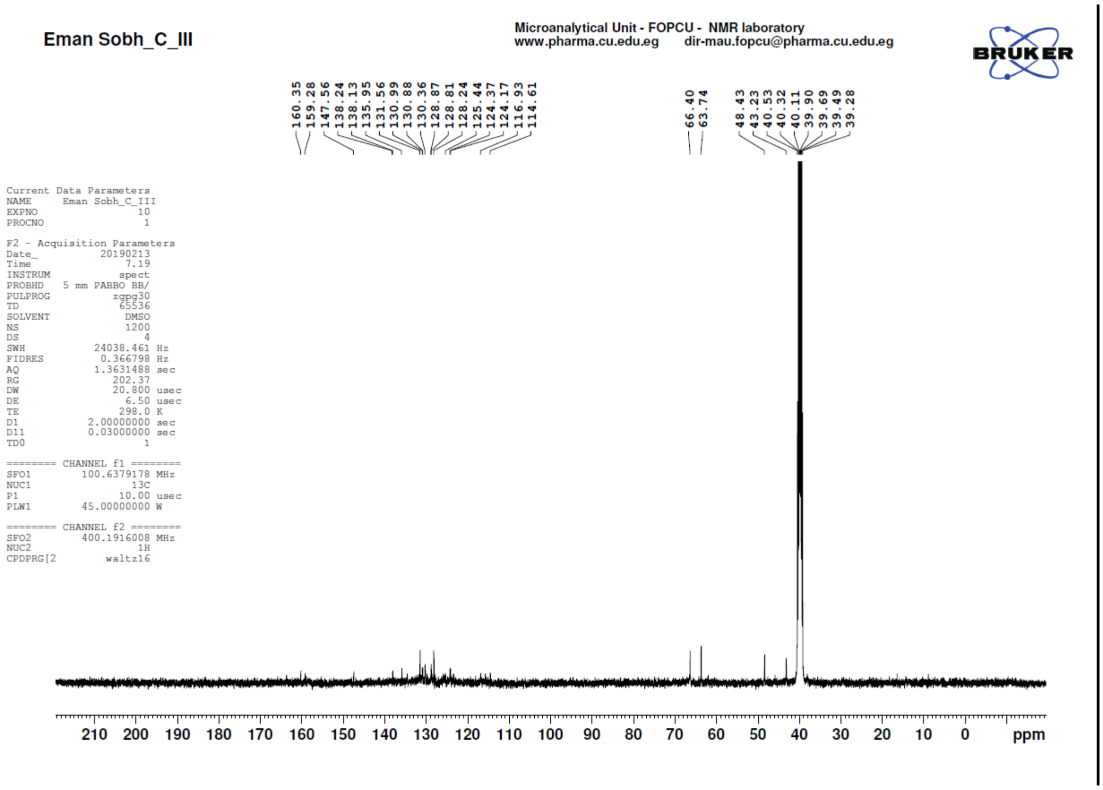

Figure S41: ^{13}CNMR of compound **8e.**

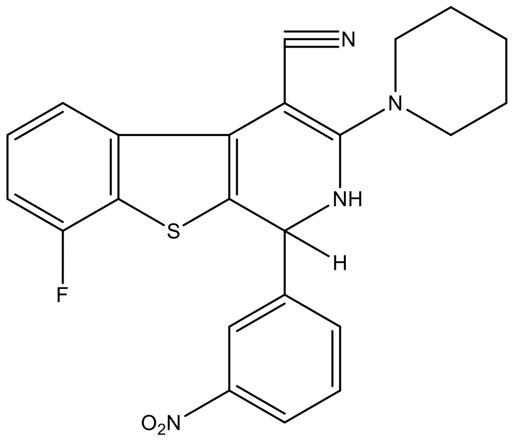

8f ^{1}HNMR

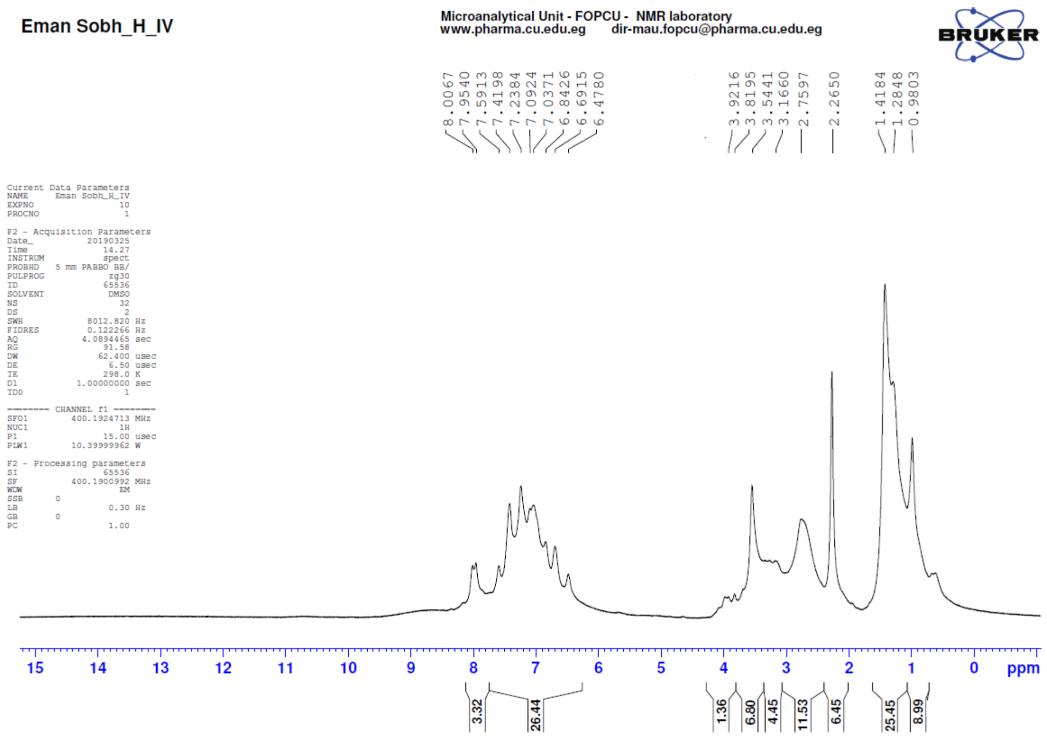

Figure S42: ^{1}HNMR of compound **8f.**

9 ^{1}HNMR

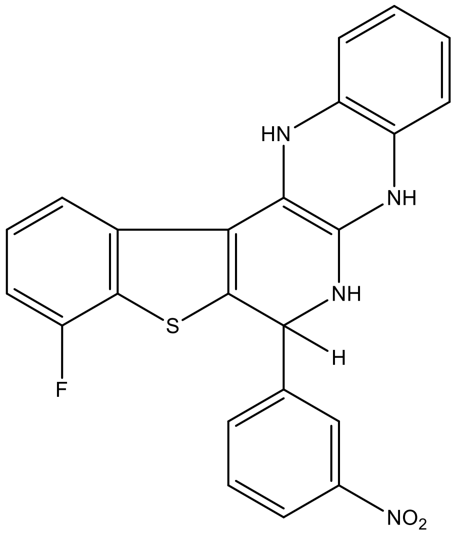

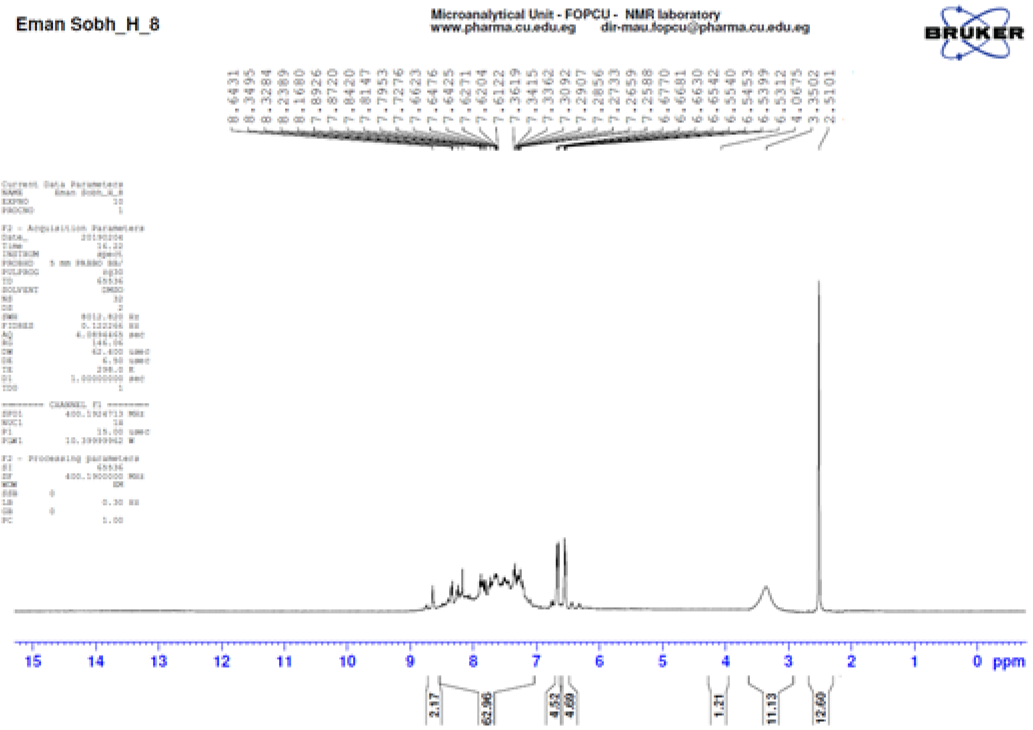

Figure S43: ^{1}HNMR of compound **9.**

9 ^{13}CNMR

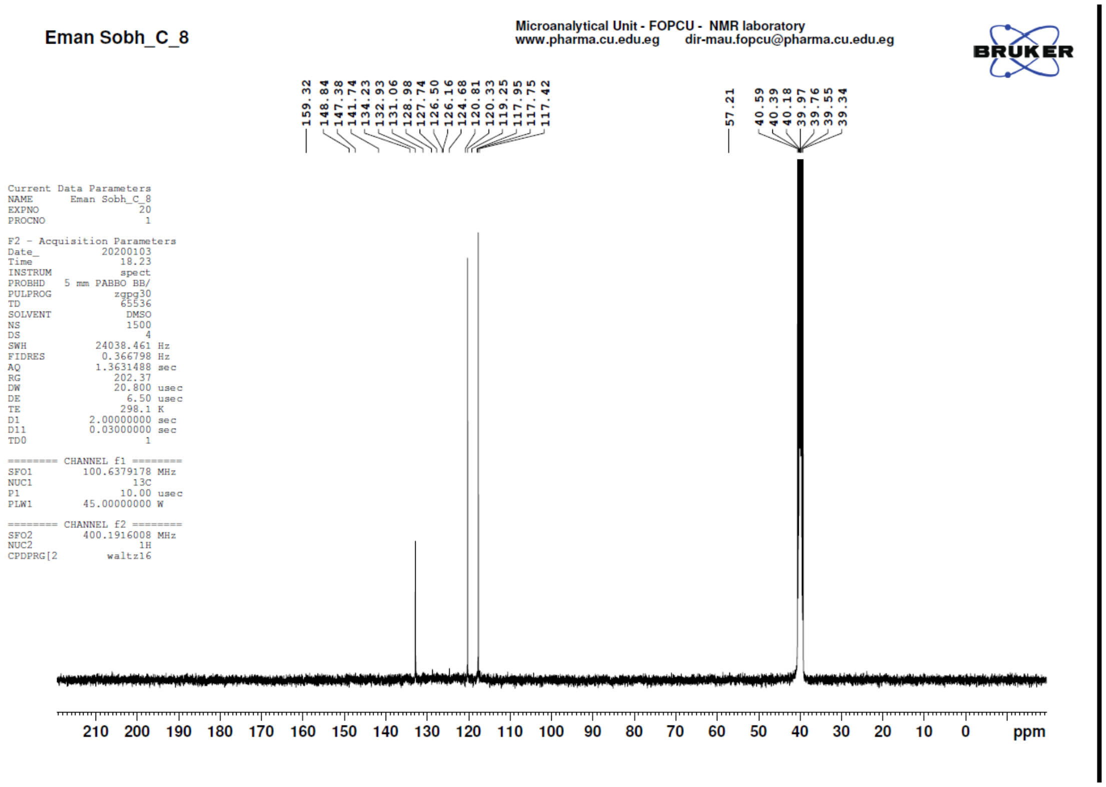

Figure S44: ^{13}CNMR of compound **9.**

10 ^{1}HNMR

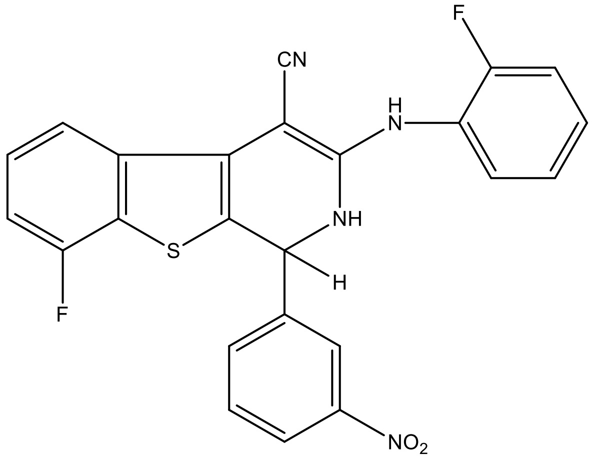

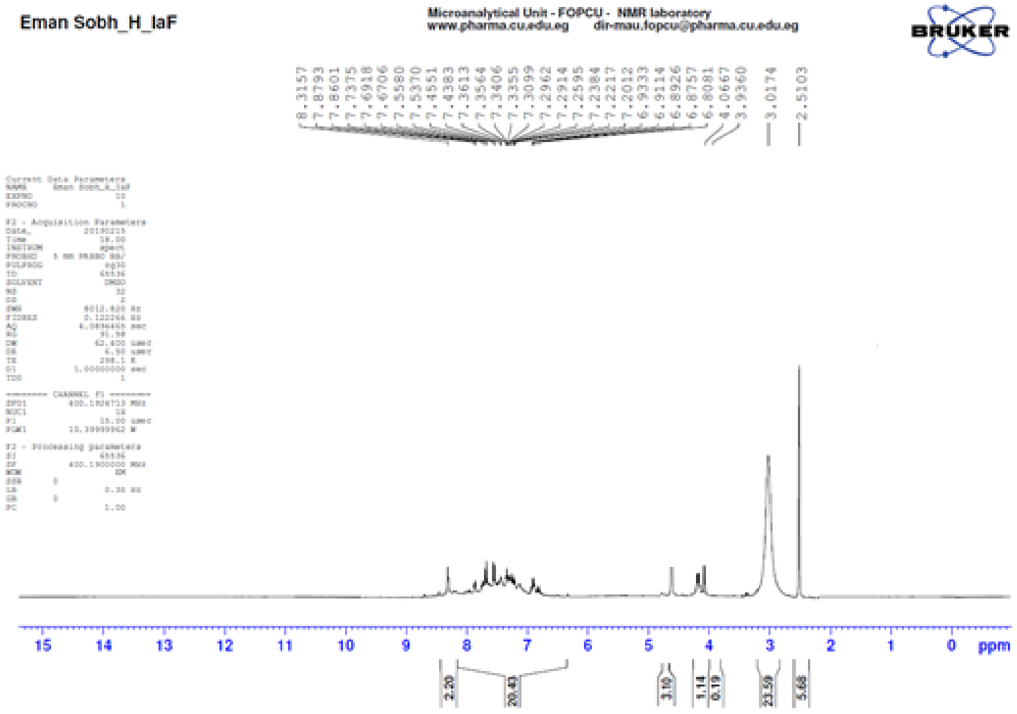

Figure S45: ^{1}HNMR of compound **10.**

10 ^{13}CNMR

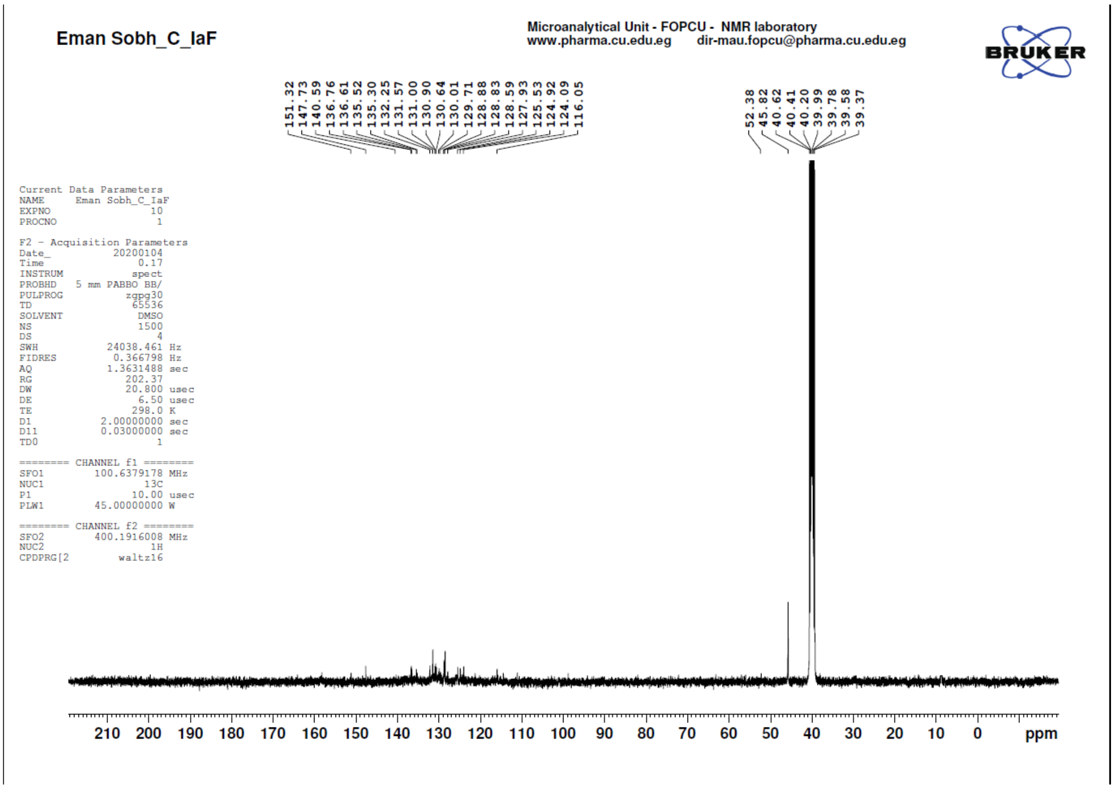

Figure S46: ^{13}CNMR of compound **10.**

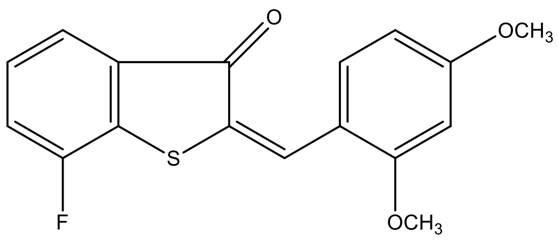

Developmental Therapeutics Program One Dose Mean Graph	NSC: D-817521 / 1	Conc: 1.00E-5 Molar	Test Date: Jul 29, 2019
	Experiment ID: 1907OS44		Report Date: Sep 10, 2019

Panel/Cell Line	Growth Percent
**Leukemia**	
CCRF-CEM	105.14
HL-60(TB)	89.03
K-562	95.24
MOLT-4	95.90
RPMI-8226	108.46
SR	93.71
**Non-Small Cell Lung Cancer**	
A549/ATCC	107.82
EKVX	105.71
HOP-62	94.01
HOP-92	108.14
NCI-H226	99.69
NCI-H23	104.04
NCI-H322M	99.75
NCI-H460	106.08
NCI-H522	93.86
**Colon Cancer**	
COLO 205	115.99
HCC-2998	112.75
HCT-116	103.66
HCT-15	109.73
HT29	109.18
KM12	105.55
SW-620	104.67
**CNS Cancer**	
SF-268	95.49
SF-295	104.69
SF-539	100.64
SNB-19	102.10
SNB-75	87.38
U251	107.91
**Melanoma**	
LOX IMVI	100.02
MALME-3M	95.81
M14	105.61
MDA-MB-435	100.27
SK-MEL-2	117.93
SK-MEL-28	107.93
SK-MEL-5	102.91
UACC-257	115.16
UACC-62	92.77
**Ovarian Cancer**	
IGROV1	99.54
OVCAR-3	107.34
OVCAR-4	106.44
OVCAR-5	109.42
OVCAR-8	104.32
NCI/ADR-RES	103.61
SK-OV-3	106.36
**Renal Cancer**	
786-0	112.20
A498	104.03
ACHN	104.06
CAKI-1	88.07
RXF 393	127.86
SN12C	100.73
TK-10	134.90
UO-31	75.11
**Prostate Cancer**	
PC-3	98.17
DU-145	108.28
**Breast Cancer**	
MCF7	95.93
MDA-MB-231/ATCC	110.15
BT-549	103.49
T-47D	103.25
MDA-MB-468	112.49
Mean	103.74
Delta	28.63
Range	59.79

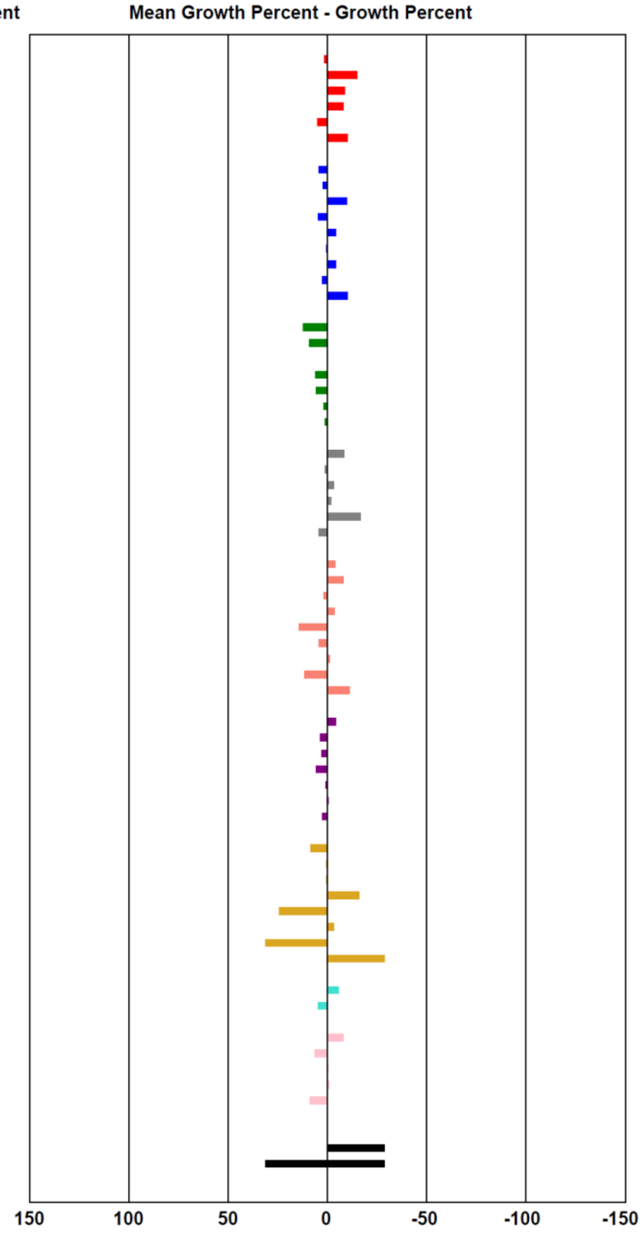

Figure S47: One-dose growth (%) and mean graph of compound **2a.**

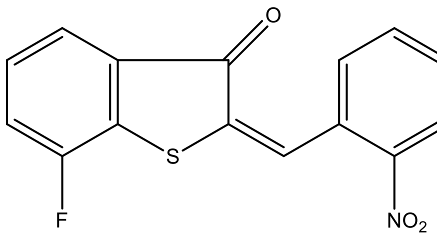

Developmental Therapeutics Program One Dose Mean Graph	NSC: D-817522 / 1	Conc: 1.00E-5 Molar	Test Date: Jul 29, 2019
	Experiment ID: 1907OS44		Report Date: Sep 10, 2019

Panel/Cell Line	Growth Percent
**Leukemia**	
CCRF-CEM	73.82
HL-60(TB)	72.25
K-562	33.12
MOLT-4	80.20
RPMI-8226	69.43
SR	62.75
**Non-Small Cell Lung Cancer**	
A549/ATCC	95.09
EKVX	84.37
HOP-62	79.73
HOP-92	111.40
NCI-H226	76.46
NCI-H23	86.84
NCI-H322M	98.02
NCI-H460	40.86
NCI-H522	97.06
**Colon Cancer**	
COLO 205	106.61
HCC-2998	111.43
HCT-116	19.50
HCT-15	101.05
HT29	107.17
KM12	96.73
SW-620	31.19
**CNS Cancer**	
SF-268	94.57
SF-295	72.97
SF-539	96.35
SNB-19	85.76
SNB-75	101.55
U251	67.46
**Melanoma**	
LOX IMVI	88.91
MALME-3M	87.45
M14	101.11
MDA-MB-435	112.11
SK-MEL-2	109.11
SK-MEL-28	104.83
SK-MEL-5	91.66
UACC-257	21.78
UACC-62	39.06
**Ovarian Cancer**	
IGROV1	33.49
OVCAR-3	29.12
OVCAR-4	59.56
OVCAR-5	56.22
OVCAR-8	93.25
NCI/ADR-RES	86.52
SK-OV-3	97.58
**Renal Cancer**	
786-0	110.98
A498	44.91
ACHN	92.72
CAKI-1	14.93
RXF 393	103.10
SN12C	92.43
TK-10	130.93
UO-31	70.09
**Prostate Cancer**	
PC-3	93.52
DU-145	108.77
**Breast Cancer**	
MCF7	38.39
MDA-MB-231/ATCC	92.57
BT-549	93.63
T-47D	63.86
MDA-MB-468	16.32
Mean	78.52
Delta	63.59
Range	116.00

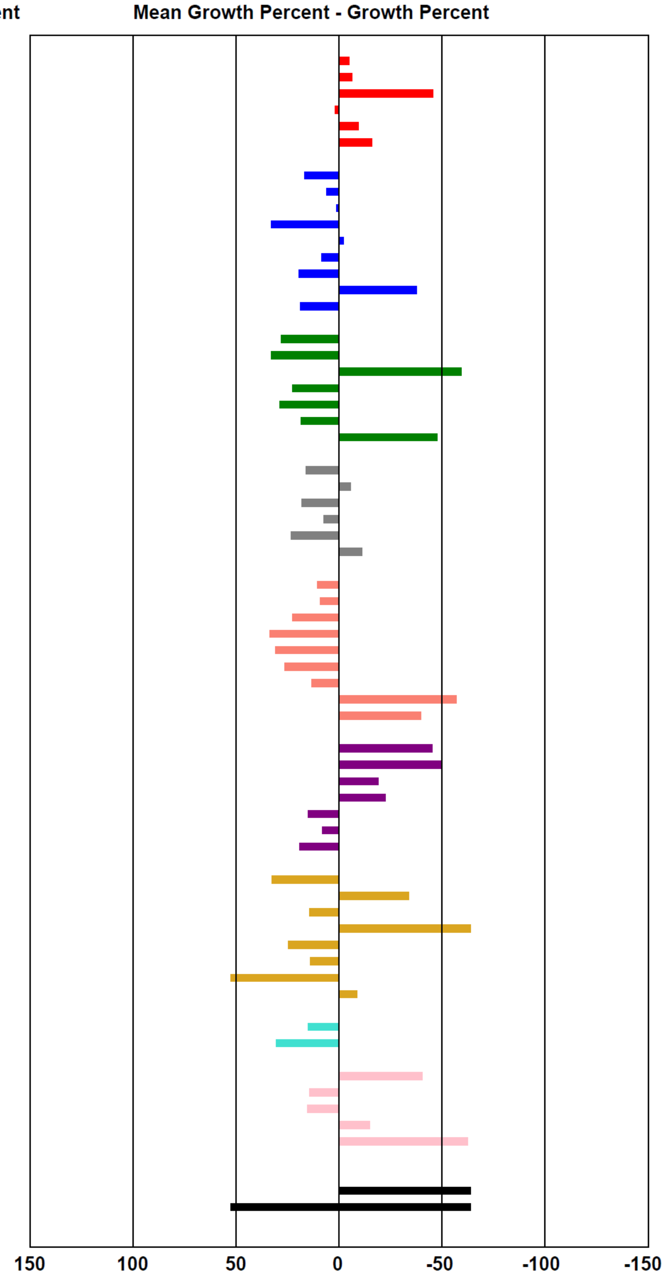

Figure S48: One-dose growth (%) and mean graph of compound **2c.**

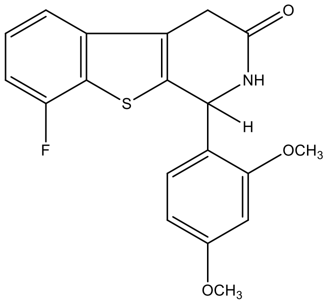

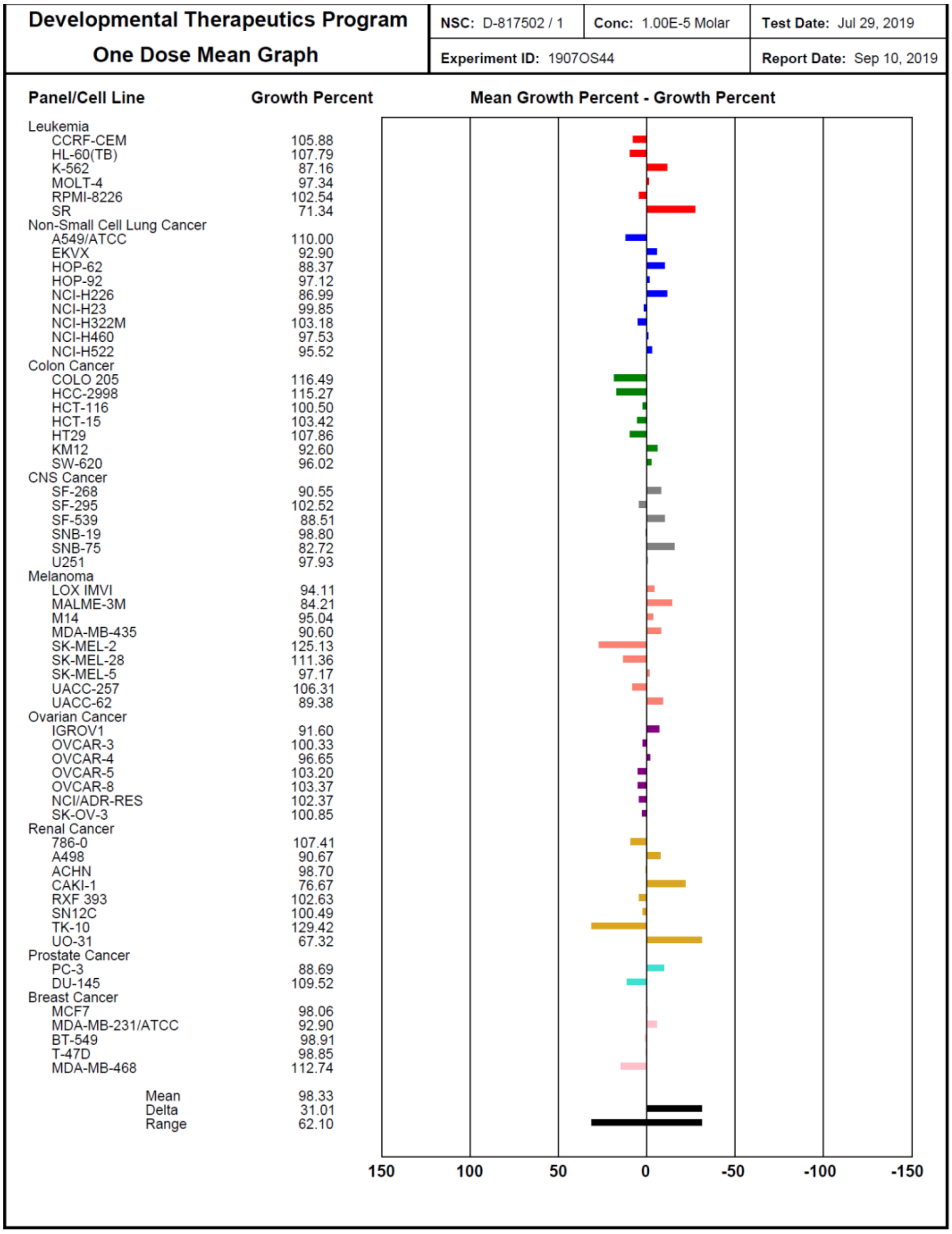

Figure S49: One-dose growth (%) and mean graph of compound **3a.**

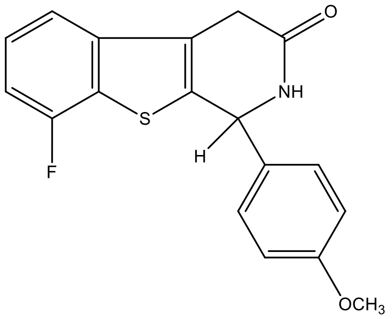

Developmental Therapeutics Program | **NSC:** D-817503 / 1 | **Conc:** 1.00E-5 Molar | **Test Date:** Jul 29, 2019

One Dose Mean Graph | **Experiment ID:** 1907OS44 | **Report Date:** Sep 10, 2019

Panel/Cell Line	Growth Percent
**Leukemia**	
CCRF-CEM	109.35
HL-60(TB)	107.38
K-562	65.50
MOLT-4	99.73
RPMI-8226	98.89
SR	69.53
**Non-Small Cell Lung Cancer**	
A549/ATCC	41.93
EKVX	87.00
HOP-62	91.74
HOP-92	94.57
NCI-H226	90.01
NCI-H23	103.36
NCI-H322M	83.75
NCI-H460	63.07
NCI-H522	88.60
**Colon Cancer**	
COLO 205	117.67
HCC-2998	112.11
HCT-116	100.50
HCT-15	91.68
HT29	110.51
KM12	95.77
SW-620	95.03
**CNS Cancer**	
SF-268	88.15
SF-295	97.26
SF-539	87.44
SNB-19	92.43
SNB-75	74.56
U251	86.39
**Melanoma**	
LOX IMVI	97.79
MALME-3M	88.93
M14	93.22
MDA-MB-435	68.20
SK-MEL-2	112.61
SK-MEL-28	98.50
SK-MEL-5	98.01
UACC-257	112.46
UACC-62	88.35
**Ovarian Cancer**	
IGROV1	89.75
OVCAR-3	102.13
OVCAR-4	100.19
OVCAR-5	108.59
OVCAR-8	101.02
NCI/ADR-RES	102.15
SK-OV-3	102.91
**Renal Cancer**	
786-0	108.85
A498	95.78
ACHN	102.10
CAKI-1	83.67
RXF 393	118.20
SN12C	100.17
TK-10	129.74
UO-31	74.19
**Prostate Cancer**	
PC-3	97.87
DU-145	107.27
**Breast Cancer**	
MCF7	79.77
MDA-MB-231/ATCC	97.57
BT-549	96.88
T-47D	106.65
MDA-MB-468	37.41
Mean	93.98
Delta	56.57
Range	92.33

Mean Growth Percent - Growth Percent

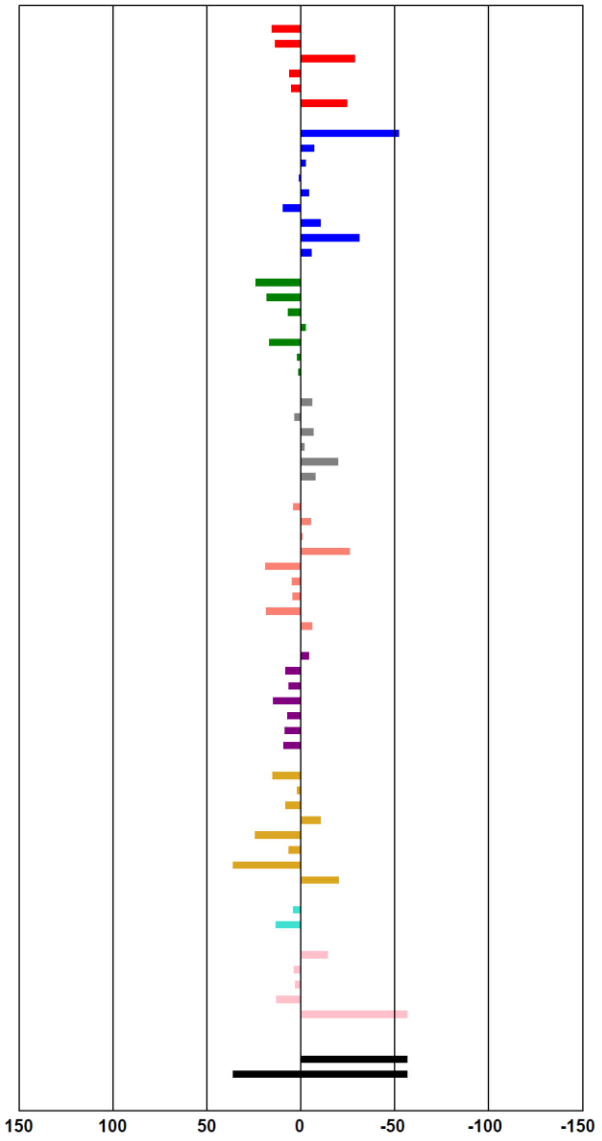

Figure S50: One-dose growth (%) and mean graph of compound **3b.**

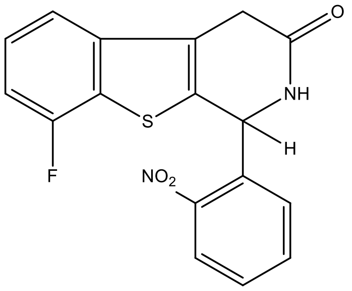

Developmental Therapeutics Program
One Dose Mean Graph

NSC: D-817504 / 1 | **Conc:** 1.00E-5 Molar | **Test Date:** Jul 29, 2019
Experiment ID: 1907OS44 | **Report Date:** Sep 10, 2019

Panel/Cell Line	Growth Percent
Leukemia	
CCRF-CEM	110.16
HL-60(TB)	115.87
K-562	97.14
MOLT-4	105.98
RPMI-8226	95.38
SR	99.40
Non-Small Cell Lung Cancer	
A549/ATCC	105.73
EKVX	85.16
HOP-62	91.48
HOP-92	92.22
NCI-H226	79.28
NCI-H23	98.59
NCI-H322M	99.00
NCI-H460	76.87
NCI-H522	101.98
Colon Cancer	
COLO 205	112.74
HCC-2998	118.72
HCT-116	85.93
HCT-15	110.00
HT29	112.09
KM12	100.49
SW-620	79.30
CNS Cancer	
SF-268	93.84
SF-295	100.58
SF-539	89.37
SNB-19	100.33
SNB-75	89.97
U251	85.22
Melanoma	
LOX IMVI	97.69
MALME-3M	94.47
M14	100.72
MDA-MB-435	107.97
SK-MEL-2	125.53
SK-MEL-28	120.20
SK-MEL-5	97.28
UACC-257	119.98
UACC-62	87.17
Ovarian Cancer	
IGROV1	63.45
OVCAR-3	97.92
OVCAR-4	93.00
OVCAR-5	108.05
OVCAR-8	107.33
NCI/ADR-RES	105.76
SK-OV-3	93.06
Renal Cancer	
786-0	109.68
A498	115.47
ACHN	100.56
CAKI-1	75.13
RXF 393	124.83
SN12C	99.06
TK-10	141.81
UO-31	69.58
Prostate Cancer	
PC-3	100.39
DU-145	107.29
Breast Cancer	
MCF7	81.36
MDA-MB-231/ATCC	84.90
BT-549	99.62
T-47D	78.60
MDA-MB-468	86.92
Mean	98.77
Delta	35.32
Range	78.36

Mean Growth Percent - Growth Percent

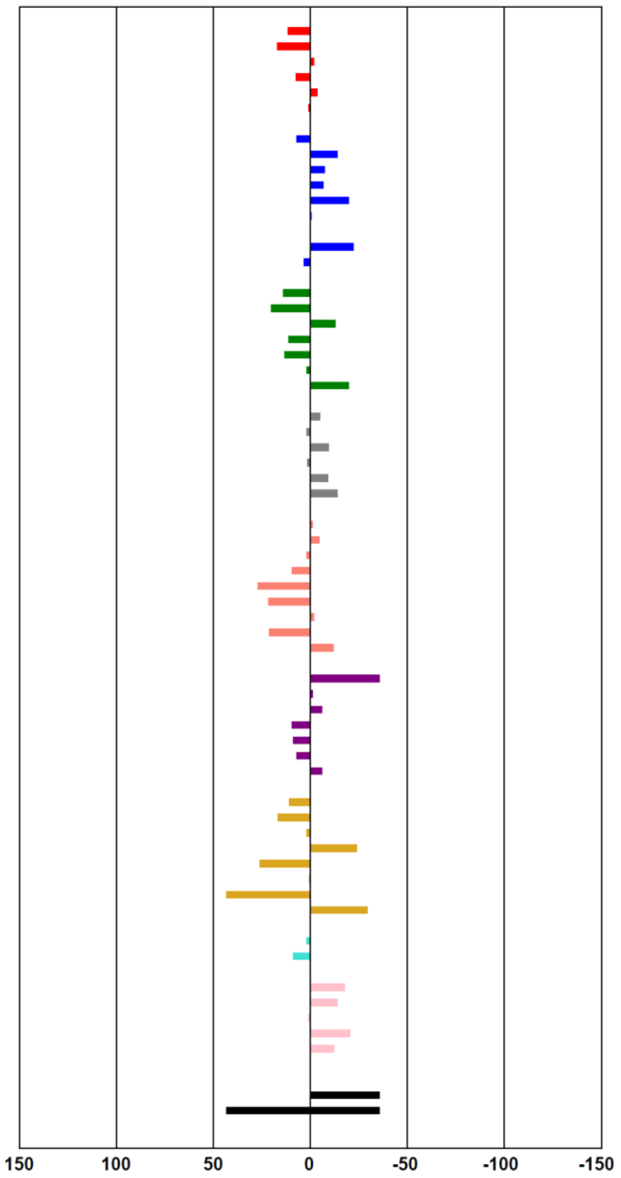

Figure S51: One-dose growth (%) and mean graph of compound **3c.**

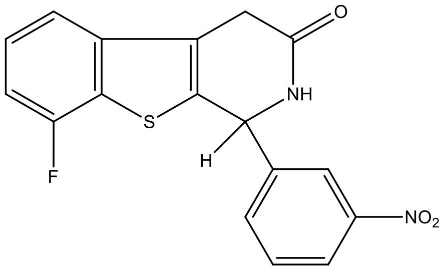

Developmental Therapeutics Program One Dose Mean Graph	NSC: D-817505 / 1	Conc: 1.00E-5 Molar	Test Date: Jul 29, 2019
	Experiment ID: 1907OS44		Report Date: Sep 10, 2019

Panel/Cell Line	Growth Percent
Leukemia	
CCRF-CEM	90.10
HL-60(TB)	88.00
K-562	84.22
MOLT-4	87.10
RPMI-8226	70.96
SR	76.19
Non-Small Cell Lung Cancer	
A549/ATCC	105.27
EKVX	92.02
HOP-62	85.68
HOP-92	89.04
NCI-H226	78.68
NCI-H23	98.96
NCI-H322M	97.22
NCI-H460	99.47
NCI-H522	82.45
Colon Cancer	
COLO 205	107.27
HCC-2998	106.11
HCT-116	76.93
HCT-15	78.77
HT29	99.97
KM12	96.07
SW-620	97.61
CNS Cancer	
SF-268	85.40
SF-295	97.70
SF-539	93.41
SNB-19	93.77
SNB-75	78.47
U251	79.43
Melanoma	
LOX IMVI	87.33
MALME-3M	106.26
M14	98.64
MDA-MB-435	103.38
SK-MEL-2	101.12
SK-MEL-28	109.19
SK-MEL-5	94.45
UACC-257	107.53
UACC-62	85.93
Ovarian Cancer	
IGROV1	79.55
OVCAR-3	99.91
OVCAR-4	101.26
OVCAR-5	108.70
OVCAR-8	97.14
NCI/ADR-RES	95.50
SK-OV-3	103.95
Renal Cancer	
786-0	110.00
A498	95.22
ACHN	93.30
CAKI-1	78.18
RXF 393	105.23
SN12C	95.96
TK-10	152.28
UO-31	65.16
Prostate Cancer	
PC-3	85.33
DU-145	106.90
Breast Cancer	
MCF7	66.79
MDA-MB-231/ATCC	92.82
BT-549	94.68
T-47D	86.66
MDA-MB-468	106.05
Mean	93.74
Delta	28.58
Range	87.12

Mean Growth Percent - Growth Percent

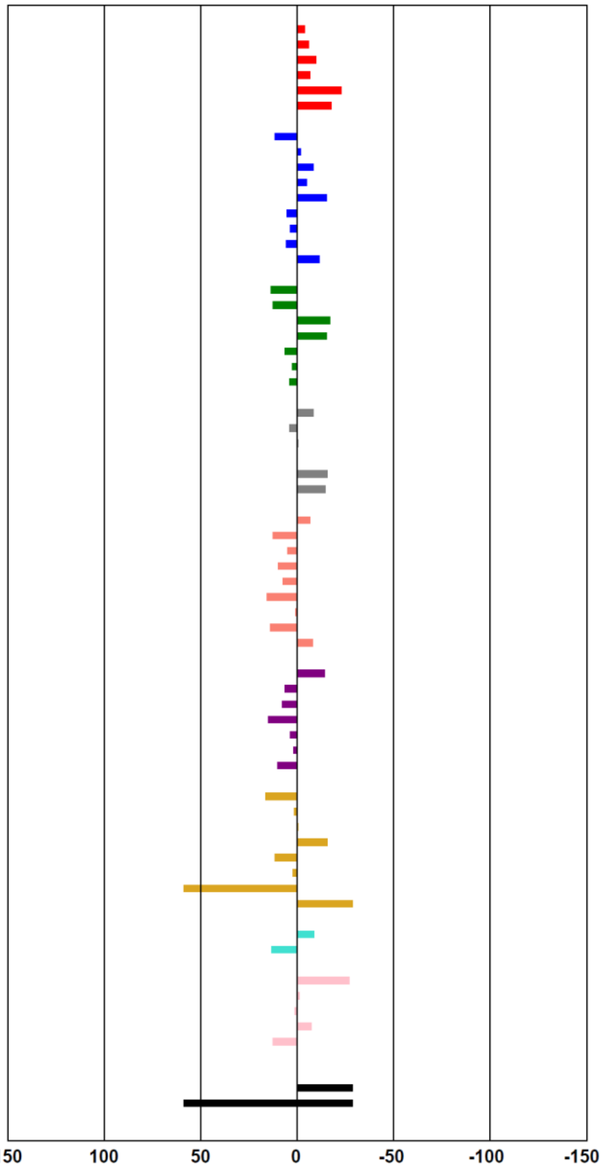

Figure S52: One-dose growth (%) and mean graph of compound **3d.**

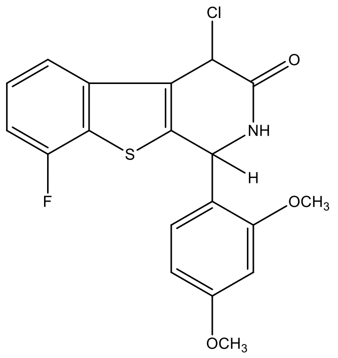

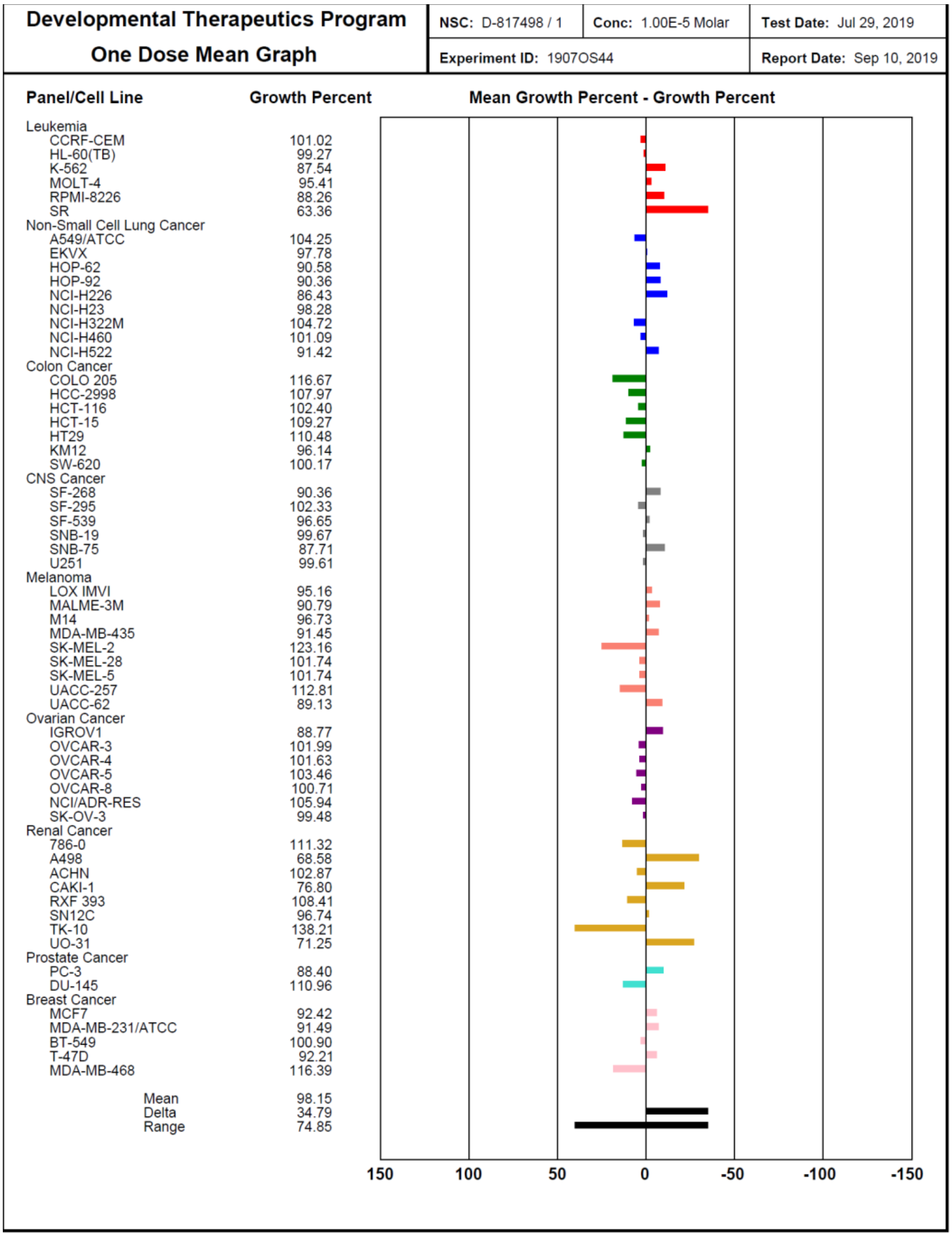

Figure S53: One-dose growth (%) and mean graph of compound **4a.**

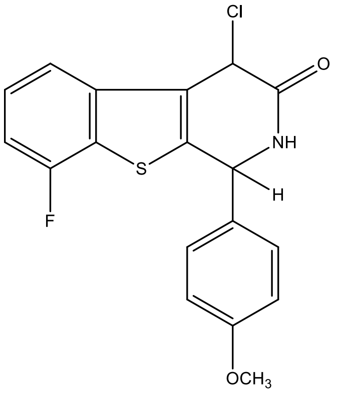

Developmental Therapeutics Program

One Dose Mean Graph

**NSC:** D-817499 / 1	**Conc:** 1.00E-5 Molar	**Test Date:** Jul 29, 2019	
**Experiment ID:** 1907OS44		**Report Date:** Sep 10, 2019	

Panel/Cell Line	Growth Percent
Leukemia	
CCRF-CEM	100.42
HL-60(TB)	105.26
K-562	68.68
MOLT-4	92.11
RPMI-8226	92.68
SR	60.08
Non-Small Cell Lung Cancer	
A549/ATCC	45.55
EKVX	87.04
HOP-62	82.84
HOP-92	83.59
NCI-H226	92.86
NCI-H23	99.35
NCI-H322M	87.98
NCI-H460	67.58
NCI-H522	78.28
Colon Cancer	
COLO 205	120.46
HCC-2998	106.39
HCT-116	98.38
HCT-15	85.80
HT29	106.98
KM12	92.41
SW-620	93.51
CNS Cancer	
SF-268	85.72
SF-295	98.18
SF-539	84.12
SNB-19	92.53
SNB-75	77.16
U251	78.94
Melanoma	
LOX IMVI	93.54
MALME-3M	89.23
M14	96.15
MDA-MB-435	66.34
SK-MEL-2	118.30
SK-MEL-28	95.24
SK-MEL-5	99.93
UACC-257	106.58
UACC-62	91.26
Ovarian Cancer	
IGROV1	85.14
OVCAR-3	99.39
OVCAR-4	92.49
OVCAR-5	112.95
OVCAR-8	97.05
NCI/ADR-RES	90.82
SK-OV-3	102.04
Renal Cancer	
786-0	100.46
A498	88.91
ACHN	103.20
CAKI-1	82.34
RXF 393	93.02
SN12C	96.34
TK-10	138.15
UO-31	68.32
Prostate Cancer	
PC-3	95.15
DU-145	106.94
Breast Cancer	
MCF7	89.38
MDA-MB-231/ATCC	101.54
BT-549	96.49
T-47D	86.45
MDA-MB-468	41.05
Mean	91.34
Delta	50.29
Range	97.10

Mean Growth Percent - Growth Percent

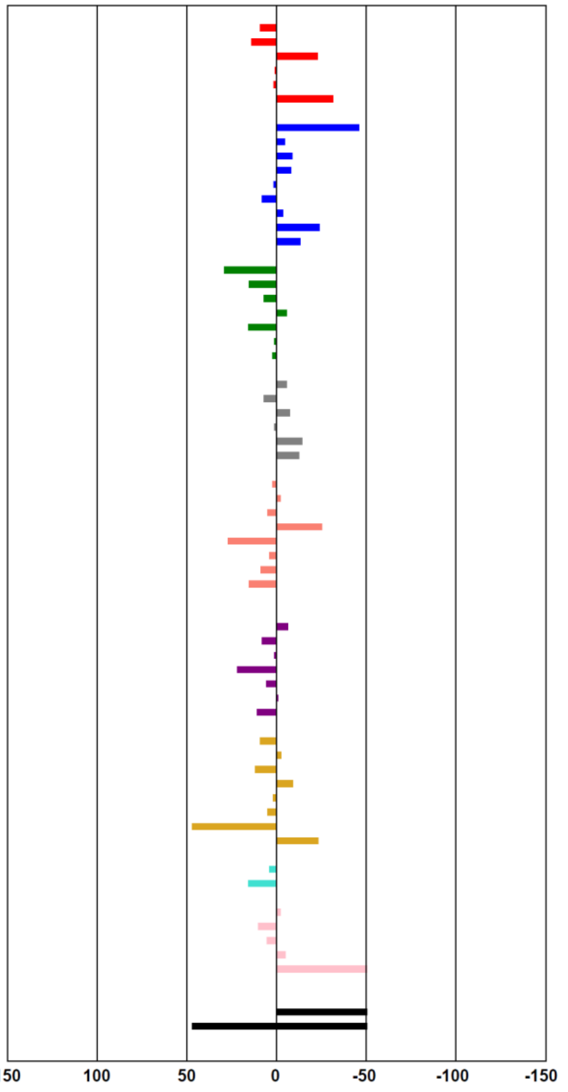

Figure S54: One-dose growth (%) and mean graph of compound **4b.**

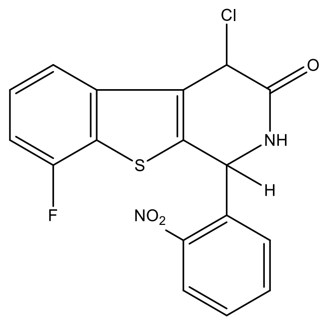

Developmental Therapeutics Program
One Dose Mean Graph

NSC: D-817500 / 1 | **Conc:** 1.00E-5 Molar | **Test Date:** Jul 29, 2019
Experiment ID: 1907OS44 | **Report Date:** Sep 10, 2019

Panel/Cell Line	Growth Percent
Leukemia	
CCRF-CEM	100.04
HL-60(TB)	105.53
K-562	76.50
MOLT-4	88.12
RPMI-8226	94.10
SR	90.80
Non-Small Cell Lung Cancer	
A549/ATCC	96.85
EKVX	88.20
HOP-62	96.68
HOP-92	111.25
NCI-H226	81.53
NCI-H23	93.05
NCI-H322M	104.23
NCI-H460	72.82
NCI-H522	93.05
Colon Cancer	
COLO 205	121.61
HCC-2998	127.42
HCT-116	88.56
HCT-15	101.51
HT29	109.93
KM12	100.66
SW-620	97.57
CNS Cancer	
SF-268	89.46
SF-295	107.26
SF-539	96.64
SNB-19	102.16
SNB-75	88.34
U251	91.18
Melanoma	
LOX IMVI	91.28
MALME-3M	90.54
M14	98.30
MDA-MB-435	107.17
SK-MEL-2	116.71
SK-MEL-28	118.43
SK-MEL-5	91.55
UACC-257	100.18
UACC-62	79.16
Ovarian Cancer	
IGROV1	67.04
OVCAR-3	92.75
OVCAR-4	88.86
OVCAR-5	102.88
OVCAR-8	98.27
NCI/ADR-RES	101.98
SK-OV-3	109.85
Renal Cancer	
786-0	116.91
A498	106.23
ACHN	104.45
CAKI-1	87.59
RXF 393	117.33
SN12C	95.24
TK-10	151.99
UO-31	76.88
Prostate Cancer	
PC-3	85.49
DU-145	108.16
Breast Cancer	
MCF7	70.36
MDA-MB-231/ATCC	88.75
BT-549	97.30
T-47D	66.38
MDA-MB-468	80.48
Mean	97.01
Delta	30.63
Range	85.61

Mean Growth Percent - Growth Percent

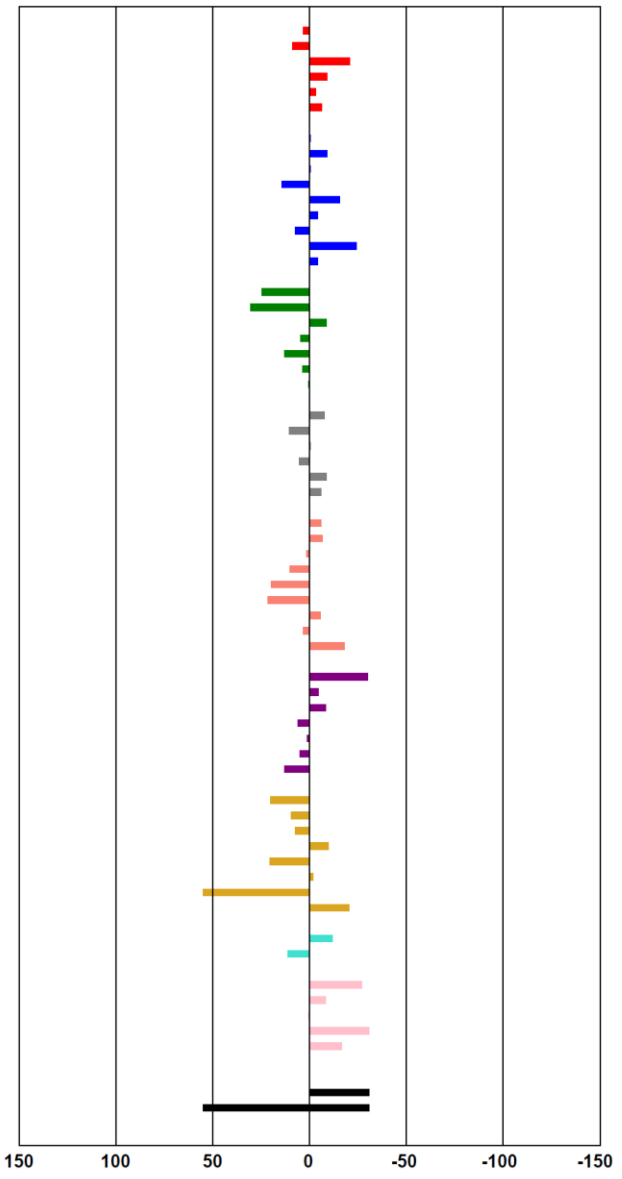

Figure S55: One-dose growth (%) and mean graph of compound **4c.**

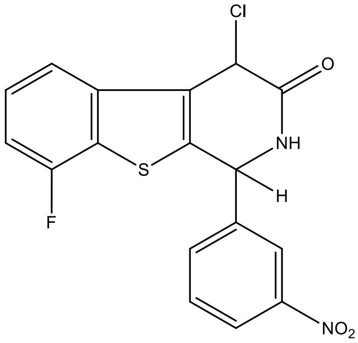

Developmental Therapeutics Program<br>One Dose Mean Graph	NSC: D-817501 / 1	Conc: 1.00E-5 Molar	Test Date: Jul 29, 2019
	Experiment ID: 1907OS44		Report Date: Sep 10, 2019

Panel/Cell Line	Growth Percent
Leukemia	
CCRF-CEM	98.01
HL-60(TB)	116.76
K-562	87.04
MOLT-4	90.82
RPMI-8226	94.39
SR	88.62
Non-Small Cell Lung Cancer	
A549/ATCC	102.89
EKVX	99.13
HOP-62	98.88
HOP-92	94.32
NCI-H226	94.74
NCI-H23	101.19
NCI-H322M	100.93
NCI-H460	98.57
NCI-H522	91.45
Colon Cancer	
COLO 205	117.16
HCC-2998	115.95
HCT-116	80.46
HCT-15	98.69
HT29	97.70
KM12	105.21
SW-620	96.57
CNS Cancer	
SF-268	93.06
SF-295	103.10
SF-539	95.81
SNB-19	100.59
SNB-75	95.77
U251	94.75
Melanoma	
LOX IMVI	94.88
MALME-3M	95.38
M14	100.99
MDA-MB-435	107.63
SK-MEL-2	126.10
SK-MEL-28	110.50
SK-MEL-5	95.95
UACC-257	115.75
UACC-62	89.63
Ovarian Cancer	
IGROV1	77.33
OVCAR-3	104.91
OVCAR-4	98.04
OVCAR-5	103.92
OVCAR-8	101.82
NCI/ADR-RES	108.07
SK-OV-3	110.24
Renal Cancer	
786-0	111.18
A498	100.03
ACHN	93.72
CAKI-1	82.60
RXF 393	118.11
SN12C	97.05
TK-10	140.69
UO-31	65.30
Prostate Cancer	
PC-3	97.20
DU-145	108.57
Breast Cancer	
MCF7	91.66
MDA-MB-231/ATCC	94.88
BT-549	96.38
T-47D	91.30
MDA-MB-468	108.27
Mean	99.84
Delta	34.54
Range	75.39

Mean Growth Percent - Growth Percent

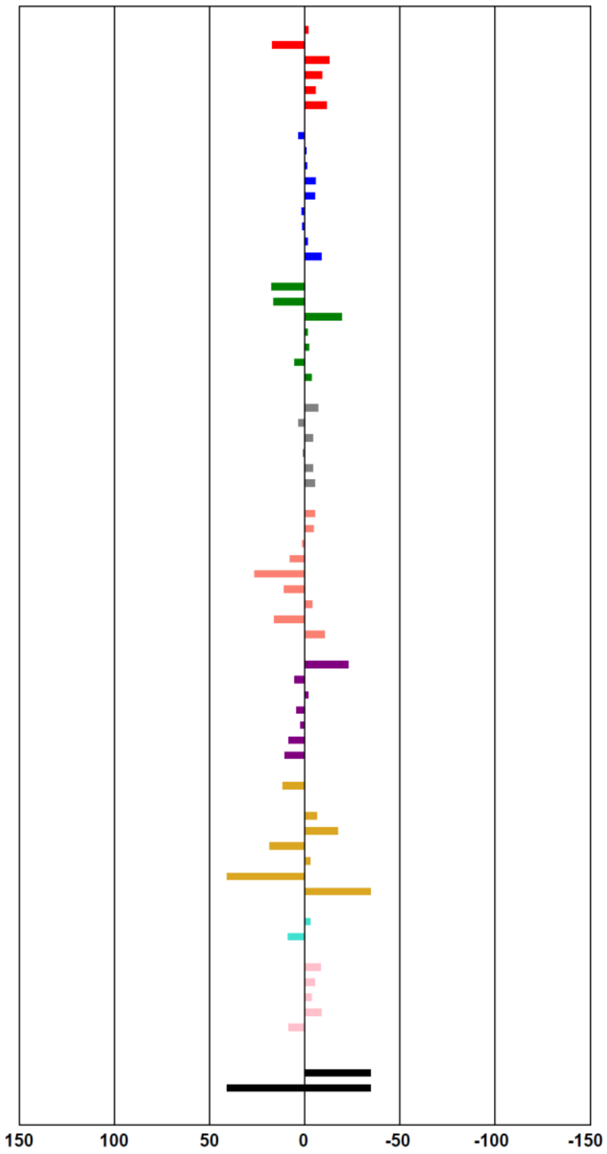

Figure S56: One-dose growth (%) and mean graph of compound **4d.**

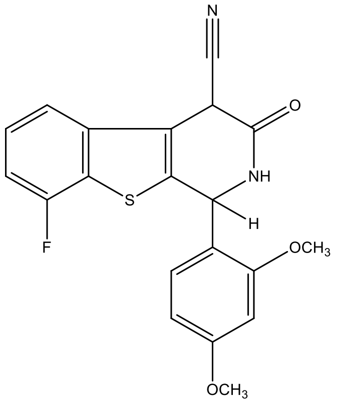

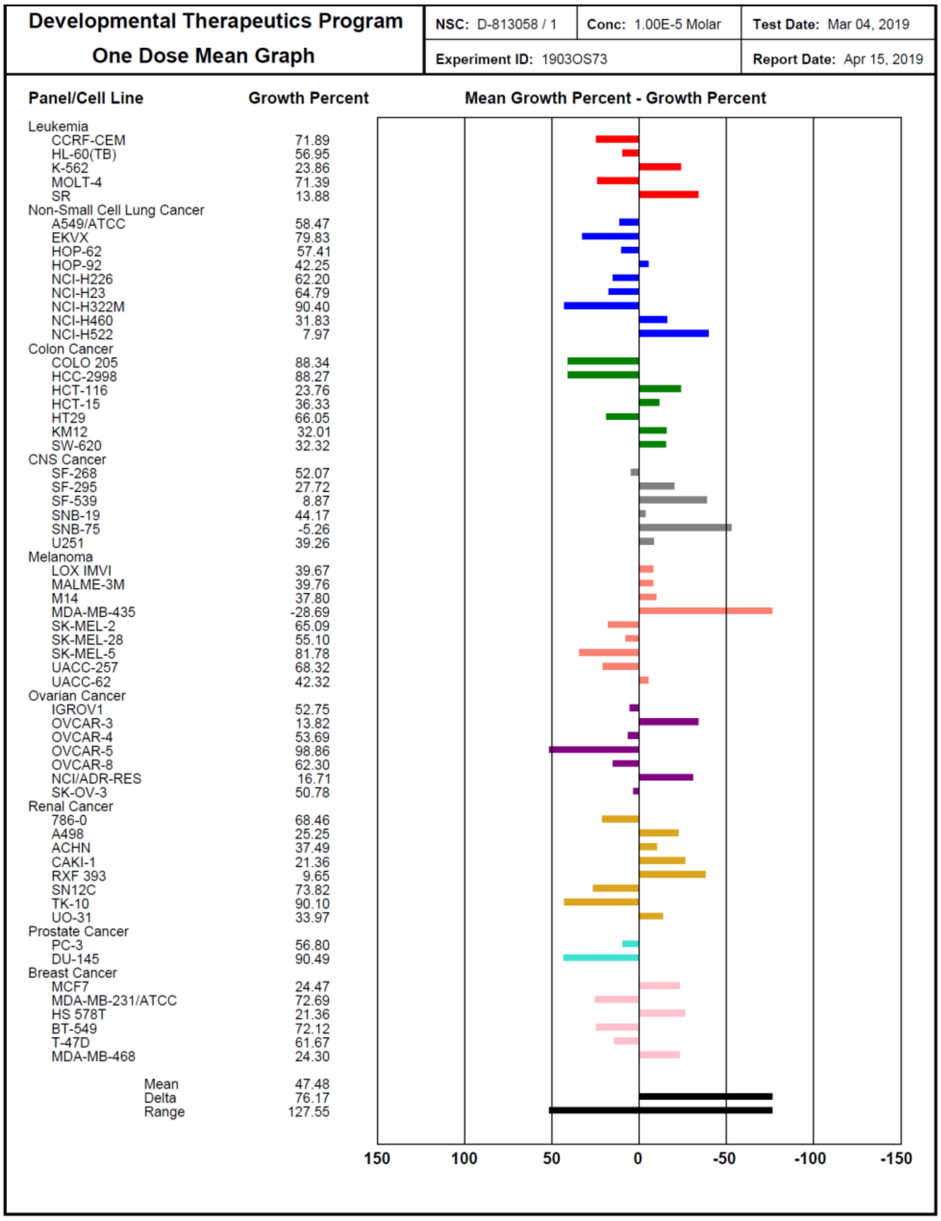

Figure S57: One-dose growth (%) and mean graph of compound **5a.**

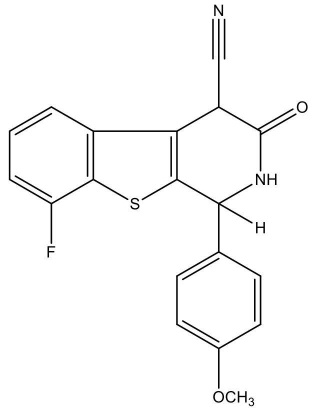

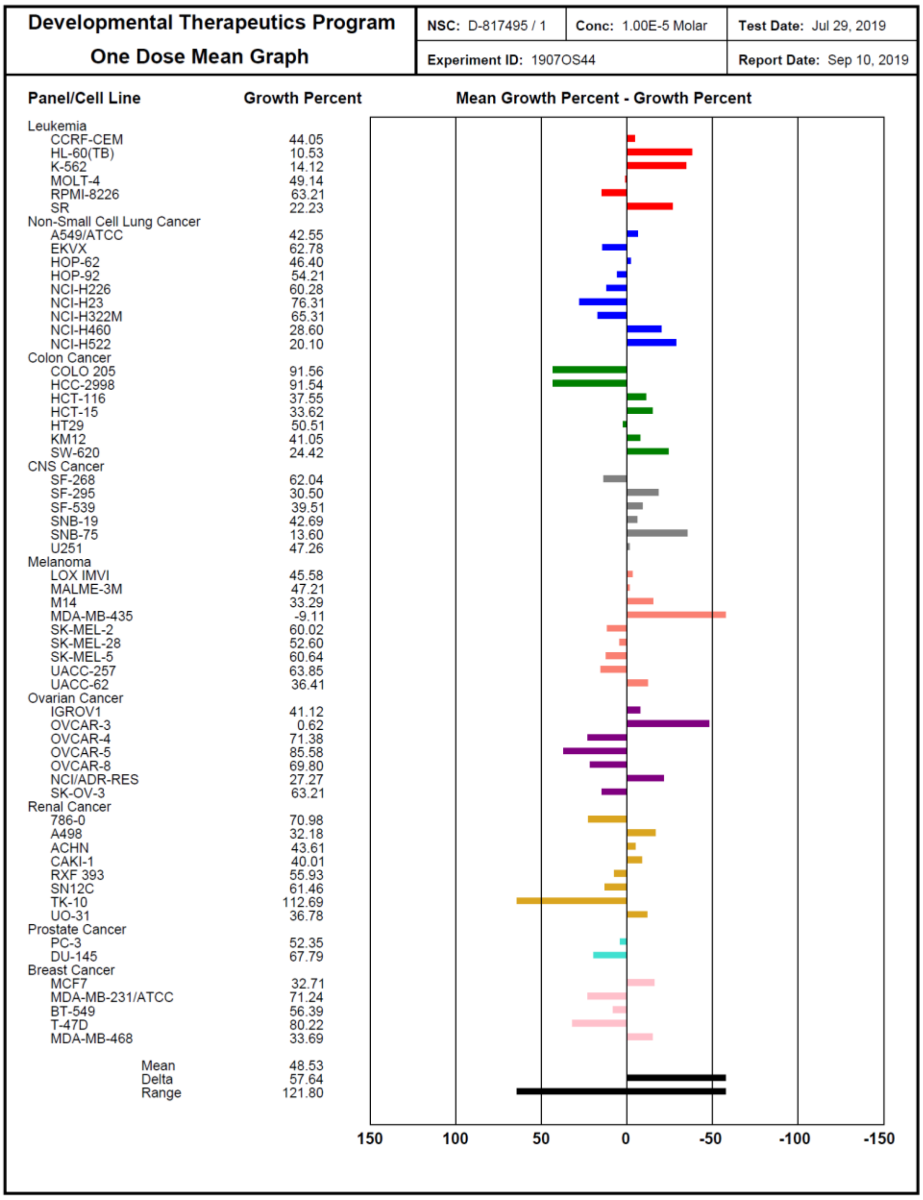

Figure S58: One-dose growth (%) and mean graph of compound **5b.**

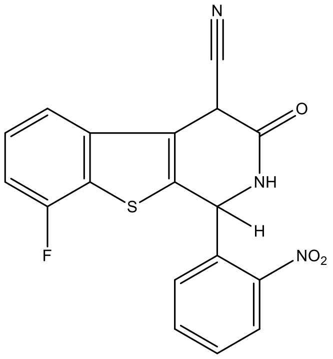

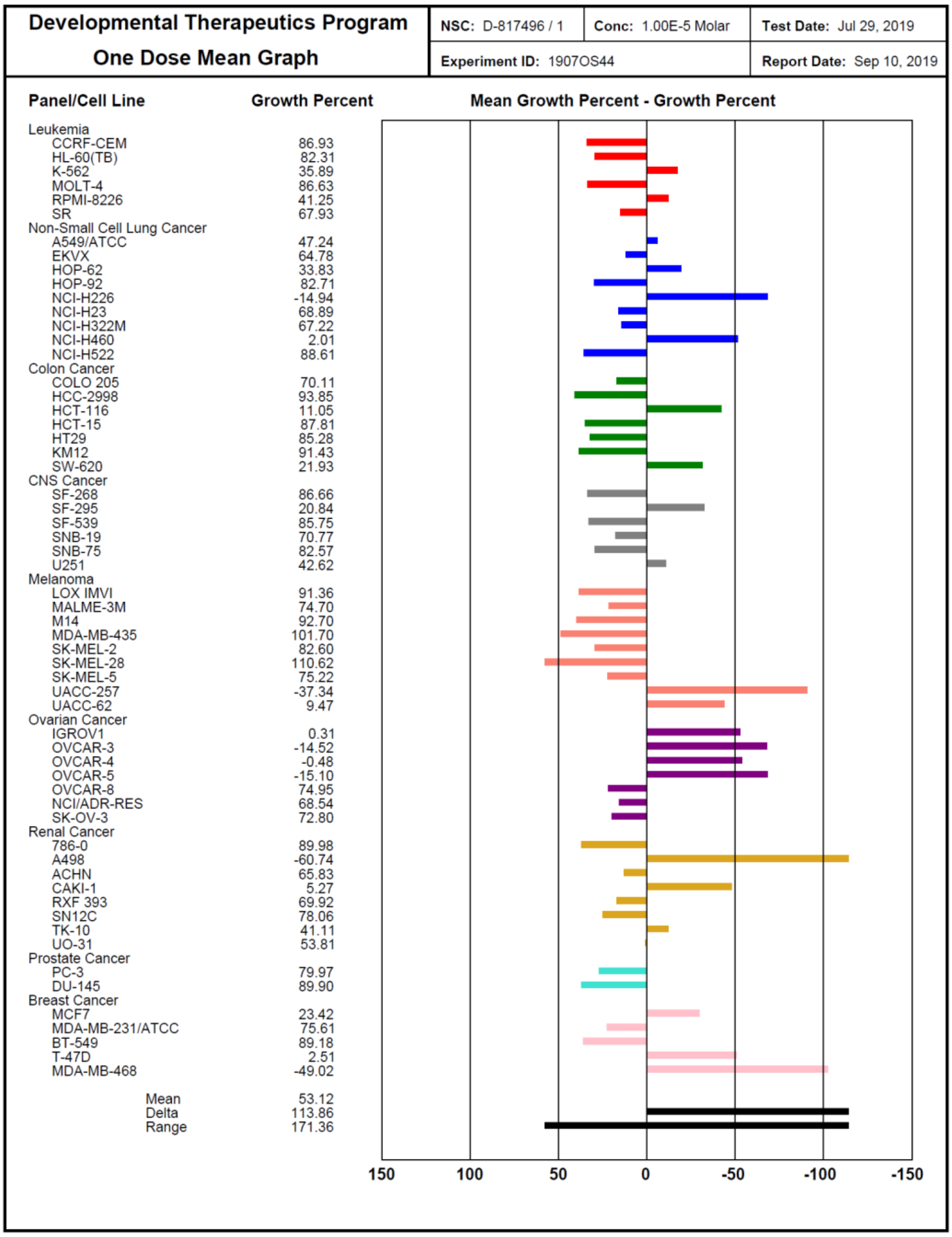

Figure S59: One-dose growth (%) and mean graph of compound **5c.**

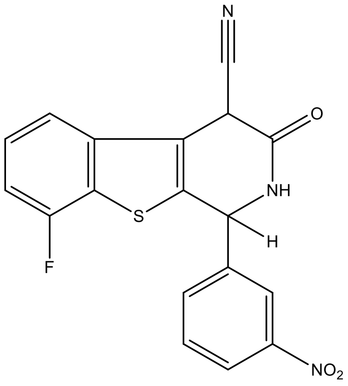

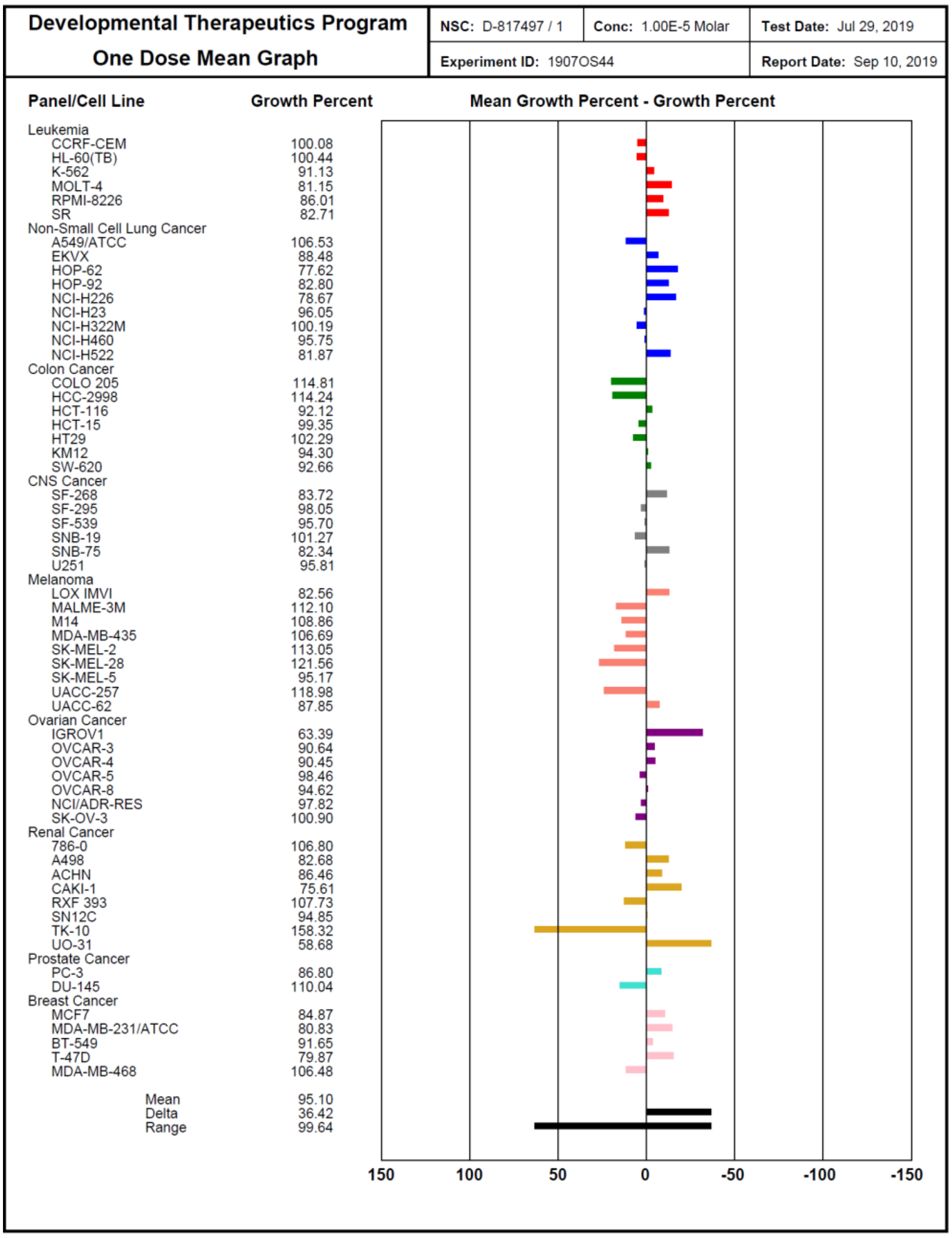

Figure S60: One-dose growth (%) and mean graph of compound **5d.**

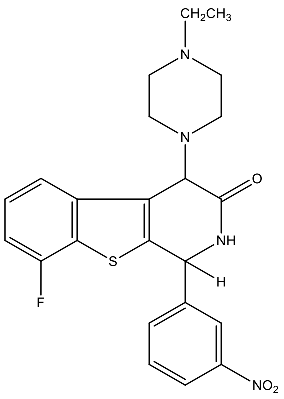

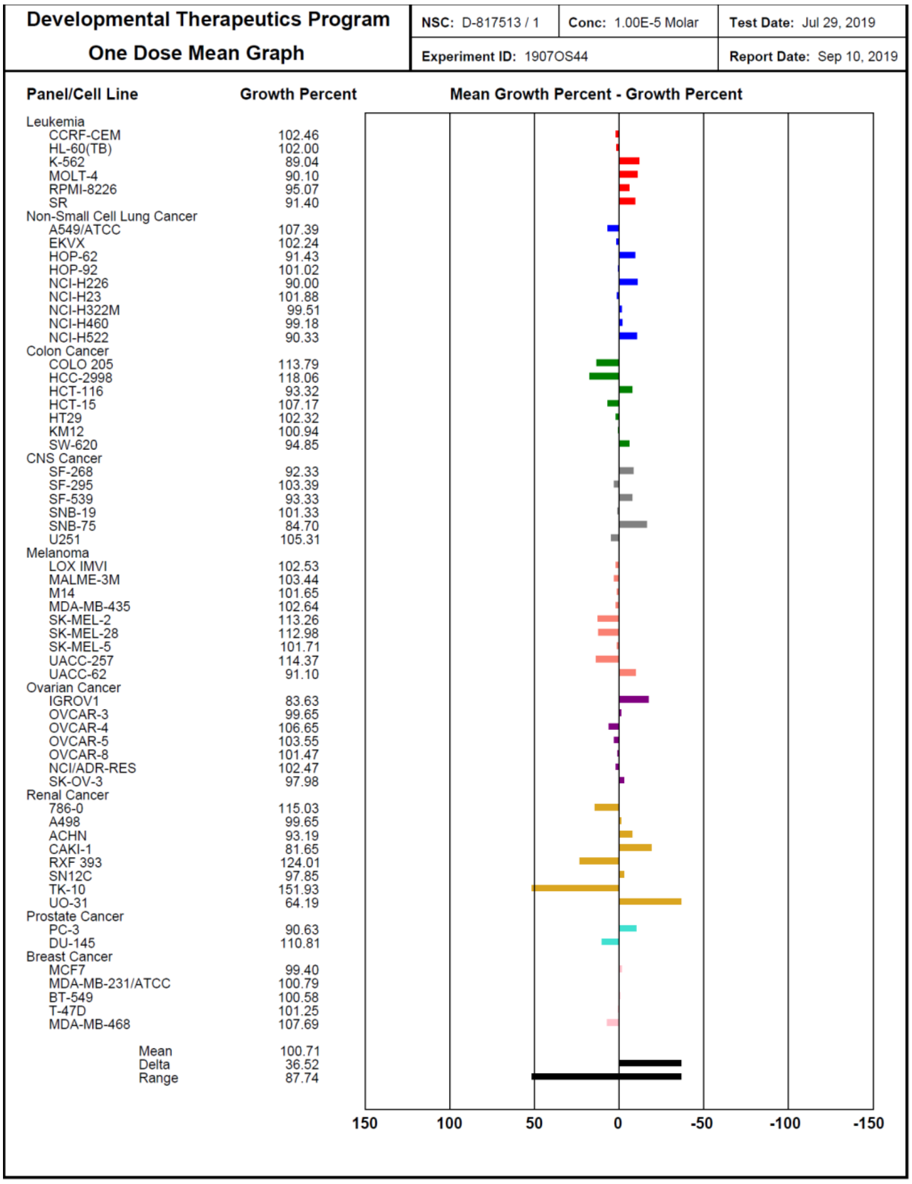

Figure S61: One-dose growth (%) and mean graph of compound **6a.**

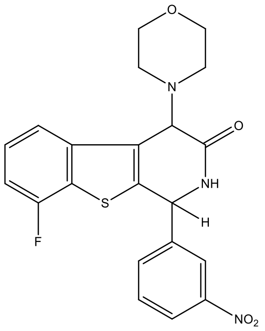

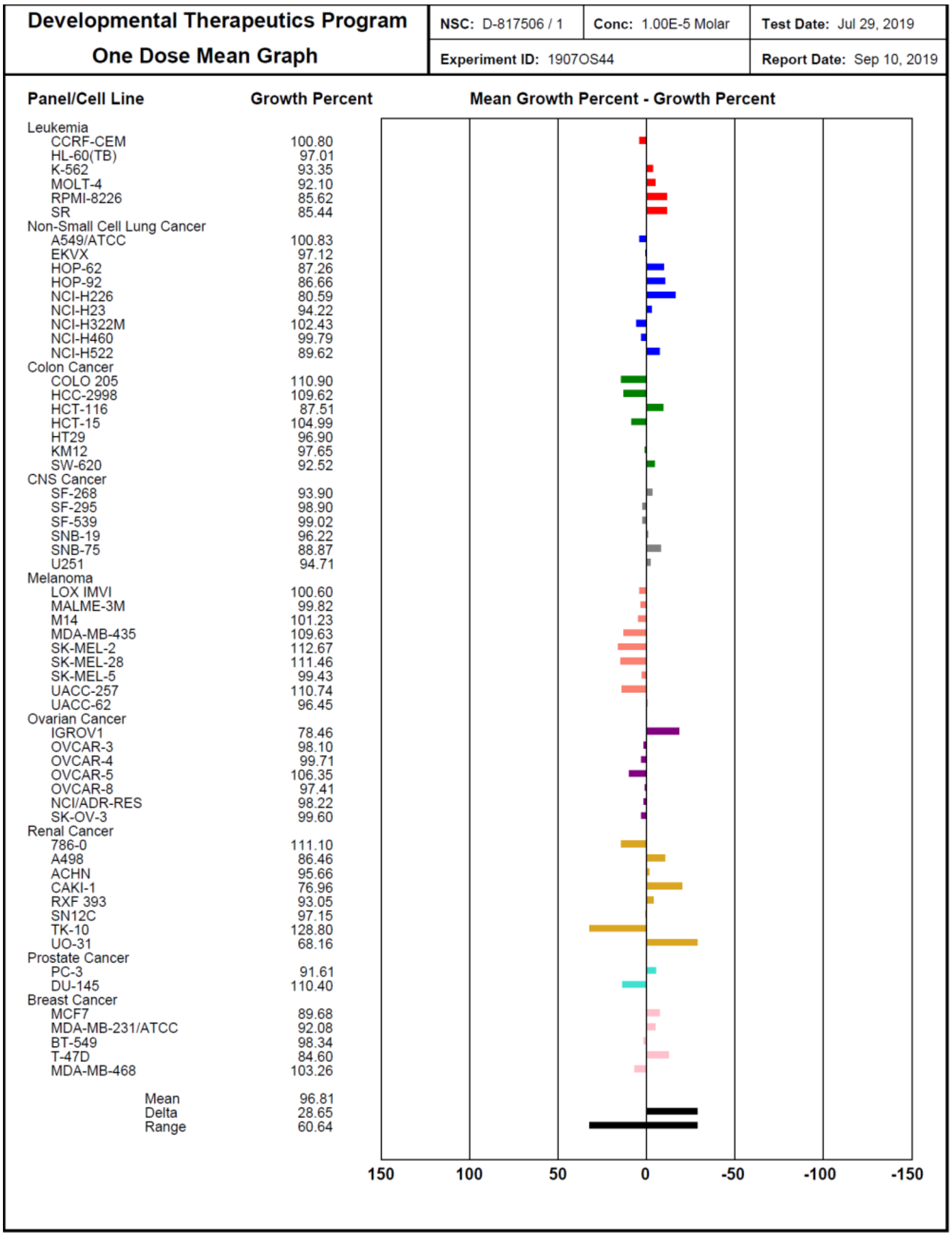

Figure S62: One-dose growth (%) and mean graph of compound **6b.**

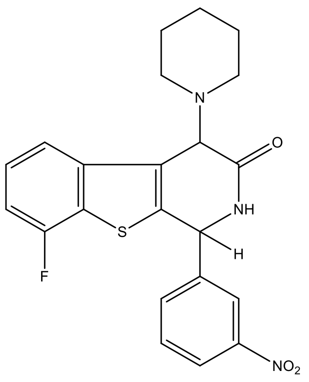

Developmental Therapeutics Program	NSC: D-817507 / 1	Conc: 1.00E-5 Molar	Test Date: Jul 29, 2019
One Dose Mean Graph	Experiment ID: 1907OS44		Report Date: Sep 10, 2019

Panel/Cell Line	Growth Percent
Leukemia	
CCRF-CEM	98.16
HL-60(TB)	116.98
K-562	93.99
MOLT-4	91.09
RPMI-8226	94.85
SR	90.70
Non-Small Cell Lung Cancer	
A549/ATCC	100.33
EKVX	94.14
HOP-62	88.47
HOP-92	85.90
NCI-H226	91.29
NCI-H23	94.84
NCI-H322M	97.41
NCI-H460	100.58
NCI-H522	85.97
Colon Cancer	
COLO 205	112.72
HCC-2998	97.18
HCT-116	79.44
HCT-15	96.26
HT29	91.00
KM12	97.09
SW-620	92.54
CNS Cancer	
SF-268	97.76
SF-295	97.72
SF-539	98.23
SNB-19	100.90
SNB-75	92.46
U251	94.74
Melanoma	
LOX IMVI	97.51
MALME-3M	101.85
M14	98.10
MDA-MB-435	108.64
SK-MEL-2	108.06
SK-MEL-28	112.43
SK-MEL-5	98.85
UACC-257	106.77
UACC-62	95.15
Ovarian Cancer	
IGROV1	79.31
OVCAR-3	102.74
OVCAR-4	104.39
OVCAR-5	101.56
OVCAR-8	95.01
NCI/ADR-RES	101.61
SK-OV-3	100.84
Renal Cancer	
786-0	106.38
A498	92.66
ACHN	90.68
CAKI-1	82.35
RXF 393	93.84
SN12C	97.55
TK-10	142.18
UO-31	67.92
Prostate Cancer	
PC-3	91.63
DU-145	109.90
Breast Cancer	
MCF7	89.81
MDA-MB-231/ATCC	99.72
BT-549	98.39
T-47D	91.98
MDA-MB-468	109.31
Mean	97.46
Delta	29.54
Range	74.26

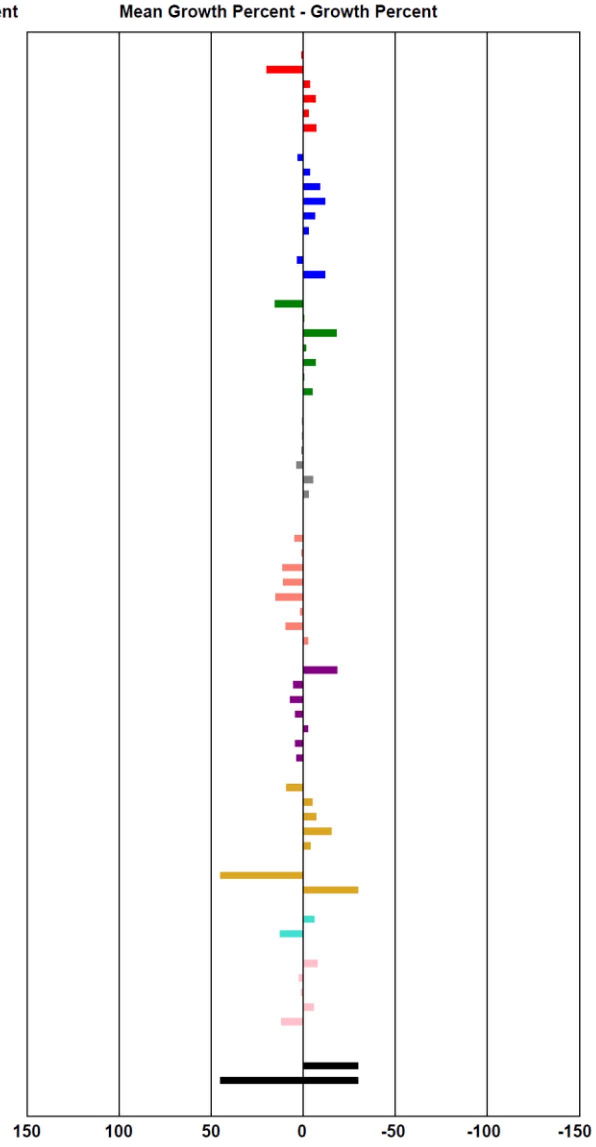

Figure S63: One-dose growth (%) and mean graph of compound **6c.**

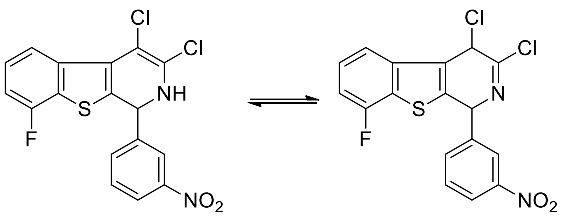

Developmental Therapeutics Program One Dose Mean Graph	NSC: D-817508 / 1	Conc: 1.00E-5 Molar	Test Date: Jul 29, 2019
	Experiment ID: 1907OS44		Report Date: Sep 10, 2019

Panel/Cell Line	Growth Percent
**Leukemia**	
CCRF-CEM	97.88
HL-60(TB)	108.33
K-562	82.06
MOLT-4	84.96
RPMI-8226	88.50
SR	83.84
**Non-Small Cell Lung Cancer**	
A549/ATCC	97.71
EKVX	92.90
HOP-62	84.50
HOP-92	85.30
NCI-H226	85.84
NCI-H23	91.45
NCI-H322M	102.48
NCI-H460	98.76
NCI-H522	87.31
**Colon Cancer**	
COLO 205	114.59
HCC-2998	116.11
HCT-116	86.62
HCT-15	101.63
HT29	101.97
KM12	96.75
SW-620	95.92
**CNS Cancer**	
SF-268	94.84
SF-295	102.06
SF-539	97.74
SNB-19	98.28
SNB-75	90.16
U251	92.92
**Melanoma**	
LOX IMVI	97.76
MALME-3M	102.96
M14	95.05
MDA-MB-435	109.15
SK-MEL-2	105.95
SK-MEL-28	111.40
SK-MEL-5	98.06
UACC-257	107.18
UACC-62	92.82
**Ovarian Cancer**	
IGROV1	89.31
OVCAR-3	103.96
OVCAR-4	96.84
OVCAR-5	97.79
OVCAR-8	98.10
NCI/ADR-RES	102.76
SK-OV-3	94.05
**Renal Cancer**	
786-0	107.16
A498	96.64
ACHN	93.04
CAKI-1	81.16
RXF 393	92.19
SN12C	97.46
TK-10	137.25
UO-31	69.84
**Prostate Cancer**	
PC-3	96.99
DU-145	104.75
**Breast Cancer**	
MCF7	94.96
MDA-MB-231/ATCC	91.91
BT-549	99.33
T-47D	82.26
MDA-MB-468	104.71
Mean	96.85
Delta	27.01
Range	67.41

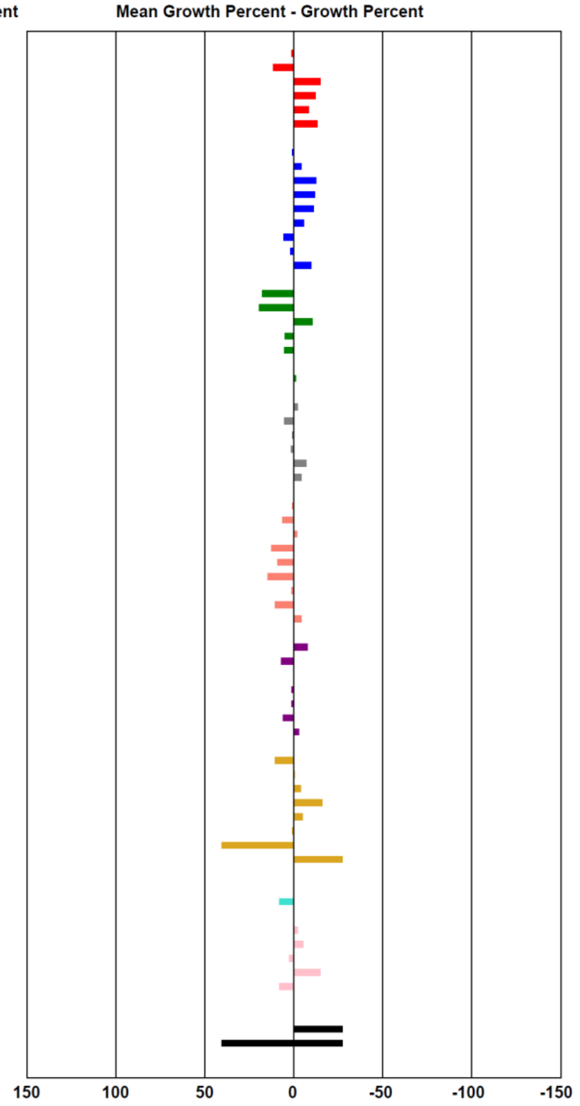

Figure S64: One-dose growth (%) and mean graph of compound **7a.**

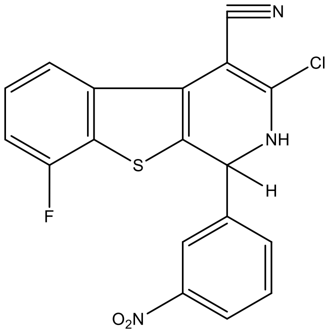

Developmental Therapeutics Program | **NSC:** D-817512 / 1 | **Conc:** 1.00E-5 Molar | **Test Date:** Jul 29, 2019

One Dose Mean Graph | **Experiment ID:** 1907OS44 | **Report Date:** Sep 10, 2019

Panel/Cell Line	Growth Percent
**Leukemia**	
CCRF-CEM	100.37
HL-60(TB)	115.57
K-562	95.99
MOLT-4	98.68
RPMI-8226	91.38
SR	88.21
**Non-Small Cell Lung Cancer**	
A549/ATCC	102.72
EKVX	87.52
HOP-62	83.99
HOP-92	81.12
NCI-H226	84.28
NCI-H23	96.10
NCI-H322M	98.15
NCI-H460	101.12
NCI-H522	95.32
**Colon Cancer**	
COLO 205	105.97
HCC-2998	112.24
HCT-116	98.69
HCT-15	106.34
HT29	104.51
KM12	100.49
SW-620	96.57
**CNS Cancer**	
SF-268	95.13
SF-295	101.19
SF-539	93.89
SNB-19	101.27
SNB-75	91.51
U251	99.10
**Melanoma**	
LOX IMVI	95.23
MALME-3M	107.54
M14	95.35
MDA-MB-435	107.84
SK-MEL-2	110.67
SK-MEL-28	115.82
SK-MEL-5	98.23
UACC-257	123.69
UACC-62	93.19
**Ovarian Cancer**	
IGROV1	76.34
OVCAR-3	102.65
OVCAR-4	100.52
OVCAR-5	104.10
OVCAR-8	101.56
NCI/ADR-RES	104.88
SK-OV-3	88.54
**Renal Cancer**	
786-0	109.68
A498	87.89
ACHN	90.72
CAKI-1	74.77
RXF 393	103.67
SN12C	98.05
TK-10	136.96
UO-31	66.52
**Prostate Cancer**	
PC-3	90.90
DU-145	111.15
**Breast Cancer**	
MCF7	90.56
MDA-MB-231/ATCC	80.23
BT-549	98.49
T-47D	77.74
MDA-MB-468	102.69
Mean	97.86
Delta	31.34
Range	70.44

Mean Growth Percent - Growth Percent

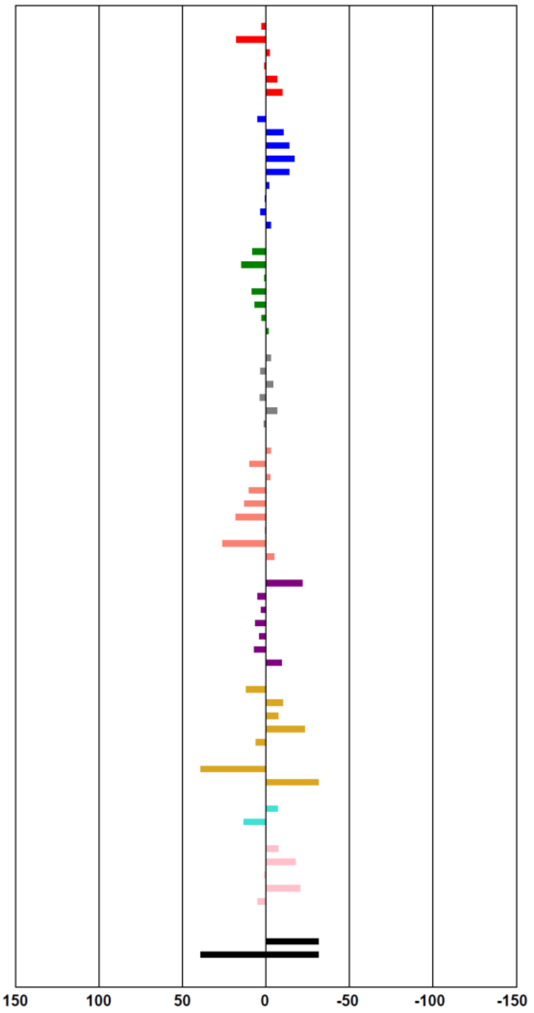

Figure S65: One-dose growth (%) and mean graph of compound **7b.**

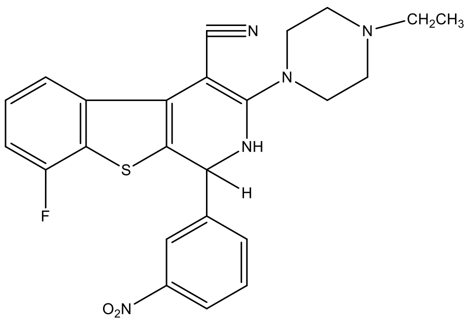

Developmental Therapeutics Program	NSC: D-817513 / 1	Conc: 1.00E-5 Molar	Test Date: Jul 29, 2019
One Dose Mean Graph	Experiment ID: 1907OS44		Report Date: Sep 10, 2019

Panel/Cell Line	Growth Percent
**Leukemia**	
CCRF-CEM	102.46
HL-60(TB)	102.00
K-562	89.04
MOLT-4	90.10
RPMI-8226	95.07
SR	91.40
**Non-Small Cell Lung Cancer**	
A549/ATCC	107.39
EKVX	102.24
HOP-62	91.43
HOP-92	101.02
NCI-H226	90.00
NCI-H23	101.88
NCI-H322M	99.51
NCI-H460	99.18
NCI-H522	90.33
**Colon Cancer**	
COLO 205	113.79
HCC-2998	118.06
HCT-116	93.32
HCT-15	107.17
HT29	102.32
KM12	100.94
SW-620	94.85
**CNS Cancer**	
SF-268	92.33
SF-295	103.39
SF-539	93.33
SNB-19	101.33
SNB-75	84.70
U251	105.31
**Melanoma**	
LOX IMVI	102.53
MALME-3M	103.44
M14	101.65
MDA-MB-435	102.64
SK-MEL-2	113.26
SK-MEL-28	112.98
SK-MEL-5	101.71
UACC-257	114.37
UACC-62	91.10
**Ovarian Cancer**	
IGROV1	83.63
OVCAR-3	99.65
OVCAR-4	106.65
OVCAR-5	103.55
OVCAR-8	101.47
NCI/ADR-RES	102.47
SK-OV-3	97.98
**Renal Cancer**	
786-0	115.03
A498	99.65
ACHN	93.19
CAKI-1	81.65
RXF 393	124.01
SN12C	97.85
TK-10	151.93
UO-31	64.19
**Prostate Cancer**	
PC-3	90.63
DU-145	110.81
**Breast Cancer**	
MCF7	99.40
MDA-MB-231/ATCC	100.79
BT-549	100.58
T-47D	101.25
MDA-MB-468	107.69
Mean	100.71
Delta	36.52
Range	87.74

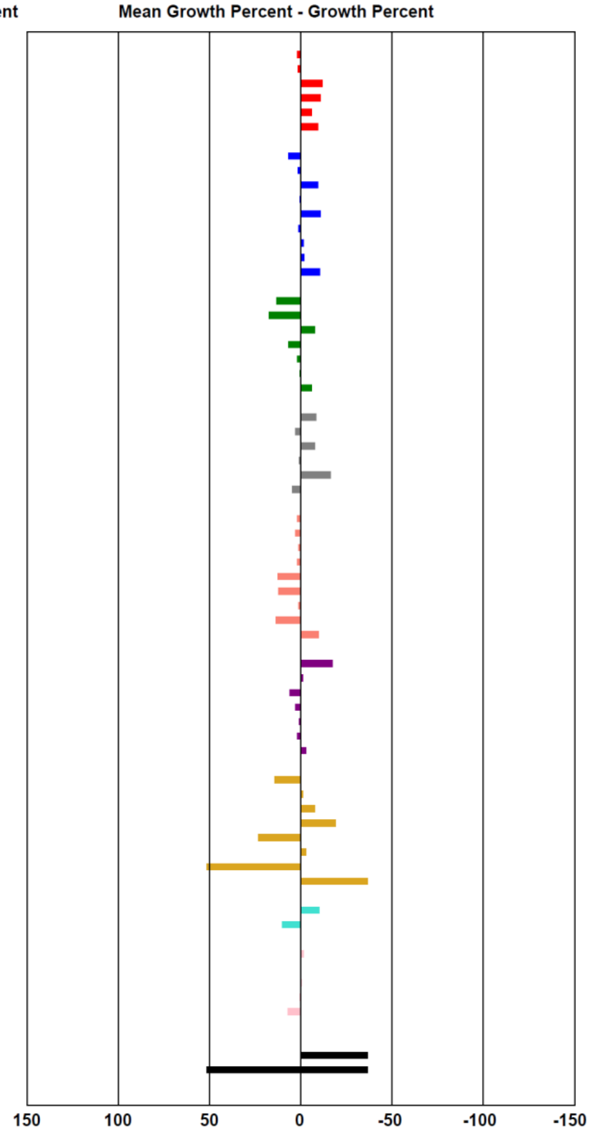

Figure S66: One-dose growth (%) and mean graph of compound **8a.**

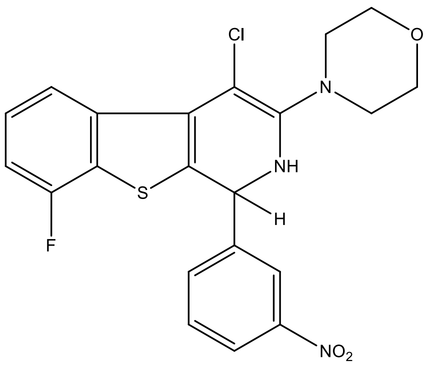

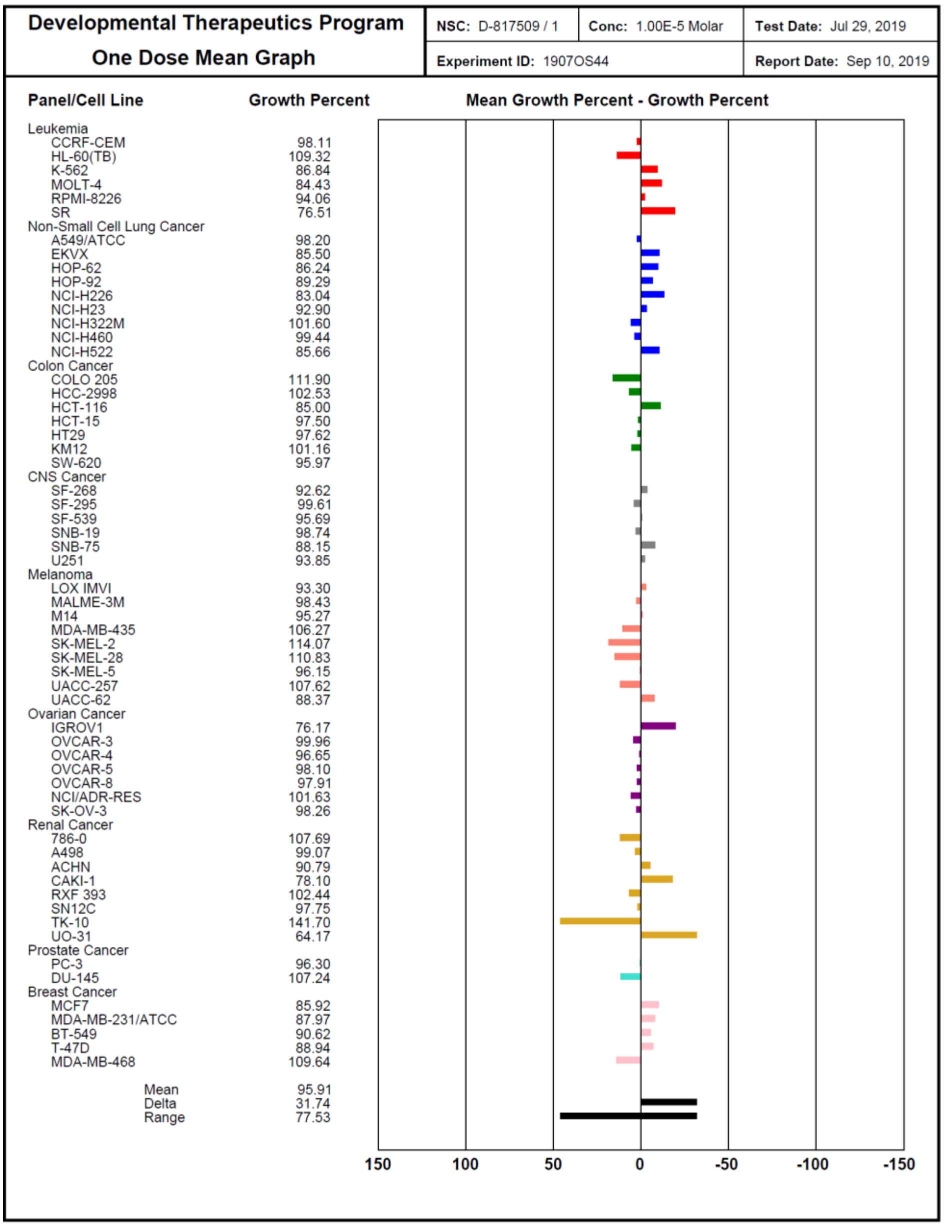

Figure S67: One-dose growth (%) and mean graph of compound **8b.**

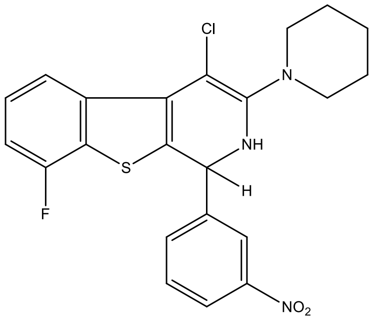

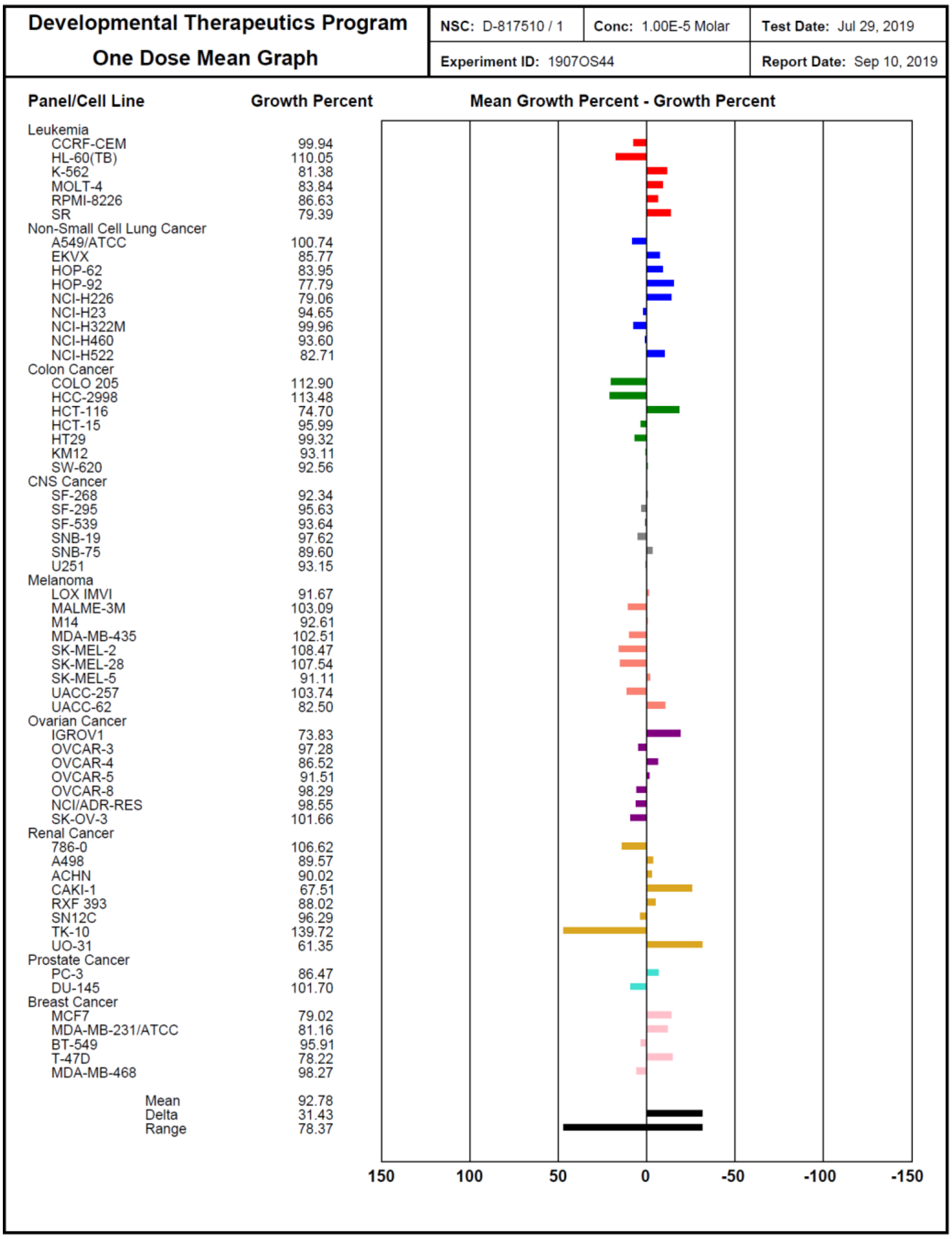

Figure S68: One-dose growth (%) and mean graph of compound **8c.**

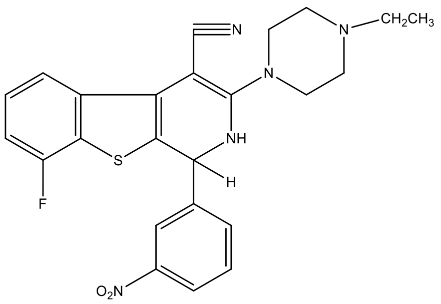

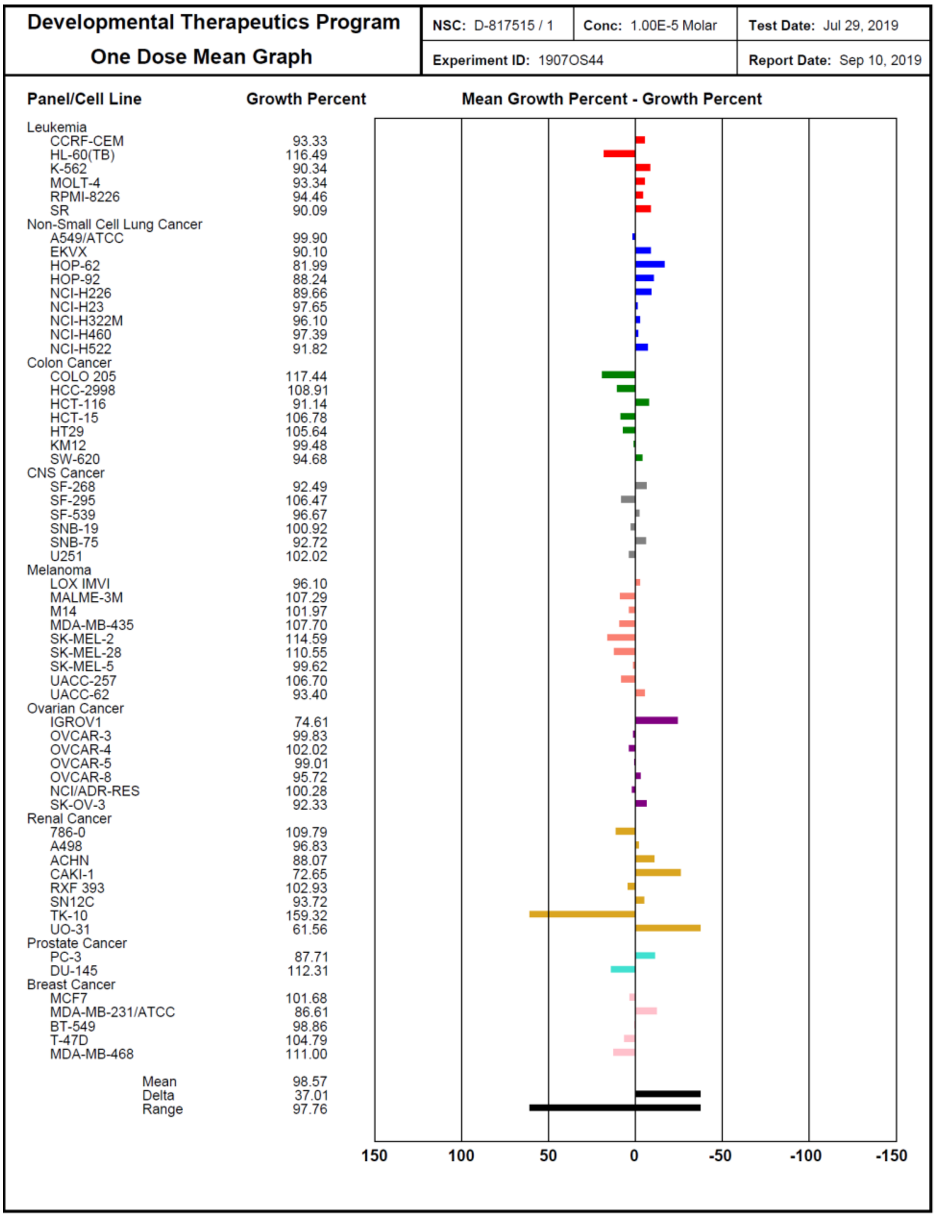

Figure S69: One-dose growth (%) and mean graph of compound **8d.**

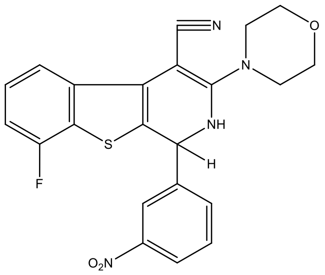

Developmental Therapeutics Program	NSC: D-817516 / 1	Conc: 1.00E-5 Molar	Test Date: Jul 29, 2019
One Dose Mean Graph	Experiment ID: 1907OS44		Report Date: Sep 10, 2019

Panel/Cell Line	Growth Percent
Leukemia	
CCRF-CEM	96.68
HL-60(TB)	103.35
K-562	76.91
MOLT-4	85.06
RPMI-8226	94.69
SR	88.91
Non-Small Cell Lung Cancer	
A549/ATCC	102.65
EKVX	94.49
HOP-62	89.79
HOP-92	95.10
NCI-H226	84.36
NCI-H23	95.91
NCI-H322M	105.64
NCI-H460	97.80
NCI-H522	90.46
Colon Cancer	
COLO 205	116.69
HCC-2998	123.24
HCT-116	95.50
HCT-15	104.78
HT29	104.42
KM12	94.87
SW-620	95.89
CNS Cancer	
SF-268	90.66
SF-295	109.62
SF-539	93.51
SNB-19	98.62
SNB-75	94.55
U251	106.11
Melanoma	
LOX IMVI	97.39
MALME-3M	110.60
M14	99.42
MDA-MB-435	106.93
SK-MEL-2	114.83
SK-MEL-28	110.68
SK-MEL-5	97.29
UACC-257	112.71
UACC-62	84.53
Ovarian Cancer	
IGROV1	85.05
OVCAR-3	99.91
OVCAR-4	99.91
OVCAR-5	94.81
OVCAR-8	100.33
NCI/ADR-RES	101.61
SK-OV-3	92.88
Renal Cancer	
786-0	112.07
A498	100.87
ACHN	85.71
CAKI-1	72.30
RXF 393	106.31
SN12C	96.32
TK-10	153.09
UO-31	66.82
Prostate Cancer	
PC-3	92.13
DU-145	108.32
Breast Cancer	
MCF7	100.95
MDA-MB-231/ATCC	84.95
BT-549	98.97
T-47D	97.55
MDA-MB-468	108.03
Mean	98.70
Delta	31.88
Range	86.27

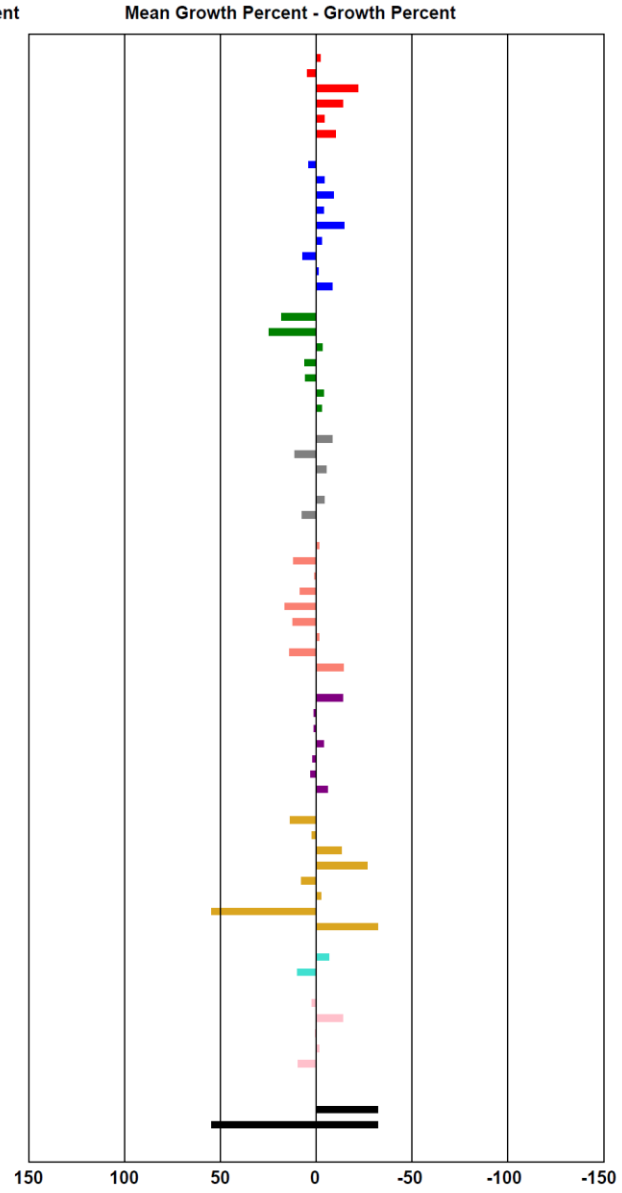

Figure S70: One-dose growth (%) and mean graph of compound **8e.**

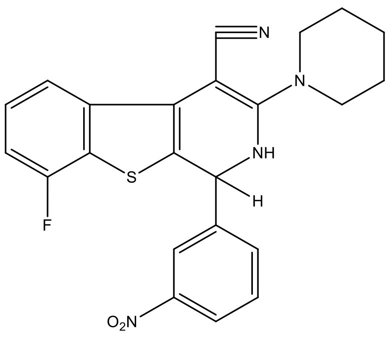

Developmental Therapeutics Program

One Dose Mean Graph

NSC: D-817517 / 1	Conc: 1.00E-5 Molar	Test Date: Jul 29, 2019
Experiment ID: 1907OS44		Report Date: Sep 10, 2019

Panel/Cell Line	Growth Percent
Leukemia	
CCRF-CEM	101.14
HL-60(TB)	103.65
K-562	83.35
MOLT-4	87.99
RPMI-8226	101.99
SR	83.48
Non-Small Cell Lung Cancer	
A549/ATCC	102.98
EKVX	95.18
HOP-62	90.55
HOP-92	101.53
NCI-H226	89.56
NCI-H23	99.88
NCI-H322M	101.56
NCI-H460	98.84
NCI-H522	95.37
Colon Cancer	
COLO 205	117.10
HCC-2998	117.51
HCT-116	101.05
HCT-15	104.08
HT29	105.27
KM12	100.46
SW-620	98.00
CNS Cancer	
SF-268	92.94
SF-295	107.10
SF-539	96.07
SNB-19	100.03
SNB-75	89.78
U251	103.59
Melanoma	
LOX IMVI	96.87
MALME-3M	107.56
M14	98.67
MDA-MB-435	107.00
SK-MEL-2	122.04
SK-MEL-28	107.98
SK-MEL-5	96.13
UACC-257	115.24
UACC-62	88.52
Ovarian Cancer	
IGROV1	75.88
OVCAR-3	103.93
OVCAR-4	102.74
OVCAR-5	96.85
OVCAR-8	103.40
NCI/ADR-RES	106.50
SK-OV-3	101.70
Renal Cancer	
786-0	110.32
A498	100.12
ACHN	84.53
CAKI-1	71.71
RXF 393	115.26
SN12C	98.29
TK-10	154.74
UO-31	63.94
Prostate Cancer	
PC-3	92.43
DU-145	111.41
Breast Cancer	
MCF7	98.09
MDA-MB-231/ATCC	82.24
BT-549	94.98
T-47D	105.91
MDA-MB-468	110.57
Mean	99.92
Delta	35.98
Range	90.80

Mean Growth Percent - Growth Percent

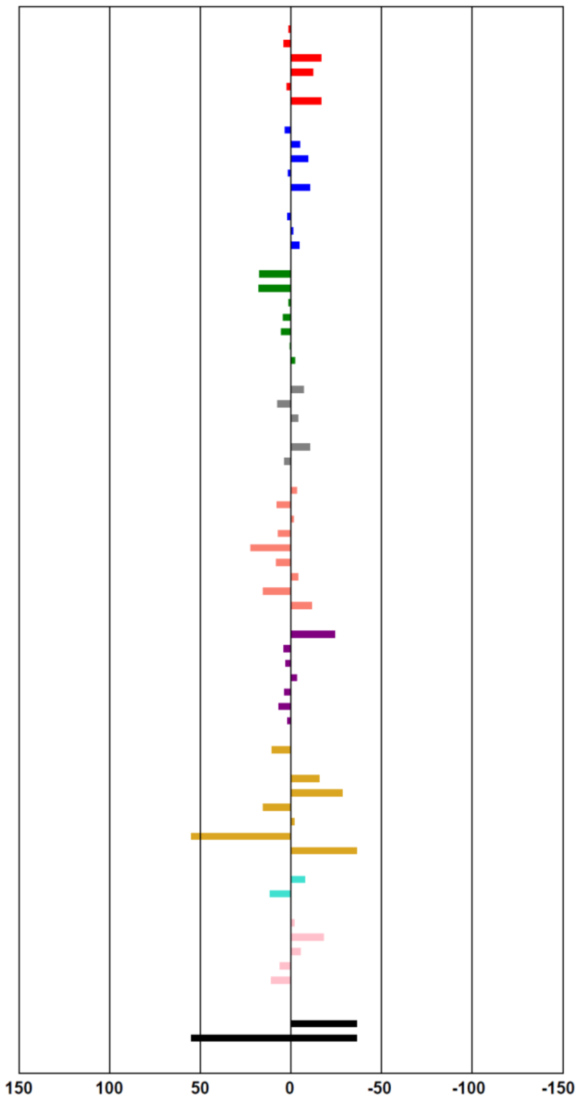

Figure S71: One-dose growth (%) and mean graph of compound **8f.**

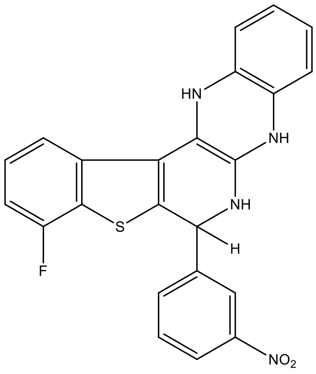

**Developmental Therapeutics Program**	**NSC:** D-817511 / 1	**Conc:** 1.00E-5 Molar	**Test Date:** Jul 29, 2019
**One Dose Mean Graph**	**Experiment ID:** 1907OS44		**Report Date:** Sep 10, 2019

Panel/Cell Line	Growth Percent
Leukemia	
CCRF-CEM	68.69
HL-60(TB)	81.30
K-562	85.89
MOLT-4	91.26
RPMI-8226	64.42
SR	89.94
Non-Small Cell Lung Cancer	
A549/ATCC	94.92
EKVX	80.87
HOP-62	94.88
HOP-92	90.73
NCI-H226	71.88
NCI-H23	96.58
NCI-H322M	100.59
NCI-H460	97.01
NCI-H522	82.29
Colon Cancer	
COLO 205	112.73
HCC-2998	106.85
HCT-116	98.05
HCT-15	96.52
HT29	102.62
KM12	96.59
SW-620	98.31
CNS Cancer	
SF-268	96.60
SF-295	99.02
SF-539	89.60
SNB-19	100.02
SNB-75	80.61
U251	96.11
Melanoma	
LOX IMVI	93.72
MALME-3M	99.86
M14	93.54
MDA-MB-435	106.42
SK-MEL-2	99.64
SK-MEL-28	110.12
SK-MEL-5	90.44
UACC-257	103.22
UACC-62	86.68
Ovarian Cancer	
IGROV1	68.11
OVCAR-3	95.44
OVCAR-4	94.29
OVCAR-5	105.09
OVCAR-8	97.09
NCI/ADR-RES	98.74
SK-OV-3	97.25
Renal Cancer	
786-0	111.82
A498	94.38
ACHN	80.45
CAKI-1	71.31
RXF 393	103.38
SN12C	94.34
TK-10	133.58
UO-31	62.31
Prostate Cancer	
PC-3	95.64
DU-145	106.34
Breast Cancer	
MCF7	59.44
MDA-MB-231/ATCC	81.50
BT-549	87.76
T-47D	78.02
MDA-MB-468	70.80
Mean	92.13
Delta	32.69
Range	74.14

Mean Growth Percent - Growth Percent

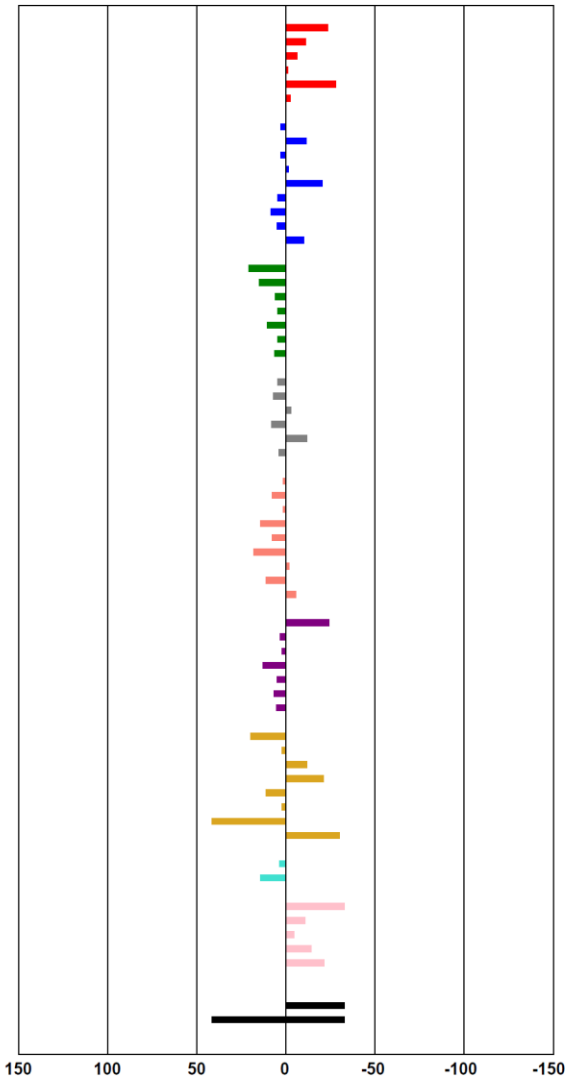

Figure S72: One-dose growth (%) and mean graph of compound **9.**

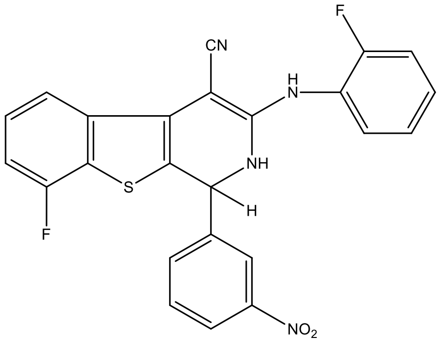

Developmental Therapeutics Program
One Dose Mean Graph

NSC: D-817520 / 1	Conc: 1.00E-5 Molar	Test Date: Jul 29, 2019
Experiment ID: 1907OS44		Report Date: Sep 10, 2019

Panel/Cell Line	Growth Percent
Leukemia	
CCRF-CEM	97.04
HL-60(TB)	113.14
K-562	97.28
MOLT-4	98.54
RPMI-8226	93.42
SR	86.97
Non-Small Cell Lung Cancer	
A549/ATCC	107.45
EKVX	90.73
HOP-62	91.73
HOP-92	90.40
NCI-H226	87.16
NCI-H23	101.96
NCI-H322M	100.11
NCI-H460	101.10
NCI-H522	102.13
Colon Cancer	
COLO 205	113.32
HCC-2998	120.34
HCT-116	96.56
HCT-15	105.46
HT29	114.25
KM12	99.86
SW-620	97.55
CNS Cancer	
SF-268	95.30
SF-295	104.19
SF-539	93.96
SNB-19	103.45
SNB-75	89.89
U251	109.90
Melanoma	
LOX IMVI	96.74
MALME-3M	115.06
M14	98.32
MDA-MB-435	104.45
SK-MEL-2	119.64
SK-MEL-28	110.97
SK-MEL-5	99.34
UACC-257	123.17
UACC-62	93.25
Ovarian Cancer	
IGROV1	83.91
OVCAR-3	101.64
OVCAR-4	103.90
OVCAR-5	103.49
OVCAR-8	106.58
NCI/ADR-RES	108.27
SK-OV-3	89.87
Renal Cancer	
786-0	112.31
A498	96.06
ACHN	90.03
CAKI-1	78.89
RXF 393	105.32
SN12C	99.51
TK-10	155.35
UO-31	68.77
Prostate Cancer	
PC-3	92.21
DU-145	108.61
Breast Cancer	
MCF7	95.08
MDA-MB-231/ATCC	82.60
BT-549	98.96
T-47D	102.94
MDA-MB-468	107.35
Mean	100.95
Delta	32.18
Range	86.58

Mean Growth Percent - Growth Percent

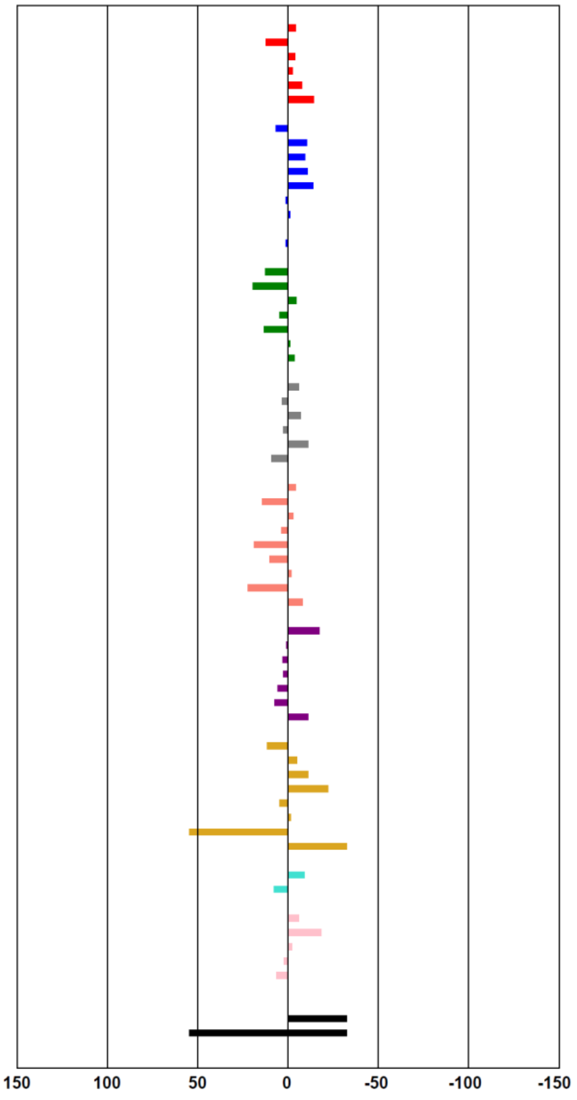

Figure S73: One-dose growth (%) and mean graph of compound **10.**

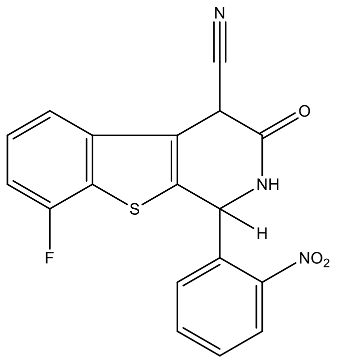

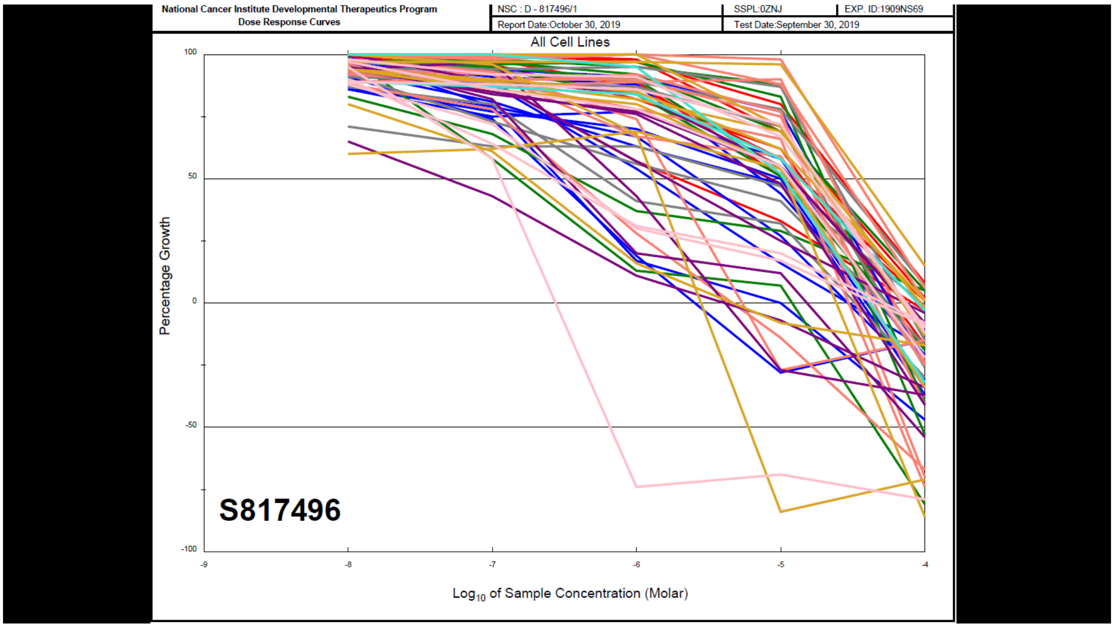

Figure S74: *In vitro* NCI dose response Curve of compound **5c** on all cancer cell lines

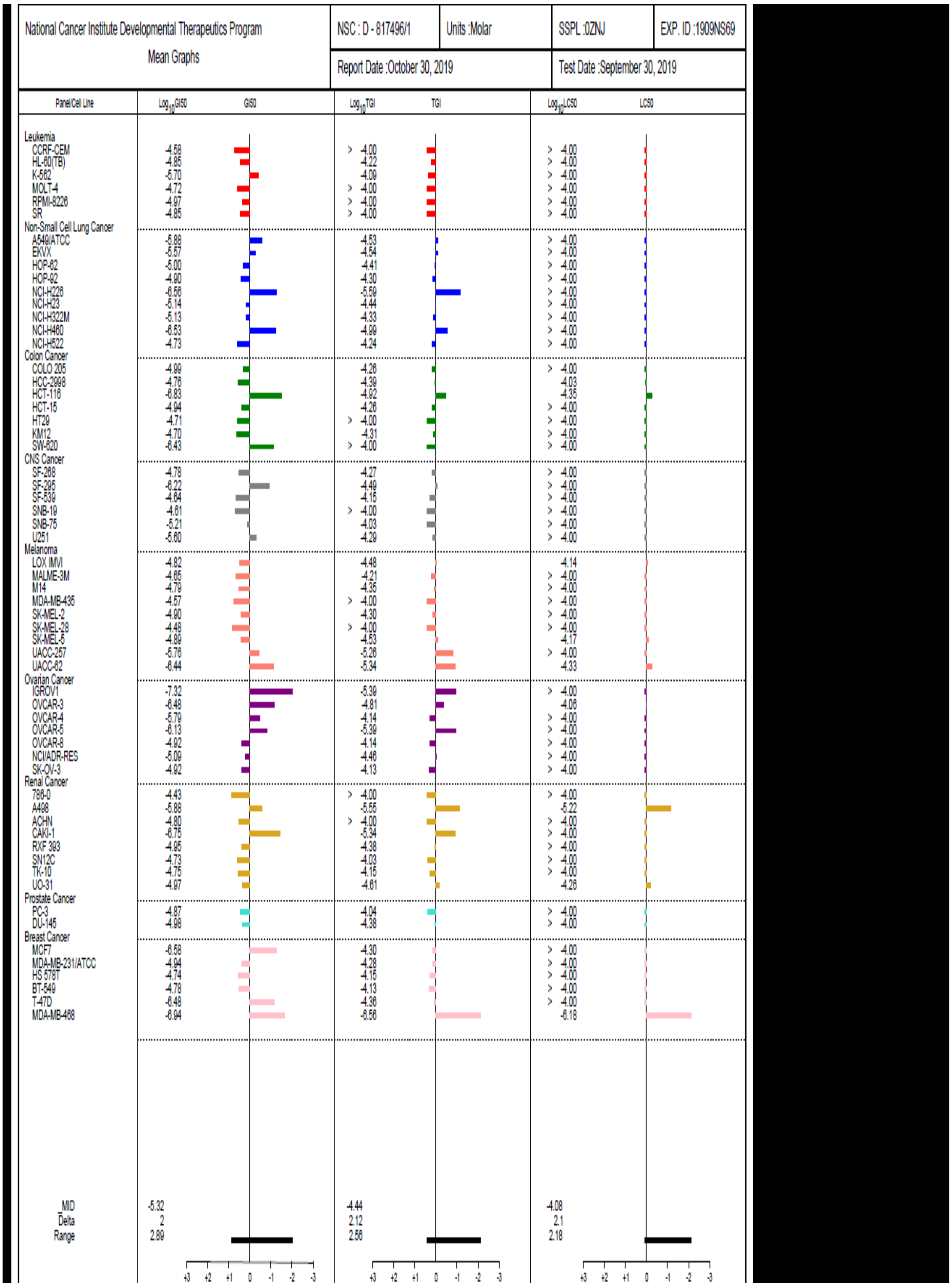

Figure S75: NCI Mean graph of compound **5c** on different cancer cell lines

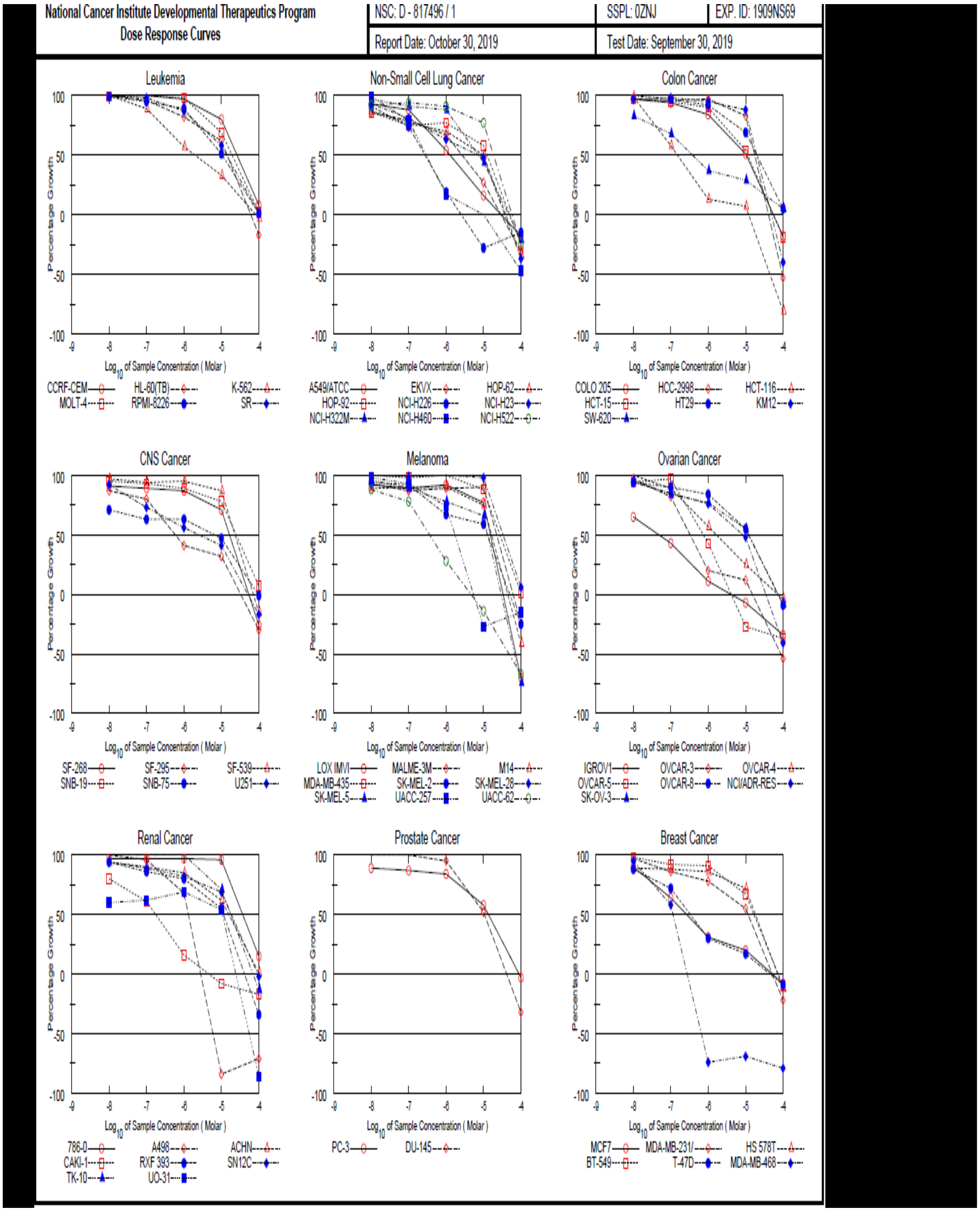

Figure S76: *In vitro* NCI dose response Curves of compound **5c** on different cancer cell lines

National Cancer Institute Developmental Therapeutics Program
In-Vitro Testing Results

NSC : D - 817496 / 1	Experiment ID : 1909NS69	Test Type : 08	Units : Molar
Report Date : October 30, 2019	Test Date : September 30, 2019	QNS :	MC :
COMI : 32	Stain Reagent : SRB Dual-Pass Related	SSPL : 0ZNJ	

Log10 Concentration

Panel/Cell Line	Time Zero	Ctrl	Mean Optical Densities					Percent Growth					GI50	TGI	LC50
			-8.0	-7.0	-6.0	-5.0	-4.0	-8.0	-7.0	-6.0	-5.0	-4.0			
**Leukemia**															
CCRF-CEM	0.406	2.254	2.256	2.271	2.192	1.887	0.562	100	101	97	80	8	2.63E-5	> 1.00E-4	> 1.00E-4
HL-60(TB)	0.776	2.878	2.902	2.785	2.494	2.084	0.642	101	96	82	62	-17	1.42E-5	6.06E-5	> 1.00E-4
K-562	0.208	2.312	2.371	2.088	1.413	0.899	0.202	103	89	57	33	-3	1.98E-6	8.19E-5	> 1.00E-4
MOLT-4	0.645	2.978	2.953	2.974	2.937	2.255	0.683	99	100	98	69	2	1.91E-5	> 1.00E-4	> 1.00E-4
RPMI-8226	1.008	2.959	2.935	2.857	2.743	2.010	1.036	99	95	89	51	1	1.06E-5	> 1.00E-4	> 1.00E-4
SR	0.552	2.476	2.486	2.430	2.219	1.675	0.578	101	98	87	58	1	1.40E-5	> 1.00E-4	> 1.00E-4
**Non-Small Cell Lung Cancer**															
A549/ATCC	0.399	2.462	2.318	2.217	1.523	0.726	0.329	93	88	54	16	-18	1.31E-6	2.98E-5	> 1.00E-4
EKVX	0.661	1.780	1.627	1.544	1.415	0.962	0.453	86	79	67	27	-31	2.69E-6	2.89E-5	> 1.00E-4
HOP-62	0.928	2.899	2.639	2.440	2.307	1.916	0.609	87	77	70	50	-34	1.00E-5	3.92E-5	> 1.00E-4
HOP-92	1.279	2.069	1.956	1.870	1.886	1.741	0.956	86	75	77	58	-25	1.26E-5	4.99E-5	> 1.00E-4
NCI-H226	0.933	2.629	2.479	2.187	1.256	0.674	0.789	91	74	19	-28	-15	2.73E-7	2.55E-6	> 1.00E-4
NCI-H23	0.602	2.102	2.002	1.823	1.551	1.321	0.377	93	81	63	48	-37	7.33E-6	3.65E-5	> 1.00E-4
NCI-H322M	0.838	2.419	2.371	2.280	2.227	1.537	0.660	97	91	88	44	-21	7.37E-6	4.73E-5	> 1.00E-4
NCI-H460	0.346	3.236	3.195	2.620	0.850	0.359	0.185	99	79	17	.	-47	2.94E-7	1.02E-5	> 1.00E-4
NCI-H522	1.161	3.115	2.950	3.004	2.936	2.661	0.880	92	94	91	77	-24	1.84E-5	5.76E-5	> 1.00E-4
**Colon Cancer**															
COLO 205	0.542	2.615	2.555	2.485	2.274	1.598	0.447	97	94	84	51	-18	1.03E-5	5.53E-5	> 1.00E-4
HCC-2998	1.084	3.287	3.219	3.202	3.222	2.906	0.505	97	96	97	83	-53	1.74E-5	4.05E-5	9.44E-5
HCT-116	0.246	2.467	2.441	1.528	0.524	0.409	0.047	99	58	13	7	-81	1.48E-7	1.21E-5	4.45E-5
HCT-15	0.345	2.355	2.296	2.252	2.156	1.436	0.279	97	95	90	54	-19	1.14E-5	5.49E-5	> 1.00E-4
HT29	0.361	2.717	2.708	2.653	2.540	1.978	0.475	100	97	92	69	5	1.96E-5	> 1.00E-4	> 1.00E-4
KM12	0.604	3.128	3.059	3.077	2.994	2.833	0.363	97	98	95	88	-40	1.99E-5	4.88E-5	> 1.00E-4
SW-620	0.344	2.482	2.112	1.795	1.128	0.956	0.451	83	68	37	29	5	3.74E-7	> 1.00E-4	> 1.00E-4
**CNS Cancer**															
SF-268	1.236	2.757	2.614	2.590	2.559	2.317	0.912	91	89	87	71	-26	1.64E-5	5.37E-5	> 1.00E-4
SF-295	0.602	2.163	1.966	1.849	1.250	1.096	0.421	87	80	41	32	-30	6.00E-7	3.26E-5	> 1.00E-4
SF-539	0.902	2.576	2.528	2.484	2.485	2.357	0.765	97	94	95	87	-15	2.30E-5	7.10E-5	> 1.00E-4
SNB-19	0.571	2.154	2.076	2.042	1.980	1.801	0.681	95	93	89	78	7	2.46E-5	> 1.00E-4	> 1.00E-4
SNB-75	0.736	1.368	1.184	1.137	1.133	1.030	0.727	71	63	63	47	-1	6.12E-6	9.40E-5	> 1.00E-4
U251	0.579	2.717	2.548	2.141	1.774	1.457	0.481	92	73	56	41	-17	2.50E-6	5.09E-5	> 1.00E-4
**Melanoma**															
LOX IMVI	0.389	3.022	2.804	2.722	2.813	2.421	0.117	92	89	92	77	-70	1.53E-5	3.35E-5	7.32E-5
MALME-3M	0.673	1.113	1.084	1.056	1.063	1.070	0.513	93	87	89	90	-24	2.26E-5	6.19E-5	> 1.00E-4
M14	0.464	2.145	1.965	1.983	1.976	1.719	0.275	89	90	90	75	-41	1.63E-5	4.43E-5	> 1.00E-4
MDA-MB-435	0.600	2.955	2.954	2.911	2.976	2.670	0.616	100	98	101	88	1	2.72E-5	> 1.00E-4	> 1.00E-4
SK-MEL-2	1.052	2.779	2.673	2.641	2.204	2.065	0.785	94	92	67	59	-25	1.27E-5	4.98E-5	> 1.00E-4
SK-MEL-28	0.845	2.383	2.413	2.360	2.382	2.352	0.931	102	99	100	98	6	3.31E-5	> 1.00E-4	> 1.00E-4
SK-MEL-5	0.558	3.272	3.174	2.971	2.687	2.343	0.147	96	89	78	66	-74	1.30E-5	2.96E-5	6.76E-5
UACC-257	1.678	3.177	3.145	3.074	2.787	1.233	1.434	98	93	74	-27	-15	1.73E-6	5.44E-6	> 1.00E-4
UACC-62	1.118	2.895	2.688	2.509	1.608	0.962	0.364	88	78	28	-14	-67	3.61E-7	4.61E-6	4.72E-5
**Ovarian Cancer**															
IGROV1	0.407	1.733	1.275	0.972	0.550	0.380	0.268	65	43	11	-7	-34	4.74E-8	4.12E-6	> 1.00E-4
OVCAR-3	0.564	1.995	1.948	1.739	0.848	0.742	0.261	97	82	20	12	-54	3.28E-7	1.54E-5	8.78E-5
OVCAR-4	0.680	1.625	1.642	1.524	1.215	0.916	0.652	102	89	57	25	-4	1.61E-6	7.21E-5	> 1.00E-4
OVCAR-5	0.732	1.613	1.559	1.590	1.108	0.536	0.463	94	97	43	-27	-37	7.35E-7	4.12E-6	> 1.00E-4
OVCAR-8	0.528	2.596	2.468	2.384	2.270	1.665	0.483	94	90	84	55	-9	1.20E-5	7.32E-5	> 1.00E-4
NCI/ADR-RES	0.507	2.109	2.012	1.863	1.721	1.270	0.300	94	85	76	48	-41	8.21E-6	3.45E-5	> 1.00E-4
SK-OV-3	0.766	1.884	1.827	1.708	1.627	1.383	0.702	95	84	77	55	-8	1.20E-5	7.37E-5	> 1.00E-4
**Renal Cancer**															
786-0	1.047	3.074	3.022	3.015	3.015	2.997	1.351	97	97	97	96	15	3.70E-5	> 1.00E-4	> 1.00E-4
A498	1.847	2.866	2.872	2.823	2.545	0.295	0.528	101	96	68	-84	-71	1.32E-6	2.81E-6	5.98E-6
ACHN	0.483	1.944	1.852	1.793	1.720	1.388	0.503	94	90	85	62	1	1.57E-5	> 1.00E-4	> 1.00E-4
CAKI-1	0.844	2.695	2.332	1.981	1.133	0.775	0.704	80	61	16	-8	-17	1.78E-7	4.53E-6	> 1.00E-4
RXF 393	0.804	1.740	1.680	1.611	1.549	1.318	0.529	94	86	80	55	-34	1.13E-5	4.13E-5	> 1.00E-4
SN12C	0.815	2.975	2.842	2.741	2.592	2.314	0.796	94	89	82	69	-2	1.86E-5	9.28E-5	> 1.00E-4
TK-10	1.037	2.043	2.181	2.333	2.229	1.747	0.907	114	129	118	71	-13	1.77E-5	7.07E-5	> 1.00E-4
UO-31	0.665	2.078	1.513	1.545	1.636	1.431	0.092	60	62	69	54	-86	1.07E-5	2.43E-5	5.52E-5
**Prostate Cancer**															
PC-3	0.624	2.668	2.447	2.412	2.335	1.810	0.608	89	87	84	58	-3	1.36E-5	9.05E-5	> 1.00E-4
DU-145	0.490	2.050	2.180	2.078	1.971	1.299	0.335	108	102	95	52	-32	1.05E-5	4.18E-5	> 1.00E-4
**Breast Cancer**															
MCF7	0.439	2.486	2.290	1.749	1.068	0.844	0.403	90	64	31	20	-8	2.63E-7	5.06E-5	> 1.00E-4
MDA-MB-231/ATCC	0.715	1.876	1.847	1.712	1.616	1.353	0.561	97	86	78	55	-22	1.16E-5	5.23E-5	> 1.00E-4
HS 578T	1.509	2.568	2.452	2.438	2.420	2.268	1.323	89	88	86	72	-12	1.81E-5	7.13E-5	> 1.00E-4
BT-549	1.178	2.386	2.365	2.290	2.280	1.982	1.064	98	92	91	67	-10	1.65E-5	7.47E-5	> 1.00E-4
T-47D	0.885	2.223	2.058	1.847	1.280	1.107	0.804	88	72	30	17	-9	3.29E-7	4.40E-5	> 1.00E-4
MDA-MB-468	0.811	2.107	2.042	1.560	0.210	0.254	0.167	95	58	-74	-69	-79	1.15E-7	2.74E-7	6.57E-7

Figure 77: NCI *in vitro* testing results of compound **5c** on different cancer cell lines.

Table S1. Growth inhibition (%) obtained from a single dose (10^{-5} M) of the tested compounds **2a,c, 3a-d** and **4a-c.**

**Cell line**	**Compound**								
	**2a**	**2c**	**3a**	**3b**	**3c**	**3d**	**4a**	**4b**	**4c**
Leukemia									
**CCRF-CEM**	-5.14	26.28	-5.88	-9.35	-10.16	0.90	-1.02	-0.42	-0.04
**HL-60 (TB)**	10.97	28.75	-7.79	-7.38	-15.87	12.00	0.73	-5.26	-5.53
**K-562 86.10**	4.76	67.82	12.84	33.50	2.86	15.78	12.46	31.32	33.50
**MOLT-4**	4.10	19.80	2.64	0.27	-5.89	2.90	4.69	7.88	11.85
**RPMI-8226**	-8.46	30.57	-2.54	1.11	4.62	29.04	11.74	7.32	5.90
**SR**	6.30	37.25	28.66	30.47	0.60	23.81	36.64	39.32	0.20
Non Small Cell Lung Carcinoma									
**A549/ATCC**	-7.82	4.91	-10.00	58.07	-5.43	-5.27	-4.25	55.45	3.15
**EKVX**	-5.71	15.63	7.10	23.00	15.84	7.98	2.22	22.96	11.80
**HOP-62**	5.99	20.37	11.63	8.26	8.52	14.32	9.42	17.16	3.32
**HOP-92**	-8.14	-11.40	2.88	5.43	7.78	10.96	9.64	16.44	-11.25
**NCI-H226**	0.31	23.54	13.11	9.99	20.72	21.32	13.57	7.14	18.47
**NCI-H23**	-4.04	13.26	0.15	-3.36	1.41	1.04	1.72	6.41	6.95
**NCI-H322M**	0.25	1.98	-3.18	16.25	1.00	2.78	-4.72	22.02	-4.23
**NCI-H460**	-6.08	59.24	2.47	36.93	23.13	0.53	-1.09	31.42	17.18
**NCI-H522**	6.14	2.94	4.48	11.40	-1.98	17.55	8.58	21.72	7.95
Colon cancer									
**COLO 205**	-15.99	-6.61	-16.49	-17.67	-12.74	-7.27	-16.67	-20.46	-21.65
**HCC-2998**	-12.75	-11.43	-15.27	-12.11	-18.72	-6.11	-7.97	-6.39	-27.42
**HCT-116**	-3.66	80.50	-0.50	-0.50	14.07	23.07	-2.40	1.62	11.44
**HCT-15**	-9.73	-1.05	-3.42	8.32	-10.00	21.33	-9.27	14.20	-1.51
**HT-29**	-9.18	-7.17	-7.86	-10.51	-12.09	0.03	-10.48	-6.98	-9.93
**KM12**	-5.55	3.27	7.40	4.33	-0.49	3.93	3.86	7.60	-0.66
**SW-620**	-4.67	68.81	3.98	4.97	20.70	2.40	-0.16	6.49	2.43
CNS Cancer									
**SF-268**	4.51	5.43	8.45	1.85	6.16	14.60	9.64	14.28	10.54
**SF-295**	-4.69	27.03	-2.52	2.74	-0.58	2.30	-2.33	1.82	-7.26
**SF-539**	-0.64	3.65	11.49	12.66	10.42	6.60	3.36	15.83	3.36
**SNB-19**	-2.10	14.24	1.20	7.57	-0.33	6.23	0.33	7.47	-2.16
**SNB-75**	12.62	-1.55	17.27	25.44	10.03	21.53	22.30	22.84	11.66
**U251**	-7.91	32.54	2.07	13.61	14.88	20.57	0.40	21.06	8.82
Melanoma									
**LOX IMVI**	-0.02	11.09	5.88	2.21	2.71	12.77	4.84	6.46	8.72
**MALME-3M**	4.19	12.55	15.80	11.07	5.53	-6.36	9.21	10.78	9.46
**M14**	-5.61	-1.11	4.96	6.88	-0.72	1.35	3.25	3.86	1.70
**MDA-MB435**	-0.27	-12.11	9.40	31.80	-7.97	-3.38	8.55	33.66	-7.17
**SK-MEL-2**	-17.93	-9.11	-25.13	-12.61	-25.53	-1.12	-23.16	-18.30	-16.71
**SK-MEL-28**	-7.93	-4.83	-11.36	1.50	-20.20	-9.19	-1.74	-0.07	8.45
**SK-MEL-5**	-2.91	8.44	2.83	1.99	2.72	5.55	-1.74	-0.07	8.45
**UACC-275**	-15.16	78.82	-6.31	-12.46	-19.98	-7.53	-12.81	-6.58	-0.18
**UACC-62**	7.33	60.94	10.62	11.65	12.83	14.07	10.87	8.74	20.84
Ovarian Cancer									
**IGROV1**	0.46	66.51	8.40	10.25	34.55	20.45	10.33	4.86	32.96
**OVCAR-3**	-7.34	70.82	-0.33	-2.13	2.18	0.09	1.99	0.61	7.25
**OVCAR-4**	-6.44	40.46	3.35	-0.19	7.00	-1.20	-1.63	7.51	11.14
**OVCAR-5**	-9.42	43/78	-3.20	-8.59	-8.05	-8.70	-3.64	-12.95	-2.88
**OVCAR-8**	-4.32	6.75	-3.27	-1.02	-7.33	2.86	-0.71	2.95	1.73

**NCI/ADR-RES**	-3.61	13.48	-2.37	-2.15	-5.76	4.50	-5.94	9.18	-1.98
**SK-OV-3**	6.36	2.42	-0.85	-2.91	6.94	-3.95	0.53	-2.04	-9.85
Renal Cancer									
**786-0**	-12.20	-10.98	-7.41	-8.85	-9.68	-10.00	4.93	-0.46	-16.91
**A498**	-4.03	55.09	9.33	4.22	-15.47	4.88	14.90	11.10	-6.23
**ACHN**	-4.06	7.28	1.30	-2.10	-0.56	6.70	12.41	-3.20	-4.45
**CAKI-1**	11.93	85.07	23.33	16.33	24.17	21.82	27.47	17.66	12.41
**RXF 393**	-27.86	-3.10	-2.63	-18.20	-24.83	-5.23	-0.71	9.68	-17.33
**SN 12C**	-0.73	7.57	-0.49	-0.17	0.94	4.04	9.45	3.66	4.76
**TK-10**	-34.90	-30.93	-29.42	-29.74	-41.81	-52.28	-25.46	-38.15	-51.99
**UO-31**	24.99	29.91	32.68	25.81	30.62	34.84	40.33	71.67	33.22

***NT: not tested.**

Table S2. Growth inhibition (%) obtained from a single dose (10^{-5} M) of the tested compounds **4d, 5a-d, 6a-c** and **7a.**

**Cell line**	**Compound**								
	**4d**	**5a**	**5b**	**5c**	**5d**	**6a**	**6b**	**6c**	**7a**
Leukemia									
**CCRF-CEM**	1.99	28.11	65.96	13.17	-0.08	-2.46	-0.89	1.84	2.12
**HL-60 (TB)**	-16.76	43.05	89.47	18.79	-0.44	-2.00	3.00	-16.98	-8.33
**K-562**	22.96	76.14	85.98	64.10	8.17	10.96	6.63	6.11	17.99
**MOLT-4**	9.18	28.61	50.86	13.47	18.85	9.90	7.90	8.91	15.17
**RPMI-8226**	9.61	n.t	36.89	58.75	13.99	4.93	14.38	5.15	11.50
**SR**	11.38	86.10	77.87	33.17	17.30	8.60	14.36	0.30	16.16
Non Small Cell Lung Carcinoma									
**A549/ATCC**	-2.89	41.53	58.45	52.76	-6.53	-7.34	-0.83	-0.32	2.30
**EKVX**	0.87	20.71	38.32	35.32	11.52	-2.24	2.98	5.86	7.10
**HOP-62**	1.22	42.59	54.60	66.27	21.38	8.57	12.74	11.53	15.50
**HOP-92**	5.89	57.75	45.89	114.94	17.20	-1.02	13.34	14.10	14.70
**NCI-H226**	5.26	37.80	39.82	31.22	3.95	10.00	19.41	8.71	14.16
**NCI-H23**	-1.19	35.21	24.79	32.88	-0.19	-1.88	5.78	5.16	8.55
**NCI-H322M**	-0.93	9.60	34.79	97.99	4.25	0.49	-2.43	2.60	-2.48
**NCI-H460**	1.43	68.17	71.40	11.49	18.13	0.82	0.21	-0.58	1.24
**NCI-H522**	8.55	92.03	79.90	17.29	22.38	0.67	10.38	14.03	12.70
Colon cancer									
**COLO 205**	-17.16	11.65	8.44	29.89	-14.81	-13.79	-10.90	-12.72	-14.59
**HCC-2998**	-15.95	11.73	8.46	6.16	-14.24	-18.06	-9.62	2.82	-16.11
**HCT-116**	9.54	77.24	62.45	88.95	7.78	6.68	12.49	20.56	13.38
**HCT-15**	1.31	63.64	66.48	12.29	0.05	-7.17	-4.99	3.74	-1.63
**HT-29**	2.30	35.95	49.50	14.72	-2.29	-2.32	3.10	9.00	-1.97
**KM12**	-5.21	68.99	59.95	8.58	5.70	-0.94	2.35	2.91	3.25
**SW-620**	3.43	68.68	75.68	88.07	7.44	5.15	7.48	7.46	4.18
CNS Cancer									
**SF-268**	6.94	48.93	38.96	14.44	16.38	7.67	6.10	2.64	5.16
**SF-295**	-3.10	72.38	69.50	79.16	1.95	-3.39	1.10	2.28	-2.06
Melanoma									
**LOX IMVI**	5.22	60.33	54.42	8.64	17.44	-2.53	-0.60	2.50	1.24
**MALME-3M**	4.62	60.34	52.89	25.30	-12.10	-3.44	0.18	-1.85	-2.96
**M14**	-0.99	62.20	66.71	7.30	-8.86	-1.65	-1.23	1.90	4.95
**MDA-MB435**	-7.63	-128.6	109.11	-1.70	-6.69	-2.64	-9.63	-8.63	-9.15
**SK-MEL-2**	-26.10	35.91	39.98	7.40	-13.05	-13.26	-12.67	-8.06	-5.95
**SK-MEL-28**	-10.50	45.90	47.40	-10.62	-21.56	-12.98	-11.46	-12.43	-11.40
**SK-MEL-5**	4.05	18.22	39.46	24.88	4.83	-1.71	0.57	1.15	1.94
**UACC-275**	-15.75	31.78	36.15	137.34	-18.98	-14.74	-10.74	-6.77	-7.18
**UACC-62**	10.37	58.78	64.69	90.63	12.15	8.90	3.55	4.85	7.18
Ovarian Cancer									
**IGROV1**	22.77	48.25	58.88	99.69	36.61	16.37	21.54	20.70	10.70

**OVCAR-3**	-4.91	86.28	99.38	114.48	9.36	0.35	1.90	-2.74	-3.96
**OVCAR-4**	1.96	46.31	28.44	100.46	9.55	-6.65	0.30	-4.39	3.16
**OVCAR-5**	-3.92	1.24	14.42	115.10	1.54	-3.55	-6.35	-1.56	3.21
**OVCAR-8**	-1.82	38.70	30.20	25.05	5.37	2.02	2.60	5.00	1.90
**NCI/ADR-RES**	-8.05	84.39	70.73	31.46	2.18	-2.46	1.78	-1.66	-2.76
**SK-OV-3**	-10.25	49.28	36.89	28.20	-0.90	-1.47	0.40	-0.84	5.95
Renal Cancer									
**786-0**	-11.18	31.64	29.12	10.02	-6.80	-15.03	-11.10	-6.36	-7.16
**A498**	-0.03	74.75	68.82	160.74	17.32	0.35	13.54	7.34	3.36
**ACHN**	6.28	62.51	55.49	34.27	13.54	6.81	4.34	9.32	6.96
**CAKI-1**	27.40	78.73	60.00	94.73	14.39	18.35	23.04	17.65	18.84
**RXF 393**	-18.11	90.35	44.07	30.05	-7.73	-24.01	6.95	6.16	7.81
**SN 12C**	2.95	26.28	38.64	20.94	5.16	2.15	2.85	2.45	2.54
**TK-10**	-40.69	1.90	-12.69	58.88	-58.32	-51.93	-28.16	-42.18	-37.25
**UO-31**	34.70	66.03	64.20	46.19	41.32	35.81	31.84	32.18	30.16
Prostate Cancer									
**PC-3**	3.80	43.20	50.65	20.03	13.20	9.37	10.40	8.67	3.11
**DU-145**	-8.57	9.51	35.21	10.10	-10.04	-10.81	-9.40	-9.90	-4.75
Breast Cancer									
**MCF-7**	8.44	75.53	67.39	76.38	15.13	0.60	10.32	10.19	5.05
**MDAMB231/ATCC**	5.22	27.31	28.82	24.49	19.17	-0.79	7.92	0.28	8.09
**BT-549**	3.62	78.64	44.61	10.82	8.35	-0.58	1.66	1.61	0.77
**T-47D**	8.70	38.33	19.88	97.49	20.13	-1.25	15.40	8.12	17.74
**MDA-MB-468**	-8.27	75.70	66.31	149.2	-6.48	-7.69	-3.26	-9.31	-4.71

***NT: not tested.**

Table S3. Growth inhibition (%) obtained from a single dose (10^{-5} M) of the tested compounds **7b, 8a-f, 9** and **10.**

**Cell line**	**Compound**								
	**7b**	**8a**	**8b**	**8c**	**8d**	**8e**	**8f**	**9**	**10**
Leukemia									
**CCRF-CEM**	3.22	1.89	5.00	30.31	-0.37	6.67	3.32	-1.14	2.96
**HL-60(TB)**	1.47	-9.32	-10.05	18.70	-15.57	-16.49	-3.35	-3.65	-13.14
**K-562**	8.80	13.16	18.62	14.11	4.11	9.66	23.09	16.65	2.72
**RPMI-8226**	12.71	15.37	16.16	8.74	1.72	6.66	14.95	12.11	1.46
**MOLT-4**	7.93	5.94	13.57	35.58	8.62	5.54	5.31	-1.99	6.48
**SR**	15.93	23.49	20.41	10.06	11.80	9.91	11.09	16.52	13.03
Non Small Cell Lung Carcinoma									
**A549/ATCC**	-10.05	1.80	-0.74	5.05	-2.72	0.10	-2.65	-2.98	-7.45
**EKVX**	-1.36	4.50	14.33	19.13	12.48	9.90	5.51	4.82	9.27
**HOP-62**	9.67	3.76	16.05	5.22	16.11	18.01	10.21	9.45	8.27
**HOP-92**	10.38	10.71	22.19	9.27	18.88	11.76	4.90	-1.53	9.60
**NCI-H226**	9.35	16.96	20.94	28.22	15.72	10.44	15.67	10.44	12.84
**NCI-H23**	4.25	7.10	5.35	3.42	3.90	2.35	4.09	0.22	-1.96
**NCI-H322M**	-3.17	-1.60	0.04	-0.59	1.85	3.90	-5.64	-1.56	-0.11
**NCI-H460**	2.14	0.60	6.40	3.00	-1.12	2.61	2.20	1.16	-1.10
**NCI-H522**	-1.38	14.34	17.30	17.71	4.68	8.18	9.54	4.63	-2.13
Colon cancer									
**COLO 205**	-14.62	-11.90	-12.90	-12.73	-5.97	-17.44	-16.69	-17.10	-13.32
**HCC-2998**	-18.20	-2.53	-13.48	-6.85	-12.24	-8.91	-23.24	-17.51	-20.34
**HCT-116**	11.73	15.00	25.30	1.95	1.31	8.86	4.50	-1.05	3.44
**HCT-15**	-7.25	2.50	4.11	3.84	-6.34	-6.78	-4.78	-4.08	-5.46
**HT-29**	-3.29	2.30	0.78	-2.62	-4.51	-5.64	-4.42	-5.27	-14.25
**KM12**	-0.12	-1.16	6.99	3.41	-0.49	0.52	5.13	-0.46	0.14
**SW-620**	3.78	4.03	7.44	1.71	3.43	5.32	4.11	2.00	2.45
CNS Cancer									
SF-268	6.82	7.38	7.66	6.19	4.87	7.51	9.44	7.06	4.70
**SF-295**	-4.69	0.40	4.37	-6.60	-1.19	-6.46	-9.62	-7.10	-4.19
**SF-539**	3.78	4.31	6.36	-6.63	6.11	3.33	6.49	3.93	6.04
**SNB-19**	-0.67	1.26	1.40	-3.36	-1.27	-0.92	1.38	-0.03	-3.45
**SNB-75**	8.28	11.85	10.40	22.37	8.49	7.28	5.45	10.22	10.11
**U251**	-7.36	6.16	6.85	-7.81	0.90	-2.20	-6.11	-3.59	-9.90
Melanoma									
**LOX IMVI**	1.46	6.70	8.33	-0.70	4.73	3.90	2.61	3.13	3.26
**MALME-3M**	-2.31	1.57	-3.09	4.61	-7.54	-7.29	-10.60	-7.56	-15.06
**M14**	-5.17	6.73	7.49	1.39	4.65	-1.97	0.58	1.33	1.68
**MDA-MB435**	-8.18	-6.27	-2.51	2.00	-7.84	-7.70	-6.93	-7.00	-4.45
**SK-MEL-2**	-2.07	3.85	8.89	2.66	1.77	0.38	2.71	3.87	0.67
**SK-MEL-28**	-11.22	-10.83	-7.54	-16.73	-15.82	-10.55	-10.68	-7.98	-10.97
**SK-MEL-5**	-20.08	-14.07	-8.74	0.27	-10.67	-14.59	-14.83	-22.04	-19.64
**UACC-257**	-9.77	-7.62	-3.74	-10.30	-23.69	-6.70	-12.71	-15.24	-23.17
**UACC-62**	8.84	11.63	17.50	8.57	6.81	6.70	15.47	11.48	6.75

Ovarian Cancer									
**IGROV1**	15.63	23.83	16.17	17.36	23.66	25.41	14.95	24.22	16.09
**OVCAR-3**	-1.67	1.03	2.72	-1.68	-2.65	0.17	0.09	-3.93	-1.64
**OVCAR-4**	-3.81	3.25	13.48	12.27	-0.52	-2.02	0.90	-2.74	-3.90
**OVCAR-5**	-3.54	1.90	8.49	-1.61	-4.10	0.99	5.19	3.15	-3.49
**OVCAR-8**	-4.49	2.09	1.71	-6.18	-1.56	4.28	-0.33	-3.40	-6.58
**NCI/ADR-RES**	1.73	-1.63	1.45	-2.62	-4.88	-0.28	-1.61	-6.50	-8.27
**SK-OV-3**	2.24	1.74	-1.60	9.90	11.46	7.67	7.22	-1.70	10.13
Renal Cancer									
**786-0**	-13.52	-7.31	-6.62	-0.24	-9.68	-9.79	-12.07	-10.32	-12.31
**A498**	19.72	0.93	10.43	N.T	12.11	3.17	-0.87	-0.12	3.94
**ACHN**	4.44	9.21	9.98	6.20	9.28	11.93	14.30	15.47	9.97
**CAKI-1**	19.82	21.90	32.49	18.80	25.23	27.35	28.70	28.29	21.11
**RXF 393**	-15.77	-2.44	11.89	-15.60	-3.67	-2.93	-6.31	-15.26	-5.32
**SN 12C**	4.43	2.25	3.71	3.66	1.95	6.28	3.70	1.71	0.49
**TK-10**	-53.79	-41.70	-39.72	-22.35	-36.96	-59.32	-53.09	-54.74	-55.35
**UO-31**	32.54	35.83	38.65	37.95	33.48	38.44	33.28	36.06	31.23
Prostate Cancer									
**PC-3**	3.50	7.70	14.53	11.89	9.10	12.29	8.87	7.47	7.80
**DU-145**	-14.25	-7.24	-1.71	-12.51	-11.15	-12.31	-8.32	-11.41	-8.61
Breast Cancer									
**MCF-7**	8.22	4.08	20.98	23.32	9.44	-1.68	-0.95	1.91	4.92
**MDA-MB 231/ATCC**	7.25	12.03	18.84	13.93	19.77	13.40	15.05	17.76	17.40
**BT-549**	-2.93	9.38	4.09	-10.51	1.51	1.14	1.03	5.02	1.04
**T-47D**	-9.14	11.06	20.78	17.49	22.26	-4.79	2.45	-5.91	-2.94
**MDA-MB-468**	-7.25	-9.64	1.73	-13.89	-2.69	-11.00	-8.03	-10.57	-7.35

Table S4. Mean growth inhibition (%) for the tested compounds **2a,c, 3a-d, 4a-d, 5a-d, 6a-c, 7a,b 8a-f, 9** and **10**

Compound	Mean growth inhibition (%)	Compound	Mean growth inhibition (%)
**2a**	-3.74	**6a**	-0.71
**2c**	11.47	**6b**	3.19
**3a**	6.67	**6c**	2.54
**3b**	6.02	**7a**	3.15
**3c**	1.33	**7b**	2.14
**3d**	6.24	**8a**	-0.20
**4a**	1.85	**8b**	6.10
**4b**	8.66	**8c**	7.22
**4c**	3.00	**8d**	1.33
**4d**	0.15	**8e**	1.30
**5a**	52.52	**8f**	0.008
**5b**	52.47	**9**	7.87
**5c**	46.88	**10**	-.095
**5d**	5.00		

Table S5. Effect of compound **5c** and abiraterone on CYP17 enzyme.

Sample code	CYP17 enzyme Mice testis Aver. conc. nM
**5c**	15.80± 0.20
**abiraterone reference**	10.72± 0.17
**Control before treatment**	35.50± 1.25
**Control after treatment**	32.90± 0.38

Table S6. Inhibitory activity of compound **5c** and abiraterone reference against plasma testosterone level.

Sample code	Testosterone Mice testes Aver. conc. nM
**5c**	12.26± 0.17
**abiraterone reference**	13.79± 0.33
**Control before treatment**	30.45± 0.57
**Control after treatment**	29.76± 0.41

Table S7. The effect of compound **5c** on accumulation of cells at phases of the cell cycle of prostate cancer PC-3 cell line in comparison to control.

Sample data (PC-3)	Results DNA content (%)			
**code**	**G0-G1**	**S**	**G2/M**	**Pre-G1**
**5c**	49.61	44.18	6.21	42.55
Control	41.52	36.29	22.19	2.29

Table S8. Percentage of PC-3 cell death induced by compound **5c.**

Code/ (PC-3)	Apoptosis			Necrosis
	Total	Early	Late	
**5c**	42.55	1.66	26.67	14.22
control	2.29	0.25	0.19	1.85

Table S9. The drug-likeness of compound **5c** and the reference abiraterone drug

Molecule	Lipinski #violations	Ghose #violations	Veber #violations	Egan #violations	Muegge #violations	Bioavail. Score	PAINS #alerts
5c	0	0	0	0	0	0.55	0
Abiraterone	0	0	0	0	0	0.55	0
